# Agarose-resolvable InDel markers based on whole genome re-sequencing in cucumber

**DOI:** 10.1038/s41598-021-83313-x

**Published:** 2021-02-16

**Authors:** Yawo Mawunyo Nevame Adedze, Xia Lu, Yingchun Xia, Qiuyue Sun, Chofong G. Nchongboh, Md. Amirul Alam, Menghua Liu, Xue Yang, Wenting Zhang, Zhijun Deng, Wenhu Li, Longting Si

**Affiliations:** 1 Molecular Biology Laboratory of Jiangsu Green Port Modern Agriculture Development Company, Suqian, 223800 Jiangsu China; 2 Julius Kühn Institute (JKI)–Federal Research Centre for Cultivated Plants, Institute for Epidemiology and Pathogen Diagnostics, Messeweg 11-12, 38104 Brunswick, Germany; 3 Faculty of Sustainable Agriculture, Horticulture and Landscaping Program, University Malaysia Sabah, Sandakan Campus, 90509 Sandakan, Sabah Malaysia

**Keywords:** Molecular engineering, Plant biotechnology, Sequencing

## Abstract

Insertion and Deletion (InDel) are common features in genomes and are associated with genetic variation. The whole-genome re-sequencing data from two parents (X1 and X2) of the elite cucumber (*Cucumis sativus*) hybrid variety Lvmei No.1 was used for genome-wide InDel polymorphisms analysis. Obtained sequence reads were mapped to the genome reference sequence of Chinese fresh market type inbred line ‘9930’ and gaps conforming to InDel were pinpointed. Further, the level of cross-parents polymorphism among five pairs of cucumber breeding parents and their corresponding hybrid varieties were used for evaluating hybrid seeds purity test efficiency of InDel markers. A panel of 48 cucumber breeding lines was utilized for PCR amplification versatility and phylogenetic analysis of these markers. In total, 10,470 candidate InDel markers were identified for X1 and X2. Among these, 385 markers with more than 30 nucleotide difference were arbitrary chosen. These markers were selected for experimental resolvability through electrophoresis on an Agarose gel. Two hundred and eleven (211) accounting for 54.81% of markers could be validated as single and clear polymorphic pattern while 174 (45.19%) showed unclear or monomorphic genetic bands between X1 and X2. Cross-parents polymorphism evaluation recorded 68 (32.23%) of these markers, which were designated as cross-parents transferable (CPT) InDel markers. Interestingly, the marker InDel114 presented experimental transferability between cucumber and melon. A panel of 48 cucumber breeding lines including parents of Lvmei No. 1 subjected to PCR amplification versatility using CPT InDel markers successfully clustered them into fruit and common cucumber varieties based on phylogenetic analysis. It is worth noting that 16 of these markers were predominately associated to enzymatic activities in cucumber. These agarose-based InDel markers could constitute a valuable resource for hybrid seeds purity testing, germplasm classification and marker-assisted breeding in cucumber.

## Introduction

Cucumber (*Cucumis sativus*) is one of the many important creeping vine vegetables. It belongs to the family Cucurbitaceae in the plant kingdom comprising several economically important species including melons, watermelon, squashes, and pumpkins. The Asia continent is being examined as a center for the first domestication as far back as 1500 BC^1,2^. Cucumber has relatively encountered genetic bottleneck all through the era of domestication, thus restricting its genetic background. In allusion to melon, following natural and artificial selection, the genetic variation of cultivated cucumber varieties are greatly reduced^3–5^. In consequence, it is easy to compromise morphological identification of cucumber genotypes due to environmental factors, especially with closely related genotypes. There has been credible advancement in the development of assorted types of DNA markers for a better phenotypic discrimination of cucumber varieties.

Restriction fragment length polymorphisms (RFLP) and amplified fragment length polymorphisms (AFLP) are the earliest types of DNA markers identified through DNA digestion with restriction enzymes, followed by hybridization with isotope-labeled or biotin-labeled probes^6–8^. The disadvantages of these markers were attributed to their time-consuming nature for detection and detrimental environmental effects as narrated by Hu and colleagues^9^. Random amplified polymorphic DNAs (RAPD)^10,11^ and simple sequences repeats (SSR)^12,13^ are markers developed on the basis of Polymerase Chain Reaction (PCR) resolvable by polyacrylamide gel electrophoresis. With reference to cucumber, these two generation of markers have proven a relatively low intraspecific genetic diversity (3–12%) in contrast to other member species of the genus *Cucumis*, consequently influencing their application in high-resolution mapping^14–18^.

The genomic sequence accessibility of Chinese fresh market type inbred line ‘9930’, the North American pickling type inbred line ‘Gy14’ together with the high-quality genome assembly of North European cucumber have served as a furnishing tool in accelerating and facilitating the genome-wide, large-scale development of molecular markers in cucumber^19–21^. Henceforth, a number of cross-species transferable SSR markers have been developed based on draft genome assemblies between cucumber, melon and watermelon^22–27^. Be that as it may, the robustness, informative and user-friendly SSR markers publicly available (10–20%) in intraspecific polymorphism application in cucumber is still limited^28,29^. In addition, accessibility to electrophoresis equipment’s might not be easy for most breeders as it is required for polyacrylamide gel separation in course of developing SSR markers.

Owning to the recent progress in re-sequencing technology, the third generation of DNA markers is based on single nucleotide polymorphism (SNP) and insertion/deletion (InDel) for the production of SNP and InDel markers, respectively^30^. These two categories of markers (SNPs and InDel) are known for their co-dominancy and genome-wide distribution and are importantly exploited in plant genetic studies. Discovery and construction of SNP-based saturated linkage map have been reported in cucumber, which has facilitated the genetic mapping of complex QTL loci controlling cucumber agronomic traits^31,32^. Nonetheless, a genotyping with SNP marker is based on sequencing and/or on SNP arrays, which are rarely performed in most breeding units. SNP genotyping is thereby mostly performed by commercial companies, and this is known to be a time-consuming process for the generation and achievement of a reasonable data. Early this year, Zhang and his team reported a new SNP genotyping in cucumber based on multiplex PCR amplification and high throughput sequencing^33^. InDel molecular markers provide advantages in different fold including high accuracy and high stability which help in deciphering the confusion that may arise in genetic analysis as compared to other length polymorphic markers. Moreover, they can amplify target fragments from mixed or highly degraded DNA samples^34^. InDel markers have been developed for several crops including cotton, rice, maize, rapeseed, cucumber etc^35–41^. It is clear that some polymorphic InDel markers were previously developed for genotyping of cucumber and has been applied on 6 typical cucumber germplasm^42^ nonetheless; they were all resolvable on polyacrylamide gel due to the InDel sizes.

Based on our knowledge and technical know-how, the use of agarose gel electrophoresis in genotyping is easily accepted by breeders due to its simple requirements and easy operation in the laboratory compared to polyacrylamide electrophoresis. We therefore, deemed it significant that developing Agarose resovable InDel markers will be a mile stone in marker development for breeders in breeding programs. This will ease and increase efficiency of plants genotyping by accelerating the procedure during breeding programs. Although, InDel markers can be developed for both polyacrylamide and agarose gel electrophoresis depending on their sizes^36^. High-density Insertion/Deletion is needed which could be exploited for the discovery of valuable InDel markers for genotypes screening through agarose gel rather than polyacrylamide gel^40^. Thus, availability of a large number of genome-wide InDel makers is essential to reach this goal.

In this study, we developed agarose-resolvable InDel markers through re-sequencing whole genomes and identify genetic variation (insertion and deletion) for the breeding parents X1 and X2 of cucumber hybrid variety Lvmei No.1 compared to the reference genome sequence of Chinese fresh market type inbred line ‘9930’. The development and detection of these InDel markers relied greatly on the separation of their PCR products by agarose gel electrophoresis, investigation on their polymorphism and crossed-parents transferability. The later was conducted using varied number of breeding parent including five (5) pairs for cucumber, three (3) pairs of melon and two (2) pairs of watermelon. A total of forty-eight (48) cucumber breeding lines were evaluated for PCR amplification versatility and phylogenetic analysis using the developed InDel markers. These InDel markers are regarded as useful genetic reservoir for genotypes identification, genetic diversity analysis, hybrid testing and marker assisted selection in cucumber breeding programs.

## Materials and methods

### Plant materials

**Figure 1:**
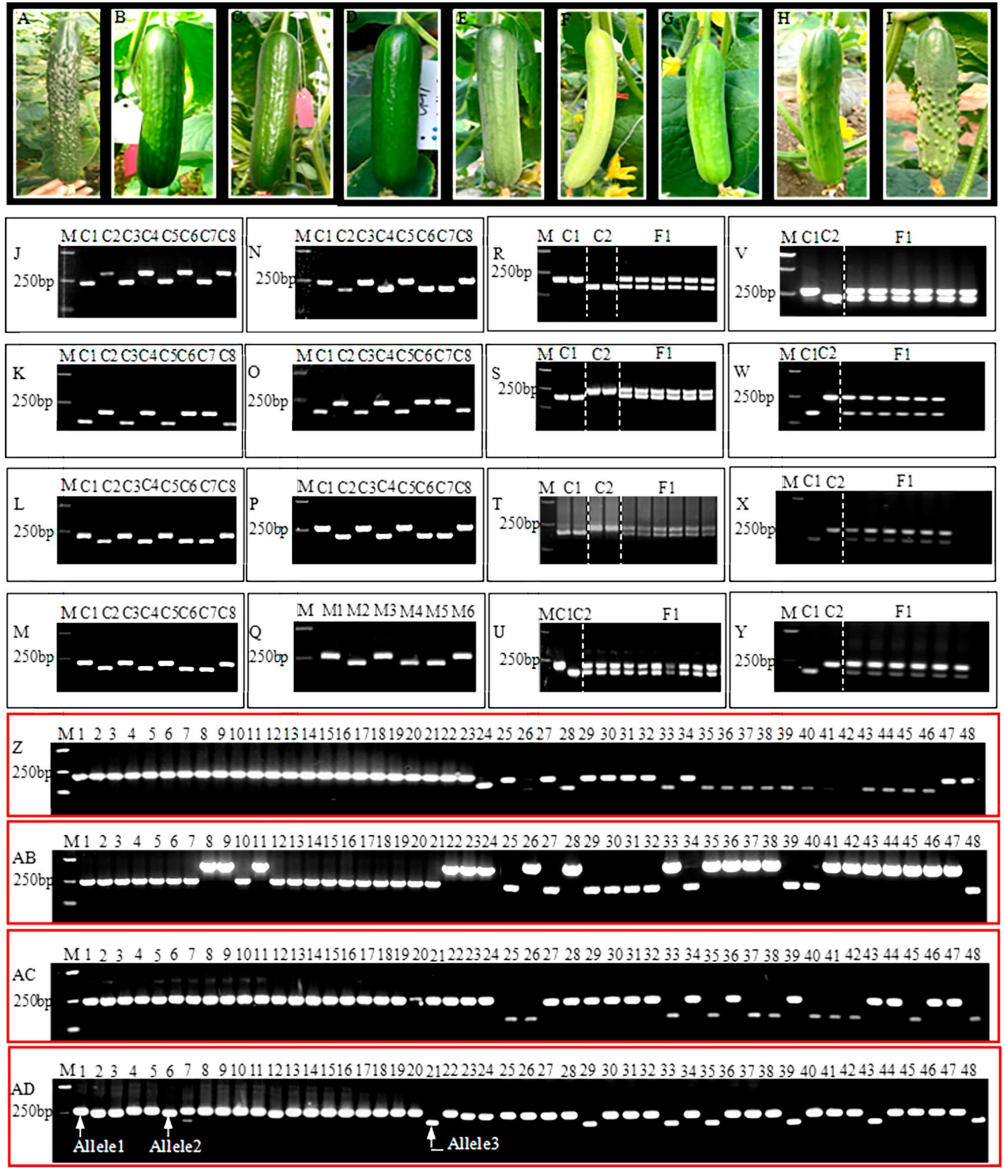
A display of cucumber lines fruits morphology and agarose gel resolvability of lnDel markers. Dense spiny cucumber (**A**), female parent (**B**) and male parent (**C**) of Lvmei No.l hybrid variety, Lvmei No.l hybrid variety (**D**), f ruit cucumber (**E**), White-green and black spiny cucumber (**F**), White green cucumber (**G**), White Green sparsely spin y cucumber (**H**), Yellow Green sparsely spiny cucumber (**I**). Agarose gel electrophoresis monograph for poly morphism analysis (**J**–**Q**); hybrid seeds purity detection (**R**–**Y**) and validation of lnDel markers for 48 cucumber breeding lines (**Z**–**AD**); polymorphism analysis using lnDell69 (**J**), lnDel79 (**K**); lnDel115 (**L**); lnDel117 (**M**), lnDel124 (**N**); lnDel170 (**O**); and In Del114 (**P**–**Q**); respectively; Hybrid seeds purity testing of the elite cucumber hybrid va riety L vmei No.l using InDel79 (**R**); InDel170 (**S**), InDel114 (**T**–**U**), InDel124 (**V**), lnDel232 (**W**), InDel269 (**X**), and InDel48 (**Y**); respectively; Experimental validation of 48 cucumber breeding lines using In Del161 (**Z**); lnDel174 (**AB**), lnDel232 (**AC**), and lnDel269 (**AD**), X1 and X2 are the parents of elite cucumber hybrid variety Lvmei No.l while C1/C2, C3/C4 and C5/C6 are 3 couples of other three cucumber commercial hybrids while M1/M2, M3/M4 and M5/M6 constitute 3 couples for three commercial melon hybrids; F1 represent individuals of Lvmei No.l; Allele 1, Allele2 and Allele3 are three different allele generated from the 48 lines using InDel269; l = Dl, 2 = D2, 3 = D3, 4 = D4, 5 = D5, 6 = D6, 7 = D7, 8 = D8, 9  =  D9, 10 = D10, 11 = D11, 12 = D12, 13 = D13, 14 = D14, 15 = D15, 16 = D16, 17 = D17, 18 = D18, 19  = D19, 20  = D20, 21 = D21, 22 = D22, 23 = D23, 24 =  B1, 25 =  B2, 26 =  B3, 27 =  B4, 28 =  B5, 29 =  B6, 30 =  B7, 31 =  B8, 32 =  B9, 33 = X1, 34 = X2, 35 = X3, 36 = X4, 37 = X5, 38 = X6, 39 = X7, 40 = X8, 41 = X9, 42 = X10, 43 = X11, 44 = X12, 45 = X13, 46 = X14, 47 = X15, 48 = X16; M represents DL 2000 bp DNA Marker. The uncropped full-length raw gel pictures are included in Supplementary Figure S1.

Two cucumber lines designated as X1 and X2 constituting the parents of an elite commercial hybrid Lvmei No. 1 were used for whole genome re-sequencing (Supplemental Table S1A). These breeding parents are derivative of three different commercial varieties 22–403, Zhongnong No. 26 and HA-414. The hybrid varieties 22–403 and Zhongnong No. 26 developed by RijkZwaan Company and Institute of Vegetables and Flowers of the Chinese Academy of Agricultural Sciences (CAAS, respectively. Hybrid variety 22–403 is prominent for its good taste, cold tolerance trait, and year-round greenhouse cultivation while strong growth potential, comprehensive diseases resistance and tolerance to low temperature and light intensity characterize Zhongnong No. 26. The third is the fruit cucumber variety HA-414 which was imported from Israel. Crossing between 22 and 403 and Zhongnong No. 26 accompanied by seven generations of selfing gave rise to the male parent of Lvmei No. 1 (X2). This variety (X2) is prominent for its late-maturity in addition to its outstanding growth character, sweet fruit and high resistance to downy mildew. The female parent (X1) was obtained after six generations of selfing of HA-414. X1 is distinguished for its early maturity, with relative resistance to downy mildew incorporated with sweet and good fruit set. Moreover, pairs of cucumber C1/C2, C3/C4, C5/C6, C7/C8; melon M1/M2, M3/M4, M5/M6; watermelon W1/W2 and W1/W2 commercial hybrids were utilized for cross-parents polymorphism analysis (Suppl. Table S1B). Forty-eight cucumber breeding lines, classified into three groups (1, 2 and 3) grounded on their fruit morphology, were used for InDel markers versatility and phylogenetic analysis. Group 1 and 2 composed of 23 dense spiny cucumber lines and 18 fruit cucumber lines, respectively. Group 3 constituted 6 white-green sparsely spiny and 2 yellow-green sparsely spiny cucumber lines (Suppl. Table S1). The representative fruit morphology for each group of cucumber lines including X1 and X2, and hybrid variety LvmeiNo.1 are detailed in Fig. 1A–I. All the plants were grown in solar greenhouse of Jiangsu Green Port Modern Agriculture Development Company.

### Library construction and sequencing

CTAB extraction method was used to isolate genomic DNA from fresh leaves of 30 days old X1 and X2 plants maintained in the greenhouse. The quality of extracted DNA was evaluated by electrophoresis, running an aliquot of 5 µl on a 1% agarose gel and concentration measured with Nanodrop spectrophotometer 2000 (Thermo Scientific, USA). Samples whose genomic DNA measured more > 10 ng/µL at an OD260/280 with values between 1.8 and 2.0 were considered for library construction. Initially, genomic DNA was sheared using an ultrasonic Crusher (Ultrasonic Crusher Q800R3, Qsonica Co Ltd, USA) to yield an average DNA fragments of about 350 base pair (bp). These fragmented DNA samples were cleaned up using AMPure XP beads (http://www.beckmancoulter.cn) and freshly prepared 80% Ethanol according to manufacturer’s protocol. Subsequently, DNA ends repair, library size selection, adenylation, Illumina paired-end adapter’s ligation to fragmented DNA were performed successively. The ligated DNA products were selected and amplified. Two paired-end libraries with 15-fold depth for each cucumber breeding line was constructed using TruSeq DNA LT Sample Prep kit. The resulting libraries were sequenced on an Illumina Hiseq X Ten, PE150sequencer (Shanghai OE Biotech. Co. Ltd, China).In this work, all steps were conducted according to OE Biotech Company deep sequencing protocol (Shanghai OE Biotech. Co. Ltd, China).

### Data filtering, alignment and variants calling

*Cucumis sativus* L. var. *sativus* cv. Chinese fresh market type inbred line ‘9930’ genome sequence was obtained from cucurbit genomics database (CuGenDB) (ftp://cucurbitgenomics.org/pub/cucurbit/genome/cucumber/Chinese_long) and was used as the reference sequence. Low quality reads data were filtered out using a custom C program based on the default parameters to recover clean reads data. The cleaned reads data were aligned to the reference genome with the help of BWA (BWA0.7.10-r789) program^43^. The alignment output results in SAM format were then converted into Binary Alignment Map (BAM) files format using SAMTools^44^. Mark Duplicates in Picard tool (v1.102)^45^ was applied to remove replicates reads and the two BAM files were used for subsequent analyses. Local realignment, InDels filtering and calling were performed using a bioinformatics software Genome Analysis Tool Kit (GATK) version 3.1 (https://gatk.broadinstitute.org/hc/en-us).

### InDels flanking sequences and primers designing

Polymorphism analysis was performed following the protocol described by Guo et al.^46^ with slight modification for establishing InDel polymorphisms between the re-sequenced X1 and X2. To find out InDel polymorphisms between the re-sequenced X1 and X2, we explored reference genome sequence of the Chinese fresh market type inbred line ‘9930’. The sequence reads for X1 and X2 were aligned to the reference sequence individually through the Short Oligo-nucleotide Alignment Program (SOAP) software^47^ with no gaps counts. The aligned reads dataset of X2 was compared to the InDel polymorphism dataset obtained upon mapping of X1 to the reference genome sequence. Only those InDels with identical sequences arising from comparison with the Chinese fresh market type inbred line ‘9930’ were regarded as real InDels for X1 and X2. Once the location of InDel polymorphisms for one re-sequenced parent and reference genome sequence was established, those between the two re-sequenced parents became readily distinguishable at corresponding positions where the second parent is identical to the reference sequence. To develop InDels markers, 150-nucleotides sequences flanking both ends of an Insertion/Deletion site were extracted. A simple Visual C++ script helped in fishing out these sequences from the reference genome sequence. The sequences then served as templates for primers designing. Primer 5 (http://www.PromerBiosoft.com) was used to design PCR primers with a varied range of properties (length of 18–28 bp, Tm of 57–63 °C, and PCR products of 80–300 bp).

### DNA extraction and polymerase chain reaction

NuClear Plant Genomic DNA Kit (CWO531M) (CW Biotech, Beijing, China) was used for total DNA extraction from the fresh leaves of 30-day old cucumber, melon and watermelon plants (maintained under greenhouse condition) according to manufacturer’s recommendation. The extracted DNA concentration was measured using Nanodrop spectrophotometer 2000 (Thermo Scientific, USA) and adjusted to a final concentration of 50 ng/µl. A total volume of 25 µl PCR reaction mix was prepared by composing 12.5 µl 2x*Taq* Master Mix plus loading buffer (CW Biotech, Beijing, China), 1 µl forward, 1 µl reverse primer at a concentration of10 µM, 1 µl of DNA extract (50 ng/µl), and 9.5 µl of nuclease free water. Amplification reaction conditions were as follows: initial denaturation at 94 °C for 2 min, 35 cycles of denaturation at 94 °C for 30 s, annealing at 55 °C for 30 s, and extension at 72 °C for 30 s followed by 72 °C for 2 min. The PCR products were separated on a 2% agarose gel in 0.5× TAE buffer, stained with ethidium bromide (EB) and visualized (UV) by Ultraviolet light at 300 nm using Gel imaging analyzer (WD-9413C, Bejing, China).

### Cloning and sequencing

PCR products from X1 and X2 using a cross-species transferable marker InDel114 and cross-parent transferable marker InDel79 were purified and ligated onto Psimple-19 Ecorv/BAP Vector. Constructs (Psimple-19 EcorV/BAP—PCR fragment) were transformed into *E. coli* competent cells (DH5α) followed by PCR verification with KOD FX enzyme (Toyobo Co., LTD, Japan). A 10 µl PCR reaction contained 1 µl of transformed bacterial cell culture in solution, 5 µl of 2× PCR Buffer for KOD FX, 0.5 µl of forward primer M13-F and reverse primers InDel114-R and InDel79-R (10 µM), 1 µl of dNTPs (2 mM), 0.2 µl of KOD FX enzyme (1 units/µl) and 1.8 µl of double distilled water (ddH_2_O). PCR products were separated on agarose gel, stained by EB and visualized by UV using Gel imaging analyzer. The positive clones were commercially sequenced by Hangzhou Shangyasai Biotechnology Co. Ltd. The sequenced fragments nucleotide sequences were aligned using the DNAMAN software (http://www.lynnon.com/).

### Genomic location of InDels

To predict the locus of the 68 InDel markers on the genome, the primer sequences were used as query for a blast search in Gramene database (http://ensembl.gramene.org/Multi/Tools/Blast?db=core) as compared to the reference sequence. The sequenced PCR products obtained using InDel114 and InDel79 primers were used to predict genomic location of these markers. NCBI (National Center for Biotechnology Database) database (https://www.ncbi.nlm.nih.gov/) and UNIPROT (https://www.uniprot.org/uniprot/Q56X52) web based tools were successively used to obtain genes description and function of their derived loci.

### Phylogenetic analysis

The amplified PCR products from the 48 breeding lines using 39 Cross-Parents Transferable (CPT) polymorphic markers that cover 6 cucumber chromosomes were separated on agarose gel. The obtained amplicons were scored according to the type of software deployed for the phylogenetic analysis. Power Marker (PM) version 3.25^48^, is capable of exploiting quantitative data linked allele size (i.e., molecular weight of allele) and was thus exploited for the aforementioned purpose. It was used to obtain the number of alleles per locus, major allele frequency, gene diversity, values for polymorphism information content (PIC) and to calculate Nei’s distance between breeding lines^49^. Phylogenetic tree was constructed with reference tothe Nei’s minimum genetic distance based on UPGMA (unweighted pair group method using arithmetic average) method and tree visualized with the help of MEGA 5.0 software (http://megasoftware.net/). NTSYS 2.1^50^ and Darwin (i.e. Dissimilarity Analysis and Representation for Windows) version6 (http://Darwin.Cirad.fr), were used for qualitative data analysis. The absent or present of allele was represented by 0 and 1, respectively.

## Results

### Identification and classification of genome-wide Insertion/Deletion event

The clean reads quantity generated was 187,403,732 for X1 and 153,584,502 for X2 recording with an average of 170,494,117. Using the Burrows–Wheeler Alignment (BWA), 182,456,273 and 149,529,871 (average 165,993,072) reads from X1 and X2, respectively were both mapped at a depth of 15 to the reference genome sequence of Chinese fresh market type inbred line ‘9930’. The overall genome coverage was 97.30% for X1 and 97.74% for X2 hitting an average of 95.8% (Table 1). Genome-wide insertion/deletion polymorphism generated 198,169 InDels between X2 and 9930 measuring an InDels density of 939.2 InDels/Mb. The distribution of these InDels was among the 7 (seven) chromosomes with variation in number recorded as follows: 16959 on chromosome07, 45372 on chromosome03, 704.5 on chromosome05 and 1150.7 on chromosome02. Comparison of aligned reads between X1 and X2 produced an average of 10,470 InDels and a density 49.6 InDels/Mb. These InDels span across the seven cucumber chromosomes with chromosome07 recording the least (694) and chromosome03 the highest (2289). There was equally a variation in the density that ranged from 30.9 InDels/Mb on chromosome7 to 68.7 InDels/Mb on chromosome02 (Table 2). In regards to the length of the nucleotide sequence, 3 types of Insertion/Deletions were noticed and categorized as small, medium and large InDels. The differences in the number of insertions and deletions for each type of InDel are minimal. The large, medium and small InDels accounted for 11.1%, 35% and 53.9% of the total genome-wide InDels, respectively (Table 3).

**Table 1:** Detail information of cucumber hybrid variety Lvmei No.1 parents, X1 and X2.

Samples	Clean reads	Map ratio (%)	Cover ratio (%)	Q20 (%)	Depth
X1	187,403,732	97.36	97.30	97.05	15
X2	153,584,502	97.36	97.74	96.65	15
Average	170,494,117	97.36	97.52	96.85	15

**Table 2:** Initially traced polymorphicInDel on X1 and X2 individual chromosomes with X2.

Chr. types	Chr. PD (Mb)	X2 versus 9930		X1 versus X2	
		InDels number	Frequency (InDels/Mb)	InDels number	Frequency (InDels/Mb)
Chr1	32.93	29,124	884.4	1734	52.7
Chr2	24.84	28,584	1150.7	1707	68.7
Chr3	40.88	45,372	1109.9	2289	56.0
Chr4	26.83	27,208	1014.1	1745	65.0
Chr5	31.91	22,479	704.5	1111	34.8
Chr6	31.13	28,438	913.5	1190	38.2
Chr7	22.47	16,959	754.7	694	30.9
Total	210.99	198,169	939.2	10,470	49.6

Chr.: Chromosome; Chr. PD: chromosome physical distance; Mb: megabase-pairs.

**Table 3:** Ranking of genome-wide InDels based on the length of nucleotide sequence.

Type	Length (bp)	Deletion	Insertion	Total	Percentage (%)
Small	1–10	2834	2849	5683	11.1
Medium	11–20	1412	1346	2758	35.0
	21–30	500	440	940	
Large	> 30	598	573	1171	53.9
Total		5344	5208	10,552	
Probability difference (InDel)		0.97			

### Agarose-resolvable InDel markers for X1 and X2

The 10,470 InDels distributed over the seven-cucumber chromosome were chosen for the development of PCR-based markers. The target fragment length of 300 nucleotides for X1 and X2, known to harbor the corresponding Insertion/Deletion sites, were utilized as templates for primers designing (Suppl. Table S2). With respect to the electrophoresis method applied, the polyacrylamide gel electrophoresis InDels PCR-based markers were consider to be those that falls under small and medium InDels type and those belonging to large InDels type (with insertion/deletion size greater than 30 bp) were categorized as agarose gel PCR-based markers. A total of 1171 PCR-based markers were identified with a variation in the PCR products size ranging from 80 to 300 bp (Table 3). Out of 1171, the arbitrarily selected 385 candidate markers with an average density of 1.8 InDels/Mb were subjected to experimental validation (Supplemental Table 3). The InDels markers with an average density of 1.0 InDels/Mb that produced a single amplicons with clear polymorphism between X1 and X2 accounted for 54.81% (211) of the 385 selected candidates. These 211 PCR-based InDels markers were recognized and considered as agarose-resolvable InDels markers (Suppl. Table S4). On the other hand, 174 representing 45.19% showed unclear or monomorphic bands under our PCR conditions.

### Identified cross-parents and cross-species transferable InDels markers

Five (5) pairs of cucumber breeding parents were implicated in polymorphism analysis for validation of the considered agarose-resolvable InDels markers. Sixty eight (68) InDel markers with an average density of 0.3 InDels/Mb revealed polymorphism between more than 2 pairs of cucumber breeding parents (Suppl. Table S5) and were designated as cross-parents transferable (CPT) Indels markers. Markers with remarkable polymorphic patterns between more than 4 matches of breeding parents included InDel79, InDel115, InDel117, InDel124, InDel169, InDel114 and InDel170 (Fig. 1J–Q, Suppl. Figure S1, and Table 4). The cross-species transferability evaluation with these markers was extended to three pairs of breeding parents from melon and two pairs from watermelon for polymorphism test. Interestingly, InDel114 and InDel177 expressed polymorphism between the breeding parents derived from melon (Fig. 1J–Q) and from watermelon (Data not shown). To be brief, the number of agarose-resolvable InDel markers recorded mark ranges from zero on chromosome07 to 59 on chromosome02 (Suppl. Table 5). However, that of the cross-parents transferable markers varied from 0 to 44 on chromosome07 and 02, respectively while that of cross-species transferable InDel markers (with restricted variation) extended from zero on chromosome02, 04, 05, 06, and 07 to two on chromosome03. Though the highest number of the InDels between X1 and X2 was recorded for chromosome03, chromosome02 was predominant for agarose-resolvable and CPT InDel markers (Suppl. Table 5).

**Table 4:** Polymorphism information of cross-parents and cross-species transferable InDel markers.

Species	Hybrid varieties (breeding parents)	Strongly polymorphic (CPT) Indels markers					Cross-species InDel markers	
		79	115	117	124	169	114	170
Cucumber	LvmeiNo.1 (X1&X2)	√	√	√	√	√	√	√
	C96/C1396 (C1&C2)	√	√	√	√	√	√	√
	C87/C360 (C3&C4)	√	√	√	√	√	√	
	C38/D18 (C5&C6)	√	√	√	√	√	√	√
	D52/C335 (C7&C8)					√		√
Melon	M13065-1/39-1-1–1-1 (M1&M2)						√	
	M13065-1/17065-2-1(M3&M4)						√	
	M170892/7305 (M5&M6)						√	
Watermelon	PK2-15/Q204 (W1&W2)							√
	PK2-18/74-1-3 (W3&W4)							

### Hybrids purity evaluation using InDel markers

In order to answer the question of how performing these markers were on evaluating hybrid seed purity, seven of the InDel markers were selected for this purpose. These markers included InDel114, InDel79, InDel170, InDel124, InDel232, InDel269 and InDel48 for genotyping of the hybrid seedlings of Lvmei No.1 variety and its corresponding parents X1 and X2. Herein, InDel114 which was shown to be transferable between cucumber and melon was used to test seeds purity for corresponding melon hybrid variety. The outcome proved that these markers were capable to perform hybrid seeds purity evaluation (Fig. 1R–Y, Suppl. Figure S1). The sequenced DNA fragment for accuracy confirmation of InDel114 yielded an amplicon of 191 nucleotides fragment length in cucumber male parent and 218 bp for female parent. Further, these two DNA fragments (191 bp and 218 bp) were simultaneously amplified from the F1 individuals (Fig. 1P, Suppl. Figure S2). Similarly, in melon, InDel114 generated 217 bp fragment length from male parent and 253 bp from female parent as well as from the F1 individuals (Fig. 1Q, Suppl. Figure S2). On the other hand, InDel79 amplified 204 bp fragment length from male parent and 151 bp from female parent accompanied with the detection of both DNA fragment in F1 individuals as resolved by agarose gel electrophoresis (Fig. 1R, Suppl. Figure S2).

### Validation and application of InDel markers

To determine the PCR amplification versatility of these CPT InDel markers, 68 primer pairs were used to amply the InDels from 48 cucumber breeding lines. These set of primers were categorized based on the PCR amplicons from agarose gel electrophoresis. Of these primers, both 29 pairs were not efficient by amplifying partial or not at all the targeted sequence from the 48 breeding lines and such primers were thus excluded from this work. Thirty-nine (39) of these primer pairs generated at least two alleles among the 48 breeding lines as presented in Fig. 1Z, AB, and AC for Indel161, Indel174 and Indel232 respectively, with an exception of Indel269 that generated three alleles per locus (Fig. 1AD, Suppl. Figure S1). However, non-group specific PCR amplicon was recorded for 27 pair of markers whereas 12 showed specific amplification tendency for group1 breeding lines. Among these 12 InDel markers, InDel227, InDel232, InDel265, InDel161, InDel172, InDel217, InDel48, InDel62, and InDel277 amplified a single identical allele from breeding lines in group1 while InDel markers InDel225, InDel265, InDel171 and InDel41 generated a single identical allele from breeding lines in group3. InDel269 could separate breeding lines in group1 from those in group2 and group3 except for some six breeding lines in group1 (Fig. 1AD).

### Polymorphism based on InDel markers

The allele frequency value of the 39 InDel markers used for phylogenetic analysis was between 0.50 for InDel269 and 0.88 for InDel265. Allele number of two (2) was observed for 38 InDel markers and three (3) for the InDel269 marker with an average of 2.03. The registered genetic diversity ranged from 0.22 for InDel48 to 0.60 for InDel269 with an average value of 0.40. Apart from InDel161with a heterozygosity score of 0.21, 38 others recorded zero making an average of 0.01. The least PIC value was 0.20 and highest 0.52 as recorded by InDel48 and InDel269, respectively marking an average of 0.32 (Suppl. Table 6). Majority of these markers (~ 72%) recorded a PIC value comprised between 0.30 and 0.52. The average PIC value for the night InDel markers that showed more specificity to group1 individuals registered an average PIC value of 0.26 but the average PIC value for four InDel markers with high affinity for breeding lines in group3 was 0.31. None group-specific InDel markers were 26 in number with an average PIC value of 0.33. We found any of these InDel markers being specific for breeding lines in group2.

### Phylogenetic analysis of cucumber breeding lines

**Figure 2:**
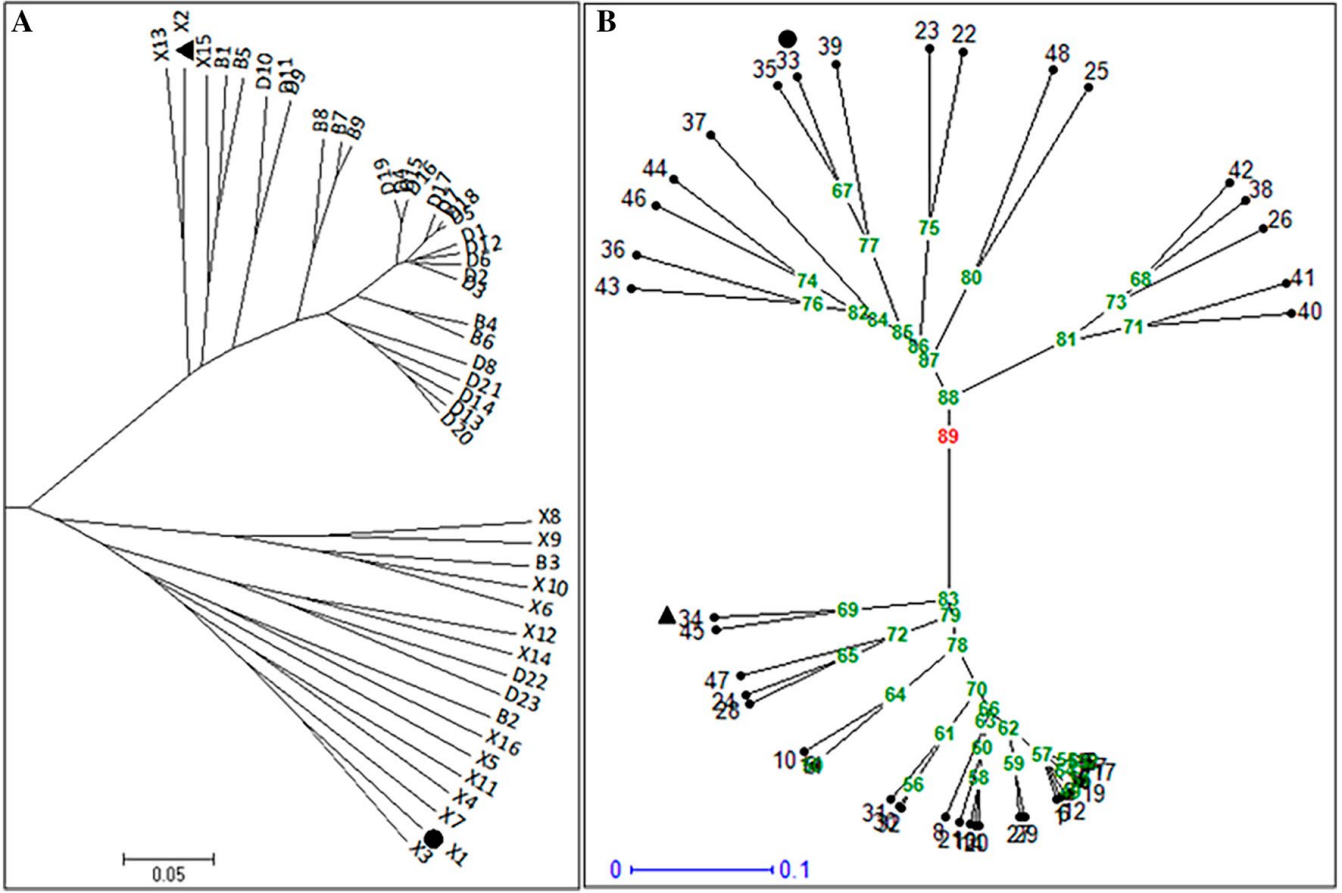
Radial display of phylogenetic trees constructed based on 39CPT InDel markers. Phylogenetic tree constructed using Power Marker (PM) version 3.25 software (**A**) and Darwin version6 software (**B**). The complete genome re-sequenced parents Xl and X2 of elite cucumber Lvmei No. 1 hybrid variety is highlighted with triangular and round shape in black color. The red colored number in (**B**) indicates genetic similarity score at root node of the tree. Numbers at each node in (**B**) are genetic similarity scores. In B: l = Dl , 2 = D2, 3 = D3, 4 = D4, 5 = D5, 6 = D6, 7 = D7, 8 = D8, 9 = D9, 10 = D10, 11 = D11, 12 = D12, 13 = D13, 14 = D14, 15 = D15, 16 = D16, 17 = D17, 18 = D18, 19 = D19, 20  = D20, 21 =  D21, 22 = D22, 23 = D23, 24 =  B1, 25 =  B2, 26 =  B3, 27 =  B4,  28 =   B5,  29 =   B6, 30 =  B7, 31 =  B8, 32 =  B9, 33 = X1, 34 = X2, 35 = X3, 36 = X4, 37 = X5, 38 = X6, 39 = X7, 40 = X8, 41 = X9, 42 = X10, 43 = X11, 44 = X12, 45 = X13, 46 = X14, 47 = X15, 48 = X16. The scale bars represent the evolutionary time unit.

Phylogenetic analysis results categorized the 48 breeding lines into two clusters designated as cluster I and cluster II. Cluster I constitutes 17 breeding lines of which 71% of them belong to groupe 2 with majority of the individuals being fruit cucumber breeding lines of the female parent X1 for Lvmei No.1 hybrid variety (Fig. 2, Suppl. Figure S3). Cluster II comprises of 31 cucumber breeding lines. This includes individuals of group1 and group3 and the male parent X2 of Lvmei No.1 hybrid variety (Fig. 2, Suppl. Figure S3). In brief, the 39 InDel markers could differentiate fruit cucumber varieties from the common cucumbers with few exceptions. Power Marker software obtained results were further validated using Darwin and NTSYS softwares (Suppl. Figure S3). Intriguing, the pairwise genetic distance between parents X1 and X2 was the highest as revealed by Nei’s genetic distance value of 0.96 (Suppl. Table S6).

### Physical position and genomic location of the CPT InDel markers on cucumber chromosomes

**Figure 3:**
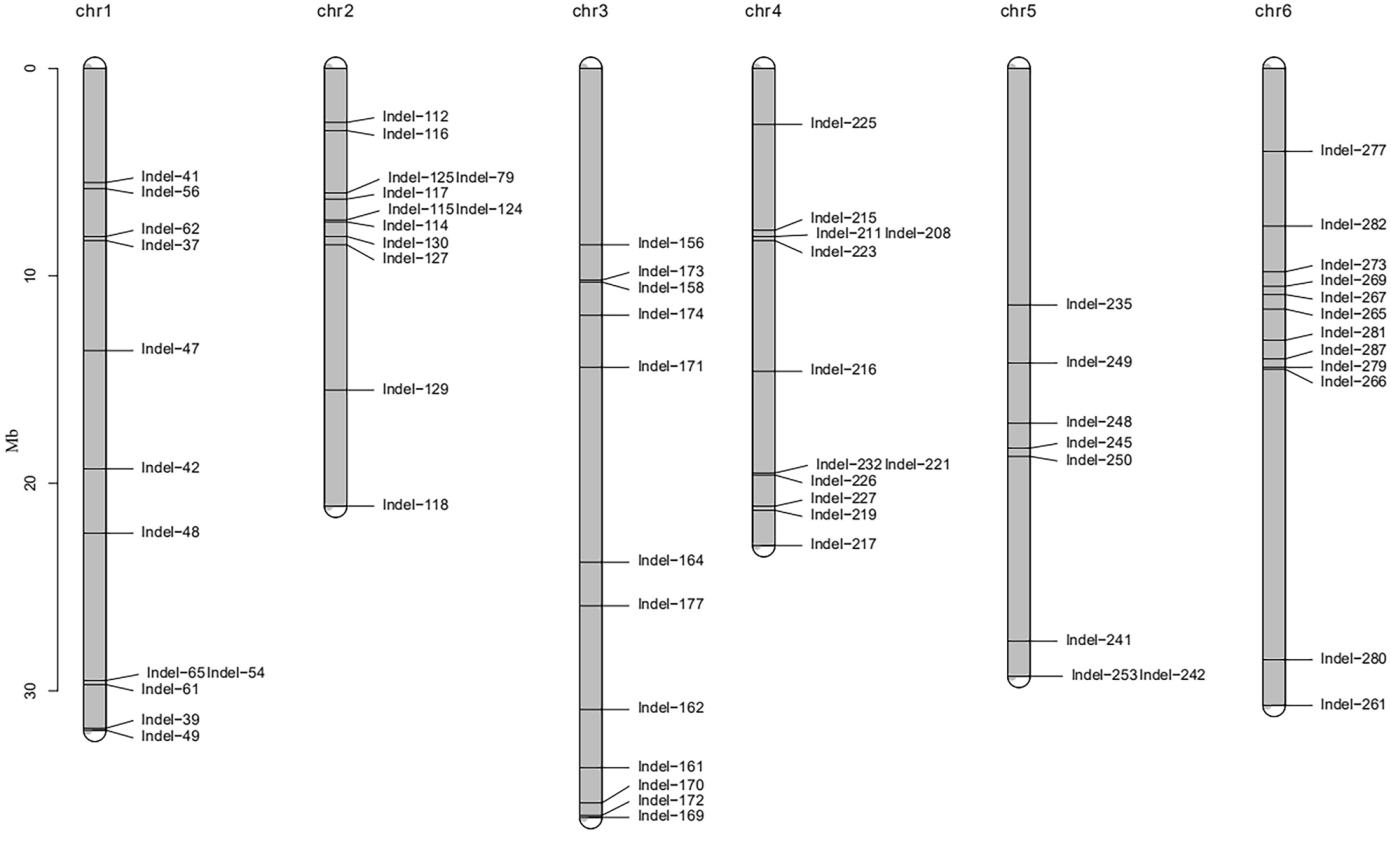
Physical map showing the distribution of the 68 CPT InDel markers on six chromosome of cucumber.

Physical map illustrated the corresponding positions of the 68 CPT InDel markers (Fig. 3). These markers showed distribution across all the cucumber chromosomes, except for chromosome07, where a relatively small number of agarose-resolvable markers was originally selected. In order to localize these CPT InDel markers on the genome of cucumber, a blast search was performed from three different database platforms including gramene database, NCBI and UNIPROT. Our search generated 16 InDel markers with position either in the exon or in the intron of certain genes. The description and molecular function of these genes were recorded which associated them to different potential functional activities. Among these 16 InDel markers, seven was related to oxidoreductase activity, three to hydrolase activity (two for membrane trafficking and one for DNA replication), one for RNA binding, one for protein biosynthesis and one for transferase activity (Suppl. Table S5).

## Discussion

Myriad of activities related to domestication, natural and artificial selections have considerably restricted the genetic variation of cultivated cucumber varieties. The identification of cucumber genotypes was traditionally performed based on morphologically observed characteristics. Unfortunately, this approach is not definitely efficient as plant morphology is easily influenced by environmental factors and in special cases of closely related genotypes. In order to circumvent this short coming, different types of DNA markers have been developed for better segregation of cultivars in cucumber breeding programs. Nowadays, SNPs, polyacrylamide-resolvable InDels and SSR markers are the available and mostly used approaches in cucumber against RFLPs, AFLPs and RAPD markers. Notwithstanding, genotyping using SNP requires a relatively complex platform coupled with the fact that electrophoresis facilities of polyacrylamide-resolvable InDels and SSR markers are relatively expensive. Strikingly, an alternative is the possibility of developing InDel markers for both polyacrylamide and agarose gel electrophoresis with dependency on the size of insertion/deletion as mentioned by Liu et al.^36,37^. Recent agarose-resolvable InDel markers approach was successfully developed for rice^9^. Though major breakthrough has been made in the discovery of SSR markers as well as the most recent efforts deployed in the development of InDel markers in cucumber^20,42^, no information is provided regarding their agarose gel resolvability during electrophoresis. Unlike the polyacrylamide gel, breeders readily accept the agarose gel electrophoresis due to its simplicity in term of usage and accessibility of required facilities.

In this study, agarose-resolvable InDel markers were developed based on whole-genome re-sequenced data of cucumber breeding parents X1 and X2. With the concern of not discovering suitable InDel markers due to the restricted genetic variation of cucumber, a relatively higher genome coverage ratio and sequencing depth were applied. A total of 10,470 InDel markers were developed on seven cucumber chromosomes with exclusion of those that are not anchored to any chromosome. More than a thousand InDels with insertion/deletion differences equal to or more than 30 bp were chosen. In order to optimize time and scale up cost effectiveness, 385 markers were selected in this study for experimental validation by agarose gel electrophoresis. Among these markers, 211 generated single PCR products (range 80 to 300 bp) with clear polymorphism resolvable on a 2% agarose gel. In the year 2015, Liu and colleagues developed and reported InDel markers for rice of which the PCR products varied between 150 and 300 bp resolvable on a 3.5% agarose gel over a long duration of electrophoresis^36^. It is obvious that the large fragment insertions/deletions (InDel) of 30–55 bp differences can be exploited to amplify DNA fragment of 300–350 bp which are easily separated on 1.5–2% agarose gel^9^. Sixty-eight among the one hundred and eleven (211) InDel markers with clear polymorphism displayed polymorphism between the breeding parents of more than 2 cucumber commercial varieties, thus depicting them as cross-parent transferable (CPT) InDels markers in hybrid seeds purity test. There is the tendency that these markers can serve as an important tool for rapid detection of seed purity and accession of genetic diversity in cucumber. A cross-species polymorphism was noticed with three of these InDel markers, but only InDel markers InDel114 exhibited transferability between cucumber and melon as successful shown by our experiment.

In the past, molecular markers transferability has been reported between cucumber, melon and watermelon. With emphasis on SSR markers and upon completion of draft genome assembly for these three crops^19,20,23,51^, a large number of cross-species transferable SSR markers have been developed. This had equally open an avenue for establishing a syntenic relationships among them^19,22–24^. The transferability aspect has been reported in previous works stressing that there is a close relation between cucumber and melon than between cucumber and watermelon. For example, an in silico PCR analysis using melon SSR markers resulted to the identification 4002 amplicons between cucumber and melon while 1085 were found between watermelon and melon^27^. Specific genomic regions have been defined using SSR products to reveal sequence homologies between cucumber and melon^52,53^. Moreover, SSR markers developed from melon have been used routinely in cucumber genetic mapping studies and vice versa^54,55^. It is speculated that this tendency might be due to the fact that the specification of watermelon has occurred earlier in Cucurbitaceae family^56,57^. Contrary, melon and cucumber diverged from a common ancestor approximately ten million years ago^2,58^. The five cucumber chromosomes arose from fusions of ten melon ancestral chromosomes after divergence^19^ and chromosomes syntenic between melon and cucumber are less complicated than that between melon and watermelon^27^. Explicitly, the low discovering or failure of obtaining polymorphic cross-species transferable InDel markers between cucumber and melon or watermelon could be related to: few (3.68%) developed InDel markers were subjected to experimental validation; some InDel markers have amplified cross-species genetic bands but with no polymorphism between the pairs of parents, thus hampering their selection as we were concerned with polymorphic transferable markers; the number of pairs of parents used for polymorphism analysis might be insufficient and the development of polymorphic cross-species transferable markers was proceeded by initially evaluating their polymorphism in cucumber and then validating the cross-transferability of this polymorphism in melon or watermelon. Our approach is reversed as compared to that previously applied by Zhu and colleagues^27^. Here, the unexploited 10,085 markers could constitute a potential reservoir of agarose-resolvable InDels which require an experimental validation. However, the full exploitation of the 10,470 InDel markers, together with an increase of pairs of breeding parents subjected to polymorphism analysis in future experimental work might go a long way to increase the cross-species transferable InDel markers. The cross-species transferable markers could be useful in map construction, comparative mapping, and genetic diversity analysis in closely related species of cucurbit crops. The ability of these agarose-resolvable InDel markers in PCR amplification versatility and evolutionary relation detection are demonstrated in a panel of 48 cucumber selection lines. Here, these markers can effectively be used for research on genetic diversity and phylogenetic relationships in cucumber. Impressively, they could clearly segregate the breeding lines in two principal clusters with clusterI composed mainly of dense spiny cucumber (group1) and clusterII fruit cucumber varieties (group2). The breeding lines of group3 were distributed between individuals in clusterI and II, indicating that they share some similarities with genome fragments from dense spiny and fruit cucumber.

On the basis of genomic location of these markers, we speculated that the loci harboring these InDel markers or their closely related genomic regions may be those participating in evolutionary divergence between parental lineages of fruit and dense spiny cucumber from their common ancestor. In this study, the female and male parent of Lvmei No.1 hybrid variety fall within the two clusters with the female parent grouped as fruit cucumber in clusterII and male parent belonging to the dense spiny cucumber in cluster I. The female parent X1 here was obtained after six generations of selfing of fruit cucumber hybrid variety HA-414 while the male parent X2 was a single plant selected after crossing between fruit cucumber 22–403 and dense spiny cucumber Zhongnong No.26. Therefore, the genetic background of X1 seems to be purely inherited from the fruit cucumber HA-414 while that of X2 is mixed despite the fact that they are recognized as fruit cucumber lines. Evolutionary divergence between lineages can be estimated using evolving characters, which are expressed via agronomical important genes. Morphological similarity between these two parents could be explained by the fact that the genomic regions carrying InDel markers used in phylogeny relationship construction may indirectly as well as not affecting cucumber fruit phenotype. Numerous genes were reported and certain have been cloned from different tissues of cucumber, including seedling, stem, leaf, flower and fruits as well as important disease resistance-related genes^59^. Information on most cloned genes in cucumber can be found in NCBI database. In this regards, we investigated and showed the location of those markers used in phylogenetic analysis. Most of them are located in genes potentially associated to oxidoreductases and hydrolases activities. To the best of our knowledge these newly developed polymorphic agarose-resolvable markers is the first of its type in cucumber and together with the 66 CPT InDel markers are of great importance in cucumber research. This will go a long way in advancing cucumber-breeding programs. They constitute a valuable genetic resource, which would benefit the cucumber industry and breeding community.

## Supplementary Information

Supplementary Information
