# Supplementary material for: Agarose-resolvable InDel markers based on whole genome re-sequencing in cucumber
Source: Sci Rep. 2021 Feb 16;11:3872. doi: 10.1038/s41598-021-83313-x (PMC7886880; doi:10.1038/s41598-021-83313-x)
Supplement: Supplementary file 1 — Supplementary Information [file 41598_2021_83313_MOESM1_ESM.docx]

**Agarose-resolvable InDel markers based on whole genome re-sequencing in cucumber**

AdedzeYawo Mawunyo Nevame^1^, Lu Xia^1^, Xia Yingchun^1^, Sun Qiuyue^1^, Chofong G. Nchongboh^2^, Md. Amirul Alam^3^, Liu Menghua^1^, Yang Xue^1^, Zhang Wenting^1^, Deng Zhijun^1^, Li Wenhu^1^, Si Longting^1^

^1^Molecular Biology Laboratory of Jiangsu Green Port Modern Agriculture Development Company, Suqian, 223800, Jiangsu Province, China

^2^Julius Kühn Institute (JKI) – Federal Research Centre for Cultivated Plants, Institute for Epidemiology and Pathogen Diagnostics, Messeweg 11-12, 38104 Braunschweig, Germany

^3^Faculty of Sustainable Agriculture, Horticulture and Landscaping Program, University Malaysia Sabah, Sandakan Campus, 90509 Sandakan, Sabah, Malaysia

Correspondence: e-mail: amen.nevame07@yahoo.fr

**All supplementary Figures and Tables**


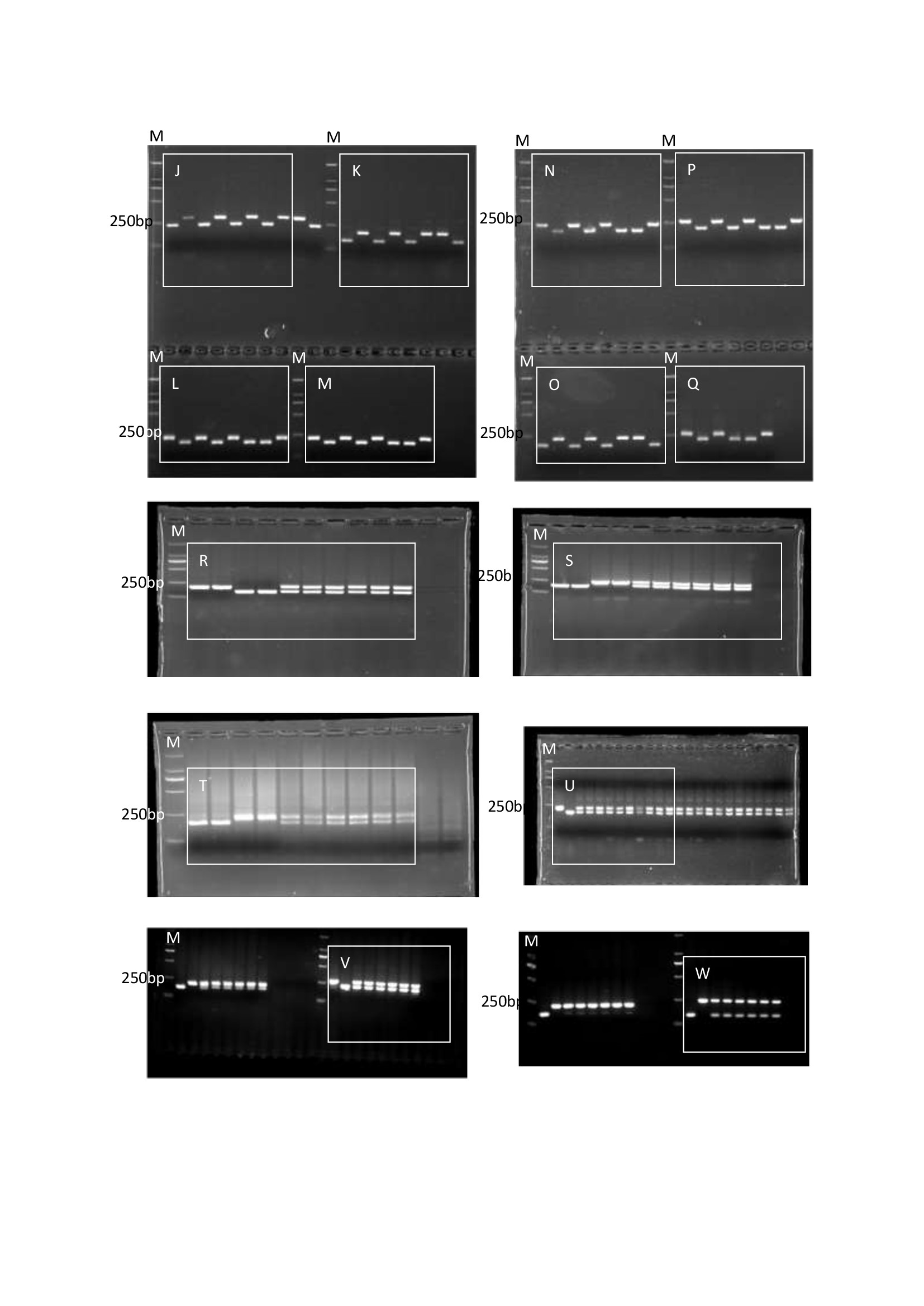


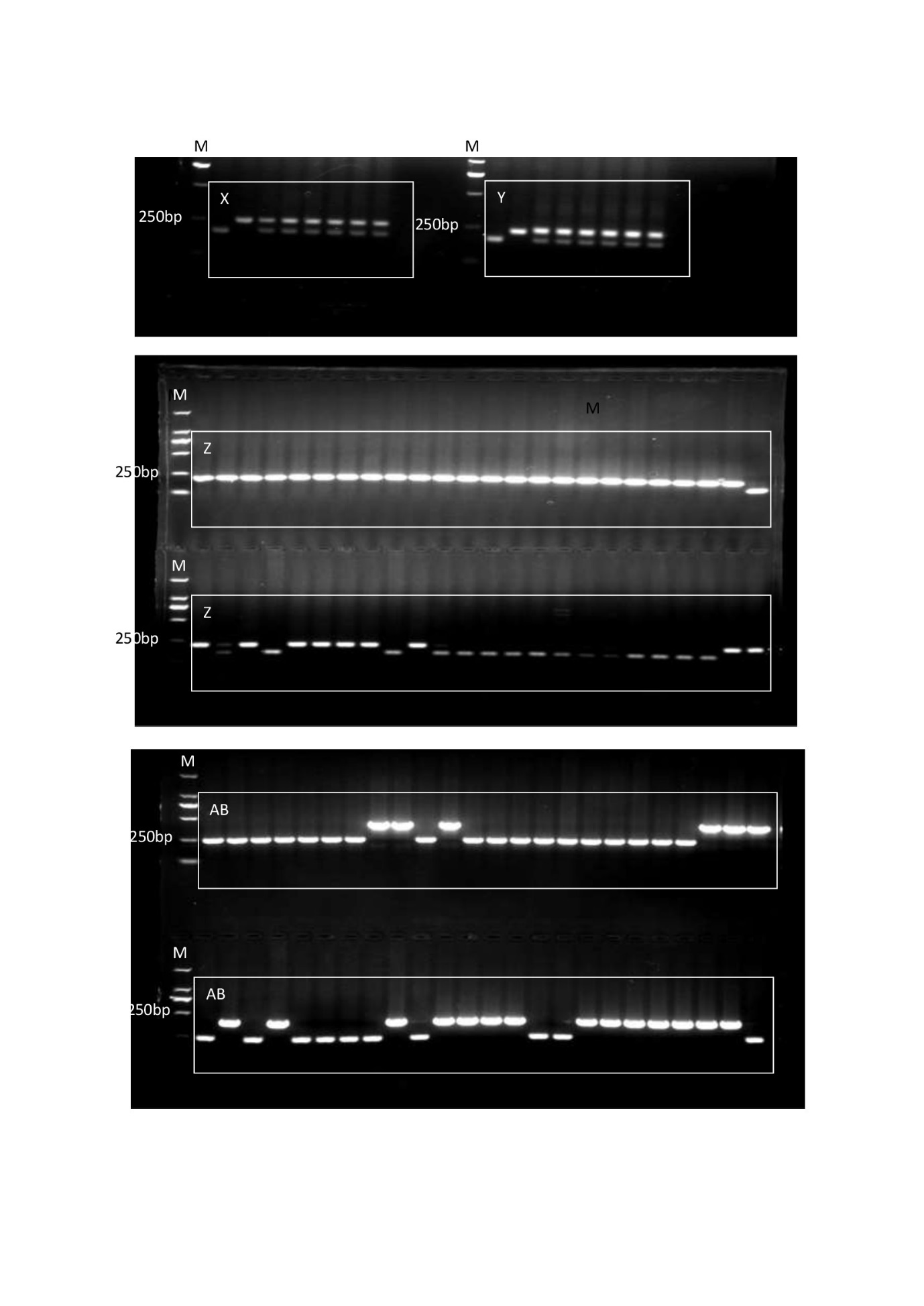


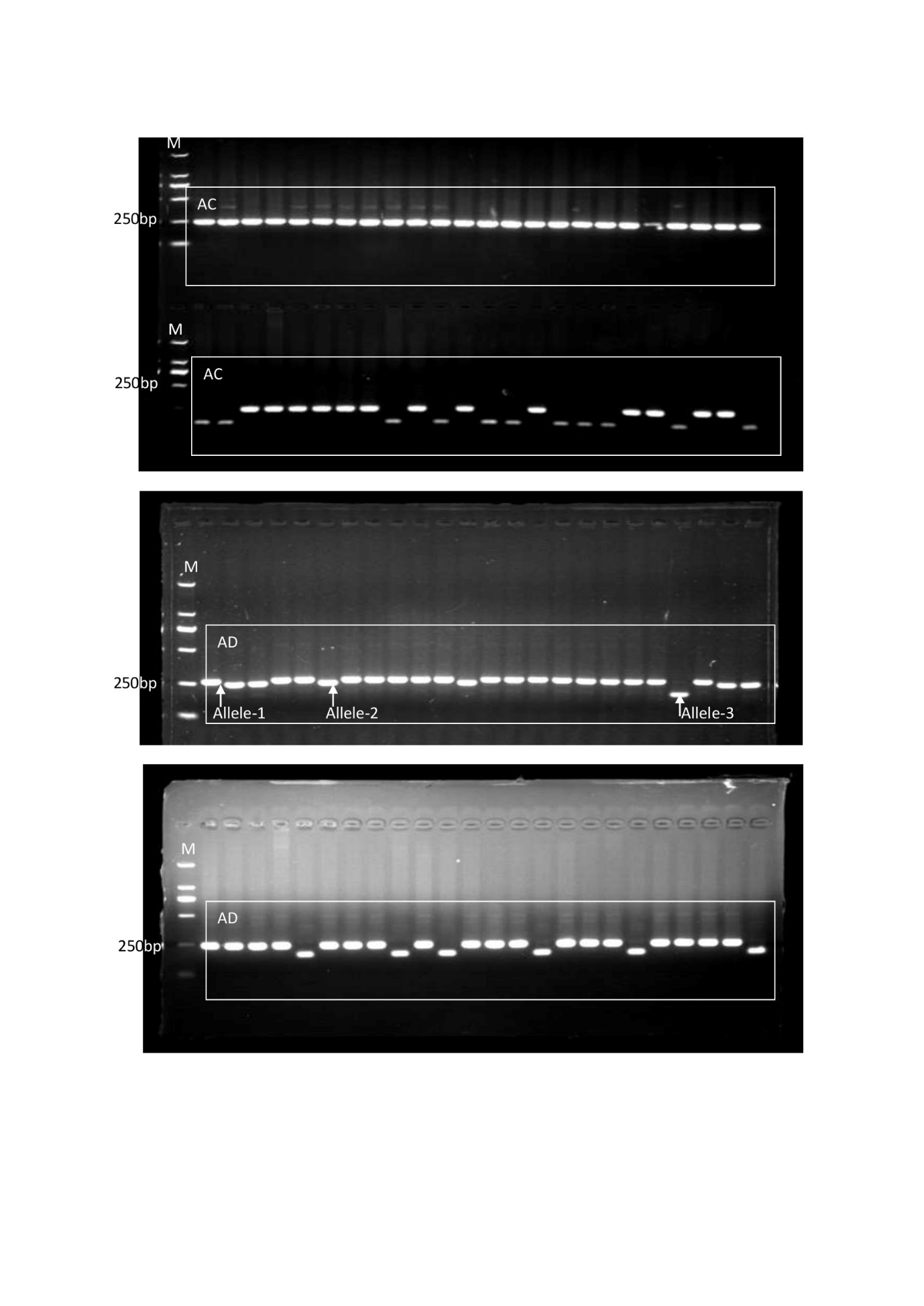


**Suppl. Figure S1.** The display of uncropped full-length agarose gel electrophoresis results using different types of Indel markers. Polymorphism analysis (J-Q), Hybrid seeds purity detection (R-Y) and validation of InDel markers in 48 cucumber breeding lines (Z-AD) corresponding to those respectively mentioned in Fig.1. The sections of gel framed with white lines represent the cropped gels results highlighted in Fig.1 of the main manuscript. In details, Polymorphism analysis was performed using InDel169 (J), InDel79 (K), InDel115 (L), InDel117 (M), InDel124 (N), InDel170 (O), and InDel114 (P-Q), respectively. Hybrid seeds purity testing of the elite cucumber hybrid variety LvmeiNo.1 was done using InDel79 (R), InDel170 (S), InDel114 (T-U), InDel124 (V), InDel232 (W), InDel269 (X), and InDel48 (Y), respectively. Experimental validation in 48 cucumber breeding lines was carried out using InDel161 (Z), InDel174 (AB), InDel232 (AC), and InDel269 (AD); Allele1, Allele2 and Allele3 are 3 different allele generated from the 48 lines using InDel269. Information concerning each genetic band is indicated in Figure 1 of the manuscript. M represents DL 2000bp DNA Marker.

**Suppl. Figure S2.** DNA sequence alignment and comparison between the sequenced PCR products from cucumber breeding lines of the LvmeiNo.1 and two other breeding lines of a commercial melon hybrid varieties using InDel114. X1 and X2 indicates the sequenced DNA fragments of female and male parent of hybrid variety Lvmei No. 1, respectively. M1 represents female and M2 male sequenced DNA fragments from female and male parent of melon hybrid variety. Pelo indicates genomic sequence of cucumber pelota gene. InDel-w specifically shows sequence difference between the two parents of LvmeiNo.1, InDel-x and InDel-z shows sequence difference between the DNA sequence of cucumber and melon parents and InDel-y indicates sequence difference between the two parents of melon hybrid variety. Asterisk indicates nucleotides similarity while the interrupted lines indicates sequences deletion. The underlined sequences are the pair of primers developed for InDel114.

B

A

B

C

A

**Suppl. Figure S3**. Displays of phylogenetic trees constructed using three different softwares: (A) NTSYS 2.1(Rohlf 2002), (B) Power Marker (PM) version 3.25 and (C) Darwin version6 (http:// Darwin.Cirad.fr). Black round and triangular indicate female X1 and male parent X2 of elite cucumber hybrid variety LvmeiNo. 1, respectively; the red colored branches (C) indicates the evolutionary pattern of the female and male parent.

A

| **Suppl. Table S1. The plant materials used in this study : A = 48 Cucumber breeding lines used in this study** | | | | |
| --- | --- | --- | --- | --- |
|  |  |  | **B = Additional hybrid varieties used in this study** | |
| **A: 48 Cucumber breeding lines used in this study** | | | |  |
| Number | Code | Group | Type | Breeding Company |
| 1 | D1 | Groupe1 | Dense Spiny cucumber | Jiangsu Green Port Co., Ltd |
| 2 | D2 |  | Dense Spiny cucumber | Jiangsu Green Port Co., Ltd |
| 3 | D3 |  | Dense Spiny cucumber | Jiangsu Green Port Co., Ltd |
| 4 | D4 |  | Dense Spiny cucumber | Jiangsu Green Port Co., Ltd |
| 5 | D5 |  | Dense Spiny cucumber | Jiangsu Green Port Co., Ltd |
| 6 | D6 |  | Dense Spiny cucumber | Jiangsu Green Port Co., Ltd |
| 7 | D7 |  | Dense Spiny cucumber | Jiangsu Green Port Co., Ltd |
| 8 | D8 |  | Dense Spiny cucumber | Jiangsu Green Port Co., Ltd |
| 9 | D9 |  | Dense Spiny cucumber | Jiangsu Green Port Co., Ltd |
| 10 | D10 |  | Dense Spiny cucumber | Jiangsu Green Port Co., Ltd |
| 11 | D11 |  | Dense Spiny cucumber | Jiangsu Green Port Co., Ltd |
| 12 | D12 |  | Dense Spiny cucumber | Jiangsu Green Port Co., Ltd |
| 13 | D13 |  | Dense Spiny cucumber | Jiangsu Green Port Co., Ltd |
| 14 | D14 |  | Dense Spiny cucumber | Jiangsu Green Port Co., Ltd |
| 15 | D15 |  | Dense Spiny cucumber | Jiangsu Green Port Co., Ltd |
| 16 | D16 |  | Dense Spiny cucumber | Jiangsu Green Port Co., Ltd |
| 17 | D17 |  | Dense Spiny cucumber | Jiangsu Green Port Co., Ltd |
| 18 | D18 |  | Dense Spiny cucumber | Jiangsu Green Port Co., Ltd |
| 19 | D19 |  | Dense Spiny cucumber | Jiangsu Green Port Co., Ltd |
| 20 | D20 |  | Dense Spiny cucumber | Jiangsu Green Port Co., Ltd |
| 21 | D21 |  | Dense Spiny cucumber | Jiangsu Green Port Co., Ltd |
| 22 | D22 |  | Dense Spiny cucumber | Jiangsu Green Port Co., Ltd |
| 23 | D23 |  | Dense Spiny cucumber | Jiangsu Green Port Co., Ltd |
| 24 | X1 | Groupe2 | fruit cucumber | Jiangsu Green Port Co., Ltd |
| 25 | X2 |  | fruit cucumber | Jiangsu Green Port Co., Ltd |
| 26 | X3 |  | fruit cucumber | Jiangsu Green Port Co., Ltd |
| 27 | X4 |  | fruit cucumber | Jiangsu Green Port Co., Ltd |
| 28 | X5 |  | fruit cucumber | Jiangsu Green Port Co., Ltd |
| 29 | X6 |  | fruit cucumber | Jiangsu Green Port Co., Ltd |
| 30 | X7 |  | fruit cucumber | Jiangsu Green Port Co., Ltd |
| 31 | X8 |  | fruit cucumber | Jiangsu Green Port Co., Ltd |
| 32 | X9 |  | fruit cucumber | Jiangsu Green Port Co., Ltd |
| 33 | X10 |  | fruit cucumber | Jiangsu Green Port Co., Ltd |
| 34 | X11 |  | fruit cucumber | Jiangsu Green Port Co., Ltd |
| 35 | X12 |  | fruit cucumber | Jiangsu Green Port Co., Ltd |
| 36 | X13 |  | fruit cucumber | Jiangsu Green Port Co., Ltd |
| 37 | X14 |  | fruit cucumber | Jiangsu Green Port Co., Ltd |
| 38 | X15 |  | fruit cucumber | Jiangsu Green Port Co., Ltd |
| 39 | X16 |  | fruit cucumber | Jiangsu Green Port Co., Ltd |
| 40 | X17 |  | fruit cucumber | Jiangsu Green Port Co., Ltd |
| 41 | X18 |  | fruit cucumber | Jiangsu Green Port Co., Ltd |
| 42 | B1 | Groupe3 | White green and black spiny Cucumber | Jiangsu Green Port Co., Ltd |
| 43 | B2 |  | White green cucumber | Jiangsu Green Port Co., Ltd |
| 44 | B3 |  | White green cucumber | Jiangsu Green Port Co., Ltd |
| 45 | B4 |  | White Green sparsely spiny Cucumber | Jiangsu Green Port Co., Ltd |
| 46 | B5 |  | White Green sparsely spiny Cucumber | Jiangsu Green Port Co., Ltd |
| 47 | B6 |  | Yellow Green Sparsely spiny Cucumber | Jiangsu Green Port Co., Ltd |
| 48 | B7 |  | Yellow Green Sparsely spiny Cucumber | Jiangsu Green Port Co., Ltd |
|  |  |  |  |  |
| **B = Additional hybrid varieties used in this study** | | | |  |
| Number | Code | Parents | Crop varieties | Breeding Company |
| 1 | C96/C1396 | C1&C2 | Cucumber varieties | Jiangsu Green Port Co., Ltd |
|  |  |  |  | Jiangsu Green Port Co., Ltd |
| 2 | C87/C360 | C3&C4 |  | Jiangsu Green Port Co., Ltd |
|  |  |  |  | Jiangsu Green Port Co., Ltd |
| 3 | C38/D18 | C5&C6 |  | Jiangsu Green Port Co., Ltd |
|  |  |  |  | Jiangsu Green Port Co., Ltd |
| 4 | D52/C335 | C7&C8 |  | Jiangsu Green Port Co., Ltd |
|  |  |  |  | Jiangsu Green Port Co., Ltd |
| 5 | M13065-1/39-1-1-1-1 | M1&M2 | Melon varieties | Jiangsu Green Port Co., Ltd |
|  |  |  |  | Jiangsu Green Port Co., Ltd |
| 6 | M13065-1/17065-2-1 | M3&M4 |  | Jiangsu Green Port Co., Ltd |
|  |  |  |  | Jiangsu Green Port Co., Ltd |
| 7 | M170892/7305 | M5&M6 |  | Jiangsu Green Port Co., Ltd |
|  |  |  |  | Jiangsu Green Port Co., Ltd |
| 8 | PK2-15/Q204 | W1&W2 | Watermelon varieties | Jiangsu Green Port Co., Ltd |
|  |  |  |  | Jiangsu Green Port Co., Ltd |
| 9 | PK2-18/74-1-3 | W3&W4 |  | Jiangsu Green Port Co., Ltd |
|  |  |  |  | Jiangsu Green Port Co., Ltd |

Suppl. Table S2. A total of 10470 InDels markers identified and successfully mapped on the 07 Cucumber chromosomes sequence for X1 and X2 parents

Chromosome Position Forward Primer (5'-3') Size (bp) Annealing Temperature (℃) Reverse Primer (5'-3') Size (bp) Annealing Temperature (℃) PCR product size (bp)

chr1 49119 ATTAACTGTGCATTGACTTGGAGC 24 60.083 TCCCCTCTTCTACTTTACTCCCAT 24 60.016 270

chr1 50116 GTTGTTTCTAGTTGCGTACCCTAC 24 59.613 AGTGTAGTACCTGATTCCGTAAGC 24 59.902 288

chr1 52277 CAATTGTCCCGTTGAACTTAGTCC 24 60.083 GGTTGCAACACCCTAAATTAAAAGC 25 59.821 260

chr1 52548 GTGAGCTTTTAATTTAGGGTGTTGC 25 59.366 AGCATCAGACTACGTATTGGTTCA 24 59.84 210

chr1 58338 ACAATTAAGCCTTGAGTTTGAGCC 24 60.022 TCCAGGTTTGCTCTTGAATATTGC 24 59.842 268

chr1 58765 AAACTCATGACCTCTTCGTTAGCT 24 60.021 CCACAGGAACAAAAGTTTACTCGA 24 59.427 238

chr1 71212 TGGTGGTCTATTGGTTGATGCTTA 24 60.019 ACCCATAAACCCTTAAACCCTCTC 24 60.018 219

chr1 73155 TTCCTTCCATCCGATTACCCTTTT 24 60.018 TAGGGAATTGGTAAGTGTCATGCA 24 60.019 141

chr1 73813 GGCTCTATTTTCACCTCAGATTGC 24 59.964 ACTAGTTGATGCTTCCACCTTCAT 24 60.019 162

chr1 78057 AATAGGCAAAAGAAGAGAACACGC 24 60.083 TTATTCTCGTCTACCCAAGCGTAA 24 59.601 299

chr1 79353 GCTAAGTACCACTATGCGACACA 23 60.428 TTGTGTTTCAAGTTTCCCCTTCTC 24 59.599 106

chr1 86143 TATTCGGCTCAGTTCAAGTCTTCT 24 59.779 TCATAACGGTGTCCCTCTTTCTTT 24 59.959 279

chr1 87421 ACCTTGATACGGCTTCTTCTTCTT 24 60.02 TCAATGTGCAAATCTATAGCCGAC 24 59.489 284

chr1 92921 ATGACCAAAACCAACCTCCTACAT 24 60.203 GTTTGAATGAGCTCGAAGGAAACA 24 60.024 153

chr1 93559 CGTCATACAAGAGGAGAAGACACA 24 60.083 CACGGTAGCAAATTGGGAGTTTT 23 59.995 290

chr1 107837 GTTGAGAGAATGAAGAAAAGGGGC 24 60.083 GCTAATAATCAACAACCACCCCAG 24 59.902 271

chr1 168264 ATTGTTATGTTCCTCTGCCTCCTT 24 60.018 GCCTGCTTCTATCATTCCAGAGAT 24 60.263 237

chr1 184128 CCTTGGGTGGATTTCATTTCATGT 24 59.777 TAAAGCCTTCCTACCCAAAATCCA 24 59.955 289

chr1 187198 TAATCGCATTTGACCACTCTACGA 24 60.142 ATACGTTGGATGCATTGGATTTCG 24 60.261 267

chr1 191315 ATCTACGCCCCAACTAAGTTAAGG 24 60.142 CACCCAAGAACTTTGAGACGTTTT 24 60.142 300

chr1 191677 AAAACGTCTCAAAGTTCTTGGGTG 24 60.142 TGACTGCATTGAGAGTGAGAAAGA 24 59.96 288

chr1 198688 TCCCTACACTTTTGATTTCATGTGC 25 60.048 GCGTTCTTTCTGATTATCTGCTTCA 25 59.936 255

chr1 200180 CCATCTCCTTAATATTTGCGTCCA 24 58.937 TTTTCTTCTTGGAATCATGCTCGG 24 59.843 194

chr1 205192 AATTTCTGCATAGGAACATTGGGG 24 59.593 GAAGAAGAGGGAAGAAGAGGGAAG 24 60.081 135

chr1 211577 CTGTAATTCCTTTCCCTACCCCAA 24 60.018 GTTTGGAATTAGCTATGTTGGCCA 24 59.84 154

chr1 216340 CACCTCCCTTCCTTTCATAGTCAA 24 60.019 CCAATAAGAGAAGACAAAATGGGG 24 57.787 299

chr1 223427 ACGCATTTTAGTGTACCAGAGCTA 24 60.082 GACGAACAAGTAAGCCGATCAAAA 24 60.084 274

chr1 223592 TTTTGATCGGCTTACTTGTTCGTC 24 60.084 GTGGAGTATTTGTGGCATCTAAGC 24 59.964 148

chr1 224088 TCTTTCCGTCACAAATACTCGAGA 24 59.783 TGCAAAATTACCATACCGTCCTCT 24 60.324 214

chr1 230298 ATATTATGATGTCGCACCCTGAGC 24 60.861 AAATCTGTTGCCTCACCATCAAAG 24 60.022 275

chr1 250481 ATACATCAATGGCGGGAATTCAAG 24 59.721 CGGCCATATCTATCTCAAGGTAGG 24 59.901 260

chr1 251255 CGAGTAACTTGGCTCTCTTGTCTT 24 60.56 TCAGAAGTTAATTGCACTCATCACC 25 59.587 80

chr1 251375 CGAGTAACTTGGCTCTCTTGTCTT 24 60.56 ATAACTAATCCCTACCGTCAACTC 24 57.598 233

chr1 259783 TTGTATACAGGCCCCTTGAAAA 22 57.61 GACTATCGAACGTCTATTACATGGA 25 58.104 120

chr1 260559 TGGTCTCCACATCTTCAACTTT 22 57.562 TGGTCCTAGTTAGTTGATGTGTCA 24 59.164 269

chr1 263288 CAAAATCTGACGAGGGTGTTCATT 24 59.784 TATGCTCAACAAAGTTGCTGGATG 24 60.083 218

chr1 267327 AGTGCTTTGATGTGTTTGAGCTTC 24 60.499 GTTGGATAGTCGTCTTCATCTCCA 24 59.902 256

chr1 273080 AAAAGAAACGTAAATGTAGGGCCG 24 60.083 ATAAATTGAATGGCTGCTGTTGGG 24 60.383 240

chr1 274571 TTTAGCACATTTGTCATGTCCTCG 24 59.846 TGCTGCCCTAATACTCATCAAAGT 24 60.08 294

chr1 282569 TTCGGTGTTGGGTAGATTGAGATT 24 60.02 TACTGCACCACTCTCACAACTTAA 24 59.9 260

chr1 287331 TCACGAAACCCTAACTCTCTCATT 24 59.473 TATTTTGAAAATGATGCCCCTCCG 24 59.901 300

chr1 295593 GGAAAATGAAGGGCGCATATTACA 24 59.963 CAGTAGCAGCACAAACAAACAAAC 24 59.968 284

chr1 1000957 CACACACTCTAAAACCTAAAGCCG 24 60.084 AGCCTTTGGAGAATGTGAACTTTG 24 59.962 276

chr1 1061101 GTGGGTTTTGCTAGCTAAGACTTG 24 60.083 ATACAATAGGCTAGCGGGATGTTT 24 59.958 280

chr1 1405647 AATAATATGGAGTCAACGGCCTTG 24 59.178 TACAGTACAGGGGAGCTTCTAAGA 24 60.018 243

chr1 1411659 GATGTCCACCACTTCAATTTCGG 23 60.12 ACTTGGTGCTTGGTTATTCAAAGC 24 60.501 270

chr1 1599051 GAATGTGGCATTTTGAGAGGCTAA 24 59.842 AATAAGGGGAAAGCAGCTAGAACA 24 60.018 190

chr1 1623452 CATTGAAGATGGCGCTTGATGTAT 24 60.023 AACATGAAGCACTTATTCCACCAC 24 59.782 248

chr1 1624053 TATGATGAAATGAAGTGGGTGGGG 24 60.631 ACCCTTGATTCTCTGGTTTCTTCA 24 59.895 283

chr1 1882121 TATGTCTCTCAGCCAACAACATTG 24 59.303 GGGATAGTGACAGTGGAGAGAATT 24 59.593 210

chr1 2000263 CCCCTCCTCTTCCATCCTTTTAAA 24 60.017 AAACAGATTTCACCCGAGGATGTA 24 60.02 198

chr1 2746320 CGAACACAACAACCATTACTTCCA 24 59.964 ATCTCTCCCTCAAAATTCCCAAGC 24 60.874 286

chr1 2747746 CTTGCCAGGCTACCAAATTTCTAG 24 59.903 GCAGCATATTTAGAACTTGGACGG 24 60.261 292

chr1 2797299 CTCAGTGCCTCAGAAGAAAAGTTG 24 60.024 AGAGGAGTGGATAGTTTAAAGGCA 24 59.217 121

chr1 2802678 TTTTGCTCCTCCCTCACAATAGAA 24 59.957 GTGAAATCTAGCCAACGACCAAAT 24 59.844 149

chr1 2811827 CTATTGAATTTTGGTTCGTGCCCA 24 60.322 CGTGGTTGTAATTCATGCTATACAC 25 58.581 244

chr1 2866689 AGAATCTTCCAAGGGTGTGTGAAT 24 60.203 ACATGAAGGTTTTGTCTGGGAAAG 24 59.658 255

chr1 5384536 GCAAATGATACATCCTTACACAACC 25 58.734 ACCTTTGTAGATTGTAGAGGAAGAGAG 27 59.876 140

chr1 5385552 CGAAGCAATTAGATCTGAGCGAAC 24 60.318 TGGTCTACAAAATCTGCTAGCTGA 24 59.777 152

chr1 5388416 AATCTCCACCCTCAAGCAAAGTAT 24 60.018 GAATAGTGGAAGAGATGCGAGGAT 24 60.022 172

chr1 5390536 TTAAGGTTCCTATGTCCTACCACC 24 59.282 GGTAACGAGCTCATAACTGCAAAA 24 59.847 151

chr1 5517123 GTCCCTCTTTCCACCCTTAATCAT 24 60.08 GCGCATTGACAACCTGTGATATTA 24 59.965 166

chr1 5521224 AAAAGGTGATGAGTTGGCTTAGTG 24 59.48 ATTTGGGGATCTTTTAAGGCAGGA 24 60.265 227

chr1 5537464 GTGCTACACTAAATTTCTCTTCTCC 25 57.703 TGAGTGTTCATGGAGCTCGAATAA 24 60.082 238

chr1 5538386 AATCCATGACTCTCTTTGCTTTGC 24 60.082 ACCACAAGTCCACAACAAAAGAAG 24 60.082 296

chr1 5540772 TGGTAAAAGTGTTTGGTGAGTCAC 24 59.603 ACCACAACCCAATTCACAAAGATG 24 60.202 285

chr1 5540964 ATCTTTGTGAATTGGGTTGTGGTG 24 60.202 GATCAACCACCCACCAAGAATTTC 24 60.322 221

chr1 5541110 GAAATTCTTGGTGGGTGGTTGATC 24 60.322 TTATGGACAAGGAAAAGGCACATG 24 59.78 282

chr1 5542049 TGTTTCTTGGGATTCTCTCATGGA 24 59.711 GTTCAAAACCCATGTCACACTGAT 24 59.962 249

chr1 5558025 GTGCATGCAACAAGTTTCTCAC 22 59.209 CGTATAGTTTAGTCTTCTTCGCATCAC 27 60.097 292

chr1 5566766 GGTCTAGATACCGTATTTTGTCCT 24 57.598 AGTGGGAGAGTGATGACAAAGAAA 24 59.897 250

chr1 5818738 AGCCACTCCACCACAACATC 20 60.251 GAAGGTCTTGCGGTACAGATCT 22 59.833 245

chr1 5839594 TTGGGACGATGATTATCTACGACT 24 59.174 GTCATTACCATTCTTTGCGACA 22 57.321 249

chr1 5845890 TTGTCTTGGGTTTTGGAGTTTTGG 24 60.323 CCTCGTATTAACAGAAGTATCAAGTGC 27 60.043 243

chr1 5875763 GTTTGGGCCCTTCTAAAGATAAACA 25 59.521 GGAAGGTTGGTATGAGTTTAAGAAG 25 57.621 145

chr1 6107617 AAATATTGCACACGTGACGGTTAC 24 60.378 AAAGTAAAAGCGTAGAGGAGGAGG 24 60.082 234

chr1 6111973 TGCATACCGGACCCTGTATTAAAA 24 60.08 AAATCCTCCAAACCCATACTTCCT 24 59.705 132

chr1 6117627 CAAAACACAAGCTACGGGAAGAAT 24 60.023 AGCATAACTCAATTGACCGCATATG 25 60.049 197

chr1 6247098 CATCATTTTGGACATGGGGTTTCA 24 60.02 CAAGTTCAAAATTGCTGACGAGTAC 25 59.379 205

chr1 6264127 GGATACGAGATAGACAATGACATCA 25 58.086 ACGTTCAAGTTCATCCACATGT 22 58.523 216

chr1 6264564 TGAGTATGTGTCATGTGTGCA 21 57.601 ATACGATATGGACTGAGAGATGCG 24 59.904 247

chr1 6268580 TCTTGCTTTAGGTCTCTTAGGGAA 24 58.908 ACTTTCACTGCAGTTCGAGAAA 22 58.468 199

chr1 6270371 CACTGGGATTGACAATAACAACACA 25 59.99 TTATAGAGTGTGGCGTGAACTACC 24 60.142 232

chr1 6276070 CACTTTCTGATGCATGTTTACCGT 24 60.083 TCTCCCTTTTCCCACACATATCTC 24 59.836 228

chr1 6278012 CAAAACGCAAATGTCGCAAAAC 22 58.981 CCCGATTTTATGTCCCTATTGTTGA 25 59.174 299

chr1 6306838 ACTGATTTGGTCCTCTCTACAAGT 24 59.159 GTCGAGTGAAGAGTAAAATGCACA 24 59.552 194

chr1 6336017 AGAAGACCAAGTAGAAGCAAGAAGA 25 59.694 CCGCGCATACACACACATATTTAA 24 60.26 218

chr1 6352659 CCCATGTCCTTTCACAGAGTAGAA 24 60.02 GGATCTTTCACTACGAGCAACATG 24 59.967 250

chr1 6355448 ACGAAATAGATTTGGCTGAGAGAGA 25 59.872 GGAGAGAAAACACATCCAACCAAA 24 59.659 234

chr1 6369615 CACTTTCTCCAAATGCTCAATTGC 24 59.612 GGCACACTTTGTCGAATTTCAA 22 58.299 255

chr1 6415539 TGCCTACGTATCAGAAAGAACAAGA 25 60.048 TAACGTGAGGAAGGAAGAGTTGTC 24 60.262 235

chr1 6431666 GAAGAGATCGAGACCAGAAATCCA 24 59.901 AGTTTCTTCCACGTCTCTCTTTGT 24 60.142 146

chr1 6456049 TCTTGGGAGAAGCTTTTGGAGAAT 24 60.203 AAAAGATCCCAATTTTCCCAGCTC 24 59.776 299

chr1 6459140 TCCCACGTTGCACTAATTACAAAC 24 60.024 TTGCAATCCTAGTTTCAAGCATCC 24 59.842 135

chr1 6459278 TCCCACGTTGCACTAATTACAAAC 24 60.024 TCGAAGGTACAGTAACAGTGATCA 24 59.237 230

chr1 6464317 ACCATTACTGTTGAGCTTCCAGAT 24 60.019 ATGGTCCTCTTTGCACATCTTTTC 24 59.781 199

chr1 6466582 TCATCTGCAATAATTGGTAGCTGC 24 59.723 CGTGCAATAAACTTGAGGGTCTTT 24 60.023 267

chr1 6467923 GTCGCTGTCAACATAAATGATCACT 25 59.936 GGTTTTAAACTGTCATGAGCACTC 24 58.547 253

chr1 6468984 TAACGTCCATGCAACTAGAACCTT 24 60.263 AGGAGAGAGATGTTGTTTAGAGCG 24 60.142 267

chr1 6470793 ACCCGTATTCTAAATACACCCACG 24 60.442 GAAATCTCAGTTTGTCATCTTCCAG 25 58.16 228

chr1 6472946 GAAGTCAAATGCCCAAACCTTCAT 24 60.263 AGTCAAAAGGAGTGTGAAGTTGGA 24 60.324 229

chr1 6473457 GGTTGAGCTATTTTCTGTCTCGGA 24 60.621 TCAGTACAATGAGAGAAGACTTGGA 25 59.225 159

chr1 6485788 GTCAGGGTTTCTTGTGCCTTTG 22 60.224 CAGCATATCAACAAAGTCGAGACC 24 59.967 290

chr1 6491029 ACACTACAAGAAATCGGAGCTCTT 24 60.021 GAATAGTTTCACTTCTTGCGACGT 24 59.85 256

chr1 6514129 ATTATCCACAGTCCACAGATGCTT 24 60.08 AGTACTTTCATCATCCCACTCCAT 24 59.276 146

chr1 6515230 AAGTGTGAGCCCATTACCATATCA 24 59.834 TATCTGATGCCTCTCTCTAGCTCC 24 60.568 229

chr1 6518890 TGGGAAGTGTGTTATCAATAAGTCG 25 59.127 GGATACATCAATGAAATACGCGCA 24 60.084 287

chr1 6534232 GGTTGGGTTGTCGGATTGTAAATA 24 59.299 GGGTTGGGTTGGGTTAAACTTTT 23 59.8 298

chr1 6534386 AGTCTGGGTTATTCGAGTTCAACT 24 59.716 TAAGCCCGTCTCTTTGTAACTACG 24 60.38 298

chr1 6541442 CATTTTCCCATGTGCAAAGTTTCG 24 60.084 ACCACCACACTTTATAACGTACTCA 25 59.989 157

chr1 6563607 AGAGTAACTATGGTGCATCATGTCA 25 59.871 CTTTCACCATTTTCTTCCACCCTC 24 60.022 297

chr1 6571079 CATTGTCCAAACCTGTGCCAAATA 24 60.262 CGATTTCAAACCACGCAGATTCTA 24 59.907 202

chr1 6614997 CAAATCCACTTCCATGTCCATACG 24 59.964 GTCTGGAGAAGGATATGGTCATGG 24 60.263 266

chr1 6630592 CTTGACGGTTGTATTCTTGCTGTT 24 60.024 GCAATGCAACGACATCTGTACAT 23 60.182 291

chr1 6661947 GTAATGTGGGAAAGGAGAAGACCA 24 60.263 CCACTTTCCTCTGTCCTTTCACAT 24 60.748 264

chr1 6662517 ATCAACTCCAATTGCACCCTTTTG 24 60.504 CTTCTTCAACAAACCTTCCCCTTT 24 59.594 282

chr1 6676672 AAATAGTCTGGACCTCAAACTCCC 24 60.019 ATAGCGCAATTCTAGACGGAGATT 24 60.022 235

chr1 6680477 CAGCCGCTCAACATACTTTTGA 22 59.516 ACATACCTACAGATTGCAGCAGTA 24 59.594 300

chr1 6706623 TATGGTTAACACACGACAGACAGT 24 59.962 TGGATTAATTGAGGAGGGTGTTCT 24 59.46 288

chr1 6711419 ATAAAGGGTGTCAAAGAAAAGGCC 24 59.717 CATATCAGCACACGTCATCCAAAA 24 59.905 202

chr1 6768433 ACTATCCTTGCCGTAGAACAAACT 24 60.02 TCCTCCTTCCTTTCCCTTCTAGAT 24 60.015 257

chr1 6792598 AAGGTAAATGGGTCCAATGAGA 22 57.054 GGCCTCACTTGCAACAAATATCTT 24 60.082 262

chr1 6800850 CCAACCCCTCAATCTCCCATATAT 24 59.458 GATTAGGTTCGCACAACTCTAAGC 24 59.967 106

chr1 6807175 CAGCTAGCACCAAAGTATAGGAGT 24 59.9 GTGCCTGAATCTCTAATCACTTGC 24 59.965 229

chr1 6815051 TCTCTACAACTTCAAATACTCCCCT 25 58.976 AGGCCTTATAGTGGTAATGGGTTC 24 59.896 298

chr1 6819146 CCGTTCTTGTTGAGCCTATACATG 24 59.729 ACCTATTGTTTTGGCTGTGTGA 22 58.439 96

chr1 6835807 GCCCTCTAATAACTCTTTGATAGTCG 26 59.136 GCGATACAACTCCACATCTCAAAC 24 60.202 251

chr1 6855569 ATGTCTCAATTCTCTTCAACGGGT 24 60.263 ATGAAGAGAGAGAGATGAAGACGC 24 59.963 282

chr1 6856611 GCACACTCTAAAACACCAGGTAAG 24 59.787 CGGGAAAGATCTAAAATGGCCAAA 24 59.84 229

chr1 6882231 TAGTAAGGCTATTGGGGTTTTGCT 24 60.018 AACACTTTCTGCCCTCCATTATCT 24 60.018 213

chr1 6889103 GGTATTGGTGAAGACATCTCGACT 24 60.142 AGTTCCATCATTCCATAGCTTCCA 24 59.833 146

chr1 6942813 CGTTTCCTAGTCCTGTCCTTAACT 24 59.78 GTTCCATTCTTAACAAGTGCCAATC 25 59.136 278

chr1 6943094 TAGAGAAAAGGTGAGTGCAGAGAC 24 60.022 TGTGAGTAAGAGAAGTGGAGATGG 24 59.536 95

chr1 6956986 CGAGGCAATTTTCTTTCCCTTCTT 24 60.022 TGGGTATGAATTGGACTTTGAGCT 24 60.264 238

chr1 6959531 CTCTACTTTTCACATCTATCCAAGGAC 27 59.291 TCCTTACGCCAATTCTTCTTACCA 24 60.02 257

chr1 6961554 TGTGGGGTTATTCTTCTTCTTCTTC 25 58.761 TCATTGAATACTACTCGGGGAAAGA 25 59.34 264

chr1 6964993 ATGCATGTTGAAAGGTGATCTCTG 24 59.602 GAGTGATTTGAAAGTGGTGAGGTG 24 60.024 203

chr1 6965442 AAGTAAGGGATGCCAACCAAAATG 24 60.02 TCTTGCTGAGCCATTTAACATGTG 24 60.083 227

chr1 6965729 TTCTGCATAATCAAACACGAGGAG 24 59.369 ATCCAGTACATTTCCTGATTGCCA 24 60.325 300

chr1 6966633 CTGATACCATTAAGTCACCCATTGA 25 58.643 TCTTCTTTGAAGTCGTCTGTCACT 24 59.902 259

chr1 6968049 AGCCATTCTCATTATTTTGCAGCG 24 60.736 AGAAGCCCTAGTCAATTTGATGGA 24 59.772 171

chr1 6978596 ACCGAGTTCTTCAGTGATTGGTAA 24 59.96 CACTGTTTTCATTTCAAGGCTTGC 24 60.025 214

chr1 7002911 ATTGATTTGAGGTCTAGGGGATGG 24 59.895 TCCGTGATAATCGAAGGAAGAACA 24 59.841 293

chr1 7013468 GAAGTTGGTTGGTGGAGTTTGATT 24 59.901 TTGTCCCCATCACGTTAAAGTAGT 24 59.959 262

chr1 7020228 CTGTTTTCCTTTGCTACCCTCTTG 24 60.023 CGTCAAAGATAGTAGTACGAGGCA 24 59.965 217

chr1 7020980 GGCAGCAGGAAATGGATTATTGAA 24 59.9 ATGACTTAGGATCTGCGAAGGTTT 24 60.081 285

chr1 7025750 ACACTTTATTGATTAGGGACCACGA 25 60.046 TGGTTCTAACAAACAAGAGGCCAT 24 60.691 290

chr1 7027138 GGAAGCATGGATAAAAGAAAGGGT 24 59.288 GATTGAGACTTCGCAGGAGAATTG 24 59.966 299

chr1 7029776 AAGGACTAAAATTGTGGAGTTGCG 24 60.023 TTTCTATGCACCCTAATCTCACCC 24 60.141 198

chr1 7031129 GGCGGATTTATCGATGTGGAATTT 24 60.023 ACCCATCTCCACTTTCTTCTCTTC 24 60.02 287

chr1 7041702 TGTTTTGTGAACGCTTGGATTACG 24 60.787 TGGAGAAGGATTATTTGGGGAAGG 24 60.079 268

chr1 7044522 GTGGTTCAAATTCCTTCCTTTTCAG 25 59.014 TTTGTGCCCACCAATACTTCTTTC 24 59.961 279

chr1 7051445 AGTGTCATTCTCAGAGGAAGAACT 24 59.162 CCCCTCAGAAGTTTGGTTTTGATT 24 59.655 289

chr1 7056056 TGACAAGTTTATCTATCCACGCCA 24 60.081 AAAAGTAGGGTGTCATGGTGATCA 24 59.957 225

chr1 7057119 GGCCACTCCTCTCAATTTCTAATT 24 58.803 AGTACCAAGTAGAGTTGCTTTACCA 25 59.694 289

chr1 7058759 CCAAAACAAAGGACGATTTCAGGA 24 59.963 GATTACTAGGATTGCAACTTGGGC 24 59.963 256

chr1 7063154 GAAACTTCGAGACGTTCCTGGAT 23 60.614 TCCAAAGACCCAAGGAAAAGGAA 23 60.052 218

chr1 7071718 AGGTAATTTTGTGAGGTTGAGGGT 24 60.141 CTTCTTCTTGTATGGAATGCGACC 24 59.965 272

chr1 7101465 TCTCAAAAGATGCACATGTAGC 22 57.048 AGCAATAGAAGTGGTCGAGTAGT 23 58.982 174

chr1 7102915 ATGTATTATGGCCCTACTAGTGAG 24 57.141 ACCACAAGACTATCAAAATATCCCC 25 58.634 293

chr1 7110242 CGCAGCTCGTATAACACTTGAATT 24 59.967 GGTGACAACGTAGATTTACAACTCA 25 59.307 209

chr1 7110384 GAGTTGTAAATCTACGTTGTCACCA 25 59.307 TATTTTGGAAGGGCTGTCAAACAC 24 59.961 297

chr1 7111584 AACCCCAACTAACCCCTGAATATC 24 60.079 ACCTTCTGATTCAGCACTCTACAG 24 60.082 257

chr1 7130847 TGTGGATGAAAATGCTCACTTTGG 24 60.262 CGCACCATATCATTGTCATTAGCA 24 59.786 184

chr1 7137058 AAACACACTAGATACCCTCCGTTC 24 60.082 GTGCTATTCATAACAATTCCCCTTC 25 58.261 215

chr1 7140699 GGTACCCATCCATTTCTACTTATAAGC 27 59.393 GAAGAACATCCGATCCCATTTTCC 24 59.963 102

chr1 7173660 AGAAGAAACTCGAACTACACACCA 24 59.901 ACTGCTGGATGGTCGTTAGTAATT 24 60.081 184

chr1 7187763 TTAGACAGCGAAATGAAGCCAAAG 24 60.083 GGAGACATATAACCACTGCACAAT 24 58.874 229

chr1 7242161 AATTGGGGCAAACAAGAGTCTTTG 24 60.443 CAGTTACCGTGTTTGTGAGTCTTC 24 60.026 212

chr1 7265558 CCTCATATTTCTTTCCCTAATCTTCGC 27 60.095 ACCCAACTCTTTACACCCACAAAT 24 60.633 300

chr1 7268248 CTAAAGAAGGGCCGATAATGTGG 23 59.185 TATGAGGAATGTACACTGCATGCT 24 60.142 193

chr1 7269410 GAGAGTCCAAATTTCATGTTTCGTG 25 59.147 GCAAGGCCCATTAAGAAAATGACT 24 60.081 270

chr1 7273575 ATTGGGCCACACTAAAAGTTGATG 24 60.021 TTTTGGACGCTACCTTTGAGTTTC 24 59.964 294

chr1 7273928 TCTCCCTTTCTTTTCTTTCCTCCA 24 59.584 TGTGGATATATGGATGGGGTCAAG 24 59.711 230

chr1 7276848 TCTAAGTTAGAGGACGAGTAGGGC 24 60.686 GGTGTTGTTTTGTTCAGGTGCTAT 24 60.202 234

chr1 7277937 GGACAAGCATCGTTTACATTGACT 24 59.846 TTTCCATTCTATTCCACTCCCCTC 24 59.834 295

chr1 7282324 TGACAAAGGAATCAAAAGGGGTTC 24 59.656 TCAACCTTCTACAACGCTATTCCT 24 59.778 163

chr1 7287133 CACTGCATCATCTTCATCCATCAC 24 60.024 ATCATCTCCAACCACCCACATAAA 24 60.018 261

chr1 7289412 AGACCGTGAGATATAGAGGGAGTT 24 59.895 TGGTTGTTTGACGTGAGAAAGT 22 58.66 295

chr1 7292009 ATTATGCACTAATCTCAGCTGCCT 24 60.202 CCATACTTTAGGGGTGGTGTAAAA 24 58.428 271

chr1 7295137 TACACATATGGACCAATCACACCG 24 60.682 TTTTACGCCAAAGATTTCAGGG 22 57.487 104

chr1 7295780 TGCGAAACAAGTCTACAGTTCAAC 24 59.966 AGAGTCGTGAGTGCTAGAAACAAT 24 60.022 187

chr1 7314415 GTCAAAACATTCTTATAGCCAAGGG 25 58.436 TGCCTTAATCGTCTGAGTTGTACT 24 59.78 200

chr1 7317559 ATTCCACTTCCACGATCAACAAGT 24 60.747 AATTTCCCCTTTGTTTGCAGAGAG 24 59.96 300

chr1 7317800 AGAACGTCATTTACTATGCCATCTG 25 59.245 TAATTGGGGAGGAAACAACAATGC 24 60.021 292

chr1 7320335 TCATACACCAAACAATGTCCACAC 24 59.723 ACGAAGAGTTGTGGTGGTAAGAAT 24 60.202 213

chr1 7320646 CTTGAAGTGACAAAATGGGAGACA 24 59.42 TGACGAGTAACCTTATCTATTGAGC 25 58.264 246

chr1 7320940 ACACATTGGTCTCATAAGCCTAGA 24 59.283 AGGAGAAGAAATGCCTCTCAAACT 24 59.957 298

chr1 7324138 TTTCTGAGGATGTGTTGGTTGGTA 24 60.141 ACGTATCAAGATAGTGTTGGTGCA 24 60.322 269

chr1 7327041 ATCAACGTAGCCTTCAAAGAGAGT 24 60.021 CTCTCTACTTCCTCATCATCAGCC 24 60.022 251

chr1 7338457 GAGTTGAAGGAATGTGATGGCAAG 24 60.38 TTCTTCGTCTCCTGTCAATCTCTG 24 60.083 285

chr1 7346272 ACTTGTACTCAACCATCACCTCTC 24 60.021 TATGACCTTCTTTGCTCCATCCTT 24 59.772 193

chr1 7352102 CCCTAAAACAAGAACTTTGGTCGT 24 59.663 GGTGATGATGTTGTCTTATTGTAGGG 26 59.739 294

chr1 7352683 CTCGTTCGTTTGTTTATTTGTGACG 25 59.889 AATGCACCCCTAATTTCTCTTCCT 24 60.017 268

chr1 7354863 CGGAGTGAATAAATTTATGTGGAGG 25 58.041 GGAAATGCCCATCACTCTTCTATT 24 58.864 199

chr1 7450199 CCTATGCCATCCACATTAAATCCG 24 59.843 TCCTAATGTGATCCAAAGTACCGA 24 59.287 297

chr1 7456985 TGGACAGGACATTAACGGTTAGAA 24 59.716 AAGATGCCTTGTTCGAATTGTCAG 24 60.083 186

chr1 7463265 CATTTACATGGGTCGGTAGTAAGA 24 58.091 CTCCAATGAAGCAAAGTTTCCTGT 24 59.962 296

chr1 7467267 GTAGGCAAGAAAGGACACACTAGA 24 60.021 TCTGTAGTCTTTGTTTCCTCAGCT 24 59.655 296

chr1 7473172 TTTCTTCCCACCTGCACATTGATC 24 61.589 TGACACCTCCTAACACTTTCATCC 24 60.263 297

chr1 7480307 GTAGAAGTTTCCCAATGTTCTCGC 24 60.143 ATGTATCCATCTTGCATGCCTTTC 24 59.72 292

chr1 7481953 TGCCTTCTACTTCCTTGTTCTCTC 24 60.021 TGTTGGGACTTGGGAAATTACTGA 24 60.141 217

chr1 7483721 ACGTCTCATATCATGCCCTAGTTC 24 60.022 TCGGCTCTGTGATACTACTCTAGA 24 59.656 207

chr1 7484063 GAGGCTTGGGTTGAAAATGATACA 24 59.538 CATACGGCCACCTCATACTATCAA 24 60.021 276

chr1 7492954 GAGTTTTGGAGGGAGAAAATTGCA 24 59.961 TGGTATTGTGGAGTGTTTGATCGA 24 60.263 259

chr1 7494598 TCAGAGAACATTTGCGAGAGAGAA 24 60.022 CTAACAGTTTGAATGGAGCAGAAGA 25 59.297 251

chr1 7500468 AGTGATTTTAATGGTGGCAAGTCG 24 60.083 CGTGTTATGTTGGTTCTTTTCCCA 24 59.963 285

chr1 7500600 GGAAAAGAACCAACATAACACGCA 24 60.498 TGTAACACATTGAAGAGCCAGTAG 24 58.762 117

chr1 7547432 GAAAGGTGAATTGATGGATGGTGG 24 60.142 TTTACCACAACCCATCAGCTTAAC 24 59.479 147

chr1 7547653 TCAATAATAACTGAAGCGGGGTTC 24 59.12 CACCAATCAACACCAACAAGAAGG 24 60.737 205

chr1 7548674 TGCTGGATATGTTGGGTAAAGGAA 24 60.018 AACTAGCTTCTCCTTTCGTCCCTT 24 61.544 221

chr1 7572620 TCTTTCTCGGTTTGAATTGTGGTG 24 59.964 TGAAGCAAATGTGGCCTTGATATC 24 59.902 195

chr1 7573696 TGGTTCCAGTGTGATTAGGTTTCT 24 59.896 CCTAGTTAGAACCCGTTTAATTCCC 25 59.181 180

chr1 7671153 TTTGTCGCATCTTCAAGTTGATCC 24 60.083 AAGCTCTCAATAATGTCCAACCCT 24 60.018 294

chr1 7671367 AGGGTTGGACATTATTGAGAGCTT 24 60.018 GGAAAGGGAAGGAATAAGGGGAAT 24 60.079 202

chr1 7673095 TTCCTTCCACTTAATCCTTCTCCC 24 59.772 TCACCAGCGGCTCTTATAACTTTA 24 59.839 184

chr1 7683754 TCTGCATGTTACCTTCTCTTTCCA 24 59.958 CTTTATATTACAACCTTGCACCCCA 25 59.345 212

chr1 7691717 AACCTAATTTTCGACCAAGCTCTC 24 59.304 GAGAAGATTGTCCAAGGTTTTCTCT 25 58.999 171

chr1 7696501 GTAATATTGCAGGGTTCATGATTCC 25 58.318 TCCATTTGTACACTTTGGCCTCTA 24 59.958 89

chr1 7697982 CTCCATGACTGCTAGCTCCTTATT 24 59.96 GTAGACACACGCTCAAGAAACAAA 24 59.966 152

chr1 7698140 TTTGTTTCTTGAGCGTGTGTCTAC 24 59.966 AGCTCCCACCTTAATTTACCTGAA 24 59.709 289

chr1 7705395 CCCACCGCTCATTCATTAAAAGAT 24 59.661 TTTCATCGGACCGTCACCATATTA 24 59.9 260

chr1 7714532 CCTCCCTGCAATATTTCCTCCTAA 24 59.895 GTTCCATCTCATTTTCGTCCAACA 24 59.785 159

chr1 7782097 ATTCATCATGTGTTGGCATTGAGG 24 60.142 TGTTGAGGATGTGGGAAGTTAGAA 24 59.65 286

chr1 7789595 TTTCCCCACATCATTTCCTTTTCC 24 59.714 GAGAGATCCAGTTCAAATTTCGGT 24 59.062 255

chr1 7790754 CCACTTTGCATGAGAGAAAATGGT 24 60.022 AGCAGTGTTGTTGTCTGAAACTTC 24 60.143 97

chr1 7791790 ATTTCTCTTCCTCTTCCACGACAA 24 59.96 CCTTCCACTACGTACCTTTCATCA 24 60.082 291

chr1 7798032 CTAATTGGGTAAGGTGGTGTCCA 23 59.99 GGTCACGCTTGGTATGTCAATAAG 24 59.966 176

chr1 7821441 CCTAAGTTGACGTCGTGAAAACAA 24 59.967 ATCAGTCATAAATGCAGGTGGTGA 24 60.324 231

chr1 7823388 TTTGGTGTTTAGAAGCCTTTGGAG 24 59.659 CATATTCCAATTAGAGTGCCTGGC 24 59.783 210

chr1 7828933 AAGGCTTGGACTTTGTTGTTGAC 23 60.119 TGTAAGGGTGGTGAGATTTTGAGT 24 59.896 183

chr1 7885438 TCAAAATCGTGAAATGCCCTGA 22 58.847 GCTTTCATGCCTAAACTACGTACA 24 59.372 266

chr1 7905689 CCAAGAGAAAAGCTAGGGAGAGAG 24 60.142 CCCTCTAGATGCTACTGATTGGTC 24 60.022 168

chr1 7978647 TGTTTCAAGGGTCGGTGATTCTAT 24 60.02 TATAAAAGCATGATAGGCCGACGT 24 60.262 273

chr1 8022239 GGTAGACCTTGCAAACTTGAATAGG 25 59.876 TAAGTCAAAGTTAAGTGGGTCCCC 24 60.202 166

chr1 8023845 GAATTTCGAAAGCAGCCTCTCT 22 58.994 AGACAACGGGAGAAAATAGCAAGA 24 60.263 160

chr1 8071584 GCTGGACTCAAAATGACTCAACAA 24 59.963 GTGGTAATGTGCTGCAAATTCTCT 24 60.083 275

chr1 8080611 GCCAGTATCAGAATATTTTGGCGT 24 59.724 CCATACACGCACGAAACAAACATA 24 60.378 248

chr1 8080918 AATGTGTGAGCTGTCCTTTTCATG 24 60.023 ACCAAACTTCCTAATCAAGCACAG 24 59.48 267

chr1 8088456 TTGATGTGTGACTTTGGACAACTC 24 59.665 AGAAACCACGAACAGCTACAAATG 24 60.024 160

chr1 8090004 ATACAAAGCAAGAAAATGGGGTGG 24 60.02 GTTTGGTAACCGTTTAAGCCTCAT 24 59.783 272

chr1 8090740 TGTAGGAAGACATTGATGACATGGT 25 60.046 GACCTCCTGATGCACCTATTCTAG 24 60.022 173

chr1 8095819 GTAAAAGACGGATAAAACAGGGGC 24 60.143 TGAAAAGAGTGTGGGAGAAAGTCA 24 60.081 221

chr1 8099005 TCATGTACAAGCACTTACCCAA 22 57.641 TGTCTTGCATGCATTTAGGAGAGA 24 60.324 225

chr1 8099932 TGGTATGTCAATATTAGTCGGCGA 24 59.721 CAAGTTTTGTCGTTCTAGTCACCG 24 60.318 195

chr1 8106058 GTCGTGCTGTTCTGAATTTAAGGT 24 59.787 GAAAGAGATGACGTGCACACATAC 24 60.202 130

chr1 8112371 GCAGTTGTGGATGGTAGATGATTG 24 59.964 TGTTAAAAGCACACCACTCAAAGG 24 60.142 296

chr1 8114379 CTTAAGCCCTCAATCCGCGAG 21 61.136 CATTGTTGGTTGCTTTGGTTGATG 24 60.025 211

chr1 8127916 CACAATTCCACTAACACCACCAAA 24 59.901 AGTCCATGAGTTGTTAACCATACCT 25 59.749 247

chr1 8129355 GCTACCATAGTTCACTCCACTCTT 24 59.839 GGTAGTCCAAGAAAATTGACCCAC 24 59.782 263

chr1 8145567 TAGATTTTGAAGGGCGTGGAAAAC 24 60.023 ACTCCGTCTAGAAGATCAGTGAATG 25 59.933 289

chr1 8148320 AAGTAAGAACGATAACCCCACACA 24 59.959 ACACGAAAATAGAAGGGGCAAAAG 24 60.022 109

chr1 8213411 TCTTCATCACCCAAGAATAGCTCA 24 59.529 TCTCTCTCTCTCTCTCCAAAATTGA 25 58.748 300

chr1 8218703 CACACACACACACACACAAAGAAA 24 60.791 CTCTCCTCTCTCTCTCTCCTTAGC 24 60.504 251

chr1 8220973 AACTAAAGTGACGTGGTTCATTCG 24 59.789 TGTTTAGGAGGCCGTAATGAAATG 24 59.362 297

chr1 8221223 CATTTCATTACGGCCTCCTAAACA 24 59.362 ACATATCTCACCCACTACATGTCA 24 59.038 174

chr1 8222931 GCAAGAGAGGTTATTTTGGAGAGC 24 59.904 CCCTACTTCTTCTCACCCTTCAC 23 60.056 163

chr1 8251788 AGTGTTGGAGGGAGAGTATTGAAC 24 60.02 AATCCCAACTTAAATCAGAGGCCA 24 60.264 171

chr1 8252920 CCTCTTTCTCTAGCTTCCATGGAA 24 59.837 ATCAGAGGGGAGTGTCAATCAATT 24 59.771 219

chr1 8255323 ACTGAAGACAAAATAAGGCATGCC 24 60.082 TGCACCCTTTTCATAAGCAATCTC 24 59.842 273

chr1 8265679 GGGGAGTGAGGAATAGTGACAAAT 24 60.08 TGATGAGAAACAAGAAGGGTGACA 24 60.142 264

chr1 8269586 TTTTAGCTACAATGATGGTGGGGA 24 60.018 TGCTCACTCCACCTACATGTAAAT 24 59.775 221

chr1 8386363 CAAGTAAATCGGTCCAAGCTCAAG 24 60.143 TTGAGCTTCGATCTTTTGGAATCC 24 59.604 265

chr1 8386742 TAGTATAGGTCTAGGAGGGGCTTG 24 59.956 TGGAACATGAGATGACAAGAAGAGT 25 59.988 159

chr1 8390594 TCTTTAACCTCCTTCATCCAACGT 24 59.959 AATGAAAGCGGGCGTAATTAGATC 24 59.787 273

chr1 8396521 TCAAGAAACCAATCCATAGGCTCA 24 60.019 CAATAAAGTCAGCAAGTTGGGAGG 24 60.083 269

chr1 8458876 GACAAATTTCAAGTTCCCTGCA 22 57.679 GCTCCAACCGTTTCATTATGTCAT 24 59.904 226

chr1 8461082 GCAACAGCCATGAGAAATTGAATTC 25 59.937 AGGAGAGAGATGGTGGAAGGATAA 24 60.078 203

chr1 8467305 GCAATCGAGAGTGTCTTTACCAAT 24 59.368 GCACACTTAAAACAACGCACATG 23 60.061 300

chr1 8535363 CAAACGTATAAAGTGGCACCATGA 24 59.845 TTGTGTTCTAGTAACGCTGATGGA 24 60.022 255

chr1 8543673 GGTTCATGCTCGACACATTTTG 22 58.763 GAGAAAACTGATATCCACCACGTT 24 59.064 300

chr1 8546830 AATTCTAACTCACTTGGGGCTTCA 24 60.203 GAATTGCGAGCTTTGATCAACCAA 24 60.852 249

chr1 8551545 GGGATATTTGCAAAATTCCGAACG 24 59.497 CAAAATGGGCAATCTCATGGAAAG 24 59.126 300

chr1 8566651 CTGTTGTAGTGTTTCAAGAAGGCA 24 59.665 GGTGAATTTGACGATTTAGGGTGA 24 59.302 296

chr1 8569340 TCTGTAACCTTCTTTCGACGTCTT 24 59.963 ACCGTGAATATGACTTTCCCTCAA 24 60.02 300

chr1 8569675 CGCAGCAAAACAAGGATCAGATTT 24 60.853 ACTTGGCGCAAATAAAACGT 20 57.214 300

chr1 8576005 GGCTTCAACGACCTGAGATTAATC 24 59.729 GGACTGCAACTACTGGACTTTAGT 24 60.263 117

chr1 8580125 AGGGCTATCAGACGACTATCAAAC 24 59.962 CTCCCAACTTCAATACATTTGTGGT 25 59.756 283

chr1 8581197 TAAGGTCTGAAGCGCAAAACATAC 24 59.847 TGGTTGGTGTGATGGAAAGATTTG 24 59.961 270

chr1 8581696 CATTCCCTGACTCCTCTTCTTCTT 24 59.775 GATCATGATGAGAAATGCAGCGAA 24 60.024 183

chr1 8584875 ATTGGCATGTACTTAGCACCACTA 24 60.081 CTTCTTTTGACGACGGTATGGATG 24 59.967 286

chr1 8591225 AACTACCATGTCTTTGCTTGTGTC 24 59.724 AGATGCCCTCAAGATCAAGAAGTT 24 60.019 200

chr1 8593085 TCTAAAGCCGACAGTTAAGACGAT 24 59.843 TTAGGGGATTTGGCATTTGAAACC 24 60.02 251

chr1 8597891 CATCAGTATAGAGAGTTCGACCGG 24 60.084 TATCACGTGGAAAATGACAAACCG 24 60.084 229

chr1 8606891 ATAACTCCCAAACGCCATATACGA 24 59.961 AAGAAGATTAAAGGAGTTGTCGGC 24 59.304 225

chr1 8611417 TCATTCTCACATCCAACTAAGGCA 24 60.019 TTCCACTACCTCCATCGGATTAAC 24 59.9 149

chr1 8611538 ACAGAGAAAAGTTTGAAGGCAGAC 24 59.663 AGAGAAAGAAAGTGGGGTTGAGAA 24 59.834 263

chr1 8614300 AGAAGTGGGTGAAGAATGGGTTAA 24 59.894 TGAGAAGATAAGAGAGGGAAGCAA 24 58.974 199

chr1 8616622 TGAACTGATGATGTGATAGGACAAC 25 58.891 AAGACTCTTAGGCAGAACAAGCAT 24 60.263 291

chr1 8620661 GAAGGAGAAGTTGAAGAAAACCGT 24 59.425 CCCTTTCTTCTTCTTCCGTATCCA 24 60.081 148

chr1 8626409 GGTAGGTAGGTATGCACTAAGGTC 24 59.72 GTAAAACACTTCCACACACAACCT 24 59.843 251

chr1 8627054 TTGACTTGACTCTTGCCAATCATG 24 59.783 GCTAAACCCTCTACCTATCACCAT 24 59.406 262

chr1 8629644 GATGGAAGCAAATAAGAGAAAGACG 25 58.518 CGTGGTTAAACTCAATTGTCATGTG 25 59.375 274

chr1 8634098 AAGCAAGAAGTTAGAGGTGGAAGA 24 59.652 GCCCTTCAACTTCATACTCAACAG 24 59.845 192

chr1 8637610 GTCATGAGGGAGTAAAAGGATATTCTC 27 59.124 GGAACAGGAAAATATGGGTCTTATG 25 57.723 300

chr1 8638874 CTCTTCTTGGTAGTGCTCTAGGAG 24 59.662 AGGGAGAGTCTAGCCTTCTAAGAG 24 60.141 251

chr1 8640231 TCTTAACAAGTATTCCACGCCTCA 24 60.021 ACTACCTTCATCATGCTGTTCCAA 24 60.263 240

chr1 8640420 GCATGATGAAGGTAGTATGAGTTTG 25 58.047 GGGAGGGCGGCAATTTAATTTTAA 24 60.383 196

chr1 8643316 GGCTTTATCATGTTGGTAGTGAGC 24 59.964 TGCCCTTATAAATATGCCCTTAGG 24 58.173 277

chr1 8643436 GGCTTTATCATGTTGGTAGTGAGC 24 59.964 TGCCCTTATAAATATGCCCTTAGG 24 58.173 277

chr1 8650606 TGGAACTTCATCACCCTCTACATT 24 59.464 GGCACATCAAATCTCATTGCACAA 24 60.857 276

chr1 8653931 GGCGTCAACTGCATCAACAAAATA 24 60.85 TTCACTGTACACCCACTTTTCTCA 24 60.081 181

chr1 8654601 AAGTGAACCTTCCCTAGATGCTAT 24 59.028 TTACTTCTGAGGTGTTTGGGGAAA 24 60.08 292

chr1 8660251 TGAGCCCCAATCTTTACCCATAAA 24 60.017 CTGAGGGCAATAGAGAAGGCAATA 24 60.202 248

chr1 8665338 CTGGATCACAACATCAATGACACC 24 60.143 CATTGGTTTTGTATTGAGGGTGCT 24 60.021 115

chr1 8673471 TGACTAATCTCACAGGACAACTCG 24 60.083 AGGATTGCTTGCCATAGGTTTCTA 24 60.08 292

chr1 8680548 GTCTAGTTAAGCTTGTCAATTCTCTCC 27 59.723 AAGCACGACAATGTAGATGGAGAA 24 60.323 186

chr1 8680930 GTCTCTACGATGTATGGACCAAGG 24 60.262 GATTCTCTTTGCACCCCTTTTGTT 24 60.202 275

chr1 8681819 ACCAGGTACCTTGTCTTTCATTTC 24 58.929 ATGAGGGTAAGATTTGTCAGGCAT 24 60.08 288

chr1 8685762 CACGCCGTAAATGTCATTGATACA 24 59.967 AAGTAGGTCATGCTTCCAGTGG 22 60.027 183

chr1 8691188 GAGACTCTTGTATGATGTTTGAGAC 25 57.481 GAGATTCTCAACCTCTCGACATAT 24 57.674 203

chr1 8691623 TTGAACCAAATTGAAATCGGACCG 24 60.558 GTAGATCGATTCGGCTTCAATTCA 24 59.255 221

chr1 8693567 ATGTTACTCTTCCATTGCCACTCT 24 60.019 TCGCGTCATATCTACACTACTTCC 24 59.787 209

chr1 8703423 TAACTTTTCTCGATGCGGTCAATG 24 59.907 TTGAACTGTCCCTGATACAATGGA 24 59.712 149

chr1 8706928 CCCCTTTTACTTCATTCTGCCATG 24 60.142 CACCACTCCTATGATACAACACCA 24 60.081 300

chr1 8721649 AAAAGTGAAGTGTGTGGGACCC 22 60.949 ATAGGTGTTTTGAGGTGAAGTCGT 24 60.202 163

chr1 8746178 AAACAAAAGCACAAGGAAGACC 22 57.877 GGGGTTTAGTAGCTAGGTGGATTC 24 60.202 164

chr1 8746588 TCAGGATCTCAACTCTCACTCTCT 24 60.019 TTGTAGGTGAAAAGCAATTGTGGG 24 60.202 174

chr1 8772002 TCTCGTCATTGTAAGCTTCGTGTA 24 60.083 CCAAAACTTCTCGGATAGGGTTGA 24 60.565 241

chr1 8777936 TCGTTTGTTGTCATGTCTCCTAGT 24 59.961 GTAGTCAAACACCATAGAGAGAGCT 25 59.874 281

chr1 8812855 TACTACGCAGCTGATTGATAACCA 24 59.901 ATCAGAGACGGTTATTACAGCCAA 24 59.838 221

chr1 8817200 TTTGCCTGCGGTTTGATCCTATTT 24 61.593 CCTTGTAGTTTCTCAATGGTTGCA 24 59.722 300

chr1 8817489 TGCAACCATTGAGAAACTACAAGG 24 59.722 CCCTCTCTTGAAATTCAGGTCGTA 24 60.082 263

chr1 8832615 TGGCCATTCTCATTATGCTCGAAT 24 60.748 CTCGTTATCCTGCAGAATAAGAGC 24 59.316 300

chr1 8833335 CACGTGCTATGTAGGATTTAGGAT 24 58.216 CTGAAGTGCACATGTCAATTACGA 24 59.848 256

chr1 8838473 TTAGCTCCCTTCCACATACTTGAC 24 60.081 GTCTACCAATATGCATGAAAGCCC 24 60.023 278

chr1 8852891 CAGTGCCCTAAAATATTGGAACCC 24 59.901 CATAGATTCATAGATCGGCACAGC 24 59.434 130

chr1 8856719 TGTTGACGTGTATAAACTGGCA 22 58.27 AGGACACACTACTACAAAACTGACA 25 59.872 261

chr1 8857036 ATCTCTCTTAGTGAACCGCGTTAG 24 60.202 GCTCTTTAGTGCGAATGATCAGAC 24 60.026 130

chr1 8888420 GCCAAATCATCGTGTTAGTTCTTTC 25 59.205 AAAACATAGCTTCCCACACCCA 22 60.158 250

chr1 8890870 GTTGATGGGGAAGTTGGAAAATGG 24 60.563 CCCCTGTCCTCTAAATGTCTTCTC 24 60.142 267

chr1 8891042 GTTGATGGGGAAGTTGGAAAATGG 24 60.563 CCCCTGTCCTCTAAATGTCTTCTC 24 60.142 267

chr1 8892184 TCCACCATGTTAAACCTAGCTACC 24 60.081 TCGGTTCTTACTTGTTAACTCCTCA 25 59.697 292

chr1 8892343 TCCACCATGTTAAACCTAGCTACC 24 60.081 TCGGTTCTTACTTGTTAACTCCTCA 25 59.697 292

chr1 8892853 TACTGGGACCATCAACATCAACTT 24 59.957 GACCAGTCAAACCCCTTAATTTTG 24 58.462 236

chr1 8897977 AGGCCATTGACACTTTTGAAGAAG 24 59.962 GAGATCCACAATTTACTAGCTTTGG 25 57.979 156

chr1 8902877 AACCCGAATTATTCAACCCAAACC 24 60.021 CTTAACACTGGATTACTTGATGCG 24 58.2 300

chr1 8905365 CTGCGGTTTGTTTGAGGTGTAAAT 24 60.499 TTTTCAAGAGGGACAATTCGTG 22 57.171 248

chr1 8909562 AACACACAGCCAGCGATAATATTG 24 59.964 GGCTTAGATGTGGAAGACGATTTT 24 59.364 208

chr1 8922870 ATTTTAAGCCAGGAGAGCAAAAGG 24 59.779 TTTCTCTGTTCGATTGGCTCCATA 24 60.081 188

chr1 9052118 TCTTGAGCTATTCTTCTCCTTCACA 25 59.518 TAAACAAACCCAACCAAGCCTTAC 24 59.9 156

chr1 9330766 TTGGTGGGTATACTTCCTTGTTGT 24 59.895 GAGAAACTCACCACAATTATCCCA 24 58.747 185

chr1 9435569 TATAAAGAACCCTAACCCCTTGCC 24 60.08 GGTGCATTTGGAAAGGGAATGTAA 24 60.021 274

chr1 9478101 TAGAATTAGGGGTGAAAACGGGTT 24 59.957 TACAAAATGTTCAACCCGACAACC 24 60.202 149

chr1 10243601 ATGCTGACACCCGGGTTTTATATT 24 60.63 ACATATGATTCGAGGAAGTAGGGT 24 58.851 216

chr1 10264257 CACATGAACCTCCAACAAAGTGTA 24 59.421 GAACCATGTCATTTGAAAAGAGGAG 25 58.615 241

chr1 10772288 GTGTTGAATTCCTGCACATCTTCT 24 59.783 CGATAGCTTGGATCTTCGTAAAC 23 57.437 258

chr1 10773541 GATCTGTTAACGTTACTCTTGCTCA 25 59.138 AAACTAACCACATTCCCCATTCCT 24 60.203 267

chr1 10824993 CCATTTTCTTGTCGTGCATAGTGA 24 59.846 AGAAGAAAGAGAGAACCACCCTTC 24 59.959 251

chr1 10829110 CGTGCATGTATGGCCGATTAATAA 24 59.847 GGTGTAAAAGGAGGTAGTTGGAGT 24 59.959 290

chr1 10834457 AGATTTGTCAAAAGAGCAACGGAG 24 60.024 TGAGTCCATCCCTTTCATGAACAT 24 60.018 197

chr1 10835520 TTTACCCCTAACAGCATACCCAAA 24 59.956 CATATATGAGATAGAGGATGGAGACTC 27 57.711 268

chr1 10836940 GTTAGGGACCCAGTTGAATACAGT 24 60.02 GGGAGCCTAGCAAGAAGAGTAATT 24 60.142 278

chr1 10839141 GGGAACGAGTGTAAATGCATATATG 25 58.168 GTTGATAGTTCCAATGAAGGCCAA 24 59.538 189

chr1 10844381 ATTGGACTCCCTAAGACTGAGAGA 24 60.017 CTATTGGTTACTTCGGTCTGTTCG 24 59.438 273

chr1 10847550 TATCTCTTGCATGGGGTCTTTCTT 24 59.772 AGAGGTTGGGTCAATGTGGATTAA 24 59.956 175

chr1 10849286 ACCTAGCTAACACGATCAACACAA 24 60.262 CATGCATGTTTGATGGACTACACA 24 59.844 268

chr1 10852940 CGCGCAATCTTTCATAGTTCCTAA 24 59.73 ATTGAAGCTGAAGAGAGTGGGAAA 24 60.203 128

chr1 10856197 ATCGGATGCAATTGAGGAATGATG 24 59.782 TGTTTTGAAGACTTTTGGTTGGGG 24 60.323 291

chr1 10856709 TAGGCTTTAACGTCGTTTGCATAC 24 59.908 ATTGTGCAAATCTATAGGTCGGGA 24 59.897 180

chr1 10856937 TCCCGACCTATAGATTTGCACAAT 24 59.897 CATGAGTTGGGTGTTGACATAAGA 24 58.997 237

chr1 10860667 TCACCACAGTTAAACTCAACATGC 24 59.964 ATAGAACGGGTTGAGTGGATTGAA 24 60.02 240

chr1 10861488 TAACTTCAATTGCTGCATGTCACC 24 60.321 CAAGGGTTGATGACATTAGTTTACC 25 58.147 238

chr1 10867257 TCAAGCCAAATCCCGAAATAATGG 24 59.901 GATCCAATGAACTCTAACTGCTGC 24 59.965 298

chr1 10868298 CCATCTTCCAGCAAAATGTTCCTT 24 60.021 TGAAAGATCTGCATGAACAAGAGC 24 59.845 186

chr1 10872361 CCAATACCCAAAACAATTCGGTGA 24 60.022 CACACACAAGATTCAGATGCATCA 24 59.845 262

chr1 10873424 AGAGAATGATCCCCTAACATGAGC 24 59.959 TTAGTGTTGCTGGAGTACTTGTTC 24 58.946 276

chr1 10873722 TACTGTGTCCATTGTCCACCATAA 24 59.713 CTGATGGACTGTGATAGAACTCTA 24 57.049 173

chr1 10891973 TGCACCTTGTTATGATCGTAGTCT 24 59.84 TCTTAGTTGCCATTGTCACTCCTT 24 60.202 192

chr1 10897423 ATGTAATTGAGGTGGGTGGGTAAA 24 59.955 GAACGAAGAAGTACTGACAGGCTA 24 60.083 261

chr1 10908766 CCACAAGGTCACCATAGTCTAACA 24 60.021 GACTGACTTTTCTGAAGACCAACG 24 60.026 199

chr1 10909371 TTTTGGTGATCTCCTTTGAACGTC 24 59.725 TGAGACAAGAGAATGACGTGCTTA 24 60.022 254

chr1 10909500 TTCTGATTGGACCAAAGTTTCACG 24 59.964 TATTATCAATACCCCACCCCACCC 24 61.508 282

chr1 10912377 TCCCAGTAGTGCTCATTCAAACAT 24 60.263 AAGGAAAAGCTTTGTCAGTGATGG 24 59.962 289

chr1 10916745 ATCTGTCCACTTCAAGGCCTAAAT 24 60.018 ACTCTCCTCTGTAATAAAGGTGGC 24 59.837 278

chr1 10917744 GCTTCTGAAAATGCCGCAATTATG 24 60.026 GTTTGCCACCATTCAGCCAAAAT 23 61.056 188

chr1 10918608 ACCCTAATCACCAACAAAGGATCA 24 59.956 GGATTTTGACTGTTGGTGCAAGTA 24 59.963 250

chr1 10919630 TACTGCTGCTACTTCTGGATGATC 24 59.961 GATTTGCAGCAACTCTAGACGATG 24 60.26 176

chr1 10920538 GACTATGAACACTTCTTCCATTTGG 25 58.154 TAGGTTGGTTGTCGAGTTGAAGAT 24 59.96 209

chr1 10924422 CACCAATGCTGTCAAATTCTCAGA 24 59.783 TAGAGTGCCATGTAACAGGTTTGA 24 59.959 210

chr1 10954726 AATCATGTACAGGAAGACGAACCA 24 60.021 CAGAGATCGCAATTCAATCCAACA 24 59.905 283

chr1 11128330 TCCACCACCATGAATCAAAACTTG 24 59.961 AAATTCCTAACTCTCCTCGCTTGA 24 59.777 273

chr1 11128702 GCGAGGAGAGTTAGGAATTTTCAG 24 59.431 AACGCCTAACATCCAAGATCTTCT 24 60.081 300

chr1 11130962 GTTAAACCACTCAGACCACCTACT 24 59.96 ACGATCGTGTAGTTTAGCTGTACA 24 59.845 285

chr1 11153667 AGTTGCAGTGAAAATTGTCTGACC 24 60.202 ATTCTGTGTGATATTTCCAGGGCT 24 60.08 288

chr1 11173176 TGAACGTGCCAAAAGTACTCTAGT 24 60.202 TCTGGTGCACTTAAATATCCCTTGA 25 60.045 300

chr1 11173751 TCCTCCGTGTGTGTGGTTATTTAT 24 60.02 ACTTGGAAGAGAAATACGACACGA 24 60.023 141

chr1 11173973 CCGCCATCATTATCATCAACAACA 24 59.963 AGTGTAAAGGAGAATGGGAGCAAT 24 60.018 206

chr1 11184265 CTTCATTTGACAACCCTCGAGATG 24 59.906 TGCATTCTTGGCTTGTCTACTACT 24 60.02 252

chr1 11184455 AGTAGTAGACAAGCCAAGAATGCA 24 60.02 CAACTATTCGATTTCCTCAACCCA 24 59.059 130

chr1 11185240 GTTAAGTGATCATGGGTGTGCTTT 24 59.782 GTGGCCTCTCTCTCCTATCTCTAT 24 60.018 184

chr1 11185496 TGCACTAACTGAACAACTCTTCCA 24 60.384 CCTGATGTGTTAACTATCCGTCGA 24 60.202 299

chr1 11190277 TCACGAATCTTCTTAACACCAGCT 24 60.262 TTCACTCATTCTCGACTTTAGGGT 24 59.473 225

chr1 11192357 GCTCTTCATTTCGGTAAGTGGTTT 24 59.784 GGCAACGTCTACAATCCACAATAT 24 59.425 295

chr1 11192487 GCTCTTCATTTCGGTAAGTGGTTT 24 59.784 GGCAACGTCTACAATCCACAATAT 24 59.425 295

chr1 11192659 TGGATTGTAGACGTTGCCCTAAAT 24 60.324 CGATACAACAACATCCCACAACAA 24 60.024 236

chr1 11193258 ATCTGACTTGACAAGGGTTAACGT 24 60.202 AAAGCACTCACTCTTCAACTTTCC 24 59.663 264

chr1 11195128 ACCACTTGCCACACTTTAACAA 22 58.907 AGTAGGAGCTGACAAACTGAGAAG 24 60.022 241

chr1 11197030 GGTTCTTCCGATGACTAAGTTCCT 24 60.082 AAACACAGATACAGATGAGGGCAA 24 60.263 224

chr1 11202696 AGTAACCCAAACCAACCCTAACTT 24 60.079 AAATATTTGGGTTCGGTTGGTTCG 24 60.321 297

chr1 11203092 ATCTATCCAATCCAACCCACAACA 24 60.018 ATTTAAAAGCAGCCTCGTCAATGG 24 60.381 272

chr1 11204389 ACTCGAATAATCCAACAACCCAGA 24 60.02 CGGGTTGGATTTGGTTGAATTGAA 24 60.502 164

chr1 11337213 CGTCTCTTCTCTTCTTGGTAGGTT 24 59.781 AGTACCATTGATTCCTCTGATGTGT 25 59.809 247

chr1 11346423 TCCCACTAGTTATTGTTCCACCTG 24 60.02 CTTCAACAAAGTCTTCCCTTCCAC 24 59.963 191

chr1 11605234 TTGGAGGAAATGAAGGTGGAATCT 24 59.955 CATTATCCCACCAAACTGATGAGC 24 59.963 247

chr1 11608196 ACTTATTGGGCTTGGTGTCTACAT 24 60.018 TTTATGCTCGGGAGGTCAAAACTA 24 60.02 193

chr1 11610924 CCACATGTTCAAGTAGCAGAATCG 24 60.202 AGTTAATCTCAAATGGTGGGCTCA 24 60.264 291

chr1 11612558 TGCAACTCTACATGTTCGATCACA 24 60.561 GTAGCTAGTTCACTCTTCCGTCAA 24 60.083 243

chr1 11616984 AATAATCAGTGGCGCTAGAATCCA 24 60.202 GTAGCTTGCATAAAAGTGTGTGGT 24 60.023 264

chr1 11618576 GCATTGCAAGATCTTCCATCTCTC 24 60.024 GATCTGGAATGCTAACGGAATTGG 24 60.024 132

chr1 11633163 TTGGTACCCGACGTATGAAGTTTA 24 59.78 CACATCATCCGTTAAAACAAGCCT 24 60.083 152

chr1 11635145 TAGAGGATAGTGTGTCTGGTGGAA 24 60.264 GTTGAAACGTGGAAGAGCAAGTTA 24 59.965 108

chr1 11635790 GTAGCGAAAGATGAATGTTTGTGG 24 58.911 TTACAAGTTATCACAAGTGCAGCG 24 60.084 219

chr1 11636563 AATTGAGAGCTTGTGGTTTGAGTG 24 59.963 ACAGGAAAGAGATGGATGGATTGA 24 59.524 170

chr1 11637891 TGTTTGAAGGCATCCAGGATTAGA 24 60.019 GTATGCTTCTCCTTCAGACTTTTAC 25 57.703 296

chr1 11638394 TAATACACCCTTTCACCACATTCC 24 58.742 TATTGTTCCTTCCACGCATCTCAT 24 60.384 268

chr1 11643190 AAGTGACTAGATGCGTTGAGAAGT 24 60.022 GACGGAGAGATGTGTCATATCGAA 24 60.024 276

chr1 11644180 CCATGCAACTAAAGTGCCCTTAAA 24 60.022 CCAAAAGGCCTATCGTCGAAGA 22 60.418 244

chr1 11651767 ATTCTCGTATCCTTCGTTGGTTCA 24 60.082 AGTGTAGTCCGAAAATTTCCGTCT 24 60.262 236

chr1 11652090 CACTTTTCGCGACAGTTATTTGATG 25 59.719 TCAGAGTCCATTGTATCATCGGAG 24 59.721 298

chr1 11652296 CTCCGATGATACAATGGACTCTGA 24 59.721 ACTCCATGCTTCACCAACTCTAAT 24 60.019 170

chr1 11652596 GCTACATCGCCTTTATATTTCAAGC 25 58.92 ACAACAACTATTAGCTGCCTCGAA 24 60.563 294

chr1 11655464 CGAGAGGAAATTTTCTACTATGAGC 25 57.599 CCCAGTGGCAGATACAAAAGAAAT 24 59.536 263

chr1 11661432 GGAATGAGGCTTAAGTGTCCAAAA 24 59.477 ACATCAAACAATACCTACCCAGAG 24 58.255 113

chr1 11743534 CGGTTTAGCTCTGTTTGTTCCATT 24 60.023 GGTGATTTGGCGGTGAAGAAATAT 24 59.902 101

chr1 11748500 ACTCAATGGTTACACAAGTAGGCA 24 60.202 TCTAAGGAAAGGAAATGTGGTGGG 24 60.508 144

chr1 11762156 GAGAAGAGGTTGAAGGAGTAGGTG 24 60.082 TAACATAGTGGAGGTGCAAAGGAA 24 59.958 263

chr1 11764579 ATAAACTTGGACTCTCTCGCTCTC 24 59.903 GACTTGTTCTAAGCCTATCGTTGC 24 59.967 201

chr1 11765797 GAACGCTAAAAGTAGGCAGTTGAG 24 60.143 AACCTACCAATTCTAGCAATGCCT 24 60.325 288

chr1 11767096 TTATCGAGTCATTAGCCAACTTCG 24 58.959 ATGGTCACTAGAAGCTAGTTGGTG 24 60.081 273

chr1 11769875 TTCTGGCTGAATATGATCCTCTGG 24 59.959 TCTGGAAAACTGGAGTCAAAGGTA 24 59.589 294

chr1 11771512 GTCTGTCCAACATAGTCTATCAGTGA 26 59.904 GCTCTTATCCCTACATTACACACG 24 59.017 282

chr1 11771780 CGTGTGTAATGTAGGGATAAGAGC 24 59.017 TCCATGGTTCGGTCTTATACAAACT 25 60.046 161

chr1 11785073 TACGATCCCTTATGATTTGGACCC 24 59.959 GCAAGTTGAAGCGATCCATTTATG 24 59.263 296

chr1 11791691 TCAATCTTCCACTTCTGTTCCATCT 25 59.987 GGATCCCACAATCAATATTCTCATCTC 27 59.664 265

chr1 11805633 AGCCAGCCATGTTTCTTAGAAGA 23 59.991 TTTGCAGGTTGTCAAATAAGGCTG 24 60.501 256

chr1 11827939 CTTGATACAACCGTTGCACTTGAT 24 60.083 TGGTATCAAGTGTATCATGGTGCA 24 60.081 83

chr1 11969628 TAAATGAAGTGAGTATGGCGGTGA 24 60.081 ACCGGGAACAAATAGCCATAGTAA 24 59.835 290

chr1 11991110 TATAGTTCGTAGGTTTGGTTGCGA 24 60.082 CAGAGTGCAGAAGGAAAAGTTGAG 24 60.024 292

chr1 12804571 CTCAATTTCAAATCCCTCGTTCCA 24 59.541 ATCTTCGCCCACATTTGTTTGTAG 24 60.083 259

chr1 12827688 AAAAGGATAGGACGAAAGCAGAGT 24 60.02 CTCAAAATTCCCTCACTTTCCACG 24 60.32 228

chr1 12831942 TGCTCCATGAAGTTTCTCTCCAAT 24 60.264 GCCATTGACTTGAGACTTGAGACT 24 60.802 206

chr1 13388182 CCATGATGCCTTCTCTCTTCTCTA 24 59.412 TGACAACTTCACCTCAAATCACCT 24 60.385 186

chr1 13577241 TTATGAACGTGTTGGCCATAACC 23 59.559 CCTAATATGGTTGGTGGCGACTC 23 61.05 234

chr1 13582538 GGTCATTGCACTGTTGATTGGTTA 24 60.022 GGACCTACAAATTAAAGCTCCAATG 25 58.436 161

chr1 13598896 CCACATTCAAGCTTCGATACCTTT 24 59.604 TAGCATCGCCTCTCATCAATTGTA 24 59.961 235

chr1 13605558 ACTCTAATGGGTTGTGTGTTGTCT 24 60.141 ACTAAAGCCCAACAACACAAAGTC 24 60.142 103

chr1 13609324 GAGATAGAATTTAAGTTGGCGAGGA 25 58.719 AGAAATTTTGGCGGTGGAGTTTAG 24 60.022 298

chr1 13611285 TCAAGTAAAGATCAAGCTCCTGGT 24 59.713 TGAGCACAACACAATGAAACCTAG 24 59.724 292

chr1 13628938 AGGACACTCTCATCGTATAGGATAC 25 58.825 GGGTAGCAACTCAGTATTGTGACT 24 60.323 299

chr1 13635487 ATTGGTCAGTTCTGATAATGGCAC 24 59.358 TTCTCTAGTGGGCTTTATGTGCTT 24 60.019 218

chr1 13636204 AACAACACCTACACGAGACCTATC 24 60.082 TCATCAACAAGCTTCATCAGGAGA 24 60.02 281

chr1 13636811 GGAGCCAGAAGTCATTAACAGTTG 24 59.845 TGATTAGCTCTCCCTTTCACTGTC 24 60.081 131

chr1 13639683 ACGACTTCACCTCAAAACACCTAT 24 60.202 CATACAGTACACAATGCAACTTCAC 25 58.92 248

chr1 13643901 GCATGGAAAATGAAGCATCTCTCA 24 59.903 CTCGAACTTGATCCACGTTTATGT 24 59.374 268

chr1 13646334 TTGGATCATTGACAACGAGAAAGC 24 60.083 TCCACCTCCCTAATAGAAGCAATC 24 59.652 266

chr1 13648917 GAGATTTCAAATTCATGTGCCACG 24 59.44 CGGAACCAGAAACATAAAACATTACCC 27 61.051 148

chr1 13659957 GTCTTTCGATCGTTTGGTTGACTT 24 60.025 GCCAAACATCCCTCGTTAAGAAAA 24 60.023 235

chr1 13662735 GAGTATTGGAGCACATAAACCTGC 24 59.964 GAGGAGGTAGAGGTAGAGGTAGAG 24 59.715 199

chr1 13663390 CCTCCAACTACCCTTCATAAGCTT 24 60.08 CTCCTGCCATGAACAATTCCCAG 23 61.744 274

chr1 13663939 CGTTGAAGCAGAAGAAAGGAGAAG 24 60.084 CCTCACCTTTCCTCTACTTCCTTC 24 60.081 84

chr1 13678979 ACAGTTGAAGTGAGTGAGTAAGGA 24 59.35 CACACCTTTCTCTTCCTTTTGCTT 24 59.901 117

chr1 13679655 TGGATCCCTCGCTAACTTGATTTT 24 60.324 GGCTCCCTCTTCCCTCTATTTAAA 24 59.587 159

chr1 13681425 TCCTTTCTTCTTCTTCCGTTTCCA 24 60.142 CAATAACCGAACAAAGCTCCAACA 24 60.261 133

chr1 13689572 GCCCAAATCTATCCTAATCACAGT 24 58.375 TGGCATATGATTGATGGTGTCGA 23 60.181 300

chr1 13719865 AGGATGGTTCTACTACTTTCTTCCT 25 58.735 TATTGACAGATTTTGCGGTGGTTG 24 60.321 210

chr1 13719995 CAACCACCGCAAAATCTGTCAATA 24 60.321 TCTCCCCTCCATTTTCCATCATTT 24 60.016 170

chr1 13738212 TCCAACGGTTAGCTTCTCTTCAAT 24 60.263 GACAATTTCCTTTCCAGACATGATG 25 58.672 179

chr1 13784583 ATGTGTTTTCCTCATTTGTGGTGG 24 60.202 CTAAACCAGGGAGCTGTGATATGG 24 60.745 231

chr1 13789592 TGAAGAATCGATTTTACGCTCAGAG 25 59.483 GTGTCCAACTCCTCCAATAATTACT 25 58.584 285

chr1 13821286 TGCCACTTCTACAGACATTCTCAA 24 59.96 TGTGGGAATTGGTGTAGAAGAAGA 24 59.65 141

chr1 13845314 TAGCCTCGTATCCTAGACACAACT 24 60.385 CATGTAGAAGAGATGGAGGAAGTT 24 57.767 287

chr1 13853079 ATGTTGTTTTGTTCGGTTCTCTCC 24 59.964 GAAAACCCTACTCTCACTTTTCGC 24 60.084 220

chr1 13855119 TTCTTCAATTTAGGGGTGGGTTGA 24 60.141 ACGTTACACCAGAGTATATCGAGC 24 60.024 267

chr1 13877178 TTTCTCTATTCCACCAACCGTCTC 24 60.322 AAATTGGTTAGTTCGCGAGGAA 22 58.595 290

chr1 13887411 CATTCAGCCTTACACATTTGACCA 24 59.782 GAGTTGTTCATGGAAGCAAACA 22 57.694 263

chr1 13888490 GTAATCTCCCACATACGACCTACC 24 60.023 TTGTGGGTTTTGTTAGTGAGCAAG 24 60.142 84

chr1 13891387 CCCTGACCAAAATACCACCTTAGA 24 60.019 TTCATTCATTGTTTCGACCCAGTG 24 60.023 281

chr1 13892365 TGGTGGACTATTCTTATCCCAAGA 24 58.715 CACTGCTTGCCAATGTGATAGTTT 24 60.321 244

chr1 13894387 TGTTGAATCCTTTGAATGGTAGGC 24 59.538 GTGCAAAACTACTGACACATGATGA 25 60.049 191

chr1 13898431 TTTCTTTAGTTCAGCGTATCCCCA 24 60.02 GACACACCACATTCACAATAAAGC 24 59.083 168

chr1 13907844 CAGGGTTAGTCTCTAAACTTTGAAC 25 57.577 GGGGTACTGCTAGTTCTTTCTTCA 24 60.02 199

chr1 13910029 TCCTCAGTGTTCTTTAGATTCCGG 24 60.082 TTTGTGATAGCATGCATTCGAGTC 24 59.966 263

chr1 13910138 TCCTCAGTGTTCTTTAGATTCCGG 24 60.082 TTTGTGATAGCATGCATTCGAGTC 24 59.966 263

chr1 13914444 AGTGGAAAAGTTGCTGCTACTACT 24 60.202 GCAGCAAAAGAAGTCGAGATAAGG 24 60.202 197

chr1 13941205 TCAGAGCCATAAATCCAACTTTACC 25 59.114 TTGAAAAGCATCACAAGACCCATC 24 60.022 292

chr1 13945587 CCCTCCTCCAGATTTAAATGCTCT 24 60.141 AGGTAAAGTTGGATTTATGGCTCC 24 58.799 296

chr1 13947786 ATTTTGTGTGGGATGAAGCTTGTC 24 60.262 GGAGAATAGTTAACTTCAGCCCCA 24 60.081 240

chr1 13955036 GCTACTGCCAAGAAAGCGTTTTAT 24 60.379 TTGTTAATATCCCCAACTCCCTTG 24 58.485 255

chr1 13957641 GGCTTCAAAATAGGTGCACAAAAC 24 59.789 TGCATGTCAAGAATGTACGTACTC 24 59.137 124

chr1 13963254 GGCCATGAAAGAAGAAGAAAACCA 24 59.961 CAACAGACAACTTCCTCTTCTTGC 24 60.261 254

chr1 13965021 GACAAAACGAAAGAAGAGGAAGAAG 25 58.577 CGATTGAGCTTCTTCGTTCTTGTT 24 60.084 259

chr1 14386090 GTGTGTACTTAGATTCCGAGACGA 24 59.906 CCAAAACCTTCCAACCCTTAACG 23 60.244 292

chr1 14436047 CGACCATCATCCTCTCCTAAACAT 24 59.96 AGAGTGAGGCAGATTTGAAAGTGA 24 60.202 149

chr1 14477570 GCTATCAACGCAATCAAAGAAGGA 24 59.905 TGAGAATCCGATCGACTCTTTGTT 24 60.082 300

chr1 14481053 AAGTGAGTTGGACAGACTACAGAC 24 59.962 GAATCGCGTCTCAATTCCACTAAG 24 60.026 292

chr1 14483517 CCCATACTCTTCTTCCCTCCCTAT 24 60.451 TTTGTTTATGTTCTCTGCTCCCAC 24 59.481 289

chr1 14516902 TTTCTTGCTTCTCTACCACTCTCC 24 60.021 ACTTTGACATCGTTGGTGTTACAC 24 59.965 198

chr1 14575243 GGGGTTGTGGATTTGTAAAGCA 22 59.369 AGGCATTGCAGTCTATCAACCTAA 24 60.08 86

chr1 14575628 GTATCTGAACATCGACCTCCACAT 24 60.202 TAGGAGTGCTAACAAAATCCCACA 24 59.958 167

chr1 14577303 TAGAGAGAAAGCACCAACACCTAC 24 60.021 AGTAGTCAATAGGCCACTGTTTGT 24 59.958 300

chr1 14595605 TTGACGTCGTTATTTTCCAAGTCG 24 60.085 CTTTTCCTCTCTCTCACAAGCTCT 24 60.021 287

chr1 14595872 AGAGGGATGGAAGCGTCAAATTAT 24 60.141 TTCATCGTCAAACCCACCAATCTA 24 60.263 294

chr1 14595988 AGAGGGATGGAAGCGTCAAATTAT 24 60.141 TTCATCGTCAAACCCACCAATCTA 24 60.263 294

chr1 14597550 AAGATAGCCACCTCATCATCTAGT 24 58.598 TACCCTCTCAATCTCACAAGCAAA 24 59.958 273

chr1 14601710 GTCAGCAACGTCAACACTATTG 22 58.201 CTTTGCTGCGATATTTGGTGTTTG 24 59.91 236

chr1 14608360 GAAAGCTTGATTGTCGCGAATAGA 24 59.967 CACACACAATTCGAGAAGGGTTTT 24 60.202 288

chr1 14611893 TTTTGACCATTTGCTACCCTTTCC 24 59.96 GAAAGCACGAAGAAGAAAGCAAGA 24 60.261 169

chr1 14612650 ATCTGACTTCCTACTTCGTTCGTC 24 60.143 CCGAATATGGTCACTAAAGTCCGA 24 60.202 136

chr1 14618488 CCCTACCCATATAAACACTACCCC 24 59.712 GTTGAGTTATGGAAGGGTTAGGGT 24 60.019 240

chr1 14630351 ACGGTGTGTTTATGTATCGTGA 22 57.823 CTGGATCTTCGATCATGACCTCTT 24 59.961 269

chr1 14631356 GCAACTGTCCAAACTCAATCTGAA 24 59.963 TCTCCCACTCTCTGTTCTTCAAAG 24 59.96 278

chr1 14640967 GGGTCATTTTCCGTATCGACAAAT 24 59.666 CTTCCCAAATGGCTCGAATACTTT 24 59.601 295

chr1 14765408 TGTTTGAGATACAGCTTAAAGGGTC 25 59.062 CAACTCGCATGTCTCCTATACGAA 24 60.498 141

chr1 14777259 TGGTAGACGAAATCAGTTACGATGA 25 59.876 CGAGGGGCATTGGTTACATTTATT 24 59.659 264

chr1 14823448 GGTTCTTTGGTTTAGTTCTACGCT 24 59.246 GGTCAAGATAGTAGTATATGTGCCA 25 57.772 137

chr1 14842760 AGTGGACCAAAGAGAGAAAAGGAA 24 59.834 ATGTGTGTCTACTCTAGGAAAGGC 24 59.839 219

chr1 14842907 AGAATGGACTGAGTAGCATCAACA 24 59.776 GACCCTATTCCCAATACAGAAGCT 24 59.897 240

chr1 14850012 AGAACTAACCTTCTCGCCTTTTCT 24 59.959 AAGGTTGGTGAGGAAGAAAGATCA 24 59.895 278

chr1 14851665 TCTAGACGCTCAAAATACAAACACC 25 59.593 ACTACTCGGATTGTCTCATTTGCT 24 60.081 268

chr1 14864819 GTAGAAGACTTCCTAACTCCTGGA 24 58.737 TCAGGAGTTAGGGCAATCTTCAAA 24 59.957 94

chr1 14872659 TGTAATGGATGTGACCTTCAGACA 24 59.714 ACACTACATCAGATCAAACCAGCT 24 60.02 261

chr1 14878828 CATTTGACGGTTCTCCTTACTTCG 24 59.908 TGACCTTTGCCTTGGATTCTGATA 24 60.019 277

chr1 14882835 GCCTTTAAAGCGAGCTCTTCTTAG 24 59.967 TTCACCAAACACAATCCCAAGAAC 24 60.142 251

chr1 14883447 CTCTCTGATTCTGGCTTGCAAATT 24 59.842 GCGAAAACTTAGTGAATGTGACAAC 25 59.605 300

chr1 14886584 GGCTCCATATTCATCTATCCATCCT 25 59.631 AATTTGATAGTGCGCCAATGAGAC 24 60.202 120

chr1 14902816 TCCTAATCAATTTGTGTCGTTGCC 24 60.083 CACGAATTTAACTCAACTGACCATG 25 58.92 145

chr1 14917254 AATTCCTCGGCTTCCTGAACTATT 24 60.08 CTCTAACAACTCGTGGGCTATCTT 24 60.142 190

chr1 14921064 ATCTCAAAATCACTCCCAACCACA 24 60.448 AGTGGATTGGAGACCTACGTTTAC 24 60.082 259

chr1 14925708 CAATGATCAAACTAAGACGGAGGC 24 59.965 AGGAACACGAGAAGACAAGACTAC 24 60.023 229

chr1 14945495 CGACATCATCCTATCATATCCTTCA 25 57.895 CGTCGGCCATTATGTTTAGTTTCT 24 59.668 191

chr1 14952278 ACGCCATAAAACCCCTCG 18 57.37 GGCGCGGTTGTTTCTTCAC 19 60.37 284

chr1 14957725 CCTTCATTCTGCCTTCATTCCAAA 24 59.779 AAGGAAAGATGAAGTTGGGAGGAA 24 59.894 204

chr1 14967277 TGTTAGGACCCAATTGAGAATGACA 25 60.222 TGTGAATAGGCCTGATGTTGAGAA 24 60.019 285

chr1 14968542 CATTGAGAGCCATTGGAATAGAGC 24 59.784 GTCTCCTATCATGGCAAGTGGTAT 24 59.959 236

chr1 14975694 CTCGATCACTAGTCTCAACGTCAT 24 59.965 TTAGTTTTCGTCGATGTGGTCAAC 24 59.79 257

chr1 15060551 TTTCCCTTTTCTTCTTCCTCCCGT 24 61.984 GGGAAGAAGAAGATAGAGGTGGTG 24 60.142 147

chr1 15146791 CCGATTCTCAAACCGAACTGAATT 24 59.846 TTCTTCTCCTTTTCGTTCCTCTCA 24 59.656 144

chr1 15336827 CTCTGTATCTTTGTTGGACACTGC 24 59.847 ACCCATTAAAAGAAAAGGAGCACG 24 60.022 218

chr1 15454352 GGGACTGCAAATATGGGTTATCAT 24 58.92 AGTAGTGTTCAAAATCCCAGCTGA 24 60.202 113

chr1 15607780 CTAAGTACTTCAACGTTGTTTCGCT 25 60.051 CATAGATGGCAATTTTACAACGTGG 25 59.257 158

chr1 15715429 ACGAACTTCCAACTCTACCATGAA 24 59.96 TTTCATGAATACCAGTGGACCTCA 24 59.712 254

chr1 15719904 TTTGCCTTCACCCAAAAGCTTAAG 24 60.443 AGCCTTACACTTTTAAGCCCACTA 24 59.958 250

chr1 15725227 TGGCCCTATAGTTAAAGACATGGA 24 59.027 GCTATCTGTAATGGTTGATATCACC 25 57.397 218

chr1 15748127 GGTTTCGTCCCATCACAAAGAAAA 24 60.202 GACCCCACCACATATGTCATATCA 24 59.958 185

chr1 15750852 CACCCCTCTAAAATGCACAAACAT 24 60.021 TAAAGTGAAATTTGTTGGGCCCTC 24 59.96 287

chr1 15759460 ACAATTAAGAGAGTGGGTACAAGCT 25 59.987 TAAGAGATTCCCAGACTCCCTTGT 24 60.513 241

chr1 15772066 TGACTCATTTGCTTATTGGGCTTG 24 60.082 TCATTTCCTCTCATGTTCAACCCA 24 60.203 260

chr1 15780010 ACACTCATAGCCCCTAATCTCTCT 24 60.141 CTCATGTTTGTTTGTAGCCCCAAT 24 60.021 86

chr1 15796750 ACTAAAATGAGAGACCGTAGATAGG 25 57.487 GCTTCAGTTGCTTTGTTGTTATCC 24 59.319 204

chr1 15816039 TCTAAGGATCTTGCAAACTTGGAGA 25 59.987 CAACTCTTAACACACGTCAAACCA 24 59.906 267

chr1 15821753 GATTCAGCTCCTCTTTTGCGTAAA 24 59.846 AAAGTGCCACAACAACCTATCATG 24 60.022 217

chr1 15836520 TCTAAACACACACATGGCTTCAAG 24 59.724 GTATTTTCCCCTTTTGACTTCTGAG 25 58.086 282

chr1 15852295 CCTTAGACTGCCCCTACTGTATTG 24 60.202 ACAAACACCAAACCTTTCTTCTCTC 25 59.874 266

chr1 15878944 ACCATTCACCAACCTTTCTGACTA 24 59.896 GCTAAAATGCGCCTTTGTGTTT 22 59.2 233

chr1 15885644 TAGGTTCACAAAATAAGGCATGCC 24 59.84 GGGGAGATTCTCTTGATTTGAGCA 24 60.627 280

chr1 15912245 CCAACAGTAGCCCCTATAAAGTGT 24 60.081 TAATGGCAATCCCTAAGACGAGTC 24 60.202 276

chr1 15922191 TTTTATGGGTTTCTCTCTGGACGA 24 59.715 TAGTTCCATCTCTAACCTCGCAAC 24 60.142 279

chr1 16016825 AAGCAAGAGAGTTTATGTGGCAAG 24 59.783 GGTCATCCCCAAATCCTTCCTAAT 24 60.141 194

chr1 16017422 TTAATGAAGGGATTTAGGTGGCGT 24 60.325 CTGAAGAGACAATTACAAGCCAGC 24 60.143 232

chr1 16017578 TTTCTTGGGCTGGCTTGTAATTG 23 59.993 CAATAGCACTTTGTCAAACCTCTGT 25 59.991 282

chr1 16026770 CCAATTTTGACCTAAGCCGTTACA 24 59.783 TTAAACTTGGAGGATTGGAGGAGG 24 60.018 274

chr1 16042404 GACAACTTGAGACTAAGATCACAGT 25 58.549 GGCCCATCAAACCTTTACATTCTT 24 59.777 266

chr1 16043758 TCTCACCACCAACACTAACTCATT 24 59.897 GGATTGGGAGAATTGAAAGAGCAC 24 60.142 283

chr1 16043995 GTGCTCTTTCAATTCTCCCAATCC 24 60.142 ATGATTATTGGGAATGTGGTGTCG 24 59.42 171

chr1 16047661 CAATTCCTTCACCCAAATGTCCTT 24 59.716 GGCTTTGATTTTGGGTTGAGAAGA 24 59.961 218

chr1 16047900 GCCTATGCCTAACCATGAAAATCC 24 60.022 ATCTGTCTGAACACAAGGGAAAGT 24 60.141 290

chr1 16048469 AGAAGAAGAAGATGGTGATGGTGG 24 60.324 ACCACCGCCATATGATCTGAATC 23 60.558 155

chr1 16132068 CATATGACACGCAGGAATTCATGT 24 59.726 TCTACACAACCACTATACCAGAACT 25 58.985 195

chr1 16247854 AAAGTAACCCCTATTCTCCCAACC 24 60.018 CTCCCATTTTCCCTTCCTTTTCAC 24 60.021 273

chr1 16260110 ATTACTTCTTGGACGGTGCTTTTG 24 60.023 AGAAAATCGTCATAGCACCTCTCT 24 59.596 225

chr1 16280965 TTCTTTAGTGGTTGGCATGTGTTC 24 59.963 ATCCGAATTGAACCCATGATCTCT 24 59.896 199

chr1 16290680 ATCCATCATGTTACCGAGTTGACT 24 59.838 GGTGGGAAGGAAGAGAATGTATGA 24 59.836 84

chr1 16291198 TGGAGTATGCTTTGAGAGTTACGA 24 59.539 CCTGATAGCCTAATCTCGACACAT 24 59.78 222

chr1 16303131 TCCAATGAGTAAGGGTTTGTGGTT 24 60.387 TCCTCCAACTTATCGATTGAGTGC 24 60.681 285

chr1 16308939 TCTAACTCCATTCTGCTGCTTCTT 24 60.02 TCCATAGCATCCAATCGGTTAACA 24 60.142 206

chr1 16318608 TCCAGATTACAGTAAGAGAGCACG 24 59.903 TTGGGGATAACTTCTAGCCAATGA 24 59.523 280

chr1 16337725 TCACCTTACAACCTCAACCATCAT 24 59.957 AAGATCTTGGGTGCTTGAGTTAGT 24 59.958 235

chr1 16341521 GGCCCACTTTATAAAACAGAAGCA 24 59.78 TGGCTAGATTGTCCACTCATGATT 24 59.835 289

chr1 16344698 GCTACAAAATGACGTCAACTCCAA 24 60.024 ATCACTTGTGTCACTCTTTGTTCC 24 59.424 280

chr1 16365810 CCTTCAACACTGTTCTAAGTCTGG 24 59.248 TGGCCCCTTAAGAAACTACCTAAC 24 60.019 232

chr1 16373533 AATTGTTCATATGGAGGAGAGGGG 24 59.895 TGCTGTAATGGAACGATGATTGTC 24 59.667 282

chr1 16385692 GGTTGGAGGGTTGAAAATTTTGGT 24 60.385 GGGTTGGGTTGTGGGATTGTATAT 24 60.633 243

chr1 16400470 CACTCTATTCCTACCATTGATTGCA 25 58.942 GTTTTGGAGTGTGTTGCATTATGG 24 59.314 284

chr1 16401736 AGTTCTTGTTCTTCCTTCACCCTT 24 60.08 TGTGATCCGTGACATTTTGTGAAG 24 60.024 300

chr1 16410190 TCTAAACACATCAACAACTAGCGC 24 59.848 TGGTTACTACGTTACTCTTGTGAC 24 58.238 270

chr1 16414003 CCAGAACATGAACAGCACTTGAAA 24 60.202 AAATCTGAGAACAAACCTGCAGTG 24 59.963 291

chr1 16424404 TGGTTTTAGGGTGTGGGCTATTTA 24 59.956 GTGTGTTTGACCAAAGTGCTTA 22 57.636 152

chr1 16427947 GATAAGACGACTCACTATGAAATCC 25 57.139 TCCAATGGAGGGTTAATATAACGC 24 58.69 204

chr1 16456876 ATTTTCCAAACGCCCTTTTAGTGG 24 60.502 CCATTTGATGCGATTTATGTGC 22 57.04 184

chr1 16462492 AGATATTTGTGGTCCCTGTTGT 22 57.342 CAAATGAACGATCTCCTCTTGTCC 24 59.669 248

chr1 16478486 GTGTAAAACGAGCGCTCAAAATTC 24 59.913 ATCGGCGATGGAGAATTAGGTTAA 24 59.96 243

chr1 16494625 AGTCATGTATGCTTACCTCTATCCA 25 58.922 ACCAAGAGAAGGATATGTATGCCA 24 59.34 236

chr1 16495465 GCAATGCCTTAAACCAAATGCCAA 24 61.577 AATGTACGAACCCACCCTCTAATC 24 60.142 275

chr1 16496427 GACAAGATATTGCACAACGTCTCG 24 60.491 TCATATTTCAAACCCGCATGTGAG 24 59.904 230

chr1 16499842 GACTCAACATTGTTTGCGACCATA 24 60.084 AGGGCTAAATGATGGGATATTGGA 24 59.392 185

chr1 16501962 GCCCTCCTTATCGTTTCTTCATTG 24 59.964 CGGTGGGGAGTGGAATAATATGAT 24 60.019 217

chr1 16507646 GCAGAAAACAATGACTACTTGGCT 24 60.023 AAGAAAACAATCTCACTGCTCGAG 24 59.55 208

chr1 16511564 CGCACCAAATTATCCAAACCCATA 24 59.901 TGGGTTTTCTCTCTCTCTCTCTCT 24 59.956 130

chr1 16513717 CACCGACCTTGTATGACATCAATG 24 59.966 GAGTCATTCTGAAGAGGCAATATGT 25 58.946 289

chr1 16522763 ACTACCGCTCACTAAGTTTTGACT 24 59.961 GCCTAGTTCAACCTAACCCCTTTA 24 60.019 288

chr1 16522944 AGAAAGACAAGCTACCACACGTTA 24 60.202 CCCTAACGCCTCAAGATGGAAC 22 61.001 286

chr1 16763252 GGAAGCTTGGAAAAGAGGTAGGTA 24 60.02 GGATCGTGATGTGTTTTCTCTTGG 24 60.143 199

chr1 16909800 ACACCGTTCCATCTAATGTGAGAA 24 60.021 TCACGTGGTTGTATAGGGAATTGT 24 60.02 286

chr1 16971872 GATCTTCTTTCACCCTTCACCTCT 24 60.02 CTTCGTCATCACTCTCCCACTCTA 24 61.164 202

chr1 17080678 AAGAGATGAAGTACGGAACACACA 24 59.961 CTCCCACTCTATCCCTCCTCTATT 24 59.955 263

chr1 17080920 AATAGAGGAGGGATAGAGTGGGAG 24 59.955 GAATACGTAGGGGAAGTGGTGATT 24 60.142 136

chr1 17122480 GTAGCCCACAAGTTAAAACAGCTT 24 59.962 AGGAAAGAGGAAGAGTGAGAAAGA 24 58.849 225

chr1 17222180 GAGGGAATCAGGCGAATCAAATTT 24 59.901 CCATGTGTTCAAGGGCCTAATATT 24 59.105 273

chr1 17287479 AACACCTTCCTTACACAACTGACT 24 60.081 AGTCGCCACCAATCATATTAAGGT 24 60.141 105

chr1 17298411 AGACCAGGTAAAATAGTGTTCTGCT 25 59.987 CTGGCCTCCTCAATTATCTTGTCT 24 60.142 164

chr1 17301023 GAAGAACACGAAGGAGACAAACAA 24 59.668 GTTCATAAAGTCTTCCCACGTGTG 24 60.084 242

chr1 17302895 TGGAGAATTGGAAGCTTGATAGGA 24 59.525 GTTCTCTAGCAGTATCCACACACA 24 60.082 257

chr1 17304650 TGTTAGGGAAAATGACAAAGAGCC 24 59.477 AACCTTCTCTAACAGATGAGCTCA 24 59.225 117

chr1 17305868 CATGGATGGAAATGTGCACACTTA 24 59.842 CCCTCAGGTTGTGTCATATATATATCC 27 58.575 226

chr1 17309854 TGATCATTCCTTATCCATGTTGACC 25 58.936 ATAGGCTGGAGATTACAAGAGTTG 24 58.077 287

chr1 17314850 AGGAAAGCACTCAATGAAGAACTC 24 59.241 GCGCTAATACCAATTAATCACACG 24 58.863 253

chr1 17324004 CGAGAAGTGAGAAGTGAGAAGTGA 24 60.024 CCTATTGCCGGTTTCCTAGATCTT 24 60.202 105

chr1 17324597 CTTTTGAAAACCATGCCACCTTTG 24 59.965 TGGTTGTACTAGGAATGGTAGCAA 24 59.467 116

chr1 17325612 AGTAGAAGGTAAGAGCATTGGAACA 25 59.752 ATCCCTCTCGATTGTTCATCAGTT 24 59.837 167

chr1 17326891 CTGAGGTTGGGAGAGGAAAACTAA 24 59.958 TCACACTACTCCTTACTCTTCATCA 25 58.754 237

chr1 17336275 TGTGTGGTAGAAGAAATAAGGAGCA 25 59.987 GGCTTATCTCCCTCCGTTATCATT 24 60.02 297

chr1 17336507 CCTCATTTCGTCTGCTTCTTGTTT 24 60.024 TTTCGATCACATACTCAAAGGGGT 24 60.02 261

chr1 17339013 ACAAAGAAACGGTAGGAGAGTGTT 24 60.142 CACTGTCATTGTCTATTTCCTTTCC 25 58.154 262

chr1 17339998 CCATATCGAATGTGTCTAGTGGGT 24 59.961 TCCCTTTGGTTTTGCTAAGTTTGC 24 60.682 232

chr1 17358212 AGCTTAACTCTTCTTCGTAGTCCT 24 58.988 AGGAGCGACTAGATTAATGGTGTG 24 60.202 285

chr1 17361278 CCGTTGAACTGTTACATTGACGAT 24 59.848 GGTAGGTAAACGGAGAGAGACAAA 24 59.781 237

chr1 17368607 GTATCCTTACTTGGCAAAAGCTCC 24 59.903 TCTCCCTTTCCAAAACCCCATATT 24 59.953 244

chr1 17373935 GTTGTTGTTGTTATTCTCCCTGGG 24 60.022 GCCCCACTTTTCTTTCTCTCTCTA 24 60.02 226

chr1 17391702 GATGTTGGTGTTCCCTAATGTGTG 24 60.083 AGGTTTGACGTCCATAACTAGAGC 24 60.382 276

chr1 17391856 CCTAAGTGATCTCCGTCCTCTCTA 24 60.202 ACTAAAGGCGAGACTTAACACACT 24 59.961 140

chr1 17405336 TCCTTCACGATAAATTGCAGGATTG 25 59.934 ACTCTAAACGATCGTCTTGCAGAA 24 60.321 227

chr1 17408008 CGGTGTCGTATATCCCATCTGAAT 24 60.082 AGATGGATTCTTAGGGATGATCGT 24 58.91 275

chr1 17409959 TTGGCCAAGTGATGAGAATAGCT 23 60.054 GGTTAGAGTGTAAGTGGTGAAGGT 24 59.96 299

chr1 17417849 CACTTTCCCACGTCTGATTGATC 23 59.628 ACAACTTGGTCTGGTAGAAATGGA 24 59.896 273

chr1 17419141 TCTCAGTAGACCCACATTCCATTG 24 60.081 TCTCCTGAGTACCATAACATAGAGA 25 57.85 246

chr1 17420449 TCCAATAGATCAGTTGCCATCACT 24 59.835 CACAAAACACATTCACACTTTGGG 24 59.67 94

chr1 17421787 TGTGAGAGGAGGGATAAATATTTGG 25 58.158 CAGCCACTACAGAATTTAACGACC 24 59.907 278

chr1 17422500 CGTCTTCCTCTTCTTTCCTTAGCT 24 60.082 AGAATCCCATCCAACATCTTCAGA 24 59.524 201

chr1 17422673 CCAAGTGGTATTTACTCCCGATCT 24 59.899 GAGTAGCTTTCTTCTTCCTCCGAA 24 60.082 186

chr1 17423403 CATGCAAAAGCAGAGGTATGGAAT 24 59.901 AGAGCCAGTTCTGAAATTCGAAGA 24 60.263 298

chr1 17426970 AAAGCCTTCCTATTTGTCAGTCCT 24 59.957 TGGCAAGACTAACCACCATTATCA 24 60.019 289

chr1 17442457 GTTCGAATAATGTGGCAGAGTACC 24 59.73 CGTCAAATCTGTCCATCAATCTCC 24 59.728 195

chr1 17445620 GATGAGGGAGGAATGACACTACTC 24 59.961 TTGGAGATTATGTGTGAGGCGTAA 24 60.081 175

chr1 17449711 CCACCTAAAGCCCACAAATTTTCT 24 59.96 TGCAAGTCTATTTGAGCTTCAGTC 24 59.307 239

chr1 17452243 CCCGGGTGTATATTATTAGGAGGC 24 60.142 GCCCATTTCTCTCGTCCTCTTTAA 24 60.623 241

chr1 17454857 AATAGGCTCAATACCACAAATGCG 24 59.963 ACACCCCATCTTAATCCATATGCT 24 59.645 245

chr1 17456419 TTTTCTACATGGCTGCCTTTAACC 24 59.78 ACGTGGGAAGTAGAAGAAGAAGTA 24 58.925 273

chr1 17457088 TCAAAGCTTCCTCACGAACTATCA 24 60.021 AGGAATGTCAAGGAAAACCAAAGC 24 60.202 208

chr1 17458259 TAGCCGTCACACTGTTTTCTATGA 24 60.022 CTCTTCTTCCTTCTCCTTTCTCCT 24 59.223 232

chr1 17461959 TTCTAAACTTGAACCTTGCCGTTG 24 60.202 AATAGTCAAGTTCGGGAAAGAGGG 24 60.324 196

chr1 17463631 CGTAAACTCGAAAATGGGCATTCC 24 60.964 CGGAGGGTAAAATATTGACGAACG 24 60.026 295

chr1 17469025 ATTCAAAGCAATGGTGGAAGGAAG 24 60.021 ATCTGGTCCCGATGGTTTTACAA 23 59.99 285

chr1 17470441 GAAAACATAGGGTGCAAGACGAAA 24 60.024 AGCAAGACTTAAGGACCTCAAGAT 24 59.466 248

chr1 17471180 ATAGATTCGTCCTTGTTGCTTAGG 24 58.639 CGTTGAAAGGAGCGTATTGAACAT 24 60.143 140

chr1 17478224 GACAATTCGACTTTAGAAGCACACA 25 60.05 TCGAGTTGCACACCTTATTGTCTA 24 60.022 156

chr1 17479670 GCAACATAACAACATGACTCCAATG 25 59.426 GGAAAGTGTAATGCTGACAAACCT 24 59.722 269

chr1 17488942 GTGGCAAGCTTAATGAGATCTTTCA 25 59.876 GCTCAAGACGTGGTAAAACTCAAT 24 59.787 285

chr1 17493703 GAACCAAATCAGCTAGTGTTAGGC 24 59.905 TTCTTCTTCTTCCTCATGCCCTAC 24 60.081 251

chr1 17501819 TTTGTCTCCCTATCCACTGTTGAG 24 60.02 AGCATATCGAACATGAGTGAGTGA 24 59.901 261

chr1 17504818 ACTGGTCTTGACATTGCAGACTAT 24 60.02 GTAACTCAGTTGGACAAATACGGC 24 60.143 291

chr1 17507855 AAACAAGGGAGAACGATGATTTGG 24 59.782 GTGAAACGAGGATGGAAGTAAACT 24 59.005 297

chr1 17508717 TCTTTATCACAGACGGAGACTACA 24 58.749 ACGGATGGACAAAGAAATGAGAGA 24 60.02 250

chr1 17509059 ATGTGAGTTGTGTGGTCTAAGGAA 24 59.897 TCAAGTTGTGTATCCTCGTGTACA 24 59.721 276

chr1 17511001 TTGGAAGATCTCTCCCTGAATCAC 24 59.837 GTGTAACTTGTTGCTGCTACTTGT 24 59.965 161

chr1 17511943 TCGTATATCAGTGTTGTGGCATACT 25 59.931 ACTTGATATGCTTGAAGTCCTACGT 25 60.105 265

chr1 17521622 AGAGCCAAAACCCTAGACTTACTG 24 60.02 GAAGAAGGTTGCGGCAGGAAGATT 24 63.668 128

chr1 17528726 AAGAATCCACCCAACTTAACAGGT 24 60.141 GATTGTACACGCCTTTGACATTCT 24 59.846 210

chr1 17537311 TATTGCCCTCACGATGTCACAAG 23 60.927 ATATTACACGTGTTGGATGCCA 22 58.12 287

chr1 17542264 TATCCGGAGTCAACTAGAACAAGC 24 60.142 CTTCATTGGGTGGATAAGTGCTTG 24 60.142 235

chr1 17554956 GACGCGGTATGACTCTAAGTATCT 24 59.548 AAGTAAGGAAGAAGTGAAGGGCTT 24 59.895 291

chr1 17560041 TCTTCTCACAACCTACCATCTGAA 24 59.162 CTTACGACGAGTTTGGTGAGCAAG 24 62.117 246

chr1 17561453 GGTTTGTTCCATTCAACGTGACTA 24 59.726 CTGCCCTCACTGTAAAACAATTCA 24 59.722 295

chr1 17561667 TGAATTGTTTTACAGTGAGGGCAG 24 59.722 TAATGACTAAAGCCCGATTCACCA 24 60.081 300

chr1 17563130 CGAGATTATTAGATCAGGCACGGT 24 60.321 TCAAAAGGACCTAGTGTGACAA 22 57.236 269

chr1 17564850 GAGACAAATTAAACATGAGGGGCA 24 59.538 AGAACACGTATTTGACATCGGACT 24 60.322 206

chr1 17644062 AAAATGGTAGGGGTGGCAGC 20 60.615 GTCATTGCTGTTCTTATGGTTGTCA 25 60.049 233

chr1 17685635 ATTAATTTAGGCCCCACTTCGTTC 24 59.357 AATTCTAGAGATTGAACCGCATCG 24 59.253 296

chr1 17689440 CGACCCAACTTTTCAAACTAAGCT 24 59.964 AGAAGAAGATGTGATCGCCATAGG 24 60.262 296

chr1 17689855 ATTGAGAGGTACCATGCGTCATAA 24 59.899 AGCATAGATTGAGGGGAAACTGTT 24 60.018 249

chr1 17690018 CACTTGCAAGACACACTACCCTAT 24 60.564 TGAATTGACTGAACTGAGGTGAGT 24 59.898 198

chr1 17697044 CTTTGGGCAATTCTCATCGTACAC 24 60.437 ACTCAAGAACCAAAGAGCAGTACA 24 60.142 295

chr1 17698945 TACAAGACCACAAGATTTAGGGCA 24 59.958 TCCATTGATCGCCGTTAATAAAGTG 25 59.993 140

chr1 17701897 CCCATGTGCATTTTCCCTCTTAAT 24 59.593 ACCTATCCAACAAGTTAAGTGCCA 24 60.203 300

chr1 17704253 ATTGCTGTTGTTGGTGTAGAAAGG 24 59.962 TCGTTAGGCACCACAATATAGACA 24 59.597 249

chr1 17713325 TATTGCGGTTGTTAGTTGTTGTGC 24 60.79 GCAACACAACGAATAATTCCAAGC 24 59.91 299

chr1 17928666 TGTAGATTCCTTCAAACACCCCTT 24 59.894 TCAACACAATTTCATAGGGGCAAC 24 60.022 293

chr1 17947501 GTTTCCTTTCTTCCTTCCTCTCCA 24 60.202 TTTCCGTAGTCTTCTCTTTGGCAT 24 60.263 294

chr1 18169530 TTATAACCTTGCCAAACCGACCTA 24 60.02 GAAGCTTCTAGAACACCCGAGAC 23 60.674 290

chr1 18172293 GGCAATAGTGTCACAAGAGGGT 22 60.29 TCATCACTCATTCACTCACTCACT 24 59.474 207

chr1 18174193 ACCATCGTTTGCATGTATTCTG 22 57.379 CCACAACTTCGTCTACTGTTTTCT 24 59.191 300

chr1 18180242 TCGTGTAAGAGGTTAAACAGTCGT 24 59.963 TTTAAAACCGTCAAGAGAGTCGTC 24 59.258 288

chr1 18189481 CGGTTAGCGGTTTGGGTTCC 20 61.58 GACGTATGGCCAAAATGTGTACAA 24 60.083 294

chr1 18204946 CCCTCAATCTCAACTTCAATGGTG 24 59.843 GAGGTTGGGAGAGAGTGTGTTTAT 24 60.02 261

chr1 18205589 GAATAACAACCTTGGCGATGAACA 24 60.083 TGTTTGGTCGGCTTAGGAGATATT 24 59.837 264

chr1 18208540 CCTATAATTTCGCCAGCTCCTACT 24 60.021 TGTGTAAGACAAACTAGACGATCGT 25 60.049 300

chr1 18213249 GAATTTGGGGAAATGGAAGGTGAG 24 60.081 ATTTACTTTCATCCAACCCTCCCA 24 59.955 225

chr1 18253066 AGAGACGATTTAACCCTACAAGTGA 25 59.521 AATACGACTGAGGTTGAAGCATCT 24 60.081 298

chr1 18264753 AAAACACTGGAGGCTAGGGATAAC 24 60.324 TGCTTGTTTATCTCTGGTCCTCAA 24 59.958 105

chr1 18273594 CTTTGCCTACTTCTTACCTACGGA 24 59.841 TTAGATTACAGCTCAAGTTCGTGC 24 59.374 296

chr1 18279676 AATGAGTTGGGTTGGAAGGAGAAT 24 60.203 ACACCAAATATAGCAAGACCCACT 24 60.018 285

chr1 18292172 CCGTCAGAGATCTTCCTATCCTCT 24 60.507 TCTACGAAGGAAAGAGAGCTACAA 24 58.992 189

chr1 18296430 TGAGAGATCATAAACCACGAAGCA 24 60.082 TGCCCGTTTATTGGATAATGGATC 24 59.235 242

chr1 18304205 AATAACAACAAGATGGACTGCAGC 24 60.083 ACATCCCAAAGGCCTTCTATAGAA 24 59.273 270

chr1 18306033 GATTGCTGCAGTGTTGAATTTCAG 24 59.615 TCTTGAAGCCATATTAGCACCTCA 24 59.836 293

chr1 18307381 ATTTCCACCAACATCTTGCGATAC 24 59.904 AAAAGCAAGAAAGGGAAAGAGTCG 24 59.963 272

chr1 18351505 CAAGAAGAAGGAAGGAAGGAAGGA 24 59.958 AAAGTGACAAGAAGGGAAGAGGAA 24 59.834 244

chr1 18362469 CTCAAAGAGTATTGCAAGAACGCT 24 59.847 AATCAATGGCTTTTGGTCTTCTGG 24 60.021 240

chr1 18363780 GTCAGTCGGTCAGAGTATATGCAT 24 60.023 AGTTAAGGAGGAAAGAGTGCATGA 24 59.713 291

chr1 18364650 CTGAGTTGATATTTCCAAGCTACGT 25 59.187 GACCCATAAACTATAGGCTCGTCA 24 59.72 180

chr1 18367480 CAACAACTGCGAACCTTGAAAA 22 58.493 TCGAGATCACAATAGATAGGTCATC 25 57.616 288

chr1 18369185 TAACCTCAGCAACACATCAACAAC 24 59.964 AGTCCAACTTTCTTCATCCCCTAT 24 59.209 290

chr1 18375489 CGTAAGCCCATTTCATCCTTCATT 24 59.661 TGTTAATTGTGTGTTGGGAGTTCG 24 59.964 212

chr1 18377884 GCACGTGGTCATCATTTATCTGAG 24 60.025 GTGTAGGAAGAAAGGCGTTTAACA 24 59.487 256

chr1 18382876 GAGACTGCTAGGAGTGGGTTTAAA 24 60.02 CGCTTGTTAAAACCGAATCAAACC 24 59.853 191

chr1 18392822 TCCTATCCAACTATCACCAGGAGT 24 60.079 GATCAGTATCGCATTGCATTGACA 24 60.025 277

chr1 18398948 GTTGCATGTTATAGTTTGTGGGCA 24 60.322 CATGTAGGTGCAGTTGTTGATGAA 24 59.784 288

chr1 18481277 ATGACTTGTAACTATGATCCGCCT 24 59.655 GAGTTTGGAAGAGTGAAGGTGTGA 24 60.683 289

chr1 18593308 GTTAGTTGGTCTGGGCTTTTCTTC 24 60.023 TCATTGGCCGTAATTTCAAGACAG 24 59.844 138

chr1 18671258 ACCTGTTCTGGATTGGATTTTGAG 24 59.231 GGTTTGGGTTACTATATTGAGGCC 24 59.174 153

chr1 18793596 GTGGCCATAAAAGAAAGGGGAAAA 24 59.959 GGAGAGAGGGGCCACATTAAAATA 24 60.141 282

chr1 19235267 TTTATAGCCGATTCGATGGACACT 24 59.961 AGAAGCAGGAGGAGGAGTTAGTTA 24 60.264 219

chr1 19238785 TATTCCTTTCAATGGGCAGCATTC 24 59.9 TTGGGTTTGTGATTGCTGGAATAC 24 60.022 236

chr1 19297282 CACCTCTTTTCTTTGATTGGGCTT 24 59.96 TCTCAATTGTCCCCTCCAAGATTT 24 59.955 248

chr1 19298372 TCCAAATGAACAGCTCTTGATTCTG 25 59.817 ACGACCCAATAAGAAGATCAGTTTG 25 59.356 204

chr1 19309418 CCAACACAAAGCCTAATCCATCAT 24 59.597 TTCGTGCTCTAATCTTCCCATGTT 24 60.323 297

chr1 19311955 TCACAAAGAGTAATGGTGGCAATG 24 59.782 AGCAGAGATTGATGGGTCTTGAAT 24 60.08 280

chr1 19333407 AAGTCAACATAACCCCTCCGTAAA 24 59.958 AAATTATAGCCATGTCGTCCCTGA 24 59.897 227

chr1 19337987 GAATGAGAAACAGGGAACGTTACC 24 59.846 CTCGGTCCGACATCCCTATATTTT 24 60.021 259

chr1 19345490 GTGTGTAAGGGTATCTCTGGTTGT 24 60.02 AGACCATTCCAAGCAACTTTCATG 24 60.022 232

chr1 19347605 GTCGGGGAAGGCTTTAAATCTTTT 24 59.78 ATCGAGTGGAAGAGAGAGAGAAGA 24 60.081 272

chr1 19360175 CCATTTTGTTGCTGTCATCGTCG 23 61.206 TCTACATAAATTCGAAGGGCAGGT 24 59.837 275

chr1 19360328 ACCTGCCCTTCGAATTTATGTAGA 24 59.837 AGGATAGTCATTGTTGTTCCCCTC 24 60.08 294

chr1 19362417 TCCAGTAGTAATGAATGGGCACAA 24 60.019 GAAGAAAATGCTCTGCTAGTCCAA 24 59.303 159

chr1 19365081 TGCAGTTCAGGTGAGATCTACAAA 24 59.96 CATCGAGTGTGGGGTTCTTTAAAT 24 59.3 221

chr1 19370348 AGACGGTAAAGTGGAGCAGTAAAT 24 60.02 TGCCTGTGGAAAAGTTAGAGTACT 24 59.653 224

chr1 19388999 GATGGTCTGCAGCCGTTTTC 20 59.831 ACGGTGTCTTCTTCTCTAACTCTT 24 59.172 290

chr1 19395251 AGGAATTATGGGCTCTTTCAACCT 24 60.017 GTGGAAGCAGAAGAGGTAAGAGAA 24 60.021 152

chr1 19398256 TCTTTTGAGGATATCGACCGCTC 23 60.243 TCTTAGTACAATCGGATGGAACTCC 25 59.931 168

chr1 19401523 TCATCTCAGAGGATAGGAAGGTCT 24 59.581 AAAAGCCCTATAGTAGCGGAATGA 24 59.653 231

chr1 19401842 TGAGAGAGAGATCGAGAAAGGAGA 24 59.837 GAATGAAGAGTGACGACGGAAGAT 24 60.676 102

chr1 19402628 ACGTTCCATTGTAGCTAGGTCATA 24 59.352 CCCATATGCATTCAAACATCAAGC 24 59.252 151

chr1 19402975 GGTGGATTAATACAGTGACATGCA 24 59.119 ACATGTGCGTTAGGAGTTTCTCTA 24 59.78 233

chr1 19406979 GCTCTTTTCTTTTCCCTTGTATTGG 25 58.841 TCACCTTGCTCTTTCTAGTCAACA 24 59.899 299

chr1 19424621 GGCAAAGGTTAGAGCTCAAGGTTA 24 60.806 GATAAAATCTCACTGCCTCCAAGC 24 59.964 174

chr1 19425061 TCCCAAAATACCAGTACGTTGACA 24 60.202 TAGTTTGCAGATTCAGAGACGTCA 24 60.022 126

chr1 19428305 TCGTGTCAAGTAAATGTTCGCT 22 58.614 TACAAAATCCGCTTCACTTGACAC 24 60.024 190

chr1 19431728 CTAGAACAAGGTCTTCAAATCGCC 24 59.906 AGCGAAAGTTAACCACGATGAATC 24 59.907 152

chr1 19435165 CCTATGCAGACTTTATGGCTTGGA 24 60.687 ATCTTCTTCCAGCCCATCTATGTC 24 59.959 235

chr1 19436366 TTACTGGTTGATGGTTCCACTCAT 24 59.957 CTGTGTTAAAATGGATTGAGGCTCA 25 59.816 245

chr1 19436747 ATGGGCTCAACGACAATACAAAAG 24 60.083 GGCTAATATCATTGAACTATCGAGG 25 57.237 292

chr1 19438665 ATGAAGCGTAGAAGAAATGGCATG 24 59.964 ATGCCCATTTCAACTTACAACTGG 24 60.021 133

chr1 19770428 GTATTTGATGCACTTTGGTTCCCA 24 60.022 TCCCAAGTCTTTCCTTCAGTTTGA 24 60.08 101

chr1 20039619 GAAAATGTCCACAGTCTAACAGGC 24 60.083 GCATTGATGTTAGCTGCTCAGAAT 24 59.964 229

chr1 20099283 CGGTAGTCAATTCAGTAATGGTTTG 25 58.457 GGGACCACTTAATTGAAATGACGA 24 59.302 126

chr1 20285975 AGTTACACAAATGGACTTGGAGGA 24 59.896 ACACACCCCACCAAATATCACTAT 24 59.77 272

chr1 20301598 AGGGTCCCTCATTTTGATTTCTCA 24 59.955 GCTTGCTTGATGATGAGGGTATTC 24 60.023 219

chr1 20307731 GAAATGGACGGTGGAGGATTTAAA 24 59.297 TTGTGGGATGCATGTGATTGATTC 24 60.142 221

chr1 20313052 TGCAACGTGAATAGGTGAGAGATA 24 59.599 TCATAACTTCTCCGTCTTCTTCCT 24 58.983 266

chr1 20316017 GGTGTTGCTTGGGGATATTGAAAG 24 60.382 GGGGTGTTTTAGTTGGATCGATGA 24 60.865 267

chr1 20397982 GATTGGGTGGAGAATTAAACATAGG 25 57.723 GATGTAAACCCACCACCCC 19 57.724 300

chr1 20402339 TACAGGAGAGGTGGTGTCATTTTC 24 60.263 TTTCTGAGCTTTGTAGGACATTGC 24 59.784 210

chr1 20402953 TCTTACTCTATGGTGGGTTATGCG 24 59.961 TATTGCTTCCGGGAGTAGTTTCTC 24 60.142 208

chr1 20414450 GAAGGCATCTCTCCCTCATTTTAT 24 58.371 TGGATCTCATGGAAATGGGGAATT 24 60.078 218

chr1 20421321 ATGTAGTATCAACACCAGCCACAT 24 60.08 TCGTTTAGATTTTGGTACGCGA 22 58.426 257

chr1 20431010 ATGAAAATCTCGGCCGAATCAATC 24 60.024 CGAGGAGGGTGTAGTATTGTATTA 24 57.111 144

chr1 20433302 CTTACCATAGAGTTTCCCTTTCATC 25 57.206 CCTCCAAAATCTAGAAGGTAAGGC 24 58.873 298

chr1 20443349 GTGATTTGAGGTGAAGTCGTGTAC 24 59.85 AAGAATGTGGGGCTCAATGTAGTA 24 59.773 109

chr1 20445477 CTTCATCCTCAGCGAACCTACTTC 24 60.972 CAAAAGGTCGATCGTTCCCTAGAA 24 60.62 179

chr1 20448347 GGTGTTACTTATCAGCGTGTCATG 24 59.967 GGTCTTGGCATCTTTTGAATGACA 24 60.022 284

chr1 20469872 GTATGGTTTTAAACAAGCTTCGCG 24 59.68 TGGAACCCGTCACAAGAATATCAT 24 60.081 170

chr1 20476175 CTCTCCTAATCTCGGGCATTACAA 24 59.961 ACTCAGATCATTTGAAGTCTCCCT 24 59.218 251

chr1 20591491 GGTAGCATTCCAACACTCATTCAC 24 60.143 AATTCTAGAGATTGAACCGCATCG 24 59.253 125

chr1 20610664 AGAAGAAAGTGTGGGTCTCATTGT 24 60.141 TAAGCGTTATTGTGTGAAGGTCCA 24 60.503 95

chr1 20618019 TTAAACACCAATACAGCCAGCTTG 24 60.022 GCCTGAACAGAACACATAACAACA 24 59.964 271

chr1 20622306 GGAATGGGTGTTAAAATTGGCATC 24 59.124 TTCCCTTCGTCATCTTCTTCCATT 24 60.019 275

chr1 20624003 TCGGGTGATTTGGCCATATACTAG 24 60.021 GGAGGAATCACATCTCTTATGCCT 24 59.959 295

chr1 20640871 CGAGCGAGTAGTACATGGTCTTTA 24 59.965 CAGCCAATCAAATGTCATTCGGTA 24 59.904 291

chr1 20645070 ACAGGAGGATGGCTTTCAAATTTG 24 60.021 CAAATGCATCCACTAACAATGTTGG 25 59.651 104

chr1 20649576 CCTTTACACACTAATAGCAACAAACCC 27 60.787 ACCAAGAGTTGCTGTAGTGAAATG 24 59.483 182

chr1 20669176 AGGATGCTGCATTCTCTTAGGTAT 24 59.403 GAAAGGGGTGTGAAATAGTTGCAA 24 59.961 298

chr1 20670125 GTTTGATAGAGAAGAGGGACACGT 24 60.082 CCAACATGTCAAATGGGTGTAA 22 57.137 267

chr1 20687495 GGTACGTGAGAAACTAGAGGTGAG 24 60.143 ATCACTTCAGATGTTGGAATGAGC 24 59.361 165

chr1 20701698 TTTATAACAGTGGACGTGCAATGG 24 59.845 ATTGATCTGCATCGCTTCTTCATG 24 60.024 197

chr1 20720633 GTAGTACATACGACTCACTTCATTC 25 57.104 AACCCCACTTGTGTTTTGGTATTC 24 59.9 288

chr1 20722169 AGGATGGAGTGAGGAATAGGAGAA 24 60.078 CCCCAAATAATCCTTCTCCCAAAC 24 59.594 256

chr1 20723700 AGTGGGATAATAAACGAGGGAAGG 24 59.897 AGAACACATGCAAAGGACAATGAC 24 60.262 295

chr1 20723933 TGCATGTGTTCTAAAGGACAATGG 24 59.782 CCTACGTTTTAAACCTTCCTAACATCC 27 60.148 283

chr1 20724131 GACAATGGACAAGAATCAACGGG 23 60.12 CCTTTTCACCCATCTACCCTTGTA 24 60.019 298

chr1 20726864 GATGCATGTGAAATGGGGATTTTG 24 59.427 CACCAAGGGAGAAAACAAAGCATA 24 59.719 279

chr1 20757689 CGGATTCATCGACACATATATCAAC 25 58.017 AGCTGTTTTCGTGGGTTAGAGTAA 24 60.202 300

chr1 20758141 GTGACGTACATCTTTGCCTTTTCT 24 59.787 TGTTAGAAAGAGAAAGCTTGGACC 24 58.936 294

chr1 20758330 CTCAAACTCAATGCCCAAGAAAGT 24 59.962 TGAGAGTTGTACGAACGAGAATGA 24 59.785 223

chr1 20766268 GCAAAGGGTAAGAATTGTTGGACA 24 59.961 AGCACCGTCTTCAGTAACATGTTA 24 60.262 182

chr1 20766642 GAAGAATCGTCGTTCCATCTCCTA 24 59.964 TCACAACTCTACTCATGGAAGACC 24 59.779 190

chr1 20767574 GCAACTTTCACTGGGATTCGATTA 24 59.605 GACGTGGTCCTCATTCCTTTAC 22 58.74 300

chr1 20768087 GGTTCTCCCGAAGTAAATTTCCTT 24 58.992 CAATTCAATCTCGGCGTTAGAACA 24 59.907 289

chr1 20771734 AGCTACCTATTGCCACTATGATCA 24 59.159 ATTGGAGGATAAGGGAAAGCTCTG 24 60.141 290

chr1 20779212 TCCAATCCAACTCAACTCTCTCAA 24 59.652 CCATTTCGTTCTCCATCTTCCATG 24 59.964 210

chr1 20780648 AGGAGGGTGATGTCTTTGTTTCTT 24 60.141 AGTTCGAGCAATGTCAACCTAAGT 24 60.503 181

chr1 20785324 GGATACCCTCATTCAAACCATTCT 24 58.55 CCTACCTCAAAACATGTCATAAGCC 25 59.933 291

chr1 20793515 CACGTCGATGTCAGTATAGGTCAA 24 60.202 TACAAGAAACCTGAGAATGGCTGA 24 59.958 293

chr1 20797695 CAACCCCACATGAGAAAGAAGTTG 24 60.262 TGTGTTGGGGTTGCAGTAGAAATA 24 60.446 220

chr1 20797814 CAACCCCACATGAGAAAGAAGTTG 24 60.262 GTGTATTGTGTGTATGGTCCAAAGT 25 59.528 300

chr1 20810909 GATTTGCAAATTCTGGGGATCCTT 24 59.837 TCACAATGACCTTCGAACCAAAAG 24 59.964 276

chr1 20814049 TTCACGAACTGGCCACTCTTATTA 24 60.021 TCAAAATGGTTGGGTTTCGTACAC 24 60.202 238

chr1 20817799 CCATCGAATTCCAATCAAGCATCA 24 59.963 GTGCGTTAATTTGATTCGTGCAGA 24 60.901 202

chr1 20831033 CCTAGTCTAAATTCACACACCTACAC 26 59.188 GTAAAGGCTTTGATGTATGTTGTGC 25 59.427 235

chr1 20832688 TGGCTAAGCACCTTTAAACTCTCA 24 60.202 GTTGGAGCTTTGAGACCCTAATTT 24 59.233 296

chr1 20841875 AAGCCCCAAATGAGATACTAGCTC 24 60.202 GTGCTTGAATGTCTGTTTCCACTT 24 60.202 159

chr1 20849267 TAATTAAGGGGAAGGGTACGATGG 24 59.651 TATACGACAAGGACCACACTATGT 24 59.048 282

chr1 20851153 TGGTCTAGCAAAATGAGTTCGTATC 25 59.189 ACATGCTAGAAATGGAGTCGATGT 24 60.142 289

chr1 20870520 GCAGCTCTATTAGGACATTGCATA 24 58.759 GCGCTTATAGTAATGGTAGGGAAT 24 58.268 300

chr1 20908284 GCTTCACCACAACATACTAAGATGG 25 59.935 GGGGACGCAAATGAGATAATAACA 24 59.18 242

chr1 20929463 CTACAGGAGCACGAAGGGTAATTA 24 59.901 TGTACCTTTTCCAAATCCCCAAGT 24 60.388 213

chr1 20929769 GAATTTGCGTTGAACAATGATGCC 24 60.435 AGAAGGATAATGGTGGAAGATGAGA 25 59.092 287

chr1 20933652 TGCGAAGACCTTTCAACAATAGAC 24 59.549 GACATCTATGCAATCTTTGGCCAT 24 59.72 251

chr1 20935219 GGTGAGTGCATGCTTGAGTTTAAT 24 60.083 TGAGTTCAGTAGCGTTTAATGTGG 24 59.311 194

chr1 20935605 ACACTGTGAAGATAGAGATTCGACT 25 59.35 TTCAATAGTGGAAGCATCGGATCA 24 60.142 182

chr1 20936821 CTCCTCTTTCTCCTTTGTCCATGA 24 60.02 TTCTAAGAAAAGTGGCTGTGCTTC 24 59.724 297

chr1 20937311 ACCATTGTGTTTGTTAGGCGTATG 24 60.083 AGTGGTCACATAAGATGATGCCTT 24 60.08 215

chr1 20938350 CCGTGGTCATTCAAGTAACAATAGA 25 59.127 ACCTGATTTCACTGCCTAATTCCT 24 60.018 252

chr1 20947894 AAAATCTCCCACACCTACATCTCC 24 60.08 TACATGTGGTCTAAAGGAGGAAGG 24 59.53 91

chr1 20949610 ATATAAAAGCCCTTACATGCAGCG 24 59.784 CGAAGATGAAGGTAGCCAACTAGA 24 59.902 264

chr1 20962554 ACCAGCTAGATGTTAAGAATGCCT 24 59.835 CTGACTATACCCTTGGGACTTGAC 24 60.142 197

chr1 20982988 CAATGCAAGTTCACCCTATCAACA 24 59.782 TGAATCGGCCAGTCTACCATATTT 24 59.897 298

chr1 20984799 CCCTTTGAAGAACCGGATTGAAAA 24 59.961 ATGAACCGGATGACATAGTACTCG 24 60.023 299

chr1 20987321 CGCTATCGTTGATAATAGGCAAGT 24 59.076 TCAGGTATGGTCAAAATTGCAC 22 57.218 197

chr1 20995281 CCCCAAAGGTAGAAACACAATACA 24 58.926 CGAACTAAATTTAAGACTCTGCACG 25 58.766 247

chr1 21004108 GAATTTGAAGTCAGCACAACCACT 24 60.202 CACCTACCCCACTAAGAATGTCAA 24 60.02 292

chr1 21021319 AGCCCATCCTCTTTGTATCACTTT 24 60.018 TCTTTCCACGTAATCCTTCTCACT 24 59.473 262

chr1 21071084 AGCATAACTCCAGTAATCGTCCTT 24 59.594 ACACCATTTATTGTTTCGAGCGAG 24 60.143 245

chr1 21071280 CTCGCTCGAAACAATAAATGGTGT 24 60.143 TCCCGTGGACAGTCATTTAAGATT 24 60.02 144

chr1 21084915 TCGTACCTGCATAATAGTCATAGCA 25 59.524 TGCATAAACTGATAGAGAGACCCA 24 59.038 225

chr1 21138357 TGGTTAACTAATGGGGTCGATTCT 24 59.528 CGGTGAGGCAATAATGAACGTAAA 24 59.906 294

chr1 21228034 TGAGTTCTGTTGTGTTGTTGCTTG 24 60.616 CTCTCTCTCTCTCTCTCTGCTCTT 24 60.142 277

chr1 21235968 GCATTCCACAACCATTTCAGATGA 24 60.082 CTAATGTGTGGTCTTGACAGTTGT 24 59.184 259

chr1 21237336 CGTAGCCAACCCGTTATTCTTAAG 24 59.731 TAACATTTTCCCAAACCCTCATGC 24 60.021 148

chr1 21237947 ACGGACATAAATAGGTGGTGATCC 24 60.202 GCTATGATGCATGTGATGTGACTG 24 60.32 254

chr1 21238360 TGAATCCAACGCAACCTTAAATGG 24 60.322 CTGAAGACACTCGTTGATCAATGG 24 59.907 284

chr1 21245221 CCAACAAAGGTAGATGTCATCACT 24 58.751 GCAAGCTTCAAGTACTCATAGTGG 24 59.669 279

chr1 21246069 ATTGTTGTTAGTTCGCTGTTGACG 24 60.552 TATTCTCTTCCTTCTGCCCTTTGG 24 60.325 257

chr1 21246327 CCAAAGGGCAGAAGGAAGAGAATA 24 60.325 TGATATGCATTGAATTGGAGCTCC 24 59.478 185

chr1 21254337 CCCATTGGTCTCTCATTAGCGATA 24 60.021 GACCAAGATCAATAGCCGGAAAAG 24 59.964 209

chr1 21256658 ATATACCCATAGTGACCGATGTGC 24 60.082 AAGCAAATGACCACACAAACAGAG 24 60.441 95

chr1 21259479 GCCTTTGCGTCATTTGGAG 19 57.593 TTAAAACGCTGTTTACCCTTGCTC 24 60.261 291

chr1 21260460 ACTAAGTACATTGGACGAGTGAGG 24 59.842 AACTACACATCTGTTGGACCTCAA 24 59.897 296

chr1 21262336 GAACATGGAAAAGAGCTCAGAACC 24 60.083 TTCTGTGAGTACTGTGGGAAAACT 24 59.837 106

chr1 21264134 GGTTTTGCTGATGATCATGGAGAG 24 59.964 TTAAGCCTAACCGTTATCTGCTGT 24 60.081 245

chr1 21264438 ACAGCAGATAACGGTTAGGCTTAA 24 60.081 GAGGGAGATGAGGTTTAGACGAAC 24 60.44 245

chr1 21267301 TTGAAATCTCCTCCCATAAGCCAA 24 60.018 AGATGATCTTAGATTCAGTGTGGCT 25 59.634 215

chr1 21279956 CATTGTAGTTATGGTTAAGCTCGCA 25 59.707 ACGCGATAAACACCATTACTACCT 24 60.142 215

chr1 21281868 TTGTAGGTGTCTGACTTGGTTGTT 24 60.324 AGAAGGTCAAAGGGATGCCTATTT 24 60.017 259

chr1 21282981 GGCTTTGGTGTTGTTATATAGGGC 24 59.962 TTGTGCTGGGAGATACAAGATCAA 24 60.019 282

chr1 21284338 AGAACACGTCCTCCTCACATTATC 24 60.142 TCCATGGATAAGCAACATACCCAA 24 60.08 262

chr1 21286888 CTTGGTAATGATGCATGCACTTGA 24 60.142 ACCAACTCATCCAAGTGTATCCTT 24 59.71 225

chr1 21287539 TGAGTAGCTAAACTTGGTTGGAAG 24 58.694 CACCCACATGCATTCTTTCTTTCT 24 60.022 165

chr1 21292865 AAAGGCAAAGGGAAAAGATGAAGG 24 59.959 TGAACCTCTTAGCCACACAATACA 24 59.959 221

chr1 21294957 TTCGTTATGGTAGTTGGATCTGGA 24 59.287 ACATTTGTCCGTCTATGAAGCCTA 24 59.838 288

chr1 21299204 ACCCTACCCTATTTCTACCTGAGT 24 59.765 CCCGGCCCTCTCTCTCTTATATAT 24 60.387 266

chr1 21299395 TATTTGCCACGTCACTCCTTTCTA 24 60.021 GGTAGGGTATATGATTTAACGAAGGTG 27 59.398 243

chr1 21301609 GATTGCACGTAACTCATTCCCATG 24 60.496 AGGAATCATACACATCTACTCTGGA 25 58.623 140

chr1 21302676 ACTCATCTTGTAGGACCCTTTTGT 24 59.649 ATACAAATGCAAGAGAACACACGG 24 60.083 265

chr1 21302977 CCGTGTGTTCTCTTGCATTTGTAT 24 60.083 TTCACCCCTACCTTTCAAAACTTG 24 59.353 152

chr1 21303099 CAAGTTTTGAAAGGTAGGGGTGAA 24 59.353 GCTTTTCAATCTTATGGCTAGTCGA 25 59.476 269

chr1 21304747 GTTTTGTGTTTGGGGTTTTCTTCC 24 59.606 AAATCCTTTTACGAACACACAGCC 24 60.261 264

chr1 21306577 AGTACAACCTAAAATGCAAACGGG 24 60.023 CCTTACTTTCAATACTCGGGAGCT 24 60.142 281

chr1 21363240 AATAGGCAATGTCTGGTCAAGTCA 24 60.263 CGTCTCTCAAAACTTTCCTTCAGG 24 59.788 229

chr1 21385981 CAATCAAAATAGTCAAATGAGCGCC 25 59.769 AGGCATGACAAAATAGGTGATACAC 25 59.41 297

chr1 21386318 TTTCTCAACCATCTCCTGTTTCCT 24 59.895 TGAGAGATTGCAGAAGAATGTGGA 24 60.02 97

chr1 21395238 TGTTGACGAAGGATGGATAGTTGT 24 60.021 ACATGACATGCAGTGACTTCTAGT 24 60.021 273

chr1 21399057 TGCACTCACTGATTCTACTTCCTC 24 60.082 GATTTGGCGGATCGTTTTCTACAT 24 59.965 118

chr1 21405085 GGTAACTTGAATCGTGCTTTCA 22 57.258 CGATGAGAACAAGAAGAGCAAACA 24 59.788 268

chr1 21410729 GGGATCACAAGACTATTATGGTGC 24 59.243 CTCTCACAATACCTTTGCCTGAAA 24 59.238 153

chr1 21419012 TACTTGCTTAGGTAGTAGGGACGA 24 60.08 AGATTAGATGAGATTAGGAGGGTGG 25 58.978 170

chr1 21420924 ATGTGTTTGGAGTATTGTGTGCAG 24 60.023 GGTTGATATTAGGGTTTCGACATTG 25 58.275 274

chr1 21422388 CAAGTGCAATGGGAGTTTCAAATC 24 59.313 GGCTTACCCCTAGAAACTGTACAA 24 60.02 243

chr1 21426165 GGGATGAAGGCGTCGAGTTTT 21 60.944 TCTAGCCACAGTCGTCGAATAAAT 24 59.903 279

chr1 21426487 TTATTCGACGACTGTGGCTAGATT 24 59.903 AAAACTCTGCCAAGCCTTAATGC 23 60.306 222

chr1 21459865 CTTTGTTGGTTGTTCATAGGGCTT 24 59.961 AGTTGACGAGAAGAGAGTGATCAA 24 59.479 248

chr1 21461487 ACTTTTCTTCATCGAACGGGTAAC 24 59.55 CTGTTACGATCCAAATACAGTGCG 24 60.26 267

chr1 21469144 TGGGTTGTTGAAGATAGAGTTCGT 24 59.96 TAGTTCAAAGGACGTTAAACCCCT 24 59.897 183

chr1 21479890 CCACTCCACATTCTATTATGATTGG 25 57.555 TGAATTTGTGACCTTGGCTCTAAG 24 59.238 279

chr1 21480581 TCCCACTGCTCCATTCTTTATTCA 24 60.019 CCAGGTTCAGTTGAGAGATTGAGA 24 60.021 231

chr1 21508246 TAGTGTCACTGTTGCAAGAGAAGA 24 59.901 TGGGTAGGGAATTATATGGGAGGT 24 60.14 218

chr1 21508701 AAGGTTGACATCAGATCCCGATTT 24 60.325 TAGCTTCGTGTTTTGTTAACCTCG 24 59.79 213

chr1 21514647 AGTTTGGCTTGTTGTTCTTGACC 23 60.119 AGGTAGTACTTCCCATAGTGTCTT 24 58.16 159

chr1 21516450 CCATAATGTGAGTTTGGTTGGGTC 24 60.082 ATTGGTGTTAGTTGATGATCCCGA 24 60.081 201

chr1 21520863 CTGGCCTCAAATCAGTTCAAGTTT 24 59.962 CTATTCAAGGGGAGGATTGTTGGA 24 60.08 287

chr1 21522609 GCATGGGGATTAGATATTGCTTAGC 25 59.933 GTGAGAAATCAACTTACCCACCTT 24 58.929 248

chr1 21524055 TCCGTAACTTCCTCTCAAATCCTC 24 59.84 CTGCAGATTGTGAGAAATGGGTTT 24 60.022 149

chr1 21538753 CCCTCTCGATTTCTCAATAGCCAT 24 60.263 TGACTCATCTTGTATCTAAGCTCGG 25 59.991 289

chr1 21729058 TCAACTTGGGATGCAAAGAAGAAG 24 59.721 GACTTCCAAATCTCAACTTTATGCC 25 58.674 296

chr1 21881318 CCTTGACCCGAAATCAGAGATCTT 24 60.384 TTGATGAAGGACGATATTTAGGGG 24 58.135 238

chr1 22060927 TGGCCACAATTCTAACAGTATCCA 24 60.019 TGCCAGGCTAAAGAGAAAGGTTAT 24 60.018 247

chr1 22199352 GAGGGTTAAGGTGGAGGGAATTAA 24 59.771 GATGCCGTATCCACTTAACCATTC 24 59.787 242

chr1 22201028 TTGGGATGATTCAGCAATGTTCAG 24 59.841 GATCCCAATCCTTTTCAATGGCAG 24 60.442 273

chr1 22202167 GTCGGTTTTGTTTCATTTTGGTCC 24 59.731 CGGTAGGTGTCCAACAATGATCTA 24 60.142 256

chr1 22204953 GCTCAACTAATCCTTGAACTTGAAC 25 58.625 TGGACTCTGTGTTTTCGACTGTAA 24 60.142 300

chr1 22205415 CACCATCATGTCTATCTAGCTCGT 24 59.783 CTACTAAAACGAGCAAAACCTCTGT 25 59.534 297

chr1 22207373 GGGCTCGAATTTTCTTTTGCTA 22 57.565 AATTCGCAACAGGTAAGAACAAGG 24 60.023 272

chr1 22209155 TCTAAGTAAGAAGGGAGGTTGGGA 24 60.203 GCAAGTTATTCAAACACAAAGCCC 24 59.789 216

chr1 22211950 AGAAACCTCTTACCGTCACTTTCA 24 59.9 GTTTTCTCACTACAATTGGGCTCC 24 60.083 207

chr1 22212088 AAGAACAAGTGAGAGAAGTGGAGC 24 60.742 TCTCTCTCTCTCTCTCTACTCCCT 24 60.079 294

chr1 22212336 GAAAACAATGTGTCCCTCATACCC 24 59.842 CATTGCCTTCTCTCTCTCTCTCTC 24 59.963 257

chr1 22217465 AGAGTGTAATGGTTTGGTTTGTCC 24 59.417 CTTGTTGAAGCGGTATGTCCTTTT 24 60.023 262

chr1 22224070 TGATTCCACCGCAAAATATTACTCC 25 59.702 GGTAACCATGACAAAACTACTCTTC 25 57.87 261

chr1 22224246 GAAGAGTAGTTTTGTCATGGTTACC 25 57.87 GCATGACCACCAAGAAATACCAAA 24 60.022 178

chr1 22225083 TCTCTCCCATCTCACCTTAGTCTT 24 60.017 TTTTGGGTTATATGGGTTGGCAAC 24 60.02 182

chr1 22286319 CAGACTTTCCAACATGCTTATCGG 24 60.202 GAATTCTCTACAGCAATCTTCGGC 24 60.025 163

chr1 22286603 AGTACTACGAGTGAATGGCCAAAT 24 60.081 CATTTCCCCTTCTACCCTTCTCAA 24 60.018 174

chr1 22291682 AAGTTGAACGTTTTCCTATGCCTG 24 60.023 AACTGAAGAAACTGGGTGGAAGAT 24 60.141 274

chr1 22293306 TAATCGACTTTCACTCCCTTGGTT 24 59.959 TTCTTCTCTGCATCTGATTTCCCT 24 59.773 292

chr1 22298117 GGAAGGGAAATTGCATCAGATGAC 24 60.202 CGAGTATGAAATTTGAGAGAGAGAACC 27 59.778 280

chr1 22299481 TGTGATCTTAGAAAGGTGGGTTCA 24 59.65 ATGGACTTCTCTATCATTGCTGCT 24 59.897 232

chr1 22300507 ACTTTCAATGGCAGTCTAGTCCA 23 59.672 GATGGGAGGCGGTGGAAAA 19 60.001 290

chr1 22304948 CCCTCTCAAACATCTCCTTAGTGT 24 59.776 ATTGAAAATGTCCGTTCCTCCATG 24 59.842 86

chr1 22344268 AAAGGAGAGAGAGAGAGAGAGAGG 24 59.835 GTGTAAGGCGAAGTTAAAAGTGTCT 25 59.764 262

chr1 22404939 CCATCACCCTTCATTCCAGTATGT 24 60.386 CATTCACCAACAACATTCATCCAC 24 59.075 247

chr1 22408005 AGCCACTCCCATGCACTTTT 20 60.179 CCGATGAAGAGAGATGGGAATTGT 24 60.444 296

chr1 22440664 AGGGAGAGTTTTGGAAGTAAGTGT 24 59.587 CTCTCTCTTCGAAAACTCTTCCCA 24 60.022 101

chr1 22444150 AACAACACACACACACACACTATC 24 59.906 AAGGAATTGAGATTGAGGCAGAGT 24 60.019 172

chr1 22446828 CAAAGCCAAGGAAGTTTCGAGAG 23 60.059 AGTAAAGAATGAGAGTCGTGGGTG 24 60.322 130

chr1 22446984 TTATGGACTAACACACACTCCCAC 24 60.263 ACATAAGAACCCAACCTCCAGTAG 24 59.775 235

chr1 22450328 CTTTCATGCAGTGGACCCAAAATA 24 59.78 CCCAACCCACATTTTAACCTAACC 24 60.021 296

chr1 22450489 GGGTTGAGTTAGGTTAGGTTAGGT 24 59.466 TTCGGGTTGGGTTGGATTGATC 22 60.882 211

chr1 22455832 CCCTTTTCCTCCGAATGTTACAAT 24 59.295 GATCATTGAAGGCATGTTCGAGAG 24 60.025 260

chr1 22459210 CATTTCTTTGACTGCCCGTATCAA 24 59.844 TCTCCATTCCATCTTTCTGACTGT 24 59.466 265

chr1 22461156 GTTGTTGATCATTGTAGCCATCGT 24 59.905 CGAAATGATGGAAACTTTGAACTGG 25 59.369 126

chr1 22462408 GGATACCTGTTGTCTTCTTCCTCC 24 60.383 TGACCTAGATACTCTCCTACGCTT 24 59.897 210

chr1 22464929 GGAGATCAGACCCACCATTATTGA 24 59.897 TTTATTTGATTTGACACAGGGGCG 24 60.322 274

chr1 22465237 GGTTTTCTAGTACTCCACCCATGT 24 60.02 CTCATACGAAGAAAATAGGTACACC 25 57.304 186

chr1 22472181 GGGCCATGCTGAAATTAAAGTACA 24 59.84 GGGGTTTTCTTTGTCGGATTCAAT 24 60.021 189

chr1 22473537 TGATGGGTAGACAAGTGATTGAGT 24 59.468 GATGTCGAGGTTGGTTTGAACATT 24 60.023 202

chr1 22481066 CCTATCCACACCCTTGAACACTAT 24 59.836 AATTCCTCCTTCACCCTTCTTTCA 24 59.894 128

chr1 22485233 CGAGGAGTTGGTTATGAGGCTATT 24 60.202 GGATACACACAGAAAACAGATGCT 24 59.303 238

chr1 22492539 TGTGCTCAAACTTATATCCTCGAGA 25 59.64 AGGTTCGGTAGCCACTTTTATGTA 24 59.777 299

chr1 22493879 TAAGCTGCAGTATAACCGGAAACT 24 60.081 GAACACACGCTAGTAATCTTGAGC 24 59.968 239

chr1 22611878 TCACTTCCACCTCTTGCTCATAAA 24 59.958 TTAGGCGTTTAGGGAGTATGCTAC 24 59.962 186

chr1 22614576 GTCGCCTCTACCCTAAGTTTCTAG 24 59.963 GAGGGTTGGCAAATAATTATCTCTCTC 27 59.826 290

chr1 22653510 GGACTAACTAAGCAATTTCAGCCC 24 59.903 CTTTTCCGAGAGTTCTTTCCAACG 24 60.319 232

chr1 22713615 GCGAGAGTAGAAGTTTAGATCATCG 25 59.151 AAACCCTTCACTTATTTCCTAGCC 24 58.738 95

chr1 22714075 AACCACTACACCAGAACCAAACTT 24 60.569 CTCTTAAAGTATGGTGGTGGTTGC 24 59.844 278

chr1 22714280 GCAACCACCACCATACTTTAAGAG 24 59.844 GATATGGGACCTTCGAGAGTGAAA 24 59.9 134

chr1 22722529 ACCCTATACTTAGTTTCCTGTTGCA 25 59.75 TATATGTTTTCGACCAAGCCCCAT 24 60.386 279

chr1 22733616 TAAGGGGTGTAGAAAAGGTTGAAC 24 58.621 TCAAGTCCTCTTCTCAATCACCTG 24 60.021 281

chr1 22734554 AGTCGGTAATCATCCAATAGCCTT 24 59.652 TTTTAACCCAATGTCGCAGCAT 22 59.439 268

chr1 22734763 ATGCTGCGACATTGGGTTAAAA 22 59.439 TTAACGGCGAAAATCACTCACTTC 24 60.084 178

chr1 22811231 AGAAGTTTCGAGTTGTTGGAGTTC 24 59.429 TGCATAACTCTACCACAACTCCAA 24 59.959 229

chr1 22817087 TTTGGACACCCTCATTAACCTCTC 24 60.263 TATGCAAACTTCACACACACACAC 24 60.438 282

chr1 22819460 CACACCCAATTTCCAAGCAATTTC 24 59.786 AAAGCATTCCAAACTCTAACCACG 24 60.023 257

chr1 22876862 CCCGTTCTCTTCCCATCTATCAAT 24 59.959 ATTTAATAGGGAGGGTCACGTGAG 24 59.899 221

chr1 22879913 ATACTCTTTTCTCCCTTTCGCCTT 24 60.019 AGCATTGGAGTTTAGAGGTAAAGC 24 59.056 205

chr1 22880021 GCTTTACCTCTAAACTCCAATGCT 24 59.056 GGTGTCCCAAATGAGAATGCTTAG 24 59.903 254

chr1 22882469 GAAGGAGACATCACAGAAAGCTAA 24 58.517 GATTTGCATTCTCTCCGCCATAAA 24 59.963 284

chr1 22883306 AAGATCGGGAAGTGGGTAAATCAA 24 60.019 CGCTTCTCATCCTCTCTTTCTTCT 24 60.142 287

chr1 22883554 AGAAGAAAGAGAGGATGAGAAGCG 24 60.142 AAGAAGGTGGAAAAGAGTGGATGA 24 59.895 279

chr1 22885765 TTTGTATACTAACAGGTGCTCGCT 24 60.082 CCTATACTATACCCACGTGCCAAA 24 59.961 296

chr1 22885894 AAAAGGAAGGCAAAGGGATAGGAT 24 60.016 TTACACGCTCCTACATACATTCCC 24 60.202 195

chr1 22891111 TTAATAAACCACAACTCGCAACCC 24 60.023 TGTCCCAAACGCCCACTAATATAT 24 59.897 225

chr1 22896244 TGGCTGCATCAAGATTAACCCATA 24 60.386 CTTCAAGTAGACACAAAGACAAGCA 25 59.706 153

chr1 22899289 ACAATAGATCTCTTCATGGTGGCC 24 60.445 ACCAATCTAGACACACATTAGGGG 24 59.836 146

chr1 22899522 CCCCTAATGTGTGTCTAGATTGGT 24 59.836 ATCCCTCCACTCTTCTCATTTCAT 24 59.274 298

chr1 22902165 AATCAACTCAACACCCCAAATG 22 57.394 TTTCACAATGGGGTAAACACATCC 24 59.718 97

chr1 22902561 GTCGCACTTATTGTTTCCTATAACG 25 58.592 GAAGAATGTACGTTTGCTGCAGAA 24 60.319 263

chr1 22903469 TTTTGGTGGTTGTGCTACTTTGAC 24 60.381 ACCAAATGAACCTCAAAGATAGCC 24 59.293 243

chr1 22914313 TCTGAGTCGTGCAAATAAGGATCA 24 60.082 GAGTTTGGTTGGTGGATTGGTTTT 24 60.384 197

chr1 22921803 TTTGAGGGGAAAAGAACAAAGAGC 24 59.901 CTCAACTCCAACTTAAAGGGATTGT 25 59.23 248

chr1 22924709 ACCTTTAGTGGCGGAGTTGAATAT 24 60.081 GACTGTCGATGTATCTGTGTAGCT 24 59.964 135

chr1 22924959 TCTATGTGTTTTCCTCGTTTGTGG 24 59.486 ACGTCAAGATTAGCATTCACCCTA 24 59.838 236

chr1 22926251 TTCACCCCTTCATGTTGATCTACA 24 59.712 TGAAACCAACACATCAAGTCAGTC 24 59.665 290

chr1 22931418 CCAACTTAGGCTCTGATACCACTT 24 60.081 GACCAAGACTTGTCACACTCTTTG 24 59.965 283

chr1 22931531 CCAACTTAGGCTCTGATACCACTT 24 60.081 GACCAAGACTTGTCACACTCTTTG 24 59.965 283

chr1 22933863 TATTGTTGCTTTTCATCGTCTCCC 24 59.605 ATCCAAGGTAGCAACAAGACTCAT 24 60.019 129

chr1 22936279 AGAATCTTAAGGGTCCAAGTTCCT 24 59.147 GTTTAGGCGTCCAAATTCCAAAGA 24 60.023 297

chr1 22936617 TCTTTGGAATTTGGACGCCTAAAC 24 60.023 GAACTAGTTTCCAAGGAGCACAAC 24 60.024 261

chr1 22941364 CAGCAGCAGTGATTTGTAGATGAC 24 60.202 AGCTTCTTGGTGGAGTACGAATTA 24 59.778 246

chr1 22946279 GAATTGCACATATCAGTCTCGCTC 24 60.085 CCAGTTGCCAATAAAACCTGACAT 24 60.021 120

chr1 22950238 GCTTCAAGTCTAAGGTCGTGAG 22 58.753 TACCCATCACACTATTATGCGGAG 24 60.021 287

chr1 22954065 CGCGAGAAGAATAGGTGAGAAAAC 24 59.968 GGCTACTCATTTTCGTCTCTCTCT 24 59.903 259

chr1 22955540 GCATCACATGAGGAAACATTCA 22 57.293 GTTTAAAATGATCATATGCCACCGC 25 59.598 300

chr1 22957790 GAGGCAAGTAAAGCAAAGACGATT 24 60.083 ATTCAACTCTCTTCTTCTCCTGCC 24 60.323 253

chr1 22960169 AGAAGTGCATCAATCTCTATCACCA 25 59.87 ATACTCCTGAAGACCTGCATCATG 24 60.202 190

chr1 22965096 ACTAAATCTATCTGCAGTGCGTCA 24 60.142 CTCAGTCCACACATCAAGAGGTAA 24 60.021 229

chr1 22977169 ACCCACTCCAAATAAATCACTCCA 24 59.956 CCCACACAAATCTCAACCTCAAAA 24 59.901 276

chr1 22978714 AACATTATCCTCTTGCTTGCCTTG 24 59.841 AGTTCTGAGTTTTGAAGGGAGACA 24 59.836 261

chr1 22981636 TCCTATGTTTCCACTCGTTTATCCT 25 59.576 CATCCAACATATATGAAGTGCTCCA 25 59 299

chr1 22991196 TATATATATGTGCTAGCATGCCCC 24 57.886 GTTGAGAAACATCCAGAGGTACCT 24 60.02 276

chr1 22994962 TAATGATCTGCTCCGAGTTGTTCT 24 59.84 GCAACCTCCATTGATTGAGAAAGG 24 60.381 225

chr1 23003458 TCTACCAGGTTTGTCTATGCAACA 24 59.959 GAGTTCTCCCTGGAAAGTCTCATT 24 60.02 293

chr1 23005042 CAGTGACATCTTTTGCTACCAACA 24 59.724 AGCCATACTCTACAATGACGTTTTG 25 59.649 171

chr1 23008677 CTATCTACATTGTCGCTCTCTCCC 24 60.083 CCCAAAACTTCAGACACTTGCTAG 24 60.024 271

chr1 23014198 GGTGGACATGACTACGAAGGTATC 24 60.499 GCAGTCTCAGTCTTCATGTCGATA 24 60.202 259

chr1 23021879 TGTCACTCATTGTCCAAGATCACT 24 59.959 TAAATTGTCTAACTGTTCGCCGTG 24 59.849 263

chr1 23025105 TGTATGGTAGGGAAAGGGAAAGAG 24 59.526 AAGTGAGTTTGGTTCGTTTGAGTG 24 60.143 224

chr1 23101436 GCCCTCCGATCAAAATTACCAAAT 24 59.901 CAACTTTCTCTTTTGCCGATGTCT 24 60.024 229

chr1 23103461 CACCAATTTTAAGAAGACCAGGACA 25 59.465 GGACCCAAAATTAAAGGCCAAATC 24 59.059 285

chr1 23104705 ACCCAGGAGTCATAATAGTTGCAA 24 59.773 TATTATGGAACCCTGGGAGATTCG 24 59.714 295

chr1 23105426 GTTACTGCCCTTTACGCCATTAAA 24 59.844 TTAATCAGTGGAGAGGTTTCAAGC 24 58.996 169

chr1 23107103 AGCCAAGGAAGTCTAACAGAAAGA 24 59.652 TCTATTGGACACGACTTATATGGG 24 57.659 125

chr1 23119988 TTTCCTACCAAGTTTCACATGCTC 24 59.481 AGCTTTAGTGGATGTGGATCAGTT 24 60.019 295

chr1 23124031 GCTCCTTCTTTCTTCCCTGAGTTA 24 60.02 CAACTTCACCCTCCATCGTTACTA 24 60.082 188

chr1 23128500 GCAAGTGACCCATGATAATTTTGAG 25 58.962 CACTTTATCCCTTCAATTCGCCAA 24 59.842 184

chr1 23134593 CCACCACAGAGAAAGAAGAAACAG 24 59.726 AGATCGTCACCTAATTTTGCGAAC 24 59.907 232

chr1 23135133 AAACGATGATGTTCCTGAAGAGTG 24 59.307 AACGGAGGAAAGAGTTTGAACA 22 58.119 271

chr1 23136310 GCTTCATACACACATACACACATTC 25 58.749 TAGGATGAATTGGTGTGAAGGACA 24 59.712 264

chr1 23139124 GAGTGTGTCCATAAAGAACGTGTG 24 60.085 GCAGGGGATATAAGAAGGTAGAGC 24 60.081 155

chr1 23139489 GTAATGGCCCTTTCGTGATTCAAA 24 60.082 TCTCATTAATGGCCTTTTCCTGAC 24 59.05 296

chr1 23146276 GAGTGTTAATCATCATGGGTTGGC 24 60.202 CAAATTTCACTTCAGAACACCTACG 25 58.864 271

chr1 23148755 CCAACGTTGCTCAAATCCTTTCTA 24 59.785 CTTGGTTGGTTCTCTTGGTTCATC 24 60.023 162

chr1 23155342 AGCCCAATAGAACCATATGAAGCT 24 59.895 CAATGTCCTGCTTTGTTTTGTTTGG 25 60.448 284

chr1 23166993 TAAAAGAGGTTGTGAGGGTTGTGA 24 60.08 CGATGGGTTCTTGTTTTGTTGGTT 24 60.679 287

chr1 23182114 TGGTGGCTTAACTATTTATGTGCAC 25 59.875 CAGTTGAGCAGAAGAGAAAGAGGA 24 60.262 265

chr1 23205171 CACATGGGGTCCTACTCACATTAT 24 59.897 CTGACCTTTGCTGACTGAATTCTC 24 59.846 112

chr1 23245748 TAGGGAGGATAGGAAAAGGGAAGT 24 60.015 AATTGTTTCTCCCTTAAAGTGGCG 24 60.022 194

chr1 23251572 TGAGGTAGACATAGTGACATATCGA 25 58.469 ATCTATAGTGACCCTAAGCTTAGC 24 57.156 265

chr1 23251934 AGGAAGCCATACGTAAATCAAAGG 24 59.118 TTTTCAGTCTTAGAGGGCCTTTGG 24 60.75 191

chr1 23261160 ATTCGTATGAGACAAAATCCACGC 24 59.966 TCCTCCATGCATCATCATCATCAT 24 60.018 209

chr1 23271217 TCCATTACTTCCACGTCTTTTAGTC 25 58.839 TTCATTTGCCTAAAGTGTGTGACC 24 59.963 300

chr1 23279214 ACCTCGTCCAAACATGTTATAGCA 24 60.323 GTGCTATGTCTGTTATGCCAAGTC 24 59.965 284

chr1 23281954 TCAACCATGTAAGTACTCGAAGTGA 25 59.758 GCTTCTTCGTTTACTGTCGTGAA 23 59.512 286

chr1 23288679 CCCTGTCGAAATTTGATCTAAACAG 25 58.454 GTCTAGTCAGCTTGAACACATGAA 24 59.009 287

chr1 23292133 CCTGAAACACCAAAACCGATACAA 24 59.963 TCTGAATCCGTACACTTGCTTGTA 24 60.022 288

chr1 23293248 GAGAGAAGACAATGTGGAGAGGAA 24 59.778 TGAGAATGGGATATGGTCAAACTTC 25 58.878 274

chr1 23298067 GAAGTCGATTGGTCAGAAAACGAG 24 60.143 GATTATTGGTTGATTGGAGTCGGC 24 60.261 210

chr1 23305519 CCCACCCTAGAAGCAAATAAACAC 24 59.842 TTATCCCATTGAAACTGCTGTGTG 24 59.782 181

chr1 23307569 CCTCTGCCCTTTCTTTCTTTCAAG 24 60.023 TAGGCAGTTAGATGGTTGAAGTGG 24 60.323 107

chr1 23317725 ATTCCATTTTACTCCTCCCACCTC 24 60.08 AGTCCATATACTTTTGTCGGCCAA 24 60.324 221

chr1 23318854 AGTTCCTAGATCAGCCCAAACAAT 24 60.018 ATTCCTCTGCATAGTCTGTTGGTT 24 60.019 294

chr1 23334005 TGAGAGATTGAAACCGAGTATGCT 24 59.84 TTTTGCCATCCCCTTCTCTGATTA 24 60.018 225

chr1 23339578 ATTTAGCTTGGTATCATCGGCAAC 24 59.724 ACTATCAAGTCTGCGTTTCTCTCA 24 59.781 155

chr1 23346625 TATGTATTTCCACCCATCTCTGCA 24 59.588 GCAGGACCCCAAATTTTAGAATGA 24 59.534 253

chr1 23348521 TTGAGATGAAGGACTTAGGAACCC 24 59.775 AGTGAGTCGTACATTTTGGATCCA 24 60.021 185

chr1 23441380 CTTGCAAGTGGTAAATCGTGTATAC 25 58.525 CCTCAACGCCTAAGTCCTGAAAG 23 61.17 300

chr1 23451334 GAATGCCCAAAACACCTCAACTAA 24 59.961 AAGGAAGAAGGAAGAAATTGGAGC 24 59.231 214

chr1 23457465 CCAAGGATCCCGAATTGTTCTCTA 24 60.142 GTGAAGCTGGCCAATGTAGAAAAT 24 60.082 148

chr1 23459975 ACTGATGCTTGCAAATTAGGGTTG 24 60.322 CATCAACCAAGAAAGGAAACGTGA 24 59.964 295

chr1 23461726 TGATTTCATGCGCTATCAAAGGAG 24 59.726 ACCATCGACATACAACGTTTAAGC 24 59.908 282

chr1 23461871 GTTGCAGCTGTATGTTTGTTGGAT 24 60.559 CCCAAACATCATCATTCAACCACA 24 60.021 155

chr1 23573905 TGGAGGTCAATAATTCACACCACT 24 59.957 TCTCCTTCTGAATCACCCAATGTT 24 59.957 269

chr1 23574328 GACAGCCACAGGAATCCATTAATG 24 59.963 ACTTGGGTTTGGGAGAACTTAGAA 24 59.833 266

chr1 23590962 AAGATGGGACGTGACAATTAGCTA 24 59.838 GACAAACGAGGTGTGAAGAGAATC 24 59.849 174

chr1 23593365 AATCTAGAGGTCCATCATTGAGGC 24 59.959 AAAGAGGACCAAATGAACTCCAGT 24 60.141 210

chr1 23597476 TGGGAGAGTATGAAATCAGGAGAA 24 58.72 CTTGGTAGGAGAAAGGAGATAGGC 24 59.96 275

chr1 23599070 GTCAATTCGAGTTGGATGAAGAGC 24 60.202 GCCTAACTTTTCTAACAAAACAGCC 25 59.31 247

chr1 23601669 TCAACCCTAAGCAAGTTCTATGGA 24 59.466 CGGGGAGATCTATGGGTATGTTTA 24 59.224 259

chr1 23607204 TATCAGCCATGTGTGAGTTTACCA 24 60.02 GAAGTTGATTGCCTTTGATGACCA 24 60.022 225

chr1 23613298 TGGCTCACTTACTTTCACTAGTCG 24 60.321 AGGCCACCAAATTCCATCAAATTG 24 60.566 296

chr1 23624839 TGGAGAAGAATTTGTGTTTAGGGAC 25 59.232 ACTGTAAAGAGGTGTATCGCATCA 24 59.84 265

chr1 23626362 TCAGGATTCCATATTTGTCGTTGC 24 59.665 GCAGAAGAGTAGAGGAAAGACGAT 24 59.903 191

chr1 23643166 ACAAGATCAAGGACACATTGGTTG 24 59.721 CCCTTGAATCTCATTTAAAGCCGT 24 59.601 162

chr1 23646606 GCGACTGGACAATGATTGATATGG 24 60.084 CAACCTAGCCACTAATAGACCGTA 24 59.418 280

chr1 23648534 GTTTATTGGGAGTTGTTGAGGGTG 24 60.022 CAATCTTGGTCGAAAACGTCCTTG 24 60.844 207

chr1 23651727 CGATTGACAATTGAGAAGCACAGT 24 60.084 GGGAAACTTTCTCTCTCAGCTCTT 24 60.263 272

chr1 23652479 CGTCCATTCTCCTCATCTCTTTCT 24 59.901 TGAACCATTGAATCTAACGGAGGT 24 60.02 266

chr1 23655362 AAGAGGAAGCCACTGTTATTACGT 24 60.02 TGCCTGTGAACTGTTACCACTTAT 24 60.202 291

chr1 23655490 AGAGGAAGCCACTGTTATTACGTT 24 60.02 TGCCTGTGAACTGTTACCACTTAT 24 60.202 290

chr1 23659328 GACAAACAACTAGGACGAGAGGAT 24 60.082 ATTATTGGTCAAGGTCAAGGTCGA 24 60.02 233

chr1 23662643 TCCCTCTTCCCTCACTTTCTTTTC 24 60.202 AGTGGGAGTAGAAAGAATCGGAAG 24 59.839 227

chr1 23670295 TTCAAGCCCACAAGTGAAGGT 21 60.064 CACAGTAACCTCCCTCTCACTTAC 24 60.082 299

chr1 23688159 TCCATCCTAGAGAATATTGGAAGCT 25 58.913 GAACAAGCTAGCGATGGAATTCAG 24 60.261 288

chr1 23691145 TCATTCTCTCCTACTCTCTTTCCT 24 57.914 ATCTCCTGGGATAAGATTGGTTCG 24 59.959 127

chr1 23692016 AGAAGGGGAAAATGTATGCCTTCA 24 60.264 CTTGAGGCATGAAGTTTCCAAC 22 58.094 213

chr1 23873080 TACACAAACCACCACAAGTTCCTA 24 60.08 TCCAGTGTTCTAAGATACATGAGCA 25 59.578 247

chr1 23971785 AAAAGGGAAAACACATGTAGCGAG 24 60.023 TGTGTGTGGAGATATTACAAGTGGA 25 59.753 285

chr1 23975585 CCTGAAGTGAAGGTAAGAAGTCCA 24 59.96 ATGCTCTTCTACCCTGTCCAAATT 24 60.018 227

chr1 23979679 AATCTCCCCGCTTTTGAATCAATC 24 59.901 CAACTTGGGTCCGTTTCGATTG 22 60.35 208

chr1 23980839 ACTCCAAGTCTTCTTCTTCTCACC 24 59.96 TGGACCATGTGTAGTGTTTAAGCT 24 60.202 286

chr1 23990686 TGCCTCTATCACGTGAAATTAGGA 24 59.596 CAATGATAGGTTGGCATGCAGG 22 59.965 298

chr1 23991117 TTGATGTATAGGGTATGGTGGACG 24 59.718 TTCTGTTGCCACCCTCATACTATC 24 60.142 253

chr1 23994473 TAGAATGATGGCTTGGTCTTGGAA 24 60.019 CTCCCAAAACTCAGCTCTAGATCC 24 60.443 262

chr1 23997686 GAGGGTCAAAAGAAATGCCGTGAT 24 61.878 CATGAAACTTGACCGTGCTAACAT 24 60.083 298

chr1 24052754 CAACCGTTTGCAAATACAAGCT 22 58.62 ATGGATGTTGATGAGGGAGAAAGT 24 59.771 198

chr1 24056474 AAGATGTGTTGTCATTGAGCTGTG 24 60.023 AAAGTTGATGGGGTTGAGATCAGA 24 59.957 231

chr1 24060023 CACACCACAAACTCAACACCAG 22 60.159 CAAGTGAGATGACATGCCAATAGT 24 59.12 299

chr1 24060316 GCATGTCATCTCACTTGTTAGGAC 24 59.669 GGCAACTTCATGAGACTTTCCATT 24 59.781 144

chr1 24061999 CGAAAGAATGGAGGTGTTGAACAA 24 59.964 CCCAACCAAAACATAGAAGCCATT 24 60.02 129

chr1 24152789 TAAATCTTTGAGAGGACGCGCTAA 24 60.381 GATTAACCATACGAGTCTATCAACG 25 57.45 280

chr1 24153696 ATGTACCCCTAGCAAACAACTCAT 24 60.018 TAATCCGACTTCTTACTCAGCGTT 24 59.843 275

chr1 24162212 TATCTCCTCCTTCTGCAAACATGT 24 59.774 GAGAAGCGTTTGGACTTATGATCG 24 60.026 220

chr1 24179440 CACTCAGCTTCGAACCAAATGATT 24 60.083 AGGATCAAGTTACAATGTCCCACT 24 59.71 152

chr1 24179743 AGTAACTATCAACATCGACCACACA 25 60.048 CGATCGTTTAACATGGTCAACA 22 57.345 199

chr1 24193936 ACCCCTTCCTTTCTCCATATTTCC 24 60.079 AAGAGAAGAGCGAGAGAGAAGAAG 24 59.604 291

chr1 24196802 CCTTTGTCTAGGGTTTGGGG 20 57.784 GAGTCACCATCGTAAAAGTGCTTT 24 59.787 122

chr1 24219991 CTTATCCATACCCTCCACCTATG 23 57.316 GGGACGTTTGCAACAATAGC 20 58.023 300

chr1 24220417 CCACATGCAACATAGTTTCAATGC 24 59.672 TTTGGGCCAACTCGAGGATAATAT 24 59.896 259

chr1 24243610 AAACTCACATGGGATCTTGAATGC 24 59.841 GGCTTGAGAGTCATACTTGGGATT 24 60.385 261

chr1 24271620 TGCTTTCCCTTTTGACATGAGTTG 24 60.202 GTTTAATCTGAGTGTGTGTGTGCT 24 59.727 121

chr1 24273174 TCGTTTCAAACCAATCTAGGCCTA 24 60.02 CCATTTAATTAGCTGGGCCAAA 22 57.248 265

chr1 24274655 TTTTCTGCCTGGTACTCGTAACTA 24 59.476 GTCCATAACCATCAAGTCTAGTTTG 25 57.691 198

chr1 24280003 CTAAAAGATGGGGAGGGAAGTGAA 24 60.018 GGACCTCTTGATCTCTCTCACATC 24 59.962 122

chr1 24284684 TTGATCATAGTGTTGGGTGTGAGT 24 59.958 TGGCCTCCATTTTAGTTCTTTCCT 24 60.203 258

chr1 24285143 ATCCATACAGTCCAAAATAGGCCA 24 59.832 GCTATGCAAGTAGTGAAGACGTTG 24 60.202 211

chr1 24286174 TTGTTGTGTGCATGTGGTTGAAAC 24 61.384 TTGTCGTCATTCAGTCACATGTTC 24 59.789 228

chr1 24306186 CCCCAACTAATCGCCATATTTCAC 24 60.023 TGTTCTGGACCCTTTAGAGTTCTG 24 59.96 258

chr1 24314012 GGCTGTAAAGTGCAAATCTACGTT 24 60.083 AGGAAAGTGAAGTATGGTGGCTAG 24 60.081 236

chr1 24315180 CAGTGGTATCCTTTGTTTTGTTCCA 25 59.932 CCTTCCTCTTCAAGAATCTGTGGA 24 60.02 239

chr1 24315846 GGGTAGGTAATATTTAGGGAAAGTAGG 27 58.399 CATCATTTTCTCGAAGCTTATGAGG 25 58.343 284

chr1 24316038 CCTCATAAGCTTCGAGAAAATGATG 25 58.343 GTCTAACCCAAATTACAACTCTTCC 25 57.858 189

chr1 24317606 GCCTTAGATTGTTTGTACGGATGG 24 59.965 TCGGTAGGAACACACATCAAATCA 24 60.263 151

chr1 24365484 ACTTCCATCGCAAAATATGTAGCTC 25 59.763 GGCGAGAGAAATGTTGATATCACA 24 59.189 275

chr1 24401823 GGCTGCATGTATTGAACTCATCTC 24 60.024 ACTCTCTCCTTATAAGTGTGGGGA 24 60.016 188

chr1 24404262 TTTGTCTGAAGGGGAAGAGGAAAA 24 60.079 AAATGAAGTTGGTGTTGAGAAGGG 24 59.658 244

chr1 24476962 TGTTGTGACGAGGAAATGAAAGAG 24 59.489 TGTTGTTGATTCTCCTCCTTCTGT 24 59.897 202

chr1 24499746 CCCAAACCTTTCTGACCTACTTCT 24 60.202 GGATTACAATGATTGCTATGTCCTG 25 58.093 298

chr1 24503281 TGGTAAGGAACAAACGTAAAGAGAC 25 59.245 GCTTCTTGTGATCATTGTTTTGCC 24 59.849 258

chr1 24509744 ACCACAATAGAGATATCACGAATGG 25 58.541 CATTGGCAATTGGTAGCTCAAGAA 24 60.082 283

chr1 24512645 GAGTCTTGGCCGATTATTGTCATG 24 60.025 GCAATTTGGCCATACAATTTCACC 24 59.905 239

chr1 24512899 GGGAAACACGATTGCTTGTAATGA 24 60.083 TCTCGAAGTCCAGACATGTCATTT 24 60.021 269

chr1 24514821 TTAACACTCTTTCATACCATGCGC 24 59.906 ACATTCTTGCATTCTCTTCTTGGC 24 60.082 231

chr1 24515780 GCATTTGATTGAGCACTACCATCA 24 59.903 GTTTTCCCCACATGAATTTGCTCA 24 60.503 147

chr1 24528085 TTAGTTTACCAGGCATGACTACCG 24 60.382 ATGCTCTTTATCTCTGTCGACCTC 24 59.963 247

chr1 24542433 CAACGAGAAGGAAGAAGGAGAAGA 24 60.022 TTACCACCGGACATCATCTTCTTT 24 60.02 84

chr1 24547032 ACAAATGGAGAGTGAGTAGGGAAA 24 59.402 TGGTTAGGGTTTAGGTTTTGGAGA 24 59.584 236

chr1 24564067 CGGCCATTACAAAACCTGAGATTT 24 60.082 ATTTGATGACACTGAGAGAGAGGG 24 59.838 215

chr1 24570856 CTTATAGAGTGCGCGAGAGATACT 24 59.609 CCCACATCTCAAATGGTTCATGTC 24 60.142 182

chr1 24580393 AAACCCAAGAAGCAGTTGAGAAAG 24 59.901 TTGTGGGATTTCTTTCTGGGTTTG 24 59.899 167

chr1 24590993 GCGCAACGAACAATAGATTCACAT 24 60.728 CTAGAAAACACCAACCATAAGCGG 24 60.143 281

chr1 24833987 TAGGTATGGTGGTATGATTCTCGC 24 59.78 TTGAAGGCATGCTTGTCAATTACC 24 60.322 268

chr1 24835075 GGGAAGATCAGAACTCAGGTGAAT 24 60.081 AGAGAGAATTTCATCTGCCCTGTT 24 60.019 146

chr1 24845349 GGAAGTTTGTCCCTTTGAGAAGTT 24 59.355 CGTTCGATCGCATGTGAAATTTTC 24 60.029 202

chr1 24848877 CGGTAGTGTTTATTGTTGAGGTTGA 25 59.533 AGCATTCATAAGGGAGTGTACATCA 25 59.869 279

chr1 24849146 TGATGTACACTCCCTTATGAATGCT 25 59.869 GACAGGTTTTGATGTCGTGGATTC 24 60.378 299

chr1 24849374 CTCCGTCTACAATTTCACTGTCCT 24 60.322 TCCTAACAGCATTACTCGTAGTCA 24 59.054 224

chr1 24849545 TGACTACGAGTAATGCTGTTAGGA 24 59.054 TTAGCCACCTCCTAGTACACCTAA 24 60.017 176

chr1 24851424 AAAATCAGAGATACCCAAGCGAGT 24 60.081 GTGAGGAGTGGTGGATAAGTCAAT 24 60.081 152

chr1 24853254 AGAGATTGGTTAGAGTCGTTTGGA 24 59.473 TGAAATCAAGTTCCCGTCACCTAT 24 60.02 237

chr1 24878450 AGGAGAGAGCTAGAATTCAAAAGGT 25 59.513 TACTACTAGGTCTATCTCGCGACG 24 60.615 299

chr1 24878633 AAAATTACGGATCTTCAAGGCGTC 24 59.906 GTTGGAGTGAACAAAAGATGGTGT 24 59.902 243

chr1 24882680 ACCCATCCCCAAAATTAAAAGTGG 24 59.712 AAATATGTGTTAGTCAGCAGGGGT 24 60.018 219

chr1 24884214 CAAACCCTCTCGAACATTAGCTTC 24 59.906 GGAACTCATCATCACTCACTTAGC 24 59.192 255

chr1 24889283 TTTATTCTTGTCGTGTTGTGCTCC 24 60.024 TGGCAGTCAGTAATGGTGAAGTTA 24 59.959 241

chr1 24890845 GCTCTCTAGTGCTTCCAGGAATAT 24 59.717 CACAGCTAACTCTCATAGGGTCAA 24 59.84 269

chr1 25062011 TGCCTTCAATCTTCTCATCCTTGA 24 60.019 ACAGATTTAGTTGGTGCACAGGTA 24 60.202 296

chr1 25063303 AGATCTTGTGAATGAGGTGCTACT 24 59.531 GGATATCAAGAGGTCCAAGTCCAA 24 59.836 295

chr1 25067402 GAGTTTGCACCGTTTTGAAAAG 22 57.501 CATCTTTCACTCCATCAAAAGCAGT 25 60.048 253

chr1 25072363 TACTTTTGTCTCTGCATTTTCCGG 24 59.785 AACCCGGTTTCTATTTTGCATTCG 24 60.617 255

chr1 25077933 ATAGTCGTTCGTTTAGTCCTTCGA 24 59.607 GTTATTGGAGATTGTGTGTGAGCC 24 60.143 266

chr1 25079657 GTCTTCGTCTCCCTATGCTATTCG 24 60.614 GAAATCGAAAACCAATGCATGCAG 24 60.202 247

chr1 25079867 ACCAAACCGAACAGCATAAGAAAC 24 60.261 CTTCCATGCGATCAGTTTGTTCTT 24 60.083 279

chr1 25085036 TGAGTAGAATGAACACGTACCTCT 24 58.992 GCAATCCAAACACACTCTAAATACG 25 58.978 175

chr1 25248795 TCCTAAGGTGTGTGTCGGTTTTAT 24 59.959 CACGTACTGTTTGAGAATTGATGC 24 58.918 234

chr1 25264340 TTTTGATGGGTTGGGTTGTGT 21 58.528 CCGGTTGGGTCAAGTTAGATGA 22 60.028 204

chr1 25264691 TGTGTTGCAATAAGAGTGAGCA 22 58.524 TTTTAAGCCCAATCCCAGTTCAAC 24 59.96 132

chr1 25265584 GAAGGTTCTCGTTGGTATCGGATA 24 59.963 TTACAACATTCACAAAACCAGGGC 24 60.442 161

chr1 25293385 AATCTCAGACCATCGAACCATCAA 24 60.081 ATAGCTTTGACTTATGGGAGGGTG 24 60.141 284

chr1 25300058 AGTTGGGTTTTGGCGAATAACTTC 24 60.262 CCTAGTTGGCAGTGAGAGAAATAAC 25 59.417 281

chr1 25303336 CAGCCTAAACTTCTCTTCCTCCAT 24 60.081 CACTTTTCACCTATAATGCTATGCC 25 58.328 283

chr1 25312849 TCTCTTGGTCTCTCTTCCTCTCAT 24 60.018 TCGTATTCCTCCTTAGCACAACAA 24 60.021 159

chr1 25317906 TGCCCAACAATTATGCATATGACC 24 59.961 CAAGCCCCAACCGACATAATAAAG 24 60.202 292

chr1 25328960 CATTCGAACACCAATTACGTCA 22 57.345 TTCTGCTTCTCACAATCTCGATGA 24 60.082 270

chr1 25333442 TCTGCTGTAGTCAAAGTCAAACAC 24 59.429 TTTCAGTACTTCCCGGAAAAGCTC 24 61.041 254

chr1 25335736 AACCTTCTTCTCATCTTCCCCTTC 24 60.019 CCTCACTTCGGTATCCTCTTCTTC 24 60.202 225

chr1 25335849 AAGTGAGGCTGTTCGATAAGGATC 24 60.442 ACGTTTCTTTCGGAGGGATTTAGA 24 60.021 281

chr1 25345837 CTCAAAATGAAGGTCCAAACAAACG 25 59.768 TGACTCAGAATTGGGAAAATTGGC 24 60.021 154

chr1 25349687 CGATTATGAGTTGAAGTAAGCCGT 24 59.196 TTTTCTTTAACGGCGTTGACACTG 24 60.728 277

chr1 25349875 CAGTGTCAACGCCGTTAAAGAAAA 24 60.728 ATGTACCTGAGAAGTGATGATCGG 24 59.961 207

chr1 25350858 TTGTTCGTGGTTAAGTCAAAGGTG 24 59.905 CCAACTTCATAGGCACAGCTTTAG 24 59.905 261

chr1 25361865 ATAACGAGCATAATATTGGGGCCT 24 60.018 TTGTGGGTCTGGTGTTGATTGTAG 24 60.986 286

chr1 25753254 ATCGTACCAGGAGCCCAATTATTT 24 60.141 CACTTTCTTCCTTTCCTTTCCCAC 24 59.962 282

chr1 25766049 CATGCATCCTCCCCTTATCTAGTT 24 59.712 CTATTGATCCGACCACCTACTAGC 24 60.083 181

chr1 25920721 TGGCCCCAAATTCCTTCTACTATT 24 59.768 CGACGGTGCTGGTTATATTTGAAA 24 59.906 210

chr1 25923322 TAATCCATCAAAACAACCTCCCCT 24 59.955 TAGATGGCAAGAAATGGGAAAGGT 24 60.264 138

chr1 25926230 CATCATCATCCCGTTCAACAAAGA 24 59.604 GTGGAATAGTTGGTGTGAGACTCT 24 60.021 198

chr1 25926852 CAAATTATGAGATAACGACGGGCC 24 60.084 TCAAATGAGTACCCAAGAGCTTCA 24 59.958 288

chr1 25928273 AAGAAAGCCTATACGAACGAAGGA 24 59.841 ATTGTCCACGTGTCAATTCCAC 22 59.448 290

chr1 25941198 GCGAGGAAGTTCATGTTAGGACTA 24 60.142 GCTTAAACTTCCTGGTAAGATGCC 24 59.903 160

chr1 25949226 GGTTGTGAAATTGGCCATTCTCTA 24 59.538 CTTCTTCAGCTGACGTTTTCTCTG 24 60.085 239

chr1 25972252 GAAACGAGCAAAGCAAAGAAAGTG 24 60.027 TACCGTTCTCTTTTAAGTTCCCGA 24 59.719 120

chr1 25980497 CTTCTCCACTACTCCACTCAAACA 24 59.961 AAGGTTGGTCTCTCTGTCTCAAAA 24 59.836 220

chr1 25984867 CTTGTGTACGACTATGGGCAGATA 24 59.962 CTTGGGATTTTAAAAGGGCACAAC 24 59.245 172

chr1 25986185 TGGATGCAAATCTATGGACAACTTC 25 59.642 GAATGACCATTTTAGCCTTGCCTT 24 60.081 268

chr1 25992705 TTCAAACCTCCAACAACACATGAC 24 60.142 GTGGCTTTATTCTCACTCCGATTG 24 59.965 131

chr1 25995060 CAAATTGAATCGAACCAAACCG 22 57.116 TTACATTGTTGATCGGATCGGATG 24 59.25 244

chr1 25997458 CGGCCACTCTTATTGACTTTCTTT 24 59.544 CAATGCAATCCCAAGAACCCTAAA 24 59.777 279

chr1 26016151 TGCTTATCATCAACCAACCCTGTA 24 60.019 GGATTTAGTATAGGCTTAAACGACGAG 27 59.674 199

chr1 26165318 TTTGTGCCAAGGAAGAAACTGTAC 24 59.903 CGTCAGGCGCATACTTCTATATTA 24 58.362 134

chr1 26170374 ATTCATCCATAGTGCTTCCTCTCC 24 59.959 TGTTCGAATCCTTTCTTTAACGGC 24 60.084 138

chr1 26187979 TCTCAAGGCAATGTAGTAGCTCAG 24 60.142 TCGAGAATAGCAATGACTCCAACA 24 60.082 220

chr1 26228299 GAAGCGTTGGTGGGTTTTGATTTC 24 61.554 TGTCATTGAATCATAACCTCAGCTG 25 59.413 223

chr1 26235023 CGTTGATTCTTAGCTTCACCATAAC 25 58.521 AGTAGCCTAATCCGATTGTCCAAA 24 59.837 288

chr1 26252097 CCCACATATTCCTCGTGCCTTTAT 24 60.747 TGAAGGTGAAGAAGGTGAAGAAGG 24 60.445 172

chr1 26253974 GAGAAAGCTAGTGGGAATGAGACA 24 60.081 TCTACGAGACAACCACCAGAATTT 24 59.96 232

chr1 26254631 TCGACTTTGCCCTTTAGTTTCA 22 58.189 CCCGCAAAAGTCAAAACAAATTCC 24 60.261 250

chr1 26255669 AACCTAGCAAGTGAGAAGATACCA 24 59.222 GTTGTGCACTCGTTGGATTAGAAT 24 59.846 250

chr1 26255818 ATTCTAATCCAACGAGTGCACAAC 24 59.846 TGTAATTGGCTTTTCTGTCACGTC 24 60.024 293

chr1 26259224 CAGTTGGTTCATGCATGTGTTAGT 24 60.023 TATAGTTAGAAGGAGTGAGCGAGC 24 59.484 245

chr1 26497356 TTGCTCCAACCTCATGACTTGA 22 59.894 TATTGTTGGGTCAAGTGGATGAGT 24 59.957 261

chr1 26506145 ACACTCCAAATCCATGCATGTTAC 24 59.842 AAAAGAGAGAGGCGGCTTAAAGAA 24 60.505 268

chr1 26509117 CAGAGCAGTTTTCCTTGATTGTGT 24 59.963 GGTGGTGCCGCTATACTTATCTTA 24 60.022 194

chr1 26511766 TTCGTTGTTGTACTAGAGACCGAG 24 60.084 AGAGAGAGTGATTGGTAGGGGTAA 24 60.016 266

chr1 26525912 CAAGCTACTATGCCAACAACAGTG 24 60.379 GGAACAGATTGCTACATATGGTGG 24 59.483 297

chr1 26535171 TTTGTTATTGTGTGTGTCCTCGTG 24 59.965 AGGGTGAAAAGAGAGAGAGAGAGA 24 59.956 102

chr1 26535334 TCTCTCTCTCTCTCTTTTCACCCT 24 59.956 TACTTTTCACGTCTCTCTCGACTC 24 59.848 281

chr1 26540256 TATTGGAAGGATGCATGACTGGAA 24 60.08 CATTGGAATTGCGTAGAGTCTTCG 24 60.26 259

chr1 26541967 AGAGTGTGGTTGATAGAGAATTCGA 25 59.581 GGTGAGTGGAGATAAAACATGACG 24 59.67 249

chr1 26544467 CCTGAGAAAATGTGGGAAAATGCT 24 60.021 CAGCCAAGTTTTCGGTGAAGTTTA 24 60.202 144

chr1 26545348 CCAGTATACAGCAGCCTAATGAGT 24 59.96 CCGTCCAGTATGTGAAGAGTCTAG 24 59.964 280

chr1 26547325 TTGGTGCAAAGGAAAAGGAGATTC 24 59.961 GAGAATCTACTGCCTTCATTCCAA 24 58.568 258

chr1 26581298 CAAATGGTTGTGGGGTAAGGTAC 23 59.492 GAAGAAAAGGAGGAGAGTGGACTT 24 59.959 267

chr1 26584098 TTGTCTCCACGTCATCAATTTTCC 24 59.785 ATCTTTTCGACTTACTGACCGTGA 24 60.023 219

chr1 26797828 AAGGTTATTGGAGAGGCAAGTCAT 24 60.018 TTAAGGAATCTACGTTGGCAACCT 24 60.263 194

chr1 26810678 GTCGAGCCAAACAATAAGTGAAGT 24 59.787 ACTCAACCCTAGACATGAAGACAA 24 59.407 249

chr1 26813391 TCCACGCTCATTTGATCTAAGTTC 24 59.13 GTGCTCGTGTGACAAAACAAATAC 24 59.563 299

chr1 26815716 AATTCCTCCCGCTTGTCCATAATT 24 60.631 CACTTGGGTCATTCAACTTTTCCA 24 59.901 251

chr1 26819169 AAGCAGTTCAGAGAGTGGAAGAAG 24 60.503 AGTAGTAGAGTTCTTTGGCAAGGG 24 60.02 297

chr1 26822853 TGTTGGATTCATGTGACGGAACTA 24 60.263 TTAGCGATTGTGTTCTTTCCCTTG 24 59.785 290

chr1 26825706 TTTTCCTCTATGCCTCCAAACTCA 24 59.957 ATGGGTAGGAAAGAATAGTCGACG 24 59.962 165

chr1 26832841 TGGAAGCCACGTCAACATATAT 22 57.584 TTGTAAGGGCAATTTTGGAAGGTC 24 59.96 280

chr1 26833036 GACCTTCCAAAATTGCCCTTACAA 24 59.96 GTAACCAACTTAGTACTTCAGCACA 25 59.013 139

chr1 26842119 TGACATGGGAGAAGGAAGAAACAA 24 60.141 TTGTAGGCACCTCAAATGGAAAAG 24 59.719 269

chr1 26857574 ATGATGTGATGGCTGTGAAAATGC 24 60.68 CGAAGTACTCCCGAACTGATGAAT 24 60.44 285

chr1 26858983 TGTGGTGGATGAGGAAGGATTAAG 24 60.08 TGAGCTAAGTTAAACGTCAGTGGT 24 60.202 202

chr1 26907516 AGTGACTAAGAGAGGTTTGCGATT 24 60.021 AGGAGGATGAAAATGAGATGGGTA 24 59.022 190

chr1 26913771 AGTATTAATTTCCTCGTCCTCCCA 24 59.033 GCAAACCTTCCCCTTTTCATACAA 24 59.96 117

chr1 26921019 GTGACTAATTAACATCCAACACGTC 25 58.469 GCCAAATGTAAATAACCGTGGACA 24 60.083 252

chr1 26926326 GGATTACTTTGCCAACTCGTTCAA 24 60.024 CGCATGAAGGTGAAGTCGATTTAG 24 60.26 280

chr1 26926717 CTTAGACTCGGTGCTCAAAGTTTC 24 59.849 TGGTGAGGTAGGTTATCAAAGGTG 24 60.02 260

chr1 26932143 TTGGTTCGAACAATGAGAGCTCTA 24 60.021 ACCGCCATTGATGATCGATATGTA 24 60.082 275

chr1 26973160 TGGTGTGGAATTAGTGAAGTTACCA 25 60.163 ATTGTTGGAGGTTGTGGAGATACA 24 59.957 273

chr1 26994766 CTTGTATGGGTTAGGTCGAGTTCA 24 60.082 CAAATGTGTTCCCATCAAAACGAC 24 59.556 300

chr1 26995329 AATGGATAAGATGCTTTTCCCTGC 24 59.657 CACTCCATCCACAAACGAAACTTT 24 60.202 250

chr1 26997982 CTAACATCGAAACCACAAAAGCGA 24 60.319 CACACTTTAATTCCTTCGACGACC 24 60.143 276

chr1 26998465 TTTTGGCCCTTCATTTCAGTCCC 23 61.575 GCGGATGCGTTTAATTGTTAGGTA 24 59.966 124

chr1 27000737 TATCCTATGAAAGCCACACTCACC 24 60.142 AAAGCATATCGTAGTTTCCCTGGT 24 60.081 206

chr1 27002112 AAAACGGTTGTGAAAGGCATCT 22 59.307 GAACATTTCAAATGCCTTCCTTCC 24 59.068 136

chr1 27005778 CGTGAGTGGTGAAAAGTTCTTCTA 24 58.952 CATCTACCCACATGACAATATTGCA 25 59.468 197

chr1 27006742 ATGCTAAGAGAGGTCAAATACGGT 24 59.594 GTGTTCAACTTCCTACGACTATTAC 25 57.496 187

chr1 27016022 ATCCAAAAGAACAAGGCTAGAGGT 24 59.957 GTCTCATCGGATGTTGGTCTTTTG 24 60.143 154

chr1 27322099 TTTGCATGATTAGAAACCCAGACC 24 59.538 TCTGATGGAGTTCAAGTGTCACAT 24 59.959 275

chr1 27436354 CTTCTCCTTTCACTTTAGCTACGC 24 59.672 TGTCAAGAACCAAAATATGCCCTC 24 59.538 117

chr1 27437288 TGGCCATCAATTTTACCCCAAA 22 58.749 CGGCTAAATGTCAATTAATCACGGA 25 59.993 216

chr1 27439332 ACTGTCCTCTCTCAATCTCCTTCT 24 60.264 GGGTGGGATTTTCAAATAATGGCA 24 60.081 266

chr1 27465004 TGTTCAATGTCATGTGGGGAAATC 24 59.78 ACATTGGGTGGAAAATCGAGATAGA 25 60.105 152

chr1 27465884 TCGCCCATGACACATGACATATAT 24 60.02 CCTTCTTGGCTTTAGTTCGATTGG 24 60.143 215

chr1 27486491 CAAACGAGGAAAATTGGAGGTGAA 24 59.963 ATTCCCTCTTGATTTACACCACCA 24 59.956 268

chr1 27495310 CGCCTTTTCTCCTACCAAGATCTA 24 59.9 CATTAATTGGCATCGTCGTTGTTG 24 59.737 155

chr1 27495717 GCTTTAGCGTGGTTTGAGATTCA 23 59.812 GTTCAGAATACATGGATTAGTGGAG 25 57.279 245

chr1 27499534 ACAGATTCTCAATTTCCTGGTCCA 24 59.957 TAAGTTCGAAATATTCTCAGCGCC 24 59.493 239

chr1 27508567 CGACAGAGAGATTATCGTTAGGGA 24 59.244 GCCACATACTTCAGATGCTATAATG 25 57.93 280

chr1 27531062 CTCTTCTCGATTTCCTTCAACTGC 24 59.907 TTGAGTGAAGAAGGGAATGGGATT 24 59.955 229

chr1 27535146 TCTGCCTCTCTGACTATTGCTTTT 24 60.02 TGTCATCATCAAGAACCACCAGAT 24 60.019 133

chr1 27536421 AGATGTGTACGGTACCAATGTGTT 24 60.263 GAGATGACGATCACAAGGAAGAGA 24 59.903 264

chr1 27543918 CAGACATGCATCTTCAACAGTCC 23 59.873 CATCTTAACGTGCCAGTGAAAAGT 24 60.024 255

chr1 27546402 GAAGATATTGACCCCTGAGATGCT 24 59.959 ACAGTTACGGTTCTATCTCCTTGT 24 59.229 262

chr1 27547915 CATAAACACTGGGGAGGAGAAGAA 24 60.019 GAGCCACGATACAGAACAATTCAG 24 59.967 222

chr1 27554338 ACCCGAACAATCCAACCCATATTA 24 60.08 AGGCGAGGAGAGTTAAGTAGTTTG 24 60.082 266

chr1 27570233 AACTTAGCTCAACTGGCATGAAAG 24 59.783 GGGGCCTTCTTCTTTCTTCTTTTA 24 58.922 291

chr1 27581719 TGATATGGTCTATGTGTGATGTGCT 25 59.929 TAGTACCCTTCCTTCCTTCACTCA 24 60.203 178

chr1 27582466 TGCTCATACCTCGACGCAAATATA 24 60.023 AAAGGATTCCACCACCACAAATTC 24 59.96 248

chr1 27585158 ATAGTTGTTCATAGGCTCCATCCC 24 59.958 CCACTAATCAAACATCAATTGGACC 25 58.667 210

chr1 27586340 GGTGAAAATGGAGAAGGTGGT 21 58.123 TCCACCATACTATTATAAGTCTCCCC 26 59.042 240

chr1 27586724 TAATAGTATGGTGGAGGAGGTGAA 24 58.216 TATAAATCTCCTCGGCCACAATCG 24 60.561 296

chr1 27586864 CGATTGTGGCCGAGGAGATTTATA 24 60.561 TCTCCTCCTCCACTATCACTATCG 24 60.263 255

chr1 27588318 GTGAGTATCCTTTAGAGAAACATGG 25 57.222 CAACCACGTCTTTCTTTCAACTCT 24 59.667 298

chr1 27594490 TTATAAATGTATGGAGGTGGCGGT 24 59.897 CCATTAGAGGAAGTTGGAGGGAAA 24 60.018 300

chr1 27599973 CCTTACACATCTCTTACCTTTGCC 24 59.364 CAGTATTTGGTGTTTGGATTGGCT 24 60.021 106

chr1 27607469 ATCATTGTGGGAAAGATCTGGGAA 24 60.017 CATGCAAAACCAAGAAGATAACCCT 25 60.047 274

chr1 27609885 CTTTTGAGGTTCTTCGAGGTCCTA 24 60.021 TCTTGCCGGTAGATGACTTAGAAG 24 59.902 118

chr1 27610505 TGATGTCTCCAAATGTGAAGGACT 24 59.958 GTTATGATGATGGGGTGGGGAATA 24 59.956 254

chr1 27636809 TCATTGTCATCATCATCGCCTAGA 24 59.718 GCCTACTGCCTTCCTACATTTTCT 24 60.627 155

chr1 27641634 TCTCCCAAGAACCCAAATGTAACA 24 60.141 CCGTTCAACACCTAACCATCTTTC 24 60.083 215

chr1 27643802 GTTTCCATCTCCCCACTTAGTCTT 24 60.019 ATACAAACACAATGATCCAACGCG 24 60.672 285

chr1 27647090 CATTTTGACAAGTGTGCATCCGAA 24 60.792 ACAATCTGACTGCTCTTCCCTATC 24 59.899 232

chr1 27660476 ATGCGCATATGTGTTATTGAGTCC 24 59.786 GCAAGTGTGAAGGAAAGTATGGAC 24 60.083 191

chr1 27661345 CCAATAACTCACATGCACACTGTT 24 60.023 GGTGTAAGTTGGGGTGAAAGATTG 24 60.022 269

chr1 27665099 AAACTGGTCGTCTGAGTCTGTTAA 24 59.901 CCTAGTTCGTCTTAAGAATGTCGC 24 59.5 235

chr1 27665348 AGACGAACTAGGAATTTCACACGT 24 60.262 TGGCAGGAAATGAGTTAAAAGACG 24 59.785 253

chr1 27670614 CACTTCGCTAGCACAATAAAAGGT 24 59.846 ACTGCTCCCCTCACTACAAATTTA 24 59.711 271

chr1 27678636 ACCAGAATCTTCCTCTTTTCTCCC 24 60.019 GGGCAGATTCAACACAATTCATGA 24 60.082 218

chr1 27679238 TTACGTCTCGAGGGAATGTCAAAT 24 60.082 TTTGGATTGCTTACTCTCTTTGCC 24 59.782 147

chr1 27680382 ACTAGCCATCCTGACACACAAATA 24 59.775 GGATGGATGTTCTGTTAAGTTTGTC 25 58.388 282

chr1 27681592 AGCTACACCCATAACCCAAATCTT 24 60.017 TTGGGGATTTGAAATGGATTGTGG 24 60.02 152

chr1 27688131 ATCCAACCAACCAACTTCGATTTG 24 60.262 TGTTCACATCTCCCAACGTCTATT 24 60.021 299

chr1 27702039 GTCATCACATCAAGCCTACACATG 24 59.965 CCTCACTACACCACAAACGTATGA 24 60.56 229

chr1 27707577 AGAAAAGAATGATTGAGAAGCCGC 24 60.143 GGACAAGAAGAGCAGAGAAGTAGT 24 59.78 256

chr1 27733172 AACTTAACTGCTCCCTTAGATGCA 24 60.019 CACGGTTTAAGTATGAATGGAGCA 24 59.366 235

chr1 27735644 TCGACAGATAGGTTGACATTCCAT 24 59.594 CTTTCGAACTTTTAAAGGAAGGGAG 25 58.331 111

chr1 27739721 TAGCTTGGGTTTATTAAGTCCGGG 24 60.384 CACTTTGAAATGGGTCGAACAAGA 24 59.964 234

chr1 27743718 TAAGCCTATAACTCCTCCTTCTGC 24 59.411 GCTACTGACTTCAACCAAACCAAA 24 59.903 299

chr1 27755437 TCGAAATCAGAACAACCCTACCTT 24 59.959 ATCGAAGGTAGCAAACAACAGTTC 24 59.787 188

chr1 27766857 CATGGAAAAGGGGAAACAAAGTCT 24 59.655 GTTTTCCTCTGTATATCAACTCTCG 25 57.313 213

chr1 27769901 CCGCAACAGACTAAAGATCATCAC 24 59.967 GAATTCCACTTCGACTTCAACAG 23 57.976 219

chr1 27778824 GCGAGTACACTAAATTCCATTGCA 24 59.906 GTCTTGATCGAGGTTGTATTAGCC 24 59.254 245

chr1 27778942 AGTGATCTAGCTTTCTGTTGACCT 24 59.47 AATGGATAGAGAGAGAGAGGAGGG 24 59.955 281

chr1 27781087 CAAAAGCTTAAGATGGTAGACGTGT 25 59.591 GGGCTAGAGTTGAACTTTTAAAACC 25 58.555 284

chr1 27785474 ATGATTATTAGTCAGGTGGAGGCC 24 59.958 GGGATTAGCTCGAGAACTCAATCT 24 59.961 243

chr1 27785607 AGATTGAGTTCTCGAGCTAATCCC 24 59.961 ATTACCCTGAGATGTCCTGGAATG 24 59.896 209

chr1 27786203 TTGTTCACCCCTACTTTACACACA 24 60.08 TGAGTGTAAGGTGACCAAACTTGA 24 60.081 295

chr1 27819867 CCACCACGCGAAAATACTGAAATA 24 59.906 ATGGCCCTTTATTCAAGTCTTGGA 24 60.264 220

chr1 27825411 GGAAAGGTTTGATCCCCTAAATTCT 25 59.042 TCCATATCCATCCAACCTTGAGAT 24 59.084 260

chr1 27825611 GCATGGGAGTAGTTGAGCTTCATA 24 60.443 AATTCTAAACCCACTCCCATCCAT 24 59.767 166

chr1 27826809 TGCATAATAGTCACATAAGGGCGA 24 59.96 GGTAGCCAATTACTTAGCCAATGG 24 59.722 256

chr1 27830078 AGTAACAGGGAGAAAGGTGTCAAA 24 59.835 CACTTAAATCCTCAAGCTTTGCCA 24 60.022 278

chr1 27835174 AACTGGACTGACAAGATTTCATCC 24 58.994 GTTTGGAAATTCAGGGTCTTGAGG 24 60.022 255

chr1 27838572 TGGTAGAACGAGGATAGCAAAACA 24 60.021 AAGTTTGGCTCTCGAGAAATATGC 24 59.666 236

chr1 27839526 TATCGATGGGCCCACAAATATTCT 24 59.957 TTTTCTTTCCCTGTCTTCAATGGC 24 59.961 242

chr1 27848539 GTAATCTCGATCGAAAATGTCCCG 24 59.852 TTTGCAAGCTCTCATTTCACTCTC 24 59.785 281

chr1 27857268 TCTCTCTCTCTCCCTCAATCTTGT 24 60.018 AACTTACCAAGAACTCCGATTCCA 24 59.959 227

chr1 27876817 AGGCAAACAACGTGTAGAATATGC 24 60.143 CTGAAGAATTGCTGAACCACAAGT 24 59.963 271

chr1 27879825 TTCAGAAGCTACAGTCTCCTCTTG 24 59.78 GTGGTGTGAAGCTGACAGAATTAG 24 59.847 260

chr1 27886834 TTTGAGAGTACTGAGGAGAGAGGT 24 59.956 CATCTCCTTCTCCTTCTCCCTTTC 24 60.142 122

chr1 27937390 GGTACATTGACAACTTTTGCCCAT 24 60.262 CCTTCCTCCATCCTATTATACGTGA 25 59.285 204

chr1 28003264 AAGCATATAACACCATCTTGCCCA 24 60.631 GAGTTGTTTGTGGACCTCTTTCAG 24 59.964 155

chr1 28032080 TGAAGTTAAAAGAGGAGGAGAGGG 24 59.467 GCTTTTCAAGTACCGACACCTTTT 24 60.202 237

chr1 28035044 TTAAAGAGTGAACTGTGAGGGTGC 24 60.982 GGACAACTGGACAAGATTCGTTTT 24 59.964 242

chr1 28055478 GCCTCCATTCTATGCTTTTCCTTC 24 59.963 TGCCTGGCTACTCACTTTAAAACT 24 60.446 298

chr1 28057153 AGGATCTGAAAACTGGATGAGCTT 24 60.019 TATGGCAGGAAAGTCTTACGAACA 24 60.021 234

chr1 28094982 TCCTATACCAAATGCAACCTCCAA 24 60.018 GACGATATGGAGATTTGGTGTTGT 24 59.124 207

chr1 28112944 CATCGAACAACTTGGGATAACGAG 24 59.967 AGGAATTGCACTAGTGAAGGTCAT 24 60.019 108

chr1 28114330 GTCCATGACTTTTCCATAGCGTTT 24 59.844 GAAACGTTTTGATAGCGCTTTTCC 24 59.912 162

chr1 28127516 GTGTGCTTGAGACATCTTGGATTC 24 60.143 AGCATGTTTGGTTACCGAGAAATG 24 60.083 159

chr1 28130883 TCAATCAAGAGAGAGGGAGTTTGA 24 59.159 TGTCTAGATTTAGCACACGATCGT 24 59.904 253

chr1 28131433 GGGGAGAGGGTTTGATTATTTTGG 24 59.594 CTAGAAAGCACACGATCCTTTTGA 24 59.309 183

chr1 28133548 TCTCAACAAAGGGAAATGAAAGGC 24 59.961 ACTTCCATTGAAACCCATCTCTCA 24 59.957 273

chr1 28152450 TCTAGGCACCCATTTTACATTCGG 24 60.926 TCACAATAGCCAATGGATGTCA 22 57.766 300

chr1 28162167 ATCACCCTGTTCTCTCTTCTTCTT 24 59.156 GGTTACCCAATATCCAAACGGTCT 24 60.627 204

chr1 28162405 TGACCGTCTACCAAATATAGCCAA 24 59.594 CACGAACGTTTAGATTTGACTTGC 24 59.393 145

chr1 28164954 ACAACACAAGATCTCACCTTCCTT 24 60.141 CCTAACATGACCGTACCTCTCAAT 24 59.9 212

chr1 28165561 CGCATAGGTAAGGTGATGTACGTA 24 60.024 TTTATAGGCCTACAGAAGCTAGCG 24 60.022 239

chr1 28168842 CCAAACAACGAGTCCAGTCAAATT 24 60.202 GGTTTCACAAATACAAGGGAGTTCA 25 59.699 224

chr1 28180756 ACGTCTTCTTGCCACAATGTTTAG 24 60.024 TGGAAATGAAATGTGAGTCCCTTAG 25 59.054 204

chr1 28188288 ACATCTATAAGCACTCACTACCCC 24 59.411 TCTTGCACAACAGGGAATATGAGA 24 60.019 239

chr1 28199849 CCTCTTCCACTCTCTTTAAGCCTT 24 60.02 TCAGAGAAGGCAATTTCCGAGTAT 24 59.838 97

chr1 28203508 TATTGTGGTGATTAGGCGGAAAGA 24 60.081 CCACACATGTTCTTCTTTGGGAC 23 59.995 145

chr1 28209261 GTTATAATGAAACGTTCGCTCCCG 24 60.549 GATGCAAGCTTCACGAACCAATAA 24 60.379 240

chr1 28211591 CCACCTCTAAGTTACTTTGCTTTGT 25 59.469 TTGGTTGTGTATACTTGCAGGTTG 24 59.723 300

chr1 28220332 TCTCTCTCCCCTCTCTTCTTTTCT 24 59.955 CTGAGTTTTCTGAGCTTTGTGGAG 24 60.024 253

chr1 28232392 CTTTTGCTTGTTGTGTGATCAGAC 24 59.263 TACGTCTTTGTACCATTTCCTCGA 24 59.782 150

chr1 28237406 ATTGGACAATTCTCACGCCTAAGA 24 60.323 CTTGAGAGGAGGGAGAAAATGGAA 24 60.019 293

chr1 28240710 TTCAGATGCGGTTGATGAAAAGAC 24 60.083 TCACCATTTTCCAACCTAGCCTTA 24 59.956 257

chr1 28242414 GCAACAGAGTAGGAGATCATGAGT 24 59.901 GGTCTCCCTTTCAATCTTCCTTCT 24 60.019 202

chr1 28242595 GGAGACCCTACAAAAGAGAGTGTT 24 59.959 GGGTTCACGTTATTCTTTTCTACAG 25 58.171 236

chr1 28243262 AAGAAAGAAAAGAGAAGGCCCAAC 24 59.658 ACTTCGTTACCGCTATTGTCTACA 24 59.843 285

chr1 28386379 AGAGAGTGTGGGACCCTATATTCA 24 60.079 TTCAACATCGCAGGTTCAACAAG 23 60.243 170

chr1 28387087 TGAGGTTTGGTCATGTTAGATGAAG 25 59.06 GACCATACGAGTCTATTAGCGATA 24 57.341 268

chr1 28388231 CGTCTAAGGGCCAAGCGATAA 21 60.202 GATGTCAATTAATCACGGGGCATT 24 59.962 300

chr1 28388553 AATGCCCCGTGATTAATTGACATC 24 59.962 TCGTTGAGATTTGGCTACCC 20 57.603 249

chr1 28395673 ACAACTATCAGACAGCTATCAACTC 25 58.431 TGATAGTCACTTGTGATAGGAGTCT 25 58.573 291

chr1 28407998 TGGCCACATTCATTTTAGCTTCTC 24 59.842 ATGACAATGTGATCTCGGACTTGA 24 60.081 221

chr1 28408430 ACAGAGAGTACGTTTCCCTTTCAA 24 59.9 TAATAGTGGAGATTCGCGAGTCAG 24 60.024 284

chr1 28410209 TCATTGCAGTAGGAGAAGAAGGAG 24 59.839 TCACTTTCTGCCCTCTTATCTTCT 24 59.22 257

chr1 28412001 GACATGTTGAGATATCCCGATTAGA 25 58.075 CCATTGCTTTATCCGCTTTGAACA 24 60.618 258

chr1 28414489 TGGGCTACTCTCACTTATGCAAAT 24 60.08 AATCGTTTATGTAGAGAGGGAGCG 24 60.262 227

chr1 28415114 TCTTTGATCGAGATTGGAGTGAGC 24 60.679 TAGCACAAATTCATCAACCGCATG 24 60.675 273

chr1 28420138 CGCCGTTGATTTGAGGATTAAGTT 24 60.143 AGCTGCCAAAACTTACACAGAATC 24 60.023 285

chr1 28420268 CGCCGTTGATTTGAGGATTAAGTT 24 60.143 AGCTGCCAAAACTTACACAGAATC 24 60.023 285

chr1 28420923 CACGCATAAACTTCTATCGCTGTC 24 60.317 GCCCTAGTATGAAGATTTGACCCT 24 59.897 291

chr1 28429432 ACATCAACACAAATCCAACTCCAC 24 59.962 CTCTCATCCTCTCGTTGACTTGAG 24 60.439 299

chr1 28429769 TTGCTTCTTGAGTACCATTGTCCT 24 60.202 TTCGTTGAGGAATTGGGGATGC 22 61.205 200

chr1 28430218 CCAAGGAATAAAGGTGGTATACAAG 25 57.432 CATGGGGCATTTTCTTCAGAGATG 24 60.202 239

chr1 28446313 AACATGCAGACAAACCTTCGAATC 24 60.32 TCTTATTCAATTACCCTTGCACCG 24 59.362 240

chr1 28482424 GCATAACTAGGCGTGTATGTCTCT 24 60.262 ACCTCTGTGATGGAAGGTCATG 22 59.762 279

chr1 28490990 CACTTGACAGAAGGATGCCAAATT 24 60.022 AAGTCAGTTGGCCTACTCAAATCT 24 59.958 90

chr1 28491788 TGGATGGACTTTTGGTTTTCTTGAG 25 59.931 TTTGCAAACTCGGGAGGTAA 20 57.358 289

chr1 28493123 TCTGCTTGATTTCTACGCCTTAGT 24 60.082 ATGAACCATCGACAAGAACCACTA 24 60.021 123

chr1 28513837 CCCCTCTTGCTAAACACTAACTCT 24 60.02 ACACAAAAGACAGAACGAAGAACC 24 59.906 134

chr1 28514075 GGTTCTTCGTTCTGTCTTTTGTGT 24 59.906 AAAGGAAGAAAAGAATCTGGCACG 24 60.023 267

chr1 28515029 GGTCTGGTTTACGTTGTTTGGAAT 24 59.963 AAGATGTTGAAGATCCTCTCCTGC 24 60.384 121

chr1 28517595 GAGCCAAACAACCAAAATTCACAC 24 59.966 AGTGAGGATTGGAAGAAGGAATGA 24 59.462 201

chr1 28524171 TACATTGTAGTTGGGCCTCATTGA 24 60.019 TTGATGTTGGTGGGTCTTCTTTTG 24 59.901 208

chr1 28528967 TTCGCTGTCTTCCTTGATCTGTAA 24 60.021 GAACAACACACATCTAACAAGGGG 24 60.023 118

chr1 28529321 GTTGGTCCAAACGGCTGT 18 58.164 GCTTTACGTTCTTTGTATAACCCTC 25 58.231 300

chr1 28538668 TGCGAACCATATCTACATCCTTCA 24 59.656 TTCTCCCTTCGCTTCTCATTATGT 24 59.838 279

chr1 28539370 TCAGATTTTCACGTGACACTAGGT 24 59.961 GGCATGCTAGTTTTAAGAGATGATC 25 58.103 201

chr1 28540849 AGGTTCAATCCCCATCACTGAATT 24 60.265 CCTTCTCCCCTCATAGCTCATTAC 24 60.021 136

chr1 28550524 AAGATAGGCCAATCGTGACAGTTT 24 60.566 AAATGGTTTGTGTAATGGCAGAGC 24 60.561 177

chr1 28565980 CAGGCTTTTGTGTTCATTCTCCAT 24 60.022 CCTAAATCGAACTCAAACGCACAT 24 60.143 298

chr1 28566887 GGGATGGAGACTGATTTTGTATGC 24 59.723 TGCATTCCAACATTTCTCCGATTC 24 60.142 144

chr1 28569344 ACTCTACAAGAATGAAGAAGCACCT 25 59.987 AAAAGTAAAGCCAACATACGGTGG 24 60.023 123

chr1 28569741 GGTCCTCCCTTGCCTTTATTCATA 24 60.141 GCTTCATTAAACTGGTCCCTTGTT 24 59.719 259

chr1 28577058 ATCGGAAGTCAGGTGCTTTCTATT 24 60.081 TCCATTGGACTTAGACTCGCATAT 24 59.411 213

chr1 28592300 GGTTTTGGGTTTGGATGGATGATT 24 60.019 GAAACGGTGTAAAATAGAGCTGGG 24 59.906 298

chr1 28596662 AGAGGTGAAAGGAGGCTTTGTAAT 24 59.957 ATGTCACTTCAAGCAATTTCCCTG 24 60.022 197

chr1 28601074 TGATGTGATGGAGGATTTATACCC 24 57.873 GATGGAGATGGAGATGGAGATCAC 24 60.082 170

chr1 28605731 CCCGTGATTGGAATGTTTTGGAAT 24 60.323 TACCTATAGAGTTGCAATGACGCC 24 60.501 266

chr1 28613780 CCAGCGTCTCGAGATAGATCATAC 24 60.202 CACTAGGTGGGAAGAGAAAGTTGA 24 59.96 297

chr1 28614548 CTTTTCTTTGGATTCGGGCCTTAT 24 59.598 AGAGAATTGACGAATCTTCCAGCA 24 60.323 281

chr1 28624746 GCTTCGGATTTGTTGGATTGAGAT 24 59.903 GAGTTGGAGAAGGTGGTTTCAATG 24 60.023 169

chr1 28627969 TGGGACCTTTAACGTGTTTAGTGT 24 60.384 TGGGTATGCTGAAATCCACAAGTA 24 60.019 212

chr1 28630837 TTGTCGGAAAGTAGAGAAATGTGC 24 59.549 TGCATGCAACTCTACAGATCATCA 24 60.383 167

chr1 28640791 TTGCGAAATTAGCCATATCTCAGC 24 59.786 ATCGATACCACCCAAAAGTTGAGA 24 60.02 115

chr1 28648884 GATTCCACTGCCACCAATAACAAA 24 60.022 GGGGCTGACAGTTGGATAGAATAA 24 60.141 226

chr1 28660923 AACGTTGTATTTGGAACCTCGAGA 24 60.502 TTGGACTTTCAACATAACCCAAGC 24 59.961 282

chr1 28662841 CTTTCCAACGAGAAGAGTGTTTGG 24 60.261 CGGTGGAAGGAAGCTTAATCAATC 24 59.964 111

chr1 28665896 TCATAAATGACATCCCGTACCCTT 24 59.589 CGATGGTGAATGTTGGAAAGTTGA 24 60.023 276

chr1 28672087 TGGGCCCATTAATAAAGTGAGGAA 24 60.017 GCGGGAATAGATCGGTAAAAGAAG 24 59.552 280

chr1 28679444 AGGCCAAGTAAGAAGGATTGAAGG 24 60.569 TGACTTAAGTAGCTCTGACGTGTT 24 59.722 281

chr1 28680434 TCTTTTACGAGATAGGAAGTGGACC 25 59.873 ACTTGAGGATTAGAAGTAACCCGC 24 60.383 271

chr1 28690224 ATGGTGGGGAGTGTTGAATAAGAA 24 59.956 GGCCATACACTCCAATTTTCTTTTG 25 59.361 161

chr1 28705356 CTTGTTCTTGCATCTTACCTACGT 24 59.07 GCACACATTTCCACATTTGACTATG 25 59.426 232

chr1 28732706 TGTTGTCATAGGGTGCAAGTACAA 24 60.445 GGTGAACCCAAGCTAATGATAGGA 24 60.141 209

chr1 28740178 CCCGACAACACAATCACCAATAAA 24 60.023 GTAACGATGATGTGGGGTTAGAGA 24 59.901 254

chr1 28784896 AGAGAGTGACACGACCATAAGAAA 24 59.477 ATAACCACCACTTTTCTTCGCGTT 24 61.516 300

chr1 28793079 GGAGTGGTTTCAAAATGGAAGTCC 24 60.262 TGACTTCCACTTGTTAGATCGTTG 24 59.011 183

chr1 28793250 CAACGATCTAACAAGTGGAAGTCA 24 59.011 GCTAGTATGAAGACAAGAACCTTAGG 26 59.018 294

chr1 28799525 ACATCAAGGTCCCATTACACATCA 24 60.019 ACAATGTAACACTTCCCTCTAGCT 24 59.467 284

chr1 28815660 ATGAGACAGAAAAGGTGAAGGGTT 24 60.141 CTTGGTCAAGCAAGAGGAAAACTT 24 59.901 255

chr1 28819238 AGCTAGTCATCTGAATACGTTGCA 24 60.142 TTTTCTATCACATCCCCTCCCAAG 24 60.08 262

chr1 28833599 AGTTCCTTGGAAATTCTGAGGTCA 24 59.895 GCTTTTCTCAAATCCCTCAAACCA 24 59.961 176

chr1 28834094 TGCACTGAACTGAATTACGTACAAC 25 60.051 CCATTCGTTAAGGAGTGATGTTGG 24 59.905 281

chr1 28836200 GATGTGGTTTAGGATTTCGACGAC 24 59.967 AGTCCAGTTGTCGCTTCTTAATAGA 25 59.815 299

chr1 28837479 CACTTGAGGGATAACATTTAGCGT 24 59.124 CCAAACAATGTCACTAAACCAGCT 24 59.962 140

chr1 28838903 AGGGCCCGCTAATAATCATCTAAG 24 60.081 TCCTCACATTTATATCCACTGCTGT 25 59.869 282

chr1 28876829 AGGTCCCTAAGTTGAACCTTGTTA 24 59.338 GTGAATTCAAGTGTCCAAGCCTAC 24 60.083 283

chr1 28877369 CCCATGTCAGAAGATCTCCATCAA 24 60.142 TCTCGTGATCATTAAAACCCACTG 24 59.065 283

chr1 28878251 GAAGAGGCTGAAAATCGAACCAAA 24 60.023 CAGTCTTCCGTTTTGTTGGTTTCT 24 60.142 143

chr1 28879339 TGCAGTTGACTAGTCCTCCATTAG 24 59.84 ATAAGGGTAGGGAAGGAAGAGGAA 24 60.015 300

chr1 28882780 TGGGTCTCCTCTTATCACTACCTT 24 60.016 CCGAAAAGATAGTGAAGGAGTTGC 24 59.906 264

chr1 28885977 CAAATGCTAAAATGCCCTCTGTCA 24 60.082 TCCATACTCCCCTTAAGCAACAAA 24 59.956 165

chr1 28887177 TCGTAGAAACTGTGTACTTCCTGT 24 59.418 CATTCCAGAAATCTCATGCTCCAC 24 59.964 216

chr1 28887450 GGAGCATGAGATTTCTGGAATGTG 24 59.964 GTTTCCTTCATCATCGTGGATTCC 24 59.964 177

chr1 28893570 GGAAGAGATAACCAGTGACCACAA 24 60.263 CACTGAAAATTGAGTTCCTAACCGA 25 59.531 219

chr1 28900455 CTGCTGTGTATGTATTTCGTTTCCA 25 59.878 TGGAATGTGATACTTAGGGTTGAGA 25 59.276 297

chr1 28922716 TTAGCATGGGGAGTTCAAATGGAT 24 60.326 CTTAACACTCATTTGACACTGCTTG 25 59.089 142

chr1 28925509 CTATTGCACACCACCACTTAGTTG 24 60.083 CGTTCACTGTTCTGATATGGCATC 24 60.025 223

chr1 28931510 CCAACAGAAGTCTATGGCTATTGA 24 58.325 TCACAACCAGCTACCCATATCAAA 24 60.019 284

chr1 28942187 CTCTAAAGCCATGACACTTGCAAG 24 60.379 ATGGAGAAAATTATGATAGCGGCC 24 59.298 292

chr1 28947969 ATTTCAAAACCGTCAAATGTGGGC 24 61.273 AAACGTAACATTTCACCTTTCCGC 24 60.79 299

chr1 28952668 TCACTTCAATTACCTCCGACTCAA 24 59.718 TTGTTGGCTTGATAGGAATGGAAC 24 59.538 129

chr1 28953174 TCTTCCTAACTCCAAATGCAGTGT 24 60.202 CCTTGCTAGAAAATGTGGTGTTTG 24 59.016 272

chr1 28953963 GTTACTCTTGACACGTTTTGCATG 24 59.329 CGCACCCAAGATTATGAAGATGTT 24 59.664 83

chr1 28955284 GGGTTATGAATTAGATGCCTTATGTGG 27 60.094 CACCCCATGTCCACTTGTTTAA 22 58.773 295

chr1 28958245 TTCCAGTGAAGGGCAAAATAGAGA 24 59.957 GATTGTTGAATGCTCCTACCACAA 24 59.541 197

chr1 28967377 TGTACCTGTCTTGAAGTCCAACAA 24 60.081 CACTTTTGGGCTGCACGTATATTT 24 60.38 233

chr1 28982028 TGAAATGATGGGTTTGCTTCCTTC 24 60.021 GACACGGACGATACACTTTTATGA 24 58.902 290

chr1 28985570 GCGGACAATTCATCACTTCTCTTT 24 59.845 TGTTGACCATGCTAAACGATTCTC 24 59.608 238

chr1 28999599 GAGTCATTTTCATGGTGGCGTTAA 24 60.083 AAGGTGCAGAAGAGTGAAGAAGAA 24 60.142 134

chr1 29011404 GTAAGACAAGTTGGTTCCCCTGAA 24 60.688 AAAGTGTGTTGTTGTTCCTTCTGG 24 60.082 257

chr1 29014768 CTTTTGGAGGACTTTGTGGCTTTT 24 60.383 TGTAACACTCGAGCTTACAGGAAT 24 59.78 274

chr1 29017441 TCCACTCACTCCACATTTTCATCT 24 59.958 TACAGACTCCAAAACCGATGACTT 24 59.96 205

chr1 29019161 TCTGAAAAGTAGTCATGCGTGGTA 24 60.022 GAAAGAGGGTAGGAATGTCTGTGT 24 60.02 142

chr1 29021703 ACAAAGTCCAATCCACACATCAAG 24 59.721 AGGTGTGTATCAAAGGGTATCGAG 24 59.9 184

chr1 29040610 TGGTGCATCTTCCGTTATTAACTG 24 59.366 ACGGTCAAATAGCTAGGTAGATAC 24 57.428 291

chr1 29043334 ACACCTCCAATCTTGTCTTCTCTG 24 60.263 GACTTTATGTTGAGTTGGGTGTGA 24 59.18 207

chr1 29046966 CGATTTGCTCCTTTCTTGTTCACT 24 60.024 CGCAATGATCCAACACGTTTATGT 24 60.672 296

chr1 29048714 TGTGATAAGCAGAAAACATGGTCG 24 59.846 TAGCCTCGGGACCTTAGTTTATTG 24 59.9 228

chr1 29057205 TGTTTCCCTTGCCTTTATACCTCA 24 59.956 CCGAGATTGAAACTTCCCCAAAAT 24 59.779 270

chr1 29059282 ACGAGAAAAGTTCATAAGTCCCCA 24 59.959 TCCTTTCCTACACAATCACACGAT 24 60.021 107

chr1 29061437 TCGCCAACTATCCGAGAGAATAAG 24 60.023 GCCTTGATGCGACATAACTTACTC 24 60.026 294

chr1 29070774 TCACTAACATTCGAATATGCTTGGC 25 59.992 TATTGGACTAAATGGGTGCTTGTG 24 59.295 254

chr1 29081625 TTGGGCTTATTGGGAGAGAGAAAA 24 59.956 TTCCGATGACAACAATCTCAGAGG 24 60.622 135

chr1 29084835 GTGGCAGATTCGAAAATTGGGTTA 24 60.082 GGCTAAACTCTAACCTTCTCCCAA 24 60.02 213

chr1 29089851 CTGTGCATGCTGTCTAACTCTAGA 24 60.142 CAACGAGACAACAGCCTAACAAAT 24 60.024 264

chr1 29095868 CCTTTCGACGCTGGCAATTAATAA 24 60.202 CCACAAACTGATTTCCACCCTTAC 24 60.022 206

chr1 29096546 TGTGTTTAGCTTCGGTGATTCATG 24 59.846 TCTTCCCCAAATCCTGTTCATCTT 24 59.955 226

chr1 29112735 AGGAAATTACCAAACGGAGAACTC 24 58.998 TGGAATGTAGAAAAGAGGGATGCA 24 60.019 272

chr1 29113359 CAGTATGTCCTTGTTTCGCTTTGT 24 60.024 ATCGGAAGGATCAAAGAACACTGA 24 60.02 271

chr1 29115234 GTTGAGACAAGTTGGTAGGAGGAT 24 60.02 CCATCATTCTCGGTTATAGTGGGT 24 59.96 259

chr1 29138370 CCAAATGCAGCTTACATCATCACA 24 60.142 CAATTTGCGTGGATCTGCTCTATT 24 59.964 262

chr1 29147403 CTCACACATTGTCAAACCTCATGT 24 59.723 GATAATTGCGCAATCCTTCCTTCA 24 59.963 160

chr1 29161320 AGGGAGGAGAACGTTGGAAATTAA 24 59.958 GCGAGATGGCTAGGTGATTTACTA 24 60.022 194

chr1 29162307 CACAAGTTCAACTCCACGAATCAA 24 59.965 CGTTTGCAACAGTAGCTTTGAATG 24 59.853 151

chr1 29173440 GTCTCGATTAATTGACATCTAGCTCAC 27 59.886 CATTGTGAATCTACCGAACGTCAA 24 59.612 271

chr1 29199316 CTTTAAATTTGGAGTGTGCGAGGT 24 60.023 ATACCATTGTTCAATCCCCACAGT 24 60.264 295

chr1 29222939 AAAATGAGTGTGATGTGAGCGATG 24 60.143 TAGTATAGGGACAAGGTTGGGTAC 24 58.789 254

chr1 29229803 CCGTCTTACTTACCCTACCGTTAG 24 59.964 GTGTCAACTAAGGCCGATTTTGTA 24 59.547 237

chr1 29232196 GGAATGGAGGAAGTGCTTTTGAAA 24 59.961 GGGTCAACTTTGGATTGTTCTGTT 24 59.901 191

chr1 29232983 CTTTTGAAACGTGGAATCAGTGGA 24 59.964 TCACGCATGTCCTAATGATTGTTG 24 59.905 163

chr1 29233334 TGACTAATGTGGACAACTTGGTGA 24 60.142 GTGAACAACTACCTAACTCATCTCA 25 58.311 206

chr1 29236354 AACCTTTATGTCAGTTTCTCCCGA 24 59.959 CCGATGCCTGGTTTACTTCTTTTA 24 59.302 284

chr1 29236486 AACCTTTATGTCAGTTTCTCCCGA 24 59.959 CCGATGCCTGGTTTACTTCTTTTA 24 59.302 284

chr1 29284910 CCAAACAGCTGCTTTCATACCAAT 24 60.322 GGGGAATGATGTAATAGGAATAGGGA 26 59.553 264

chr1 29307486 ATGAGAGGCCCTGAAGCATAATTC 24 60.749 GATCAGACATTTGGAGTTGAAGCT 24 59.301 199

chr1 29330019 CGAGCCAACAAAGTGAAAAGAGAG 24 60.554 GTTCGGCGTCTTATGGCTTATTTT 24 60.202 217

chr1 29341480 AACCAAAATCGAGCATTAGAACCC 24 59.842 GAATCTGATCTTCATTCCTTCGTGT 25 59.185 293

chr1 29347036 TCTCAAGCGAAAAGTTAGCAATGC 24 60.614 TCCAAGGCCTAATGTACGTTAACA 24 60.02 166

chr1 29352979 ACATTAGGGGAGTTTGCTGAGATT 24 60.018 TTCACGTGCCTTGACATAGAGTTA 24 60.022 207

chr1 29354688 GCAATCCACTAAAGCACCTAAACA 24 59.782 GATCACCATCTTCCACTTGTTTGA 24 59.238 141

chr1 29358764 CAATTTGGCCGATTCTGTAATTGC 24 59.732 TCCTTAGTTTTGGGAGTTTGAGGT 24 59.833 240

chr1 29359513 CATCGCCTAGGAATCAGACACATA 24 60.022 TCATTGGTTCTGATGTAGCTTCGA 24 60.082 263

chr1 29364730 GTTTCACAAGGCTTTTGATGTTGC 24 60.26 ACGTTTTGAGGGGATCTTTGAAAG 24 59.722 286

chr1 29365108 GGCTCACAGTCAATCACCAATAAA 24 59.541 GAAGTGGAGATTCGTACCTCTAAT 24 57.606 290

chr1 29371980 CCTCTCTTTTCACCTCATAAACTCA 25 58.532 TGCATGAACAACTCTAGCGTAAAG 24 59.611 174

chr1 29372086 CCTCTCTTTTCACCTCATAAACTCA 25 58.532 AGTAAATGCATGAACAACTCTAGCG 25 59.936 180

chr1 29388968 ATCGTTTAATCCATGTGTTGCCTC 24 59.904 AGGGACGGGAAATCACAATTAGTT 24 60.264 295

chr1 29397141 CAAATTTACGTGGAAACCCGAGTA 24 59.548 TGATTTGTGTGTTAGCAAGTTGGG 24 60.202 232

chr1 29405500 GTTAACTTGATGGTTTCCTGAGCC 24 60.083 CTGTGTCCTTGAGTTCTCAGGTAA 24 59.961 122

chr1 29426203 AGTAGACGATGAAGGTTGGTTGAA 24 59.96 TGGACCATGACAGATTGTTCTATGA 25 59.81 218

chr1 29428080 GTCCTGATTGACCCTTCATCTGTT 24 60.566 AGCGGCATTCCATGTATAAGAGAA 24 60.202 294

chr1 29431712 TAAACAACAAGGGACGACATTTGG 24 59.963 AATATTCACTTGCTTTCAGTCGCC 24 60.143 235

chr1 29436967 GCTCGAACCATTTCATAACAAGCT 24 60.143 TAAGGTCTCAGAGCCAAGTTTGTT 24 60.141 255

chr1 29448277 CGGAGAAGGAATGACAATTGATGA 24 59.122 ATTAGACGGTTGTATCAAGTGGCT 24 60.081 218

chr1 29449013 GCATACTATCTCTTGCCCTACACA 24 59.961 AAAGTGATCAGGAGGGTCTTTCTC 24 60.02 193

chr1 29450468 AGTTGTGAAAAGAGAGGTAAGGCT 24 59.897 CACGAACCATAAATGTTTTGCCGA 24 60.849 282

chr1 29464599 GTTGTTCGTGTTCCTTATTTCGCT 24 60.319 GGCTCCTTGTTCAAGATCCTTAGT 24 60.324 230

chr1 29467944 GACATGCCAAATAGCCTAAGTGTG 24 60.202 AAGTGACAAGGACAAGGCAAGTTA 24 60.628 130

chr1 29492436 AGCCCACTAATTCCAACAGTAACA 24 60.203 TAAGTTTCTCTACAGCCCTCAACC 24 60.02 214

chr1 29492748 ACAAAATACAGTCACCTCCTTTCG 24 59.245 TTAGGTCTCCAACAGTCAACAGTC 24 60.202 204

chr1 29493247 TAGGTCGTGTCCATTGATGCTTAA 24 60.081 TGTGATCTAACCCCTGCATTGTTA 24 60.019 290

chr1 29498717 GCTGATTTTGATAATCTTCGGGTTG 25 59.026 CTCTCCATAGCCTTTCCAATATAAC 25 57.086 265

chr1 29517182 GGTACATGAGGGTTATACAAAGAGC 25 59.241 GAGTCTCTTGGTTGAAATGATGCA 24 59.543 226

chr1 29526622 TAGGCTCTGAGTTCATTTGTGTCC 24 60.564 GATGCTTAATTCCTTTGTTCCCCA 24 59.534 249

chr1 29533121 GGTAAACCCAAAGAAGCACCATC 23 60.057 GTGGAGTACGGTAACCCATGTATT 24 60.142 270

chr1 29535614 AGAGTCCAAGTCAATTAGCACACT 24 59.959 GAGGAGAGTTGGATGAGCATTTTG 24 59.904 241

chr1 29548529 CCAATTTGAGCAACACACCTTACA 24 60.202 TATACATGTACACGAGCACACACA 24 60.083 279

chr1 29549198 CTGAGGTTGTTTCCACTTCTTTCC 24 59.963 TGAGAGCATAGAGGGCATGTTAAC 24 60.443 217

chr1 29555677 TTATGTTCCCAGCTCTCCATTCAA 24 60.019 TAGGTGACATGCAAATGAGGGTAT 24 59.834 255

chr1 29556611 TATGATGGATTTGGGTGGTTCAGT 24 60.018 CATGAGCAACAAGAGAATGATCCC 24 59.964 213

chr1 29571632 CCAAATAAGATTGTCAGGGAGGGA 24 60.08 AACTTTTCAGCGTGTTGGTTCTAG 24 59.965 204

chr1 29583166 CATGACTAGTGGTGCCTTGC 20 58.91 CCACATATTAGGACATTTCAAGCTAGG 27 59.826 300

chr1 29584026 TGATCCTTATTTCCGTTGTGCATG 24 59.904 GCAACCACATAGCCACTAGAGTTA 24 60.383 269

chr1 29586805 AGTCTCACAACTCGGTTCTTTACA 24 59.901 GAGAGATTCGTCCTTACCCTTACC 24 59.962 253

chr1 29597326 ATGGAGTAGTAACAAGCAGTCTGG 24 60.081 CAGCTTGATTGTCACCCATCATTT 24 60.082 141

chr1 29605490 GCAACCCGAGCCTAATTAAAAGAA 24 59.843 TACACCACTAAAAGCTGATTTGCC 24 59.782 215

chr1 29611120 GAAGAAAACTCCTCACTGCCAAAG 24 60.261 AGGGCCTTTGTTTCTTATCCATCT 24 60.017 229

chr1 29616498 ACATAATCTTGGTTCGTGGAGGAT 24 59.836 TCAAGCAATCAATTAATCCTCGGC 24 59.963 246

chr1 29616613 GCCGAGGATTAATTGATTGCTTGA 24 59.963 TTGACTCACCAACTTTAACGTGTG 24 59.906 194

chr1 29628894 TGACCGTACTTCCTGAGAAAAGAA 24 59.657 GTCGTAGGGGATGTAGGAGAATG 23 59.744 280

chr1 29644325 TGGAGTTAGGAGATCAAGGAAGAA 24 58.658 GGTTTCCTTGACTCATTATGTGCA 24 59.541 284

chr1 29644427 GCACATAATGAGTCAAGGAAACCA 24 59.541 CACCAGCATCCCTTGAAGAATTTT 24 60.021 141

chr1 29646446 CCGGTATCTCTCTCCAATTCTGTT 24 59.9 CAGAAGGCCTTGAAATTTTGTTGG 24 59.487 268

chr1 29646773 TCAATTGAGCCCTTGGAGATAGTT 24 59.772 AAAGGATATGGAGAGATTGCAGCA 24 60.141 259

chr1 29649575 GATTTCTTTCTCCGTTCCATCCAC 24 59.905 AGGAGCTGGGTAAGTTTTATGTGT 24 59.957 244

chr1 29661178 TTGGGGAGATTTACACGACAAGAT 24 60.02 ATCGTCGTAGTCTTCTTGATCGTT 24 59.905 247

chr1 29671466 ATTTGATGTGGAATTTGCCCTGAG 24 60.081 TGGGTTAGTTAGTTACAGCTGTACT 25 59.223 194

chr1 29671632 CCTTGGATGGAACTTTGTCAAAAC 24 58.95 GCTGCTCTGATACCACTTTGATGT 24 61.16 290

chr1 29689938 GTCACACACTATTCCAATCCTCAA 24 58.754 GATCAGTGAGCCATTCTACGAGG 23 60.552 269

chr1 29690768 TGAGACTAAAGCTTTCCCCGTTTA 24 59.959 TTGAATCTCCTCGTGTACCATGTT 24 60.021 209

chr1 29696624 CTTCAACTTCATGTAACTACCGCC 24 59.907 GAAGTTAAAGGGAAGGAGGAGGAG 24 60.081 150

chr1 29725759 AATCCAAACCGTCCAGTAGATCAT 24 59.836 TAAGGTTGGAGTGTTAGAGAAGCC 24 60.02 217

chr1 29726274 AGCGTTCCTGAGATTGAGTTTAGA 24 59.779 ACGAATACAAAGGGGATAGTTGCA 24 60.324 296

chr1 29748747 TTTCTTTCACTTGTCGATTTCCCG 24 60.024 ACGTTCTCTGTTTCTCGTTCTCAA 24 60.44 281

chr1 29754399 TACCCCAAACCTTAACGATCTCAT 24 59.528 ATATGTGTGTGTGTGTGGAGAGAG 24 60.322 252

chr1 29756130 CAAGTTGACCAAGACACCTAACAG 24 59.727 TCCCACCCTTTCATTCCTACTTTT 24 59.893 180

chr1 29775736 AATTCTTATCTCATGCATGCGTCG 24 60.084 CTTTCTCATCCGCCTTGTTTCTTT 24 60.023 209

chr1 29779766 GTGCCAAGTACCTAGGTCAAAATG 24 59.844 CATGTGTTGTGAAATGATTGCACC 24 59.849 294

chr1 29837833 GTCATCTCGTTGCAAATCCTTCAA 24 60.083 GAGTGATTTGGAATCTTTGGCGAA 24 60.083 181

chr1 29839347 CACGCTTCTAGTTATGCAAATGACT 25 59.936 TCGTTGGGGTCTTTATTCATGTCT 24 60.02 140

chr1 29846247 TTGGCGTGAAGAATAGGGAAAGAA 24 60.506 GCAACACCAAGAAGAAGAAGAAGAA 25 59.933 178

chr1 29846442 TCTTCTTCTTCTTCTTGGTGTTGC 24 59.424 CTTGGCACTGTCACATCAAATTCA 24 60.262 296

chr1 29847750 CCAACCACCATCTTTAATACTAAGC 25 57.972 CTCGATTGAAGTGGACTATGAGGT 24 59.901 155

chr1 29848537 CAGATGGACAACAACATATCAGGC 24 59.964 CTAAGGTAGAGTTGGGGATTCAGG 24 59.898 285

chr1 29849821 CGAGTTTGGAGCTTAATAACTTCCA 25 59.358 TGTAACACCCCATATACTAAGGTAC 25 57.398 245

chr1 29850336 GCGTGATTCATGACTCATCCATTT 24 59.964 TGCCTTATCAAATCTCCTGGTCAA 24 60.019 83

chr1 29851365 TGAACTACTTGAATGAGGCAGTGA 24 59.96 TAATGCCCCAATTAAACATGACGG 24 59.901 88

chr1 29858178 TTAAGAGATGATGGGGCCTATTGG 24 59.957 GCTCTAGTGCAAACTTATAGACTACC 26 59.081 270

chr1 29863456 GACGAGGTGATTCAAGATGTTTCG 24 60.202 CAAGAAGATGTCACACTCCAAACC 24 60.024 292

chr1 29867295 TCTCACTCTTAAACCCAAGCCATT 24 60.203 CAACGTTGCTCCTCTTTTCTGTTT 24 60.439 249

chr1 29871917 CCCTTCAATATAACCCTCCTCCAA 24 59.584 GCCGTCACTCTTCATTTTGGTATT 24 59.844 273

chr1 29881062 TGCAAGTGAGCCTAGAAAGTACTT 24 59.96 TCATTTGACTAGTTTTGGGCGAAC 24 60.024 172

chr1 29940340 GCTATGCATTTAGTTTTGTACGTGG 25 59.263 TGGATTCGAGTTGATTAGGATAGGT 25 59.161 283

chr1 29960897 ATGACAAAGTGGTAGGGCACTATT 24 60.018 ATCAAACTCGTTTCCTCTCTACCC 24 60.082 228

chr1 30160165 CTCTTGCAACAATGTCAGAGAAGA 24 59.246 GGCCATGATGAAGACAAACAACAA 24 60.501 267

chr1 30161059 GAAAAGGATGACTAGTTCTCCCC 23 58.228 CGGTAACAACGATTAGTCACCAAC 24 60.143 148

chr1 30161380 TTAATCAGCAGCCAAACCTAAACC 24 59.78 GTGTAACCAAAGCCTTTTCTTCCA 24 59.901 96

chr1 30180207 TGTCTGGCTCTATGGAAACTTTCA 24 59.958 GCGATCAGACAAAAGAGCTCATTT 24 60.143 207

chr1 30180532 ATCCTCTAAAGTCAGAAACCACCC 24 60.019 ATTAAATGTCTATGTGCGCACGTG 24 60.494 144

chr1 30187224 TCTCCCTCCCTCCCTCTTTT 20 59.2 TGATTAGGTGCATGGGAGC 19 57.193 300

chr1 30208653 CCATTTAGCATGCAATTAATAGGCC 25 59.129 TGGCCTATTAATGTGGTTGTGA 22 57.691 242

chr1 30281905 AAAATTGTAGCCCGGATGTAGTCA 24 60.324 CTCGGGCAATTCAAAGGGTAAAAT 24 60.082 297

chr1 30358428 CATTAGTGCCTCGTTAGTTGTTGG 24 60.143 GTTCGATTCGCAATGGAGCTTTAT 24 60.261 142

chr1 30447147 TGGTAGGGCTTTTATGAGTGAACT 24 59.711 AAAGACGAAATATGACCCTTGCCT 24 60.568 274

chr1 30627003 TGGTGGAAACTGAAATGCAAGA 22 58.705 GCTCCATTTTCGTAATGTGGGATT 24 59.902 242

chr1 30629276 TACGGATACAACTTGCTTCGTAGT 24 59.843 CTTGTAGTGATAGGCAGGAGTTCA 24 59.84 255

chr1 30634560 CTTTGTGAGTTAGGTGTCTACATC 24 57.047 GCGCACATAATATATTTGGACAACG 25 59.436 180

chr1 30637805 AGTTTCACTCTAAGCATGGAAGTTC 25 59.297 GGTGGTTCCTTGGATGATAGATTC 24 58.933 296

chr1 30642744 GCCAGTTGAGAGGGACCTATTAAA 24 60.081 TCATCAAATCATAGCACGACAAGC 24 59.966 274

chr1 30643570 TGTAAGAGCAGACATCAAATGAAGG 25 59.355 GAACTGCTTAAAAGACACCAACGA 24 59.965 237

chr1 30647238 GAACGAGTGTCATACAAATCCACA 24 59.311 ATTGGCTTCATCAAAAGAATGGGG 24 60.081 271

chr1 30668280 CACTAGCAATGCACCCTTAAAGAA 24 59.541 CATTGTGAACTTTGATTACCCGAGT 25 59.819 194

chr1 30883980 AAATGTTGTGTGTATTGTGTGGGC 24 60.739 TTAGGCGTGTACCAAGTGTATCAA 24 60.021 257

chr1 30886077 GGGAGTATTATGTTTGGCTAATCTC 25 57.326 ACCATAACGCCCTTAATCAAGTGA 24 60.324 170

chr1 30905488 TCCCAATGCCACAACCTAGTAAAT 24 60.264 ACACATTCACCTCTGTACGAAAGA 24 59.961 274

chr1 30905811 CGCGGCAGATTAGGTTTTAGAAAA 24 60.143 GTTGGCATGATCTTCTTATTCGCA 24 59.964 262

chr1 30911972 ATATCCTAACCTCACTTGCCATGG 24 60.203 CCTATATTCATGGGTATATTCGGGT 25 57.625 175

chr1 30917692 GTTACTATTCCGTGGTCCAGTCTT 24 60.082 TGTAGGGACATACTGAGATCAATG 24 57.589 198

chr1 30918692 TTGTACCCACTATGAACCTTGTGT 24 59.897 CCACTAGTAGCCTTTGAGTCATCC 24 60.442 300

chr1 30919239 GAAATGGCCAGAGAGAAGGTTTTC 24 60.083 TCACATTAAGAACTCAGCTCAACC 24 59.003 260

chr1 30920139 ATTGTTGATTTGCTCCTCAGTTGG 24 60.022 GACATGAAAACAAGCGATCAGTCT 24 59.847 299

chr1 30965712 CGTTGGCTGCTTTGTGATAAGATG 24 60.962 TCAAATCATGCCTCTCAGAACTCA 24 60.02 208

chr1 30970834 CCCTAGTAGTTGCAGTGAAAGGTA 24 59.778 CCCACGGACACTAAAATTGAGAAG 24 59.846 97

chr1 30971585 CCCAACAAACAAATAGGCTCAAGT 24 59.961 AGCTCCAACACTGATCTTCTCTTT 24 59.959 230

chr1 30972575 AACTCGCTCTTCCATATCTCTTCC 24 59.961 GCATGTGGTCAATTAATAGCGTGT 24 60.202 296

chr1 30976613 TAATTTGGCTTGTGTGCTTGAGAG 24 60.023 TGGCCACTGATACATGTTCACTAA 24 60.02 231

chr1 30979937 AGGTCTCGTTTGGTTTGTGATACT 24 60.202 TCAAATGCACAGCCAAACACAA 22 60.094 199

chr1 31006523 GTGTGTTCTTGAATGTGATGAGGG 24 60.083 TCCAGCTTCAACACTCCTTATTCC 24 60.566 199

chr1 31036411 GCCAGAGATTCTTTCCGTTATTGG 24 59.964 AAGAACCTCATGAAATTTGCCGAG 24 60.083 297

chr1 31039935 GAAGCTAGAAGATGCACAAATCCC 24 59.964 GACCTGATAGTGTGGCTTCTTACA 24 60.082 100

chr1 31043227 GTGGGTTCTCTCTCTACTGTCTTT 24 59.475 TGTGTTCATCTTCTCCTTCACCTT 24 59.897 191

chr1 31146112 TTTACCTCTTGATGTGTGGGTTGA 24 60.141 GTGCACATGATTCATAACGTTTCG 24 59.508 201

chr1 31151617 TGGTTCGTTCATGTGATTTCTGAC 24 59.787 ACCTATTAGCTTATGGTCGTGCAA 24 60.142 163

chr1 31153387 GTTGCCTTTAACCTTATGTGCACA 24 60.262 TGTGAAGTGAAGTGTTACTCTGCT 24 60.142 297

chr1 31162385 TACGGCAAATTGAGATAGGCATTC 24 59.485 GGGTCTCATTGATATCTACGTCCC 24 60.082 292

chr1 31163041 ATAGGCCTCAATAGCTCTCAAGTC 24 59.717 CTTCTTCGTTGTGAGTGCCATTAG 24 60.143 150

chr1 31165446 TAGCACCAAAGTCTAAGATCCAGG 24 59.838 AGGTGTGCTTATATGGGTGAGTTT 24 60.018 172

chr1 31180377 TCCTAGCTCCTTTGAATTCACACA 24 59.958 CTGCCAATTGTTTGTGAAGAAAGG 24 59.491 176

chr1 31184758 ATACGATGACAAAAGGAGGACAGT 24 59.777 TGATGTCGAGATTAAGGGGTTCTC 24 59.9 210

chr1 31187229 CTTCGATTTGGGTGATTCAAGAGG 24 59.905 CATAAAAGCGGGAGTCATGGAAAG 24 60.202 124

chr1 31204770 TCGGTCCCTTTCTCTTTCAGATTT 24 59.958 TGTAATGCATCGAGAAATAGGGCA 24 60.444 243

chr1 31213950 CCAACTGCTCATATCCTCGTCTTA 24 59.962 TAGGCACGACTGTTCACTTCTATC 24 60.142 229

chr1 31293048 TATTGCACTCGACTCCCTTCTAAG 24 59.902 GCATGGAACGACAAAATGAGAGAA 24 60.083 283

chr1 31313625 CAATAATACCACCACAAACCCCAG 24 59.84 ACCCCAAGTTCTGAAAGGTTATGA 24 59.894 232

chr1 31317166 CTTGGAGCCATTCAAATACACTGT 24 59.54 CAAACTGTATGGCCTTTCATGTGA 24 59.782 268

chr1 31321581 CTCATCGCTTTCACATCCACTTAC 24 59.967 AGCCGTAGTAGTAACAACAATGGT 24 60.021 270

chr1 31329795 CGTAGCAAGTCTAAATGATCACGT 24 59.201 AGACAGAGACAAACCACTATCACT 24 59.164 201

chr1 31333649 AAGACACTCACCTACTTGATGCTT 24 59.959 CCTTGGGCGATGCATGAATTTT 22 60.419 227

chr1 31476518 GGGTGAAGAAACGACTCAAAATGT 24 59.964 GGGTATTTTCGTCATTTCACCTCC 24 59.904 287

chr1 31480685 GCACGAAACGCTAGCATTAAAA 22 58.515 TGGGGAGAGGTTTATTATTGGTAGT 25 59.026 269

chr1 31483020 CAAGCAGTTGGGTCATTAAAGTGT 24 59.962 CGAGAGAGTTATATGAGATAAGGCA 25 57.616 261

chr1 31489044 ATCAGAATTGCAGATGGTTCCTTG 24 59.598 GAAGTACTGTTAGAGGAAGCGTCA 24 60.083 290

chr1 31502380 GCTTGACATGGAAAAGAAACGGTA 24 60.024 GTGACCACAAAACATAACAGAAACG 25 59.546 189

chr1 31505055 GCCTCTTTGTTGTAGTCCTCAATC 24 59.607 CTGAACAACGGAGAATTTCATCGA 24 59.611 280

chr1 31509524 ATTCTTTTAACGACTGCCCATGAC 24 59.844 ACTAGATTTGGTGGTTTATTGGCC 24 59.29 277

chr1 31509682 ATTCTTTTAACGACTGCCCATGAC 24 59.844 ACTAGATTTGGTGGTTTATTGGCC 24 59.29 277

chr1 31534729 CGTATGCCCAAAATATAGTAGTTGCC 26 60.348 CTCCTGCACTGTCCCATATATTTT 24 58.62 269

chr1 31535004 GTTACTTTTACGGTAGAATAGGGTC 25 57.006 TGAGAGTGTGAGGTTTGAGGAATT 24 59.897 249

chr1 31535217 AGTGGTGAATCTTCCTATCCATGA 24 59.029 GCATGTAAATAGGGGTAGACGGAT 24 60.02 182

chr1 31536885 CGTGTGGTCTTATCAACTCAATGG 24 59.907 GTGGAGAGAGTTCAGAGAAAGGAA 24 59.718 300

chr1 31538710 TTTAGTCCACTTCACGATTAGGGC 24 60.622 ACTTTCTTGATGATGCACCGAATC 24 59.905 232

chr1 31543583 GGAGACAGGATCAAAGTATAGGCA 24 59.655 TCTTGCACCCTATATCCCTAGCTA 24 59.955 283

chr1 31545686 CAGGTTGATTGGGTTGGAAATGAA 24 59.96 TCATATCAGACTCTCAACCACTTG 24 58.029 202

chr1 31548409 CGACAAAGAGAACGCACATAAAAC 24 59.393 TCCAGGAAGCAATTTGTGAGTTTC 24 59.962 293

chr1 31548745 GAAACTCACAAATTGCTTCCTGGA 24 59.962 GCAACTCCTAGGGTTTCATCACTA 24 60.081 214

chr1 31555191 TGAGTCTCACCAACATGCCAATAT 24 60.324 GTGCAGATTGTTTTCCCTGTTTCT 24 60.202 287

chr1 31578755 TGACATATGGCAAGGAGTACGTC 23 60.181 CCTTTCATCAACGTATGTAGTGCA 24 59.37 275

chr1 31643327 TTTTGTCACCTCCCTATCAATCCA 24 59.709 CATTTGCAGGCCATTGATTTTC 22 57.39 279

chr1 31661141 CAAGGCATGATAAAGCAAAGGA 22 57.276 TCATCTTGGTCGCTCATAAACTGT 24 60.323 208

chr1 31702631 GCAAGAATTTATGTCCCTATCCACA 25 59.172 CAGTGATACAATTTGCTATAGGTCG 25 57.89 267

chr1 31767459 TGTAGGCTTGTTCAGTTCACTCTT 24 60.142 GAGATCACAACCAACATACCTCCT 24 60.081 223

chr1 31782814 GGTGTTATTTTGCGTAGTTGGAGT 24 59.785 GGATGATCAATACAAAGCCACAACA 25 60.106 246

chr1 31860430 GGTGGGCCGATGAACTTTT 19 58.346 GGAAAGACGATGATAAGTAAGGGC 24 59.249 257

chr1 31861724 ATCACTTCATTTCTCCTCAGCCTT 24 60.019 CATAGAACACGCAACTCAAACACT 24 60.025 253

chr1 31863391 TTGGTAATGTGATGGTTGATGCTC 24 59.601 AGTGCTCTGTTTTCTGTTGATGAG 24 59.486 247

chr1 31868162 CGTGAATTAAGTACTTTTGTGGCG 24 59.156 GGTAGACCCTCCAATCGAATCTC 23 59.994 260

chr1 31872173 CAAGTTTTGTTGGAGGTTTGAGAGA 25 59.875 TTGAATGTTGGAGCTTCTTCATGG 24 59.781 273

chr1 31875777 GAGAACCACAATAGCTTTTGAACCA 25 59.99 AATATCCAGACTCACGCCTTCTTT 24 60.081 196

chr1 31880088 AGGTTGTAGGGTAAACGATTGTGA 24 59.959 GGCAACCTGAATAACCCGAATTAA 24 59.6 176

chr1 31881761 TCCCACTTTAGTTCCCTTCCATTT 24 59.893 TCATCACCACCATCATCTTCCATT 24 60.08 296

chr1 31881864 ACCTTCCTCCCATCAACCTCTATA 24 60.078 TCATCACCACCATCATCTTCCATT 24 60.08 234

chr1 31888198 ATGCTCACTCTTAACCTTGGACTT 24 59.958 GATTTGATAACACAAGCGCTAGGT 24 59.667 227

chr1 31889010 CAACACGCATATCTCCAAAACACT 24 60.083 TGACTACTTTCTACACCAACTACCT 25 58.927 235

chr1 31892490 GTCCACAACTGCTACAAAGTATGA 24 59.005 AAGCACGGTTAGACTTCAAGAGAA 24 60.202 300

chr1 31894655 ACGTCAAGATGGAGAGAGACAAAT 24 59.778 ACGAGTTGGGATTTTGTAAACCAC 24 59.963 134

chr1 31898028 CAAGGGAAATTGGAATGGATAACC 24 58.083 GTATTGGTTTAAGGGGTAGTTGCA 24 58.988 295

chr1 31898327 AAAGGGCCAATATTGCATCCACTA 24 60.633 CAAGACTTCTGAACTTTGCTGCTA 24 59.487 244

chr1 31900066 CATGCAGGGAAAGGAACCATAATC 24 59.962 GTGACACCCTTGAACTTGATTTGT 24 59.902 119

chr1 31904882 CCCTATTGCTCAACCCATTAACAC 24 59.902 AACAGTGTTTGGTGAATCGATC 22 57.251 191

chr1 31923255 TACCCTCGACTTTTAAATCTCGCA 24 60.082 GAAGAAAGACAACGCTCGATGAAA 24 60.085 187

chr1 31931795 ATACCTGTCAACCTTGCTTAACCT 24 59.957 ATGGTGTTATTTGGTTGGGACTCT 24 60.203 194

chr1 31936616 GTTGGCCACTGTGCATTAATTT 22 58.337 AGCCGAGATTATAGATGACCAAAGT 25 59.694 239

chr1 31937333 CCCATTTTAGATCTCGGTTTGACA 24 59.059 GCATTACATTCGAGAACTCAAGCA 24 59.907 277

chr1 31940819 AAGAGAGATGGAGGAGGAAGCTAT 24 60.141 AGTCTTTAGTGGAATGGCTGATAGT 25 59.573 250

chr1 31947456 CTCTCAAATTGTGGGCCTTTCTTT 24 59.96 GCCTCCAACTATACAAAACACACC 24 60.083 257

chr1 31950181 TGAAAGTTGAGCGAGACAAGATAG 24 58.836 GACATGTTCTACTATCTCCCAAAAG 25 57.222 243

chr1 31951979 ACTCACCTGTGTTATGGACTTTGT 24 60.141 TTCAAATCAGGTACATGGAGCCTT 24 60.264 124

chr1 31959867 TCGATGATTCACTTGAGCTACCAA 24 60.082 GGTCGACATCATCCTTTTAAAGATTGC 27 61.151 238

chr1 31964306 CCCATGACATATCGTATAAGAACTCC 26 58.964 TGGGAATAGTTGTAACGACCAA 22 57.379 283

chr1 31965416 TCCCCATTACTTTGATTGGTCCAA 24 60.203 TTAAGATTGGAGGCAGAAGATGTC 24 58.568 295

chr1 31971087 CACTGAAGTTTGGGGAGAGACATA 24 60.02 TCCCTATGCCAGTTTCAGAGTTTT 24 60.203 218

chr1 31979347 ATTACGTCTTGTTGGAGCCTTTTG 24 60.023 TGGACAGTTGTAGTAGAGAGAAGC 24 59.54 113

chr1 31980480 AACTATTTGCACTCTTCCTACCCT 24 59.464 GCCCAAGAAAAGTCAGTCATAGTG 24 59.845 210

chr1 31982122 CCAAACTACCAGAGAACCAACAAC 24 59.964 TTTAGGGACAAAATCGAATGGGGA 24 60.264 270

chr1 31996602 GCTCCTATACCTTGTGCAAAGTTT 24 59.541 CAACTAGCATGAAGAGCACAATGT 24 59.845 281

chr1 31997834 ACACTTAGAGTAACGGGAGATTGA 24 58.985 TCTCAATCAATTATCGTTAGCTCGC 25 59.597 220

chr1 32004093 TTCGTAAAGGTCCCCAACAATCTA 24 59.713 ACCCATAGTTCATTGCATTCCCTA 24 59.832 165

chr1 32015036 AGGGAGCTTAGTGATGTTTTGACT 24 59.958 CAGGCAGGGGTAAGATGAGATAAT 24 59.712 284

chr1 32017275 TGACCCAGTGAACTAACATCCAAT 24 59.957 CCATTTGTGAGTCTTGGTGTTCAA 24 59.902 85

chr1 32024835 GGAGGTAGTGTAGTTGGTTCTTGA 24 59.717 TTGTTTCCAATTCCCAAGACGAAG 24 59.963 91

chr1 32034005 TGTTGTATTCTGTAGCCTTGGTCA 24 59.959 CTTTAACCCCTTTTGCCATCCATT 24 60.019 203

chr1 32034868 CTTTGTCAGAAGGCAGAGGAAAAC 24 60.261 CACTATGATCCCTTCCCTACCATG 24 60.02 264

chr1 32035615 GAGATGCTACCAAGGGCTTTCTAT 24 60.202 AATTGGCTTGTAACTCCACCATTG 24 60.021 137

chr1 32041452 TAAAAGAACGGGCTACTTCCAGAA 24 59.959 ACCCTATCACCGATAAATTTGAGG 24 58.379 250

chr1 32043857 GTGATCGCTTTGAGGAGAGTTTTC 24 60.143 GTCGGACACAAAATCGTTAGTCAA 24 59.79 246

chr1 32044034 TGATCGCTTTGAGGAGAGTTTTC 23 59.003 ACAAGTGTTCGTTATGTGTCCAAC 24 59.965 300

chr1 32047230 AGACTTCAGCAACGATCATATGGA 24 59.9 GCTATCTGGTATAGTTGGAGCTCC 24 60.082 281

chr1 32055403 GGAAATGGTTATGACTAGGTCCCA 24 59.835 GGCTCTGCGACTTATACTGGATAT 24 59.841 292

chr1 32111243 TCGCTTTTCCCAACTTTCTTCTTC 24 59.964 CAGAAGGCTCATAGTGGAATCAAA 24 58.812 216

chr1 32115480 TTCCTTGCCGTCATGTATGTAAGT 24 60.323 CCCTTGGAGTTTTAATTGGTTCGT 24 59.72 230

chr1 32156685 ACAACGCTCGATCTCTTCTATGTC 24 60.497 AGAAAGGTCATACTCACAACAGCT 24 59.959 223

chr1 32215805 TATGGGTGTAAAGGAGTCGAGTTG 24 60.082 CGATAGTGATGCATCTTTGATGTGA 25 59.536 300

chr1 32216624 ATGGTGTATGTGTGCCTATCCTTT 24 60.08 CTTCCTCACAACCTCAAACTCTTT 24 59.116 258

chr1 32219383 GAAGAGTTTGGACCAGTGACAATG 24 60.024 GAAACAATGTACATGCCTCAACCA 24 60.022 256

chr1 32228752 TTTCTTCCCAAAATCGTAACCCAC 24 59.721 CAATGACATGTGTTTTGGTCTTCAC 25 59.54 286

chr1 32233629 CTATAACTTGACTACGTGTGGTGG 24 58.9 TCCTAATTAGCTTTTCCGCCTCTT 24 60.081 297

chr1 32249028 CCTGATATTTGAATAGGTACGGTGT 25 58.478 CATGGAAAATGTTAGGGAGGCAAA 24 59.777 203

chr1 32254597 CTCGAGCTCCCTTAGACCTATACT 24 60.263 TGGTGACGGATGGAAGTTGATAAA 24 60.263 180

chr1 32270668 AATCCTTTCACCGGTTCCAATAGA 24 60.019 TGGAGCCAATGTGACTTGATAAGA 24 60.019 251

chr1 32283986 AGGGATCGAGATGAGAATTATGCA 24 59.47 TGAATTGGGAGGTGTTTGGAGTAA 24 60.141 275

chr1 32285398 TGGATTATGCATTGGTGGTAGCTA 24 59.896 CCTAGTCATGCAAGTAACCCCATA 24 59.897 261

chr1 32288077 GTTCCTCTCTTGTAACCTTTGCAC 24 60.024 TGGAAGGAGGATGACAACTTGTAG 24 60.02 254

chr1 32301569 GTATGTGTGAACTGGGCTTCTTTT 24 59.722 GTGGAGACGTAACAATCAAATAGTC 25 58.009 289

chr1 32305275 GAACTCGTGACTTGATGTGGATTC 24 59.908 AAGTGTGGGGTATTTTGTTTGGAC 24 59.9 266

chr1 32309869 ATCCCTACTTGCATGGAAATGACT 24 60.08 AGTTTGGCTAAAGAGTGAACCATG 24 59.48 270

chr1 32312851 CAAGATTAGATTGAATGGCTCGCC 24 60.32 ATGTGACACAAAGGAGCTAAGAGT 24 59.959 260

chr1 32313646 CCCCTTCTTTCGAACATTGTCATA 24 59.059 GTGATCAGCCTGCCAAGTCTATAC 24 61.036 281

chr1 32318735 AGTGACATCCAAACCGTAGAAGAG 24 60.322 ATAACTAGGACACACACACACACA 24 59.9 123

chr1 32322531 TCATTGCTGACTTAAAACACCACC 24 59.963 AACATAAGGTGCTCAACAAAGACG 24 60.024 280

chr1 32363463 AGAATCACGTGAAGAAGCTACCAT 24 60.081 CTTTTGAGTTGATTCTGCAATGGG 24 59.073 238

chr1 32370912 AGTGTGATAAGTGGCTACATGAGA 24 59.288 TGGGGCCCGATATATGAAGAAAAT 24 59.956 240

chr1 32372196 CCATTACACGAACCATTTGAACGA 24 60.084 TTTCCAGTCAACGATTGTGCAA 22 59.315 258

chr1 32373578 CCAATTGAAACTCCGTGGTTACAA 24 59.963 TCTTCTGTCCTCCACACTTCTTTT 24 59.836 140

chr1 32373859 GGCTGCATCTCAACAAATCATAGT 24 59.662 TCCTTCACTCTTTGTTTCTAACCCT 25 59.868 270

chr1 32376262 TGGCATGAATTTAGCAACTTGGAC 24 60.322 CAAAGGGAGTGAAAGTAAGGGACA 24 60.446 197

chr1 32377114 TAGTCCTCATCATCCCAAAGCTTC 24 60.142 CCAACCTTTGCACCTTCATTTTCT 24 60.443 211

chr1 32378947 GCTGACTAAACAAAGGCCTCAGTT 24 61.522 TCCTACTGTCTGATTATGTCCACA 24 58.731 228

chr1 32379920 TGTACGCCGACACAATAATAAACG 24 59.968 GTCTAACGAGTGAGATGGTAGACC 24 59.965 248

chr1 32380587 TGGGTGGGATAAATACTCTCTTTCA 25 59.271 AAGGTGAACTAAGGCATTTGTGTG 24 59.962 223

chr1 32405832 AGCATCAGATGTATCAAGGAGCAT 24 59.958 ACCTGCTTTATTTACTGCCCACTA 24 60.019 265

chr1 32410936 AAAATAAAATGGTGGTGGTGGTGG 24 60.202 CACCTGACACAACCTCTCCTTATT 24 60.263 299

chr1 32417143 AAAGGAGAAGATAACATGGGACCC 24 60.08 TGGTGGCATCAAATCAACTTCA 22 58.771 196

chr1 32419513 CCACCACAACATACTAAAATGAGTCC 26 60.128 ATCCTCCCTCCCTCCCTAATATTT 24 59.886 103

chr1 32438397 GTCATTACTGCAACTCTTAGCGTC 24 59.968 CAAAGTATAACAAACCCTCCATTGG 25 58.37 220

chr1 32442961 GGAATAGCACACCAACCTTGTTTT 24 60.202 AGACGAACAAAACCAACCAAGTAG 24 59.666 298

chr1 32443127 GCACATTGGTTTCCTTTTCTCCTT 24 60.202 TCAGTAATAGTCTTGAGTGGCTGA 24 58.983 262

chr1 32448937 TGGAATCAGCCCTTAAGAGAGAAA 24 59.464 CCACTAATAAACTTGTATCGCTCGG 25 59.825 192

chr1 32449037 CATAGGTCACCCATGCAAATCAAA 24 59.84 GCTCATGTTCCCTTCCATAGCTTT 24 61.171 269

chr1 32452062 ACAGAGATATGGTAGATGGGATGT 24 58.34 TCCAACAGACCCACATTAGTACAA 24 59.652 248

chr1 32457195 AGTTCTATCTTCTCTCCCCTGTCA 24 60.017 CACCTTTGCACCTTTGAAACCTAT 24 59.961 292

chr1 32467255 TTGCATAAATTCCCCAGTTTCTCG 24 59.842 ACAACGGTTACAGTAAGTCCTACC 24 60.022 238

chr1 32474911 GATATTCAAAACCAACCAAGGGCA 24 60.021 GTTTAACTTTGCTTCTAGTCGGTTG 25 58.866 229

chr1 32475549 CTTCATTTTCGAGAACATCAAGGTC 25 58.69 GGAAAATGATCGTGTTGGTTGTGA 24 60.261 272

chr1 32479287 TTAGGTCATTGATGGTAGCTCTGG 24 59.898 CCAACCAATTAGTAACCATCTGCC 24 59.902 267

chr1 32480986 GATATCTAGTGCAATCAAGGCCCT 24 60.263 GAGTCGAGTATATGAAAGGAGGCT 24 59.479 250

chr1 32484668 AAACCAAAAGACAAGAAGACAGCC 24 60.142 TGGTTGGTGGATATGGTAATGGTT 24 60.017 284

chr1 32486851 GAAAGTTTGGAATGTGCGAGATGT 24 60.32 AGCAATTCGGTTTAGTAGTCCTCT 24 59.533 196

chr1 32489212 GTTGACTGTTTGAGAAGACTGCTC 24 60.025 ACCCAAAGTCCTTATCCAAAACCT 24 60.141 223

chr1 32500652 GCTTGGGTACTATACTGTGGGATT 24 59.897 CGATGGAGTAGGTTTAGGAGGATG 24 60.022 280

chr1 32512003 GTTCGCACCAAGAAGAGAAATTGA 24 60.024 TCTTCCTGTGGTTAATTGAGGTGT 24 59.896 263

chr1 32520653 GGTCTCTCTCTCTCTCTCTCTCTC 24 59.961 TCATCATCAATCTCGGAACCATCA 24 59.899 234

chr1 32521167 TCGTCCACAGTTCTCATATATGCT 24 59.415 GTCGATCCTCAGCTAATTGTCAAA 24 59.13 263

chr1 32521361 AGTCCCTCGAGTAATGAAAATGGT 24 59.775 TTACACACGCCGAGTTATTTCATG 24 59.908 284

chr1 32522650 AGTCTCACCCATGTTGATCAAAGA 24 59.958 TTCCCTTAACAAAACAGGAGGTGA 24 60.08 189

chr1 32528221 AAAACCACATCTTGAACCCCTT 22 58.347 ACTCCCACCGCTATTACTCATTTT 24 60.081 276

chr1 32535680 AGTCTGAATTGTCTGGTTACTTGGA 25 59.928 CTTTTGAAGTCGAATAAGGGCATCT 25 59.645 239

chr1 32547339 TATCACCAAGCCCAATGTGTAAGT 24 60.264 TTCTTCACTGGTCAGCTTAAAACG 24 59.727 226

chr1 32549266 TAGACTACAACACTCTGCCCTCTA 24 60.019 TGGTTAAGCTTCCCTATGGTCAAT 24 59.77 296

chr1 32550511 GAGTGGCACTTAGATATTTGTTAGC 25 58.051 ATTAGATATGCTCCCTCTCCACAG 24 59.223 93

chr1 32550859 CCTGAAAAGGTCGGCTCTAAGA 22 59.766 ACTATGGCTGTTGAAATGACCTCT 24 60.019 149

chr1 32563977 TGGTGTCTAAAATTGGTAGGGGAG 24 60.019 ACAACAAAAGATGGAGAAGGAGGA 24 59.895 124

chr1 32567673 CAACATGAGAACTGATATCCCAAGA 25 58.648 GTGTGTGAGAAGGGATTGAAAGTG 24 60.024 147

chr1 32592284 TGCGACTTTAACGTCTTCATAGGA 24 60.083 GAAATCGACATATGACCACAAGCT 24 59.428 182

chr1 32602293 GACAAAGGTATTGGATGTGGAAGC 24 60.142 GTACAACATTGGATGCGACATCTT 24 59.905 292

chr1 32695288 ACTAAGAACCAGCCACCCATTTAA 24 60.203 TAGCCTGAAAAGAGAGAGGAGAGA 24 60.018 129

chr4 805360 ATTGATAGGGTGAGAAGATGGAGC 24 59.959 CATCACGAGTCCATGGAAAAGATG 24 59.965 229

chr4 1860434 ATTGTAGGCATGTGTTTGAGCT 22 58.575 TCTCCCTAACATCTCCTAAAGCAA 24 58.97 268

chr4 1881303 TTCTCTTCTTTAGCCGCCAATACT 24 60.081 TTCACACTACTCGTTGCATCAAAC 24 60.025 277

chr4 1920524 TGCCCACTCCTATATGATTCTTCC 24 59.713 CGATGTATTGAATAGTCGACACACA 25 59.258 192

chr4 1920696 TGTGTGTCGACTATTCAATACATCG 25 59.258 GGTGAATGAGTGCAAACCATATGT 24 59.842 163

chr4 1924619 GTTACCCTCAAAACCAACAAAGCT 24 60.142 GACTTCTATTCTAACCGGCTTTGC 24 59.966 138

chr4 1928788 TCCCCAGTGCTAGTTTGTTATTGT 24 60.203 CATTTATGAAATTGATAGCCCTGCG 25 59.14 276

chr4 1937488 TTCAGTTTGACCTACCACATCACA 24 60.142 ACAAATACGTCCACTTAGCAGAAG 24 59.07 193

chr4 1939808 TAAACGAGGAGCATGACAGAGAAT 24 59.84 TCCCAAAGTGATCTATTCCCCAAT 24 59.518 258

chr4 1942763 AATTTATTAACACGGGGCTTCGAG 24 59.666 GCACCGACGGATTTAGTATAGGTA 24 59.784 230

chr4 1943115 TCAGAAGAGCTAAGTGATGATCGG 24 59.963 TCATCCCATCCCATTCCATGTATC 24 60.018 231

chr4 1943471 ATTCGGCTTAGGGACCCATTATTT 24 60.141 CATAGAGCTACATGAGGAGGTGTC 24 60.022 221

chr4 1944428 ACTTTACCACAAACCCAAAAGAGAG 25 59.64 GGTACTCGAAGTTTCTAGGCCTAC 24 60.202 180

chr4 2005099 TGGATATGACCGTAGATTGGAGAA 24 58.855 TCTTCTCCTTAAAATGGGTGTGGT 24 59.894 281

chr4 2041106 CCACGACTGCAAAACTCAATGAAT 24 60.557 TCCTTGTTGTTGAGACGTTTCATG 24 59.965 214

chr4 2042835 TTGGTTTAAACTCACTCCTCGTCT 24 59.9 GCAAGAAGTGGTGGATTGTGATTA 24 59.541 166

chr4 2139783 TTCTCTCATCTTTCCTTGTGCGTA 24 60.021 ATCCCTCTTAGCCTATCTCGTACA 24 59.957 197

chr4 2149140 TCCAACACTAACTACGGAGAGAAC 24 59.783 TTAGAGAGGAGGTTTGGATGTTGG 24 60.019 151

chr4 2151629 GAGTATACACCAAGATGCTACATTC 25 57.355 AACTCTAAACGCAGGTGAGATTCA 24 60.262 297

chr4 2154274 ATGATAAACCAAGTGACTCGAGGT 24 59.777 CCCACCTTACATCATATCACCTGA 24 59.652 282

chr4 2157725 GTTGCATGTCATGAACTTTCCTCA 24 60.023 CTGCAGCGATTTCTAAGAGACATG 24 60.026 262

chr4 2161353 GAACAGACTCCTAATCGCATAAACT 25 58.957 TTTGGAGGCGGTGATATGAGATAG 24 60.021 203

chr4 2199499 TCACACACACCATTAAAAGTCAGC 24 59.964 TGTATGAGACGTTGTACTATTGTGC 25 59.197 165

chr4 2330735 CTATCAAGCTACCAGTTCGTAATGT 25 58.726 CCTTTTCATACCTCCTCCACAAGA 24 60.019 236

chr4 2346886 CTCCTCCACTCACCACTCCTAATA 24 60.631 TGGCAGTTTGTAGTCTATCAAGGA 24 59.469 286

chr4 2351889 CACACCGTACACTTTGATGCTTAA 24 59.788 GGTTGAGTTTGACTTTAGCACCTC 24 60.024 296

chr4 2356444 CTTGATCGTCGGTTTGCTTCTCTT 24 61.617 CTAGACGAACCCAGAAGAGATGAC 24 60.202 180

chr4 2357665 TCCCAAATTCTCCTTCTCCTTCTC 24 59.774 TCATCGAGCATAAGAAACGGATCT 24 59.962 299

chr4 2361713 TTGATGTCCGATCTCTTCAACACA 24 60.262 AACTATATGCCTACGAGTTTCCCC 24 59.96 265

chr4 2397504 AGGAGAATGTGTTTTCGTGAGGAA 24 60.444 TCATATGAGAAGTTCCCAAGCCAC 24 60.626 279

chr4 2627832 GAGAGGAAATTAGTGGTGCGTTTT 24 59.784 AGGACAATGAATCTCTACACATCGA 25 59.639 238

chr4 2661870 TGGGAGAAAAGATTACTGACTGCA 24 59.958 GCGTTTCTTGTGTTTTGTTCTCCT 24 60.674 281

chr4 2673622 CTGCCCTCAACCTGAAACATAAAG 24 60.083 ACACCTAAATGCAGTAGAGTCAGG 24 60.081 269

chr4 2674312 CTGATTGGAATTCTAGGTTTGCGA 24 59.364 CTCATGTTTCTCTTTGGGCTTCAG 24 60.083 262

chr4 2704758 TGCTCCCATTTGACAGAAGAAGTA 24 59.958 TAAGCCCTTTGTGAAGTAACCGAT 24 60.263 243

chr4 2711601 CTGTCGAATGATGAGCAAAGGATC 24 60.025 AAAGCCTTGAAACCCTACCTAGTT 24 59.894 258

chr4 2717876 ATTTTCATTGGAGGCGTCGATATG 24 59.786 CCATTAAACCGCCAAAAGAAAAGC 24 60.084 279

chr4 2718148 GCTTTTCTTTTGGCGGTTTAATGG 24 60.084 TTCCTCAAACACTATGCTCATCCA 24 60.019 216

chr4 2725862 GTCTTCGTCTCACAATGCTCAAAT 24 59.847 CGAAACCAAGCTTGTTCAAGAGAT 24 60.024 246

chr4 2734535 TGCCAAGTTCTTCGATGATCTGTA 24 60.082 TCCACCAACTTTTGCATAGTAAGC 24 59.782 229

chr4 2739480 GGAGGTTTGAGTAAGGTTGACGTA 24 60.262 TCCACAGATTCCACACACATAT 22 57.157 246

chr4 2759407 TGTTTGTGAGTGGTTTTGGATTGG 24 60.382 CAGCTCATTCATTCCCTAACCAAC 24 59.903 299

chr4 2766428 CCTCTAATTCCCCTTCAACAATATGC 26 60.016 CCCAATGTTTCCTGTTTTGTTATGG 25 59.3 299

chr4 2768624 CACTCTTGGGAAAGTAGAGGTCAA 24 59.96 CTTTCTCTCTTCCTACTCCCCGTA 24 60.628 237

chr4 2773588 ACTGACTGTCCTTTTGAGTGGA 22 59.228 CCTTGGAATATATTGTCACGTGTCA 25 59.185 255

chr4 2773701 TGACACGTGACAATATATTCCAAGG 25 59.185 GTAGTTTAGTGGAAGGTGGCAAAG 24 59.784 248

chr4 2773834 GTCGGCTTCTTCTTTCATAACCAT 24 59.364 GTAGTTTAGTGGAAGGTGGCAAAG 24 59.784 111

chr4 2776075 TCCACAGAAATAAGCGTTCAAAGC 24 60.32 AGAGAAAATCGTGGTTCCAGATCT 24 59.776 237

chr4 2783015 TTCTGGACGACTCAAACAGGTAAT 24 59.96 TCGAGTACCCAATCCCTCATACTA 24 59.895 230

chr4 2783486 GCTATTTCGCTATCATCCTACTCT 24 57.797 GAATGGTTTGCTGTACACATCGAA 24 60.084 287

chr4 2796097 ATTTGACAATCTTACCTGCTCCCA 24 60.264 TTACTATTCTTGGCCTCTTCTCCA 24 58.97 287

chr4 2804627 GGCTATGAGTATTTCTTTAGACAGG 25 57.045 TCTCTAATCCCACTTCCCAAACAC 24 60.263 268

chr4 2806120 GGGAGCTTAGTTTGGAACCATTTC 24 60.082 ACGGTCTCTTTATCAATCTATCGTG 25 58.56 215

chr4 2812981 CCTCTTGTTCATCGACCTTCCTTA 24 60.082 ACCTCTCAAATCCTCACTCCTTTG 24 60.263 287

chr4 2818160 CAAACCAAGAGTAAAATGCAACAGC 25 60.051 CCTCATATTAAATCGCAAATGTCCG 25 58.92 287

chr4 2819756 CTTGTGCTTTGAGTCTTTGCTTTG 24 59.733 AACGAGATTACACCGAAACAAAGC 24 60.319 241

chr4 3053773 TCAAATCTACCTCTACCTCCACCT 24 60.016 TGGAGTACATATGGGAACTTGCAA 24 60.019 235

chr4 3062298 AGAGTAATGAAGCGATCCTATGGG 24 59.778 TTTATTCCAACCGAGGTGTGTTTC 24 59.724 262

chr4 3063874 CCTTGACCAAACAACCTAACAACA 24 59.841 AACTTCCAGATGATAAACTTGCGC 24 60.143 292

chr4 3281699 TCCCATTTTAAGGCTCATCAATCTC 25 59.17 ACCTATAAAATCCTCGTGCTTCTCA 25 59.871 296

chr4 3300184 CAAGCCCTGCCACATCTAATAAAG 24 59.963 GACTTGGAAGGTTACTGAAGGCTA 24 60.02 178

chr4 3300807 ATAACCATTCTCCAACGCCAAGTA 24 60.324 GATTGGGGAAATTTCAACACTGCT 24 60.263 85

chr4 3300924 GCAGTGTTGAAATTTCCCCAATCT 24 60.263 TTGATTCTTACCGTTCGTGAATGC 24 60.143 273

chr4 3354078 CTTGCACGTTCTCTCCATTTCAAT 24 60.083 GAAGATTGATGAGATACTGACATGG 25 57.345 164

chr4 4108095 TAACTTCCACGACCTTCAATCTCA 24 59.718 GGAAGGGAAGGAGTTGTAAGTGAA 24 60.202 298

chr4 4110240 TGTTGTTTGGGACCTGACTATGAT 24 59.957 GCCCTCAAGTGTCACCTATATCAT 24 59.959 208

chr4 4114160 GCCACTACAGTTTCCCATTTTGTA 24 59.479 CGTTATCGTCATTTCACAGCTCAA 24 59.909 144

chr4 4116550 GCAATTTGGCGAGCATGAGTG 21 61.06 CAAATTGCCCAATATAGTCCCCTT 24 59.099 300

chr4 4120826 TGTGGCGCTTGTATACATTCTAGA 24 59.901 AGTAAGGTTAAGAAGGCCGAAGAG 24 60.082 222

chr4 4123797 ACGTATAGTTTGAGGCATGAAAGTG 25 59.649 CCTTGCAACTCTTCACTAACAGAA 24 59.184 278

chr4 4125795 TGAGATCGCGTGAAAGAGTTATGA 24 60.142 CTCTTCTGCCTTGTTGTAGTTGTG 24 60.025 252

chr4 4126403 TCCCTTTAGTTTCCTTCCTCTTCT 24 58.837 AGATCAGGTTAGCATGTTATCCAC 24 58.385 273

chr4 4131413 ATCTCCATCCGAATTATACTGCCG 24 60.382 GAGGAATTGACAATTGAACGGTGT 24 60.023 254

chr4 4175318 CAACACCATGAGATGCACAATGAT 24 60.142 AGCAACAGTATTACCCGAAGAAGT 24 60.02 272

chr4 4178397 GAACAACTCAGCCAACTCGAAAAT 24 60.261 TTCTCATGCACCTAATCCAATCCA 24 60.08 215

chr4 4179384 TGTTGGTATAGAGATGGTAGGTGT 24 58.475 ATCCACTTGATTTCGCTCTTCCTT 24 60.566 226

chr4 4195837 TTTAGAGGAAGCTCAAGAGGGAAC 24 60.02 GGAGTTATCAGACACCCTTCACTT 24 60.02 262

chr4 4233465 GACGATGAAGATGGAGAAGAAGGA 24 59.901 TATTTGCTAGTGGGTGGTTGGATT 24 60.264 293

chr4 4252213 CATTGATCATAGGCAGCATTCCAA 24 59.72 AGGCAGAGACACCCTATTTTCAAT 24 60.018 213

chr4 4255560 TGCTGTCCTCTTCAATCTTTTCCA 24 60.446 GATCAAAAGGACACATCAAGAGGT 24 58.994 285

chr4 4256249 CCCTATTGTTGGATGATTGTTGACC 25 60.164 TCATGTGGTTTGTTACTTTGTAGCC 25 59.991 299

chr4 4257626 GGTCTCTCTCTCTCTCTCTCTCTC 24 59.961 ACGACTTCACCTCAAAACACCTAT 24 60.202 287

chr4 4266709 GCTGCCTACATCTACTACCAGAAA 24 59.9 TCCGATCATACCGTTGACTTGAAA 24 60.322 229

chr4 4268030 TTGGTTTGAGGGTTTGAGTGATTG 24 59.901 TTTTGGTTGTTCAAGTTGACTCCC 24 60.082 279

chr4 4273779 GGAGAGAGACGGAGGAAATCAAAT 24 60.142 TAGAGTTTTGTTGGGTGGTGTTTG 24 59.841 225

chr4 4274181 TTTGAACATTTCGGTGAGTAGGTG 24 59.486 GTTATAGTCCTACTCGAACACGTG 24 58.738 223

chr4 4274455 GTTCGAGTAGGACTATAACATGACA 25 57.976 TACATCGGTATAGTGCTAGAAGGC 24 59.542 259

chr4 4277927 GTATCACCAAATACCGCCATCAAG 24 60.024 TACCCAACTCGACCTAGAAACTTG 24 60.022 147

chr4 4283375 TTACGTTCATATCCTCCACATCCT 24 59.101 ATTACTCATCTCCCATGTTGCCAT 24 60.141 203

chr4 4284540 CCAATTGAAACGAGCCACAAATTTG 25 60.559 TTTTGAATATGGTTATGGCGAGGC 24 59.963 282

chr4 4292120 ATGCTAAATTCAGTTCGCCTTCTG 24 59.905 TGGTCCAATAGAAAGAGTAGGGAG 24 59.037 202

chr4 4292474 TGGTAGTTTTGGATTACGGATGGA 24 59.775 GGAACCTTCACAGCAAGAAACAAT 24 60.202 260

chr4 4631036 AAAATAGTTTCTCCCGTTCCCGTT 24 60.748 TGAGAGGAAGAGGAAGAGGATGAT 24 60.079 252

chr4 4632419 TCACCCACCTTCCTTAGCAAATAA 24 59.956 GAGATCAAACTCCCACAAGAAAATG 25 58.615 221

chr4 4741751 CCTAATATCCCTCGATGCAACTGA 24 60.021 GCCCGATTCCTTGTAGTGTGTATA 24 60.202 289

chr4 4928701 GGTTATTCCTCTCCTAAGCAATTGG 25 59.467 CCCCTCCTCTCTTTGACAAAACTA 24 59.958 268

chr4 4934153 TTTGGGATTAAAGTAGAGCAAGGC 24 59.295 ATCCCTACTTCTACTTCAACCACT 24 58.657 143

chr4 4945077 TATCAGCTCTCACAACTTCTCCAC 24 60.082 GACGTATACCCGATCCACAGTTTA 24 59.963 259

chr4 4949994 GAAGTCATATTGGAGCAACGGTTT 24 59.844 CCTATTCTTCTTGTCGCCCTATCA 24 59.961 293

chr4 5021306 GTCACATGTTCTTTTGGTAAGAGGA 25 59.237 TGTGTTGTGAAAAGGACTAGATGG 24 58.937 160

chr4 5341562 GCTTAAACTCTAACAAGGATGGCC 24 59.903 CCTGCTGATTGAACTGTGCTTTAT 24 59.843 223

chr4 5368790 AGAAGGGTACGTGGCAGAATTTAA 24 60.263 ACTTGGGTGGATTTCATTGGTTGA 24 60.939 211

chr4 5491022 CATGTTCCTGATCGCATTCACAAT 24 60.202 CTCACATCTATGGTTTGCATCGAC 24 60.025 274

chr4 5724278 AGAAGAAGAGCACACCCAATTTTG 24 59.962 AGGTCAAGGATATTACACACACACA 25 59.987 282

chr4 5736685 CGTAAGGTTCAACACAAATCAGATC 25 58.693 AGCCTGAAGAACAAGGATGAATCT 24 60.019 215

chr4 5742861 TAGGATGTTGTAGTTGCAAGTCGA 24 60.022 GGGATCTTGGATTGGAGGAAGAAT 24 60.141 175

chr4 5764383 CAGTACTCATGTCTACCGTTTCCA 24 60.082 AGAAAGTAGTGCAAGGGACCTAAG 24 60.02 114

chr4 5764961 CGTTGAATTAATTGTGGCCATTCG 24 59.735 ACCCACCGAAATACTAACACTCAA 24 59.959 274

chr4 5765437 CTGAACCACGTAAATCTGCATTGT 24 60.083 GGGCCTTTAATTTTGGAGGAGAAG 24 59.84 282

chr4 5768948 GAGATAAAACAAGGCATGGAAGGG 24 59.901 GCCTTGCTTATTACATTACCCCAC 24 59.962 213

chr4 5782356 TGTGACACAACTCATAAGGGAT 22 57.091 TGCTATCGAATTCTTGCGCATTAG 24 60.084 251

chr4 5784054 GACGGATATGAAAAGAGAGGTGGT 24 60.142 CACCACTCAATTTCCTTTGTCCAA 24 59.901 176

chr4 5784994 CAGAGCAAGTTTGTTTTGAGGTTG 24 59.436 ACACTCTCGAACTCCTTGAATCAA 24 59.961 223

chr4 5788654 CAAGTTCTGACAGCCTTCTCAAAG 24 60.024 CTCAACCGTAAGCACTTCTCTTTC 24 59.849 183

chr4 5793099 GGGAAACTTAGTGTAGGAGTGACT 24 59.473 AGCTTCATTCCACCATGTAATCCT 24 60.08 287

chr4 5796876 CTACGCCCAATAACTGTACCCTTA 24 59.9 GGTGAAGAAGAAACGAAGGGATTG 24 60.083 221

chr4 5837987 GAATAGGAATAACAACCAACTAGCC 25 57.739 TGAAAAGAAAGAAAGAAGCCCGTG 24 60.202 203

chr4 5839869 CAGTGAAGGGGAAGAGGAAGAAG 23 60.307 TCCCCAAAATCACTTTCTGACTGA 24 60.141 300

chr4 5949740 AGGGTTGGGTTGGGTTAGATTTTA 24 59.892 CGTGCAAAGTGAACCAAAATGAAG 24 60.027 246

chr4 5976807 GACATAGGGTGCAAGACGAAATTC 24 60.202 TCACAGGTTGTTTGTAATCTTGCC 24 59.963 240

chr4 5979249 AATTTGGGTTCACGTGGTATCA 22 58.243 GATCACTCCATTTCACATGCATTAG 25 58.564 254

chr4 5983163 AGGAGATTTGAGAAAAGGGAGCAT 24 60.018 CCTCACATTTTAGGTTGTTTCGGA 24 59.483 276

chr4 5984949 AAGTCTAAGCTGGAACCAAGGATT 24 59.957 TCGCATTTAAGAAAGCCCTAGAGA 24 59.838 290

chr4 5987563 AAATTGTGCCTCGGTACTTCAAAG 24 60.023 AAATTGAATGCTTGGAGAAGGTGG 24 60.021 159

chr4 5990432 TCCTTTTCCATTTTCCTCTCGTGA 24 60.202 CACGCAAAGAGGAAGATGAAAACT 24 60.024 253

chr4 5993987 TAAATCACCAATCCGACCCAAAAG 24 59.539 CTAACGCTCTAAGTACATGAGGCT 24 59.963 283

chr4 6000951 AGACTACTTTCAACGCTGGAT 21 57.037 TCCTGTATTGAAAGTAGCCTTGGA 24 59.466 266

chr4 6005758 CAAACCACATTCAAACGGTCTACA 24 59.964 AGCTTGTATGTCCTTTCCTTTCCT 24 59.957 281

chr4 6055848 TTCTGTTGCGTTGAGAAAGTCT 22 58.468 GTGATAGATGTTGAGCGCCTAGTA 24 60.023 101

chr4 6073751 CTCGTGGCCATATTTTGTAACGTT 24 60.143 GTAAGCTGTAATGATGTGCAATCAG 25 58.803 256

chr4 6074713 CCAAAACACCACACCCTGAAAATA 24 59.9 GGCTTCTTGAATTGTTCTCTATCCC 25 59.702 275

chr4 6079887 GAGGTTATGCAAATCTTGTCGAGG 24 59.965 TCTTCGTTCATGTACCCTCCAATT 24 60.02 191

chr4 6080532 TCTCTTTCTTGGCACGTAAACAAC 24 59.965 TTGTTGCATCAGTGAAGATTGGTC 24 60.023 290

chr4 6080782 GACCAATCTTCACTGATGCAACAA 24 60.023 CTTCTCCTAACCCCATCATTCCAG 24 60.445 252

chr4 6114340 TTACCTCAGAAACCCATCTTCGAG 24 60.082 GTCCTTCATCGCTGATAATTGCTC 24 60.084 300

chr4 6128175 ATAAACAACACCAAAGGAGGCTTG 24 59.961 AAAGTTAGGTTGCGACAAAGGATG 24 60.023 283

chr4 6128466 TGAATGCTGACTTCCTTTTCCCTA 24 59.957 ATGGGTGGTTTAGGATATTCGAGG 24 59.958 265

chr4 6151330 TGATACACCTAGAACATCATCCAGT 25 59.1 CGTCGGTGATTTCCTTCTCTCATA 24 60.202 282

chr4 6158766 CTCTGTCACACCTCATATCGTACA 24 59.664 TTGAAAGATTTGCGACGAGATGAC 24 60.143 293

chr4 6174587 AGCGTCAAGTTAGGTTCAATAGGA 24 59.778 CACAGGTGATGATGAAGCAAGTTT 24 60.023 202

chr4 6182273 TCAACTACGGTACTATTGCTGCAT 24 60.142 ACAACTCCTAACCTGAATCGTGAA 24 59.96 179

chr4 6186466 GGTTGGGCCTGGGGTATATATTTA 24 59.707 CTTGCTCTAACGCTTCTTCACAAA 24 60.025 244

chr4 6195533 CTAAGTTGACACCCCATAGTACCC 24 60.142 CCAATTTTCTGCTCAATCAAGGGT 24 60.021 222

chr4 6269795 CAAAAGTTAGTTCAGAGCAAGTCAC 25 58.805 TTTTGACAATTTGATCGGTGGAGG 24 60.022 114

chr4 6271651 TCGAAGGAGAACAAGTGTGGATAG 24 60.082 CAGAATGGTAATGCGAACTAACTTG 25 58.749 300

chr4 6280902 GCACGCTTAATACTCAAAATACCCT 25 59.704 CTCTCGCAACAGTAAGAAGCATAG 24 59.498 270

chr4 6285160 GAAGCATGCACGGATTGAAGATTA 24 59.964 AAAGTGGACTCCGAAAATCACCTA 24 59.959 282

chr4 6289425 CGCGATGAGAGTAAACCCTAAGTA 24 59.964 CACACGTTTTGGCCTTCTTCTTAT 24 60.023 202

chr4 6290276 TCCATCATGTCTCTACTTTCCATCA 25 59.337 GAAAATTCTTCCTTGCACGCGTTA 24 60.846 172

chr4 6291943 TCCTCTCGAAGTCTTTAAACACCA 24 59.657 TACCGTCACTCATTTGTCACCTAC 24 60.321 214

chr4 6296174 CAGGATTAATTGGCGCGAGTAATA 24 59.311 ACGATTACTTACCATAGTCAACACG 25 58.968 298

chr4 6313251 CCATTTACGACTAATAGGGCCTGT 24 60.202 ACAAAGTGAGTCGTATCCATAGTGT 25 59.815 291

chr4 6383085 GCGTTGCAGTAAGAGATTTCATGT 24 60.143 TCATACCCAACATTCCTCATCTCC 24 59.897 298

chr4 6385845 ATTCCCCGCTCCCCATTTTATTC 23 61.008 CGATTTATCCGTGTTGACTGAGAC 24 59.734 288

chr4 6390963 ACGGGAAAGAGTAATTAGTGGAGT 24 59.224 TCAAGTTTACTCAAGCTACCGTTG 24 59.252 288

chr4 6399208 AATGAAACCTACACAGTGCACAAC 24 60.202 ATAGCGAAAGGATCAGTAGGCAAA 24 60.142 195

chr4 6399740 GAACCTTTCAATATTATCGAGCCG 24 58.083 GGCAAAGCACGATTAGTCTAAGTC 24 59.967 262

chr4 6402643 GTCCAGACGAAGAACTCTCTAAGG 24 60.143 CTACTTGTAGCCTCACATGACCAT 24 60.142 277

chr4 6410357 TGCAGATAGGTGGGGATAAACAAA 24 60.018 CCTTAATTGATGCACCAAAAGCCA 24 60.563 139

chr4 6419958 AATCTGGGAGTGGTTAACGAGAAA 24 59.959 GAACTGCTTCATCCTTTTCACCTC 24 60.083 245

chr4 6420772 ACTACCCCAAAAGTTCAAGGAAGA 24 59.833 CTCTTGGCTAGGTTCTTGGGTTAT 24 60.08 205

chr4 6424799 CAGCAATCTTAGAGTAGGACCCTG 24 60.202 TGCGATTTCTTTATCTCCCGTACA 24 60.142 239

chr4 6427502 ACAGGTGAATGATGATGTTGAGGA 24 60.019 CAGGATTGAGGATTTTGCTGGAAA 24 59.779 220

chr4 6427627 TTTCCAGCAAAATCCTCAATCCTG 24 59.779 GGTTTCACCGAACTTCAAGCATAA 24 60.024 258

chr4 6433015 CACACCTACGCACCGATTTAATTA 24 59.432 GACCTCGTACCAAGTCTAAATAATC 25 57.073 254

chr4 6435440 CCACAAGAAGCAAAGCACAATTAC 24 59.555 CACCCTCATAATCGCAACTCTAGT 24 60.202 282

chr4 6443769 AGTTTTCCCCTCAACAGTCTTACA 24 59.835 TTCTGAAATGTGGTTCTCGGTGTA 24 60.202 205

chr4 6443930 TACACCGAGAACCACATTTCAGAA 24 60.202 GAATTTTAAGTGTTCGGTTGGGCT 24 60.262 280

chr4 6444984 TACACAACTATGACCTGCGCTTAT 24 60.142 TGGTGTATCATATAGCTCAGGGGA 24 60.203 120

chr4 6472551 AGTGGATATATTTAACGTCGTCCCA 25 59.697 GATAAAAGGGAAGCAAAGGGTGAG 24 59.841 189

chr4 6475255 AGATTTGGTGCATGATCGTGTAGA 24 60.383 CTACCAAAATCCCCAAATCCCCTA 24 60.079 231

chr4 6476783 GACTGTCAAATGGTCGTCAGAAAC 24 60.319 CTCAGTCTCCTCTTTGTCATCCAA 24 60.021 254

chr4 6478112 ACGATGTTGTTTTATCTTGGGGAC 24 59.542 CAGGAGACATATGAAAGACGTCGA 24 60.202 158

chr4 6479352 TGTAAACAGGTTGGGAGACATTCT 24 59.896 GAAAGCTAAATGACCGTATAAGAGG 25 57.591 204

chr4 6482625 GTGAACTAAAACTGTGGCCAAGAA 24 59.903 CAGCGCATAGTATCTAACCACTCT 24 60.023 110

chr4 6489253 ATGGTGACGTCTGAAGAACCTAAA 24 59.96 GAACACTGCACAATCTGGCTAAAT 24 60.083 251

chr4 6495422 TGGGAGTGAATTTTGTCTTGCATC 24 60.022 TTGGTTAAATGTGTCTCCCTACGT 24 59.959 295

chr4 6496012 CCCCATGATCAAAAGGCTTAAAGT 24 59.533 AGGCTACAATTTCTTCGTCGTTTG 24 60.084 289

chr4 6497327 CGTGTGTTTGGACCAGCTTG 20 59.971 CGAGAGTTACTGGTTCAAGCAATC 24 59.908 292

chr4 6497979 GAGATGCTAGCAGAACCAAGTTTC 24 59.906 CCAAGGTGCATTTATTCAAGGGTT 24 60.02 221

chr4 6563354 GTGGCCAATTGAGAAATAACCTCC 24 60.142 GCTGCCTACTTCTTTGCTTGTTAA 24 60.023 154

chr4 6566124 GGAATCGTTGGAGCTCATTCAAAA 24 60.083 TTACACCAAGCGAATAAAGGAAGG 24 59.302 222

chr4 6568324 GATTGGATCCGGGTCGAAGTTAAG 24 61.269 GGACATACCCGAAAAGAAGGCT 22 60.356 207

chr4 6568570 CTTACCCGATTGATGAAATTACACC 25 58.275 ACTGCGTTGGATGAGATTAGAAGT 24 60.081 289

chr4 6611128 TCTGGATTTGTAGTGGTGTCTTGT 24 59.897 ACGTGTGTGACTAATTAATCGTGG 24 59.376 206

chr4 6613765 TAACGAGGGCAGAGTTGGAATTAA 24 60.02 ATGAAGGATGGAGTTTTAGTGGCT 24 60.018 295

chr4 6615018 CCACGTGACATACATAAATTTGGC 24 58.966 AATGCTAGTTCTTACCGTGACCTT 24 60.02 213

chr4 6620893 TTCAAACTTAAAACTGGGTGGTGG 24 59.839 TGTAAACACCTAGCCCACTAAGTC 24 60.021 269

chr4 6624925 ATTCTTGATGGTGGGGTTTTCTTG 24 59.716 AGAGTTCATGTCACTACCTTCTCT 24 58.671 293

chr4 6626328 TACAATCGCGCATTTGAGAAAGTC 24 60.436 CGAGGATTTGGAAGATCTATTTGCC 25 60.049 146

chr4 6628800 TGATTACAGTGTGGTTCGTATAGTG 25 58.673 ACCACTACAACCAATTTCTGCTTG 24 59.962 181

chr4 6628901 CGACTCTGCCCCTCATTATAGAAT 24 59.778 TTCTTGCCAAATCCAAACCACTAC 24 59.961 124

chr4 6636446 TAGATTGCCCCGTACAAAAGTACA 24 60.02 GTCAATCAGTTTATGTAGGCTCCC 24 59.183 221

chr4 6640301 ACTGGATGAGACTTTTGATGAGGT 24 59.712 AAACTATTGTGGCTGGAGATGGAA 24 60.264 289

chr4 6644027 TCTAACATTACTGGTTGGCTCGAA 24 60.021 TCCATGCTAACCGAAGAGTTTGTA 24 60.021 211

chr4 6648400 GTCCTGTCATTCGGTCTTCCTTTA 24 60.322 CCCACCCCAGCCCCTTATATATAT 24 60.891 283

chr4 6652462 GCAAATGTTGAACAGAAGCACATG 24 60.085 GAACACCATTTTAGCACAACCAGA 24 59.963 129

chr4 6655503 TCTCTCAAAAGCTAGAAGACTCCT 24 58.671 TGCGACATGGTTTGATCTCTAGAA 24 60.082 182

chr4 6662185 AACCATACAAACCCTCCTGATCTC 24 60.08 CCTCCCAAACCATCTTCATCTCTA 24 59.59 296

chr4 6663931 CGCCAAAGAAATATTAGAACTGCTACG 27 60.984 GACCAAACTCTTATGATCTATCCCTCT 27 59.765 128

chr4 6668525 AGAACGTGAAGGAAGGTTACATCA 24 59.96 CTTTCTCACACCGCTTTTCAAGAA 24 60.202 297

chr4 6672585 ATCCCAACCAATACAATGCCTTTC 24 59.838 CCCTTCGCTATTATCTTTGCCATC 24 59.845 154

chr4 6733824 CCTTCTGAAATTTGTGGCTATGCA 24 60.082 TTTAAGTACTACCCTCACCTTGGC 24 60.02 278

chr4 6739111 GCAACCAAGCTAGTAAACTGTCAA 24 59.725 GGTTCTGTAAAATCTTCTGTTGCGA 25 60.05 226

chr4 6747595 CATGAATTTAGGCCTTGCAAGCAA 24 60.86 ATATGCTCGTCAACCATACCATCA 24 59.96 258

chr4 6748083 TTCCTGATTCATTTCTTGGTGCAC 24 60.022 AGCACGTTCTATACCTCTTTGTGT 24 60.021 201

chr4 6750815 AATTTCTACATCACGAGAGCAGGA 24 59.84 ACTGATGACTGTTAATGCCCTCAA 24 60.263 271

chr4 6751579 TTCCTTTCTTTCCAAATGCACCAC 24 60.443 ACTTCAGTACACTATGCTTAGCCC 24 60.142 253

chr4 6753119 ACTGCATACTGAAGGTGGGTTAAA 24 60.203 TGGTAAGAGATGGAGGAGATGGAT 24 60.141 262

chr4 6763575 TTGGTAGTTTTCTCAACTTGTCGC 24 59.965 GAACAATTCAGAATCCTTGGCGAA 24 60.083 291

chr4 6772083 CTTCCTGCCAAATCCGAATCAAAT 24 60.142 ATCTTCACAGGAGAGAACTTGGAG 24 59.777 292

chr4 6777795 AGTCACTTGTAGCCTTTGAGTCAT 24 59.959 ATAGTAAACCAGCCTATACACCGG 24 59.717 296

chr4 6778137 CATTTATGACGTGCAAGTTTGATCC 25 59.487 ACGACCTAAAACAAGCTCCTAAGT 24 59.96 181

chr4 6782871 AAACCTCAGCATTCGGAAAAGATG 24 60.083 TCTCTACTCACAGATCCTTCGTCT 24 60.081 263

chr4 6785698 AGTGCCTATAAATGGAGTGCAGAA 24 60.08 CACCACATCAATCAACCCTCTTTC 24 60.083 246

chr4 6792966 TGTGAGAAGGCCCTAAATCTATTGT 25 59.808 TGCGCTACCTAATACAACAGATCT 24 59.659 245

chr4 6793182 AGATCTGTTGTATTAGGTAGCGCA 24 59.659 ACCCCAATATCTCTATCCACCTGA 24 60.14 113

chr4 6794694 CTCTGGAAGCCTCTTAACCTAACA 24 59.777 TCATCGGTTGGTAAAATTGGAAGC 24 60.082 127

chr4 6803362 CGAGAAGTTGCCTGCCTTTATAAG 24 59.965 CTCTTAGCGCTTTTGTCGTACTTG 24 60.433 129

chr4 6808702 CAAGATAGCCCAAAACAGTTCGAA 24 59.785 AAATTTTGGGTTTCACACGGCAAG 24 61.45 185

chr4 6815755 GGTTGCACCGTTTATACATCATTTG 25 59.485 CGTCCGATTGCTATTGTTAAGGAG 24 59.791 250

chr4 6839903 TCAGCGTTGTTTTCTCACACTATG 24 59.789 GCAGACCACATCCATAGTATCCTT 24 59.959 272

chr4 6842953 TTCTACATCTCGCAGACATCTCAC 24 60.202 TGAATGTTGAGATGTGTGCAAG 22 57.507 159

chr4 6848231 ATCATTCTCGCCCTCTATTTCTCC 24 60.021 ATATCTTGACTCATCCTGGAACGG 24 59.96 162

chr4 6856181 TGTATCCGGGCAAAACAACATTG 23 60.306 GTGCTTGGTTGATCTTTTGCTTCT 24 60.5 229

chr4 6859615 GTTGCACGATCAATGGAAGGAATC 24 60.732 GGCAGTTTGTTTGGTTGTTTGTCT 24 61.096 288

chr4 6860794 TGCAATAGAGGTGGAGGATTTTGA 24 60.019 TATCCGCAAGTAAACCTCCTTCAA 24 60.02 260

chr4 6865457 TCTGTGATAACGAAGCGGCTATAA 24 59.963 CCTTGAGTTTGAGGGAGGGTATTA 24 59.526 287

chr4 6866347 TACAATGTGCGAACAGGTAGATCT 24 59.84 CAGAGCCATACCCATCAGTAATCA 24 59.959 218

chr4 6877820 TTCATAGCATCCACAACTCACTGT 24 60.263 CTTCGATTTGGGTGATTCAAGAGG 24 59.905 298

chr4 6880950 GCGTTCGGTAGATTCACAAGATTT 24 59.907 TGTTCAAGATGGGGTATGACAACT 24 59.957 295

chr4 6881359 TAAAACTTCGGGATGACTCAAGCA 24 60.504 GTTTTGGCGCATAGTTTCAGATCT 24 60.143 250

chr4 6895320 AAAATTTGATCTGCAGCACTCCTC 24 60.082 GCAATGCACAATCTTAATGGGAGT 24 60.142 215

chr4 6901424 TTAACTATGTCACACCCTGATCCG 24 60.142 GAGTGTCTTACATTTAATCGCGTCA 25 59.712 239

chr4 6902795 TTTTCTCGCCAAACCTTTCTTCTC 24 59.964 GTTGGCATTTGAATCAATCCACGA 24 60.618 279

chr4 6910789 CTGCCACATCAAACCCAATGAAG 23 60.616 TTGTGTGTATGTTTGGAGCATGTC 24 60.023 286

chr4 6913014 TGTGTTAGCGACTAAGAGACTCAT 24 59.299 TCATAGGCAAGTTCTACAAGGACA 24 59.469 121

chr4 6913827 CAACACCCCAAAGTTAGAAAGACC 24 59.962 AAGGGATGTTTAGTTCGGTTTTCC 24 59.478 169

chr4 6918028 ACATCCTAGCATACCTATCTCAGC 24 59.291 CACTCCTTATTCACCAGTCTCCAA 24 60.02 197

chr4 6935753 TGTTTATGAGAAGTGGTCGAGGTT 24 59.96 AAGTCCGGAGAATAGTGAGTCAAC 24 60.082 298

chr4 6936045 GTTGACTCACTATTCTCCGGACTT 24 60.082 GCCAAGTAGTGAGATATGGAAGCT 24 60.202 270

chr4 6936433 TCTTGACTAATTTTGACCTCCCGA 24 59.715 AAAGAGAGAGATACCGACGATTGT 24 59.359 296

chr4 6938199 GAGTTTGCAAAAGATCGAACCGTA 24 60.084 TCTGTCCACTTTCACCGGTATAAA 24 59.716 205

chr4 6943183 ACCTCTTACATGTGTTCTTCAAGTC 25 59.008 AGAGAAATTGATGGAGAAGGGACG 24 60.384 176

chr4 6944198 GTTATGATTGTGGCGTGCTATGAT 24 59.786 ATCCAGTGATCACATAAGGGACAC 24 60.142 295

chr4 6945907 GCGAAACTGAAGTAACTGAAGGTC 24 60.084 TGACTGTCACCTTATTGAGCTCTC 24 60.082 142

chr4 6947082 TGACTGTGAGATTTTGTACCACCT 24 59.897 TCTGTCTCAAGGTAGGCTGTACTA 24 60.019 195

chr4 6950454 ACACATCCCTAATCTTTTCTCCCC 24 60.08 TAACTCAACTGGCACCTGTATGTT 24 60.202 299

chr4 6962382 ACAGTCACAATGCACAATCATCTAC 25 59.877 CGTTTATGAATCGCAACACACGTA 24 60.433 237

chr4 6963716 GCCACACATTCTAAATTTGGGG 22 58.139 CTCTTCGCTTACTTCCCAATGTTA 24 58.824 300

chr4 6965944 TAAAACGACATCAAAGACCAACCG 24 60.025 ACCCACATCTATGCTAGCGTTATT 24 59.96 163

chr4 6977210 TACATAGAAACCGAGGCGAGAAAA 24 60.082 CAATCGCCCTAAACCACAACTAAC 24 60.379 164

chr4 6977918 CATAGTATGCATATTGTGGGGCTA 24 58.256 ACCGTCAACTTAAATACCTCCAATC 25 59.121 288

chr4 6981964 ACTCATTGGGCTAAGTACACATGT 24 60.019 CCTTGTTTGGGCCATAATCCTTTT 24 60.019 124

chr4 6982198 CCCTACAAACTACCCTACTTCACA 24 59.471 GTGTTAGGTAATATGTGTTTGGAGG 25 57.683 268

chr4 6991194 AGTGTTGGGAAGCAGAAAATGATG 24 60.022 GGTCTCCAATCAAATGTAACCACC 24 59.842 278

chr4 6995854 TAAGGTGGTTTTCGAAGGTTGTTG 24 59.903 AAATGGCTTCACTTATTGTCCACC 24 59.779 119

chr4 7005441 ATTAGGACTCCATACACGAACGTC 24 60.202 GCAAGTCATCCCTTTGTTCTTCAT 24 59.781 242

chr4 7009230 ACGACGTTCCTATGGATAGTTTCT 24 59.356 TGTTGTTGGTTGAGATTTGAGTGG 24 59.902 296

chr4 7011202 AGGTCTCATTCTCTCAATCACAGC 24 60.383 TCCCTTGGATGTACTTTAGTGGTG 24 60.02 223

chr4 7135803 GGAAAAGAGAGAAAAGCAAAGCGA 24 60.261 TGTTGTAGTTGAGTTGGGGTAAGT 24 59.835 258

chr4 7162805 TCTCTTAATCACGTCAGGCTCTG 23 59.871 TATTTCGCCTATCTGCATCCCAAA 24 60.446 300

chr4 7166350 CTGCGCATGTCAACAGTATCTATT 24 59.491 GCCATCAGAGTCTAGTGTACTACC 24 59.724 258

chr4 7172477 GCCAAACAAAGCGAGCTAACTTAT 24 60.379 ACTCTCAAAATATCCTGGTTCGTCT 25 59.812 283

chr4 7181016 GCCCAAAAGACATTGCCAAAATG 23 60.058 CTAACAAGGTTGAGGGAGCAATTC 24 59.843 135

chr4 7181182 GAATTGCTCCCTCAACCTTGTTAG 24 59.843 CCGATTTCATCAAGTTCCCAACAT 24 59.842 253

chr4 7212246 CAAACCTAACGTAAATTGATGGCTC 25 58.972 GTCTATCGTTCATCAACTTTGCCA 24 59.608 279

chr4 7214226 GTTTCTCCTTTCCCTCGTTTAAAAG 25 58.564 TGTCAGGAGATGTCAAGGGATCT 23 60.567 285

chr4 7216750 GGTTTGCATTCTTTTGGTTCATGAG 25 59.822 GGTTGCCCTTGTATATTGGAACTG 24 59.902 178

chr4 7216982 GGGCAAAATACTCCAACACTCAAA 24 59.961 TTTCTCCCCTCACATCATTCATCA 24 59.772 207

chr4 7219032 GGAAAGGGATTGAAAGAAAGAGGC 24 60.082 GAGGTTAGTCAAAAGGAAGTTGCC 24 60.023 222

chr4 7277848 ATTACCACCCATGCCTTCTCTTTT 24 60.511 TTTTCTTATACATAGGCCCACGGT 24 59.835 227

chr4 7281301 CAAAATGGCTATCAATGGACACGA 24 59.904 GCTGGGTTATTTGTCATTAGCCTC 24 59.963 226

chr4 7283710 TGCTCCACGATCTTGTAACAATTTC 25 60.107 TTCCTCTAAATCTAAACGGTCGCA 24 60.082 145

chr4 7287524 ACAATTCTATGTTGGGTGACGAGA 24 60.021 CTATCAAACGAATCACTCAACGCA 24 59.909 291

chr4 7289882 CCCATATTTCCACTTCTCTTTTGCA 25 59.814 ACCAAGTCTAAACGATCTTCTACCT 25 59.284 297

chr4 7293895 TCACTCCAATCAAGATCATCAGCT 24 59.836 TCAACAACAAGCACCATCTACAAC 24 59.964 266

chr4 7356879 GACTTCCATCTTTCTTGTACCATAG 25 57.222 ATCAACTCCCAAATCATTTCACCG 24 59.842 275

chr4 7384344 ACGTCTAGGACTTCACTTCTCAAG 24 59.783 CACTGCCATTCCATTTGTCTTTCT 24 60.022 155

chr4 7384637 AAGGGCTGTATTTTCTGGATAGCA 24 60.08 AAATTTGTGATGCTGACCGTACTC 24 59.846 288

chr4 7385966 TTGGAGTCTCTTTCTTTGATGGGA 24 59.649 ATAACCCAAGAAACCGTAAGTGGA 24 59.958 222

chr4 7448313 ATTGCGTCTTATTTCGGCAGTCTA 24 60.68 TCGATTGGGCTGGAGAAACTTTT 23 60.751 284

chr4 7449649 AAACCGAACATATGCCAAAGACAG 24 60.083 AGCATATGAGTCGGGAAGATGTTC 24 60.502 297

chr4 7451623 GAGTAGAAACAGCCAATAAACCCA 24 58.992 GCTTGAATACTGAAGAAAGGTGGG 24 59.843 229

chr4 7453147 GAGCGATCGAGAATTTACAACGTT 24 59.969 GTAGTGTTATGAAATGTGTTTGGGG 25 58.613 189

chr4 7454591 ACTTCGCCCTCAACTTATTCTCTT 24 60.02 CTACCTGAGTTCCTATGATGTGGG 24 59.96 243

chr4 7461110 TGGATAATCTAGCAACAACCACGA 24 60.081 CCCATTTCTCGTTTCTTTCGTTCT 24 59.787 96

chr4 7646809 TATTTGCTCCACCAAGAACTTAGG 24 58.747 ACACCCCGAGATTCTTTAAGATGA 24 59.53 243

chr4 7651296 AAAATGGCCGAAGAAGAGAAGGAA 24 60.75 TGTTCTCAATCACATGCATACTAGG 25 58.949 250

chr4 7654598 CTCTTGTCCCCTTACTGTTCTACC 24 60.082 ACCCTAAACAGAGAAACTTGAGGT 24 59.587 253

chr4 7656320 ACAGTAAAACGTTATGGAGGCAAC 24 59.785 CAGTTTCAATCGGTTCTCAACTCC 24 60.084 188

chr4 7657157 GGGAAAGAATATGGCCATCAACTC 24 59.721 CCCTCTATAATTGAATGGTTTGGGG 25 59.46 266

chr4 7658535 ATGATGGACCCAATCTCTATCTCC 24 59.218 ATAAAGATGGGGCGTTTGACAAAG 24 60.082 295

chr4 7664576 CTGGAGGTTTGTATTTCATGAGGC 24 59.903 CAAAGTTCGATTGTAAGGTGGGTC 24 60.083 156

chr4 7667179 GTTTTCCTCTGCCTTACTTCGATG 24 59.906 ATAAAGAGACCAAGGGATCAGAGC 24 59.898 135

chr4 7670824 TTCACAACACTTCTAAGAACGTGC 24 59.966 GTTTAGCGAGAATAGTTGAGGTGC 24 59.967 251

chr4 7679291 ATCCATTGACCATCCAAACAACAG 24 59.779 GAAGTCATGTGTTCAATTCGTGGT 24 60.024 177

chr4 7694158 ATAGGTTGGGGTGTAATTGGTTCA 24 59.955 GTCTCATCTTCTTTCTCATGGGGT 24 60.081 244

chr4 7699822 TCCGGTAAATTCTTCCACTTGACT 24 59.959 TGGACCGTACACAATCATTGAAGA 24 60.263 255

chr4 7704581 ATGTGGGACTGGGATTTCTGAAAT 24 60.265 TCCCATTTATAAGCAACATGTCCTG 25 59.406 204

chr4 7704796 GATCAGACTTCCTCTCCCCTCTC 23 61.003 TGAAATAGTGTGCAAAGTCCATCG 24 59.846 271

chr4 7712888 TCCCGACACTTAAGTAGACTATCA 24 58.252 GGTTTGGTTCATCTTAAGGTTTGGT 25 59.93 183

chr4 7713876 TCAGCCAAGTACGGTATTGTAACA 24 60.021 GACAAAGTACACCGAAGTAGAGGA 24 59.783 256

chr4 7715718 GATGATAACTTACAACACGTGCGT 24 59.909 TGGGGTCCTTTACTATAGCTTTCT 24 58.714 158

chr4 7718197 ACTTCTTCCCTATTTTGGCCTCAT 24 60.017 TTGTTGTCCTTAATCGCCAAGTTC 24 60.024 148

chr4 7723322 TGGAGAGCACGAGAAGGAGA 20 59.962 CGTGGTAGTTGATAGTTGATAGTTG 25 57.549 300

chr4 7728409 TTGTGGGTGTGAGAGAAAGTGTAA 24 60.081 GACATGCCCAATCAAAACTCATTC 24 59.134 298

chr4 7734252 CATGGTTGGATTGGATGGATGTTG 24 60.442 ACACGTTATCCATATTCACAGGGA 24 59.592 220

chr4 7780927 CACAGTCAATCCCATCACCAAATT 24 59.779 CTAACTTCAATGCCGAGTCATGGA 24 60.919 298

chr4 7788687 CCTTTACTTTCCCCTCCACACTTA 24 59.958 CATCCTTCTGTTTCACCTCACAAC 24 60.024 274

chr4 7794029 GGGAGTTTAATTTTGGTGTGTGTG 24 58.953 GTTGGAGGCTTGAATTGGATTGAA 24 60.021 229

chr4 7800842 CTACACCTAGGACACATCTCAACC 24 60.142 GAATGATGTTGTGTTGATGTGCCA 24 60.559 220

chr4 7806812 AGATGGAGAGAATTTTGACAGCCA 24 60.264 TGGAATCTCTGGAATTCTGCTTGA 24 60.019 265

chr4 7810765 TCCCTTTATCCAACCACTCAGAAG 24 60.019 AAGAATGATGTGCGACTTCCTTTG 24 60.083 246

chr4 7819864 CCAGAAAATCAAGCCCACAAAAGA 24 60.202 CTGTGTTGTGGAGTTTGTAGAGTG 24 59.729 242

chr4 7821591 TATTCACCACAAGCAAGGAGAGAA 24 59.958 GTTGCATCAGAACGGTTATAAGCA 24 59.906 129

chr4 7823825 TCCGATGAGCAGTATAAGGAAGTG 24 59.962 CACTCCAGAACACATAGCAGGTAT 24 60.142 294

chr4 7823987 TGAGATGATCCTTGTATCCTATGCA 25 59.218 GTGGAGTAATCGTGACGCATTTAG 24 60.027 228

chr4 7824432 ATTGAAGTGAGGACAACATGGT 22 57.896 GGAACCCTCTACAACGAGTTACAT 24 60.082 216

chr4 7833153 GGGGAGTTTAGGTGTTCATGTAGT 24 60.02 CAACTACACCATTGATGCACCTAC 24 59.905 189

chr4 7833269 ACCAACTTCTATGCCTCTTCTTTCT 25 59.986 CAACTTCAAGAACAACACATGCTTC 25 59.769 293

chr4 7868499 ATTTGAGTGAGAGGTGGGAGATTG 24 60.324 ACGAACACTCATGACTCAACTACA 24 59.962 198

chr4 7868950 TGAACCAACTATCCAAGACTATGCA 25 60.046 GTGAACCCAAACGAGATTATACAAG 25 58.228 264

chr4 7871995 ACTCAAGCTTTCTCTCTCTACTGC 24 60.082 TCATGTGTTGATACCTCCTCTTCT 24 58.974 139

chr4 7876068 CAAGCTCCTCTCCAAGATATCTCC 24 60.021 CCGACCGAACTCTACGTACAAATA 24 60.202 230

chr4 7878316 CTTGGCCTAAACGCATGATTCTAG 24 60.025 CCAAGTGTAGAGAACGATTCAGGA 24 60.082 265

chr4 7879418 CCATCTCGACTAATAATTACCCTTCCT 27 60.039 CCCAACTTTATCCCTATACTATAGACCC 28 59.903 270

chr4 7880400 TGTAGCTTTTGATCTGTAGGTCCA 24 59.469 TCTGCGATGCTAAAACATGAAAGG 24 60.143 286

chr4 7882697 CCCATAAGGCAGACAGATGTTTTC 24 59.903 GCTGGACCAACTATCTGATCTTCA 24 60.142 294

chr4 7890070 TCCACCTGTCCTTTTCTTATTCGT 24 59.959 AGGAGATGAGGATGGGAATGAAAA 24 59.52 127

chr4 7891015 TGACGGTTATTTAGGTGGTACGAT 24 59.598 TCTCTTGCACTCTTAAACCTTCCA 24 59.897 267

chr4 7893827 ACCCAAGTTCTGCTACCACTTATT 24 59.957 GGTAATTTTCGATGGGTTTTGGGT 24 60.021 228

chr4 7894241 CTTAAACCCTGAAACAAACGCTGA 24 60.202 CCCCTTCATCTTCCTCCTTAGTTT 24 59.772 291

chr4 7897817 TTTCTCTCTCTTTGCCGGAACTAA 24 59.96 GTGCTTTTGTCGAACCACTCTAAA 24 59.965 103

chr4 7901369 GGGTAAATGCGATGAAAAGGGAAA 24 60.082 ATGCCATTTTCCAAGAACAGGTTG 24 60.504 221

chr4 7901534 CAACCTGTTCTTGGAAAATGGCAT 24 60.504 CTCTGTAATTGAGGTGAAGAAGAAG 25 57.406 276

chr4 7905335 AGTTGATGTGATTTGACAAGGCTG 24 60.023 TTTTCCACCACTCTTTACGGTTTG 24 59.903 148

chr4 7906022 TCTGAAAGGAGAACAAGTGTGGAA 24 60.081 AGAAAGGTCTCTGTGTTACTAGGC 24 59.779 256

chr4 7906452 CTGTTTCGCTTATACTTGGGCAAT 24 59.904 ACTCTAGTACCATGGACCTCTTCA 24 60.017 287

chr4 7941473 TTGGGTCTAAGGGGAATGCAAATA 24 60.017 CTGAAGTTTGTTCGATTGTGTGGA 24 59.965 157

chr4 7942678 CAATGGCATGTTTTGGAAGCAATC 24 60.143 CCTTCCTTCAAATTTCGGGTGTTA 24 59.479 220

chr4 7942816 GCTAGAGATGACATCAATTGCTCA 24 58.945 CCTATTTTCCTCCCTCTAAGCCTC 24 59.959 226

chr4 7949801 GAATGAGTGTTTTGGTGGACTTGT 24 59.902 ATCCAAGAAGACCAGTTTGAGACC 24 60.506 171

chr4 7981469 TTAACGCCTTTACTCATCATGTGC 24 59.906 AGAGTTCAAGCTAAGGGACAAACT 24 59.897 292

chr4 7983030 GATACAAATGCAATGATCCAACGC 24 59.558 ACTCGGTCTTGTCCCTAAAATGAT 24 59.775 282

chr4 7996030 AGAAGTAATGTAAGACTCGTGCCT 24 59.537 ATACAATCGGCGAATCCTCTTACA 24 59.961 130

chr4 7997870 GTTGGCAAAATTTGAACCCTATCG 24 59.375 AATGCTTCACCTCTTCAAACGAAC 24 60.261 158

chr4 8005309 GCAACAGGAATGGAGAGAAAGAAG 24 59.844 CACTTGCTTCCATTCCAATCACTT 24 60.022 267

chr4 8008252 ATTGGCTTATGTACCCTGAGAGTG 24 60.142 ATTTCTTCCACACCTGAGCATTTG 24 60.022 221

chr4 8009893 GTGCATTCCAAGAAGTTTTGCTCT 24 60.5 CACAGTCTAAATGCATCCCTTGTC 24 59.905 295

chr4 8011228 GGCAGTGGAAAATTCTAAAAGGCT 24 60.021 TTCCATTCCAATTGATTCCCGAAC 24 59.84 195

chr4 8016711 CTGTTTTCTCTGAGATTAAGGTACC 25 57.164 CACACATATTATAGTTGCAGTGAGACC 27 60.042 286

chr4 8018160 ACCCACTAGAACAACTTCCATCAT 24 59.71 CAGAGCTCCCATGTTCCTGATATT 24 60.202 300

chr4 8027157 AGCTTAAACGCAAACTTCTTCTCC 24 60.024 TGATCGTGTTGACAATGCTAAACG 24 60.377 286

chr4 8043571 CTACAGTGATAGTGATTGGGGAGG 24 59.96 AACATGATGGTCCCTTTCTCTTGA 24 59.957 234

chr4 8045508 AATGCATCACTACGTACCTGTTTG 24 59.608 TCCTCTTAAAGTTATCCACCCTCA 24 58.653 235

chr4 8052558 GATTACTTGAGGCATTGAAAAGCG 24 59.205 TAATGAAGTCCCACAGTCCTGATG 24 60.081 213

chr4 8058131 TTTTCCATTCCCTTCTCCCATCAT 24 60.016 AGAGAAGAATCACACAAACCCCTA 24 59.402 216

chr4 8059099 TTTTGGATCTGAGGCTATCTTGCT 24 60.08 TCTCGACCTGCTGCATATGATTTA 24 59.961 288

chr4 8064537 AGTGGATTGCTATATTGGCTCT 22 57.01 TGGGCTTGTTGTGTGTTGAAAG 22 60.094 299

chr4 8065129 CCTAAACTCATAGCCCTGTACACA 24 59.838 GTTCATGCAAGTTTCTTACGAGGA 24 59.549 260

chr4 8066890 AGTGATTCAGCTACTCTCCTTCAA 24 59.225 CGAGAATCTGGTAATTTCGTTCCC 24 59.73 284

chr4 8074073 CCTAAACTAACCTACCAACAACAACC 26 60.017 GAGGATGAGAGAAGAGAGCAGATG 24 60.023 291

chr4 8074241 AAATTAGTCAAATCGCCACCACTC 24 59.844 GAGGATGAGAGAAGAGAGCAGATG 24 60.023 263

chr4 8075880 CCGAACGATGATTCCTTGCAAATA 24 59.965 TCACTCCTCATGCTTCATCTTTGA 24 60.02 141

chr4 8076263 CCAGCTCTTGACTTGCATATTGTC 24 60.202 CTTCTTGCCATCTCCCAACAAAAT 24 60.021 153

chr4 8080207 AATGCACGTCCATAATGTTACTGC 24 60.202 AAGGAGCTAGACTATTTTGGGGTG 24 60.08 245

chr4 8085334 GCAAGCTTTACCTCAACCTTCTTT 24 59.962 TTACTATTGACGCTGAGTGGGAAA 24 60.021 285

chr4 8085938 ACTAACATGCGAAAAGTGTACTGC 24 60.084 CACTCAACATCTACTTCTTGTCGC 24 59.909 141

chr4 8090854 TTTCCCAAAATTTCCCCATTTCCC 24 60.203 AGTTCTCTAGTGTGTCAAGGTTCA 24 59.35 217

chr4 8109431 AACAAATAAAATATCGACCGGGCC 24 59.963 GTTTTAGGGAACATTATGGGGCTG 24 59.901 175

chr4 8110541 CTCTTTGCTTTCTCCAATCTGTCC 24 59.844 TTGCATCCCTTGGTTCCATATTTG 24 59.838 154

chr4 8118874 CCTCTTCCCTTAATATCCTGCGAT 24 59.776 CTTTTGCGAACTACCCACTCAATT 24 60.023 255

chr4 8120320 AAGAAGATTGAAGGATTTGCGACG 24 60.143 CACGATGGTCAATGTAACACAAGG 24 60.378 207

chr4 8134185 CGTTCAGTTTTCTCCTCACATCAG 24 59.849 TTCCAGTAATTTTCAGTCCCCGAT 24 60.019 275

chr4 8134900 ACCCTAGCATCGAAATTTGTCCTA 24 59.837 CAGTAGCAAGACCCCTTTAGG 21 57.732 184

chr4 8135513 AATAGGTGTTTGGGTTTAATGGGC 24 59.776 CCATCAAGAAACGAAGGACCAAAT 24 59.782 282

chr4 8136483 TGAGGTTCTCTGTTATGGGGTCTA 24 60.265 GCTAACAATCAACAAACCGACTCT 24 59.787 178

chr4 8142603 AAACAGCCCAATTAAGCCAACAAC 24 60.982 TGACACCTAACCTACTAAGAGTGA 24 58.116 254

chr4 8219150 AATGATCTTGAGACACGATGTTGC 24 59.906 CAACGAGCTTTCATACCGGTTTAG 24 60.202 240

chr4 8250198 GATCTTGGAATGGGGTGTGACA 22 60.291 TCTGTAAAGTTGACGCCCCTAATG 24 60.862 157

chr4 8260559 AAGTAGACTAGTGTTGTGCTTAGC 24 58.529 CCAGTGAGCTAAAGGGTTATTGAC 24 59.364 240

chr4 8263634 ACCCTCCTAGAGATTATTACAAAGGTC 27 59.71 GCTACAAATTTGAGGGGAGAGAGA 24 60.081 277

chr4 8264666 AATCCTTAGGCGTTTTGATCACTG 24 59.604 TTTGACTATATTTCCAACCGCCAC 24 59.603 219

chr4 8276633 CCTCTTGTCAGTCTTTGCATGTTT 24 59.963 TGGAAGAGTCGTGGTGATCATATG 24 60.202 176

chr4 8359599 TAGTAAGCGTTTTGAGGTAGGCAA 24 60.263 TCCGGGACATGAGTTTTACAAGAT 24 60.02 215

chr4 8361952 AGACCTTTAAGTGAGAACAGACCA 24 59.346 TTCGTCCAAGGAATAGTTCAAGGA 24 59.715 288

chr4 8379284 GCCACTAACCATATTTGCTTCTCA 24 59.36 CCTTTACTGAAAACGAACGCTTCA 24 60.26 272

chr4 8380015 CAGATCGAGGTGAAGAATATATGGAC 26 58.967 CTTACGGCATCTCCTTCAACTCTA 24 59.902 260

chr4 8382285 CCCTGGTTGCCTAAAATTTTCACA 24 60.202 ATTCACCGCTAACCATCTTACAGT 24 60.081 246

chr4 8404548 TTTGCTCTCTCTCGATGTTGTTCT 24 60.262 TAATGTCAACAACCTGGGCAAATG 24 60.262 284

chr4 8495885 TCAGATTAGACATCTCTTCGCCTG 24 59.963 CACGCCTTATATGAGACTTGTGTT 24 59.13 201

chr4 8496068 TGACCCTTCTATTCCAAACACAAG 24 58.93 CCGCTTGACTTGGAAATTCTCTTT 24 60.023 300

chr4 8522939 ACACAAGGAACCTAAGCTCTTACT 24 59.406 ATTAAACTCTTCCTTCCCTGCCAT 24 60.017 198

chr4 8542512 CTCATTTCTTCTTGTGGGTGATTTC 25 58.615 CGAAGCACAACTGATTTTAAAAGGC 25 60.108 207

chr4 8543307 GCCAAACCATAGAGAGGAGTGAAA 24 60.566 AATCTTTCCTGCTCAGACCTTCAG 24 60.565 265

chr4 8554180 CATTACCACCAAGCATCCTTTCAG 24 60.142 AATGGTCTAGCTACACAAGAGGAC 24 59.839 259

chr4 8561938 TCCATCCGCATACTTTACTGACAT 24 59.899 GATACGTACCATCTAGTGACCACA 24 59.422 291

chr4 8563987 TGCTCGGTAGTAGAAGACAAAACA 24 59.962 GAGCAAGGTAGGGATTGAGGTAAA 24 60.081 147

chr4 8567764 TTGAAGTGCTGAGAAGAGGAATGA 24 59.959 TGTGTGGTTTAATAGAGAGTGCAAC 25 59.531 175

chr4 8640952 ATACTGTAAAACATACCGCGGAAG 24 59.193 CTAGCAAATGACAAGAAGTGACCG 24 60.143 85

chr4 8642722 AAAATCAACCTCTTCTCCCTCCTC 24 60.019 TGTTGAGGTTATGGTCGATGAAGT 24 60.021 242

chr4 8692176 CTCTTTCAAGGAATATTGCAGCCC 24 60.202 ATACACCAGTGCAGTAGAAGGAAG 24 60.081 196

chr4 8693142 GCATGCTTCTCAACCTCTAGTACA 24 60.382 TCCAATCAGTTCACTTCATCGTCT 24 60.021 271

chr4 8740035 AAAGAGAAACAGATCAAACGGCAG 24 60.024 GCAGAGAAAGCAAAGAGTGTGAAA 24 60.202 297

chr4 8750416 AGATTTTGAAGCCGCTAGAAATGG 24 59.903 CTCCATTAAATTCCCAAGCCCATC 24 59.96 233

chr4 8775049 CACAGTGGTCATAGAACGTAACAA 24 59.014 TAAGTCCCTTAGGTACCCATGCTA 24 60.078 182

chr4 8775234 TCCTATGGAACTAGCATGGGTAC 23 59.098 TGTTCATTAGAGGAATCAGTGGCA 24 60.019 300

chr4 8778664 CACATCTTCAGCATCTTCGACAAG 24 60.202 CGATCAAGTTGGTTATGGTAAGCG 24 60.26 282

chr4 8779483 CTAGATAGCATGTGTTCGAGAGCA 24 60.261 TGAGCCTTATCCATAGTGTTACCA 24 59.034 294

chr4 8780834 CGGTAAGATATGCGAGAGATATTCA 25 58.214 GATTTGTAAGAACCAACTGCGATG 24 58.911 266

chr4 8782609 TAACATCACGTCATCTCTCGAGTC 24 59.966 ATGGTCACCGTACGAACTTTTCTA 24 60.022 107

chr4 8786274 AACCTGACTTTCAACCTTAAACGG 24 59.663 CACTCACCAAATCAATCTAGGTATGTC 27 59.774 241

chr4 8793751 CTAGTTTGGACATGTTTGGTTCGG 24 60.32 GCATGAAGGAGAGTGTTGAATGAT 24 59.361 299

chr4 8815469 GGTGAAGCGATGAAGTTTATGGAG 24 59.965 GGGCCGGTAAGTGTGTGTATATAT 24 60.021 250

chr4 8816477 CCCGAAAATATGGATTGGGTTGAA 24 59.596 TACAAAAGGAATCGCGCACAAA 22 59.709 264

chr4 8818632 CCGGCTGTATGGAGTAGGTAATAG 24 59.842 AAGAAGATGAAGGATTTTGGCTGC 24 60.082 276

chr4 8819532 ACGAGAATTTCCATTTACACCCTC 24 59.058 GCAAATCAAGAGAAATGAGGGAAAC 25 59.134 300

chr4 8825838 GCACCTCTTTACTACTTTCCACCT 24 60.263 AAGAGTTTGTTGAGATGCTTGTCG 24 60.025 225

chr4 8830921 CCATTTTAGGTGTCATTGCTCGAG 24 60.202 GGGAAAATGAAGGAAAGGAAAGCA 24 59.96 288

chr4 8837970 CAATCTGTTTCGACGGTTTCTGAA 24 60.025 TAACCAATCCCCATTTCACTTTGC 24 60.021 298

chr4 8843395 TCTCACCCATACAAATCAAGGACA 24 59.712 TCGCCTATTTTCTCTCTCTGTTTTC 25 59.13 280

chr4 8844578 AGTTGTTCCAGAGGTGAATGACTT 24 60.141 AAGTCAATAGAACACCACAGTCCA 24 59.897 199

chr4 8860828 GGAAGGCAGAAGATCGAAACAAAA 24 60.023 CCGTTCTGTGCTTCTTCATTCATT 24 60.083 246

chr4 8872421 AGAAAGTACGCATCAATACCGGTA 24 59.902 CCAACCATGAGATATAATCCAACCA 25 58.694 125

chr4 8872643 TGGTTGGATTATATCTCATGGTTGG 25 58.694 TTTGAAACCCCAACTTCTCTCT 22 57.48 293

chr4 8905097 GTGAAATGAGTGACACGTGACATT 24 60.025 GTGAAATTGGACGGCTTAACTCAA 24 60.024 298

chr4 8936874 GCGTTTACCATACTCTTTTGCTGA 24 59.847 TGATCTTTGGTACCCTACACGATG 24 60.142 290

chr4 8937123 AGCATCCCACACTAGAACACATAG 24 60.142 CTAAGAGCTCCACAAGAAGAAGGT 24 60.021 261

chr4 8967556 GGATAAAGAGGCAGCAATTTCCAT 24 59.657 ACTCTCCCTTAGGCTGATTTGAAG 24 60.081 215

chr4 8968684 ACAACACGAGAAGAGATAAGGCAT 24 60.081 CTTAACCTTCCTTTCTAGTGGCCT 24 60.019 237

chr4 8977510 GTTCACTACCTTTGTCTAGTCCCA 24 59.717 ATATCCGAGTAATTAGGGCGCTTT 24 60.02 261

chr4 8978785 GGTGAATCTCCCATAATCTCCCTA 24 58.906 TATTTTGTACATCCCTTCGAGCCA 24 60.081 169

chr4 8979603 GCAACGAGAGAAAAGGTTTCTGAA 24 59.965 ATTTATTGAAGTGGGCCCCTTTTC 24 59.775 269

chr4 8980484 CAGGGATTGTGGAATTATTCTGGC 24 59.962 GGCACCTTAAGTCTGTTGATTCTT 24 59.238 160

chr4 9006419 TCTCTTCAACTTCCTTCCCATGTT 24 59.895 ACCACAACATCAATCCATCTCGTA 24 60.081 228

chr4 9006671 TACCAACTCTCCTATAGCAGCCTA 24 59.894 AGAGTAGGCCCTGTTGCTTATAAG 24 59.898 276

chr4 9011514 AATAACCGAACCCATTTTCCCTTG 24 59.778 CAAGAGTTGGGAGAGCTTTATAGT 24 58.016 298

chr4 9016269 ACAGTCCAGACACTTTACTCCAAG 24 60.202 CTCTGCATAGGTTCATTTTCTGCC 24 60.202 152

chr4 9027050 TCACCACAGTCGGAAGTAAAATCT 24 59.96 CGTTCACCGTGATTCTCATTCATT 24 59.907 277

chr4 9027335 AACCCTATGAAGCGACAACAAAAG 24 60.023 CTTCTCTTCTTCTTCGTCTCCCTC 24 60.142 238

chr4 9028971 ATCACCTTCAACGAGCTTCTTGTA 24 60.262 CCCGGTTCTTTGTCTAGAGGATAG 24 59.962 297

chr4 9319042 ATCCACTCTACTTCCTGGGTTTTG 24 60.264 CAATCCATGACCTCTTTACTTGCT 24 59.054 297

chr4 9322314 TGACCATGCCTAGATAAACTCCTT 24 59.278 GCCAATGACCTAACCAAAAGTAGT 24 59.235 273

chr4 9322574 ACTACTTTTGGTTAGGTCATTGGC 24 59.235 AGACTTCAAAAGAGCTCAAGGTGA 24 60.142 204

chr4 9332174 CCCTAAAGAATTCAGAGACGTGGT 24 60.323 CGTAGTTTTAGAGATTGGCTTCCT 24 58.58 211

chr4 9334340 GCACGTCCCTTAATCACAAAATATC 25 58.801 TCTCTAATGAAATGGTGAGGTGGT 24 59.464 288

chr4 9338004 CGAGTAATTTGGAAGCAACGAAGA 24 59.848 TCCAGTTCCAATTCCAACATCTCT 24 59.957 268

chr4 9338809 GTCAAAGCTTAACACCTCTACACG 24 59.85 TCTTGCGGTGTATGTTTCTTTGTC 24 60.024 290

chr4 9354165 ATGTGGCCGATTAATGACATGTTG 24 60.202 GCATGTACACACCTTCAACCTTTT 24 60.202 299

chr4 9361869 GTACTTTTACTTACGGGTTTGGGG 24 59.544 GTAGATGGTGGTGGTCAAGTAGTT 24 60.02 293

chr4 9362242 ACCGATATCTATGCTGGCGTTATT 24 60.082 GAGCCGCAAACAACCTTATAATGA 24 59.904 224

chr4 9366515 AAACTGGGATGGACGAGAATAACA 24 60.02 GCATTTGTCTGTATGTACGTGGTT 24 59.847 278

chr4 9374121 AATCCAAAGTAACTCTGCAACAGC 24 60.023 TCTGGGTATGGGGTTATGTTTGTT 24 59.955 165

chr4 9471754 CACTTAAGTACCATGTGCTGAACG 24 60.143 TAAAGGAGAGTCGGGTTGTAAGTG 24 60.022 283

chr4 9471863 CACTTAAGTACCATGTGCTGAACG 24 60.143 TAAAGGAGAGTCGGGTTGTAAGTG 24 60.022 283

chr4 9527234 GATGTTTGTGGTTTACGGTTTGGT 24 60.44 CCCTAACCCCTAAACTTCGAAACT 24 60.263 292

chr4 9627142 AAACAAACTTCATCCGTATCGAGC 24 59.907 TCTGTGGGCAATTAAAATGGGTAAC 25 60.047 294

chr4 9678684 AAACCAGCCTATTTCCTTTGCTTC 24 60.021 CTCAACCCTCTCATCTAACCACAA 24 60.02 266

chr4 9740018 AGAGCTGTATTTGAGACTCATCCT 24 59.038 ATAGTTTGTGATTTGAGGTGCGAC 24 59.846 222

chr4 9758144 ATTGCACGACTTCCTCTTACAGAT 24 60.081 TGAGTTGGAAGCTATTGGAAGACA 24 59.958 276

chr4 9760835 ACATCTTCAAAACCCAGCAATAGG 24 59.536 ATTTGAGGATGGAATTCAGGGCTA 24 59.831 187

chr4 9763142 CATGCAAAATCGTATGTAGTTGGTC 25 59.035 GGTCAAAATGATCATATTGCCCTTG 25 59.013 188

chr4 9770979 TTGAAGGAAATTGAAGTCTGGTGC 24 59.962 AATTCCCCAATGAAACCTTCGAAC 24 60.021 265

chr4 9796525 TGATACAAACTTCGAGGTTCATGG 24 59.065 TGGAGAATACAGAGAAAGACGAGT 24 58.989 189

chr4 9801298 TTTAGGTTGCAACAACAAGGGAAG 24 60.142 CCATCTTCAACTTCATCCATCTGC 24 59.964 238

chr4 9805221 CAGTGCACTTTGGTTCATTGGATA 24 59.782 CTTACTTTTCCTCTCCGAGTGTGA 24 60.022 226

chr4 9807878 TTTGTAGTTTGGGTGGCAATTCTG 24 60.202 AGAAACCGAGAGTCAAAGGATCAG 24 60.322 131

chr4 9809599 TCGAGTCAACCCAATCCTTTACTT 24 59.959 GACCAACCCTGCAGATTAATCAAC 24 60.142 126

chr4 9820047 TGAAATGAAGAGAGGATGTTGGGT 24 59.957 CGTGATTTGTGACATGTGTATCTCA 25 59.652 251

chr4 9836182 TGTGTTTTGATCTTAAGTTGTGGGG 25 59.932 TCACATTACAGCTACGATGGTCAT 24 59.901 262

chr4 9852686 ACAAAGGTGGTTATAGAGGAGGTG 24 59.775 AGTAGTGTCTTCAAAGGTAGTGGC 24 60.263 210

chr4 9855811 GTGGACCAACAATGAGAGAAATCA 24 59.238 TTTTACTAGTCGGTCCCCAATGTT 24 59.958 190

chr4 9863879 TAGCTGGAGGATGTGTGTTGATAG 24 59.9 TAGAGAGACCAAAAGGCATCACAA 24 59.958 243

chr4 9869163 ACATGCGTTAGAGGGTAAGGATTT 24 60.081 GAAATATTTGATGTTGGCGTGGGA 24 60.142 289

chr4 9872299 TCATATGAAATTGAACCGAACCGC 24 60.202 TTTCTGGAACTTAGTGCTTTGGTG 24 59.662 253

chr4 9872620 CACCAAAGCACTAAGTTCCAGAAA 24 59.662 CGAATCGGACTGCTTCATTTAA 22 57.142 280

chr4 9884761 TTGGGAGTGAATTAGGAGGAAAGC 24 60.568 TGGGGCTAAATTTAGGACACTTCT 24 59.709 199

chr4 9885019 AGAAGTGTCCTAAATTTAGCCCCA 24 59.709 TATGTTTGGTCCCCGTACTTTCAT 24 60.019 227

chr4 9898876 AGACGAAAGAGAAGACAACCATGA 24 59.961 CATGTTTTCAGTCCCTTTTCCAGT 24 59.658 80

chr4 9902263 AAACCCAACTCATCCCTTTTGA 22 58.078 CCCGATGGAAAGAAAGGACTTGTA 24 60.565 187

chr4 9902596 TACAAGTCCTTTCTTTCCATCGGG 24 60.565 ACCCCATTTCAACCATCCAATAGA 24 60.017 270

chr4 9903039 GAAAGAAAGACCTCACTCTGCGAA 24 61.034 CCTACTTCTGCAATCTCTACCTCC 24 59.961 275

chr4 9910568 GACGTCCTCAAAAGAGCTTGTTTT 24 60.202 TTTTCTAAATGGGTGTTGTGGGGT 24 60.88 184

chr4 9919550 TGCATGTAGTATGATGACTCCTCC 24 59.718 TATTTAATCCAACTGAGGGCTCCG 24 60.444 234

chr4 9921884 TTCAAGAGCAAACCCGATCCTTTC 24 61.579 AGCAATGAAGGAGTAACAAATGTGG 25 60.048 299

chr4 9922769 CAACATTGGCCAATTGAGATCCTT 24 60.081 AGTCTTCAACACCATCTCCCAAAT 24 60.203 248

chr4 9931237 ATTAATTGCACTGAGATGGCTTGG 24 59.901 GATCTCTAGGGATGGGTGCATTTG 24 61.046 300

chr4 9943252 TGAAGGGAGATATGGAGGAAAAGG 24 59.586 AGACCTTCTGCCAAGTTAGTCAAT 24 59.958 295

chr4 9981537 TCCTAGCAAAAGAAATTCTCCCCT 24 59.708 CTCCCACTTTCTCCAAATCTCTCA 24 60.02 224

chr4 9982230 AGTCCTCAGGTATTTCAGCGTTAG 24 60.142 ATTTTAGCTGGCTTGAAAGGATCG 24 59.903 297

chr4 9984823 CAGAGGTCCAGGTTCTATGTTCAA 24 60.02 GAAAGGCTCAACAAGTGGAATCAA 24 59.962 143

chr4 9984923 TTGATTCCACTTGTTGAGCCTTTC 24 59.962 TCTCAACGTGTTACTCCTTCTCAG 24 60.023 195

chr4 10000829 AAAGGATTGGTCAGGTTGTCATCA 24 60.448 ACAATTTTAGCCCAGTCAACGATG 24 60.083 293

chr4 10022562 TTGGGGTGCACAACGGTT 18 60.044 ACTCGCACACATGTCAGACT 20 59.324 300

chr4 10028241 CCGCTTATGGGTTTTGTGAGATTT 24 60.082 TTCAACATGGAAAATTGGCAACCC 24 60.986 294

chr4 10044630 AAGGTGGGATTAATTGATTTGGGG 24 59.279 AACACCGAATCTACATCCATACGT 24 59.901 273

chr4 10063343 GTCCTCTCTTCTTCGTCTTCTTCA 24 59.783 GGCTTGAGGATGGAAATGTCAGAT 24 60.931 211

chr4 10081141 GTTCCACCCTCAATTTGACAAACA 24 60.142 GAAATATCAGAGCGCACTTCACTC 24 60.026 254

chr4 10144050 TGAGCACAAATCTAACACCTCA 22 57.65 ACTGCTACGTTAAGAAGAGAAAGC 24 59.076 269

chr4 10216690 GCGAAAAGAAAGAAACTGCAAAGG 24 60.027 TGTCCGGTAGATTCCACGATTAAT 24 59.657 291

chr4 10306945 GCGGTTGAAATTTGATAGACATTGG 25 59.484 GTACAAGTGAGCAACAGATATCAGT 25 58.897 280

chr4 10371872 TTCTCTCCACTTTCCTGGCTAAAA 24 59.896 CTGGCTCACTTCTTTTGGGATCAA 24 61.29 297

chr4 10379648 CAAGTGATTGTAAGTAGGATGATGC 25 58.047 ACGATGGCAAGTCTATGAACT 21 57.105 215

chr4 10398057 AACACCAACCAGATTTACGGACTA 24 59.959 TTATGCGATGTACTAAATGGTGCG 24 59.79 267

chr4 10405106 GGTAACATTAGACTGTGCTTGACA 24 59.005 CAAATGCCCCATAAACTTCACCAT 24 60.081 264

chr4 10415358 ACAGATTCATGCTTGGGAGATTGA 24 60.325 GCTTACCTATCTCACTACTTGGCA 24 59.9 250

chr4 10415615 TGCCAAGTAGTGAGATAGGTAAGC 24 59.9 GTTGAAACTACCACTAGCGTTGAC 24 60.085 294

chr4 10415739 TGCCAAGTAGTGAGATAGGTAAGC 24 59.9 GTTGAAACTACCACTAGCGTTGAC 24 60.085 294

chr4 10431794 TTCCACCACTGATGAAGATGAAGT 24 59.958 TTACAAAGCTAGAAGAAGAGGGGC 24 60.324 207

chr4 10433730 TCCTAGTTTGTATAGTGCCCCAAC 24 60.081 CGTAAGTTGAAGCTATGCCCAAAA 24 60.083 190

chr4 10473617 GTTTATCTCCTCAACAACACTGCC 24 60.083 TTTTGTGTATCCGTGAGTGTCTGA 24 60.202 295

chr4 10479400 TTTGCATGCTTAGCTTACCTTGTC 24 60.083 CGTTGAAAGGAAGAAGACCACAAA 24 59.904 285

chr4 10502939 AAATAGGGAGGGAAAGGAGAGAGA 24 60.015 CCCCTCTCTTCTCCTTTTCTTTCA 24 59.958 88

chr4 10569944 TCCCATCATCATGTATTAGTCGCC 24 60.323 CGATATGAGAACGAAGCATGCATT 24 60.084 236

chr4 10584747 ATGCTTCCCAATGAGACCTAAACT 24 60.018 GGATTTATCAAAGGGCATTGCGAA 24 60.441 275

chr4 10592738 TCCCCATCCTTCTTCAGTATTTCC 24 59.834 TATCCTCTACTGCATTCTCAACCG 24 59.962 237

chr4 10600194 GCAGAGAAAAGCATAAGTTGAAGGA 25 59.818 ATTGATTGACAGCACATTGATCCC 24 59.901 198

chr4 10606362 GGATGGGATGGGAGAATAGAAGAG 24 59.775 GTCAAAGCATGTGGGAAGAACAAA 24 60.441 266

chr4 10612218 TAAGTTTACCCGTAGTGCCAACAA 24 60.444 AATTAATGTGGGGATGGGAGATGG 24 60.45 247

chr4 10612574 CTTTCCTTCAAGAGCAACACAACA 24 60.142 TTTGTTGTTGGGAGAGAGAGAGAG 24 59.96 132

chr4 10638002 GACTCTCTACTAGCCGCTGTTTTA 24 59.904 GAACCCAAGTAACTCAAACGCTAC 24 60.084 158

chr4 10663051 ACTCTGTGTGATAAGCTTGCTCTT 24 60.263 ACATTCTGAACGGATTAGCATAGGT 25 60.163 283

chr4 10663610 TCTTATTTACTTGCATGGGAGGCT 24 60.08 AATCTCTAGGCGGGCAAGTTTTAT 24 60.385 246

chr4 10820063 CAGGCAAACAAGGTTCAAACTTTC 24 59.67 GTCAAACTTCTTTATCAGGCACGT 24 59.787 258

chr4 10841503 CCCCACTATACGATTATGCTGCTT 24 60.564 TTTAGTGTACGAATCTGGGAGTGG 24 60.082 296

chr4 10953371 ATGTATTCCCCAAGCTCCATTAGG 24 60.203 CCTGTTTCTCTCTTTCCATGTCCA 24 60.506 299

chr4 10992375 GCTCACTTTTCCGATTGTCAGTTT 24 60.261 GGAGACGAAACTGATCCAACAATT 24 59.544 150

chr4 11893449 GGTCATCGGGTAGAATTGGTAAGA 24 59.9 ACTAGGTCCCAATTCTTTTGTCCA 24 59.894 255

chr4 12773056 CTGTCGCATTACACTTTCGCTAAA 24 60.143 GAATCATATTCGCACGCTTGGTAG 24 60.376 280

chr4 12847630 TTTCTCTAAATAAGGCGCACAACC 24 59.845 GGGTTGACAGTCATGCCTTTTATG 24 60.381 169

chr4 12881095 GATTTGTACGGGTGAGAGTGGATA 24 59.901 AGAACTTTGACTTTCTTGACGCTG 24 59.965 221

chr4 12958605 TTAACGCTCTTTTCTTCACTGCAG 24 60.025 AGATGTGCTACAGACAAGTCCAAT 24 60.02 216

chr4 13079914 TTCATAACGGCCCATACCAACTC 23 60.683 ACTGGTTCGGTTATGCAAGTTT 22 58.781 294

chr4 13082214 ACAACATTGACATAATCCGTACGG 24 59.431 GCACCATTTTATCCACGTATCGAA 24 59.728 159

chr4 13086055 AACCTTCCTAGACCACTTGACATC 24 60.02 TCTTGAAAACGAGCACAAAGAC 22 57.727 257

chr4 13089699 AGCTTCACTTTATTCCCTTCCACT 24 59.957 AATTGAAGAGAAAAGAGCCCTCAG 24 58.991 298

chr4 13118950 TGACATCGGAGGTTTTCTTCTT 22 57.648 TCTTTGGGGTGTTGAGTTCTCATC 24 60.747 274

chr4 13125666 TTGCTAGCACCTCCTTGAAGTATT 24 60.019 GCGAGGTAATGCGTTGAAGAAATA 24 59.966 194

chr4 13127783 TTTCTTAACCCAACCCAACCCTTA 24 60.079 CTGTAAAATCTTTCAATGCCTAGGG 25 58.2 267

chr4 13154376 TCTTCTCTAACTAATCCTTCCCCT 24 57.894 ACTACTTCCAAGCTTTGATACCGT 24 60.02 263

chr4 13162944 TCTTTCTCACAACAAACCACAACC 24 60.082 TTGCATCAATAGTGTCCCCAGAAT 24 60.325 269

chr4 13163322 TAGAGACATGGAAGTAAGGGCATC 24 59.655 CTTACCCCTGAAATGAGAGGCTTA 24 59.836 221

chr4 13164453 AAGACACCTACCCCACTAAGAATG 24 59.775 TGTTGACCCACTATTCTCCAAACT 24 59.896 294

chr4 13174673 AGCCAAACCAAGATACTTACGTCT 24 60.02 TCTTTTAAGTTGCTACCCTACCTAC 25 58.06 240

chr4 13518516 CACCGGAAACTGAGAAATGGAATC 24 60.143 AAGAGATGGGATGGATGCGTAAAT 24 60.203 277

chr4 13618504 TGGAAAGCAATGAAAAGAAGAGGG 24 59.718 GTGAGTGTGACAATCCAAAAGCAA 24 60.44 270

chr4 13631191 AGGTAAAGGGAGAGTAACAGAGGA 24 59.954 TGCATCCCTTTCTTTCTTTCTTCC 24 59.476 257

chr4 13647908 CGATTTGGTAGCCGTGACTATAGA 24 60.023 CTCTACCCATCACCTAAACACTGT 24 59.777 229

chr4 13666115 AGAAGTGAAGAGGTAGCTGTGTTT 24 59.898 GTTGGGTTGTTGGGTGATATTGTT 24 59.96 295

chr4 13675169 GTAAGCTCTAAGATATCCCTGCGA 24 59.54 TATGTTAGTGGTGCGTGCTTCTAT 24 60.142 276

chr4 13687599 GGATGCAATACCAATACCACACTG 24 59.963 TCTAAAGAGAGGAGGTTGAGAAAGT 25 58.921 188

chr4 13690361 CAGTTCAAATAATGGCCCCTGTTT 24 60.02 GGCATCTCTCTCGCAAATCTAAAC 24 60.025 288

chr4 13701858 TGTATAGCTGAGTAGACTTTGCACA 25 59.815 TGCCAAAGTATATCCATATTCCTCC 25 58.279 219

chr4 13720099 CTTTCTTGGTAGGATTAGGCAGGT 24 60.08 TCACATCAGCAAGGAATTGAAACC 24 60.022 254

chr4 13726563 GAAGTGTGTGAAGATCATCATCCA 24 58.819 GATCTTTCTCATCTTCGCCACATG 24 60.025 235

chr4 13734550 TCATGGGATCTAAGATCGGATTAG 24 57.218 GGCTTGCAATGTTTAAGTGTTCCT 24 60.501 246

chr4 13739996 TACAACTCTCTTCTACGCTCATGC 24 60.439 ATTGTAAACCGAACTCAACCCAAC 24 59.963 221

chr4 13740149 CTATGTCAACGTGAACATCCATGC 24 60.495 AACAAGGGAGAGAGGAGTATGAGA 24 60.017 235

chr4 13749707 TGGCTCCTTTCTATCCCTTTTCTT 24 59.708 TGATGATGAGATTGAGAACCCTTGA 25 59.81 217

chr4 13865151 CAACTCGGATGAACATATGGGATT 24 58.934 TAGCAGTCACCACTTGTTCTCTC 23 59.994 271

chr4 13866818 CAACCCAAAAGTCTCATTCCTGAC 24 60.023 CCTTACGCTCATTTGTATCCACTG 24 59.729 277

chr4 13889353 GCATGTGAGAAATTTGTTGTTGAAGCG 27 62.792 GGGGATCGATAGGTGACACTTAAT 24 59.716 228

chr4 13890963 TTGTTTTACAGAGGCCGTAGAGAA 24 59.961 AGGAATAGTAGGGAAGTGGTCTGA 24 60.016 90

chr4 13891076 ACCACTTCCCTACTATTCCTGAGA 24 60.016 ATACCAAGAGTTAGTTCTGCCTCC 24 59.837 180

chr4 13897278 GAGAAATTGTCACTCACCCTTTC 23 57.636 TGCTTTAAGAATGTGTCCACCTAGA 25 59.987 274

chr4 13897431 GAGAAATTGTCACTCACCCTTTCA 24 59.178 TGCTTTAAGAATGTGTCCACCTAGA 25 59.987 274

chr4 13899594 AGTACCATCATCATTCCCGTTTCA 24 60.081 GCAACAATCACAAAGGAAACCAAC 24 59.966 284

chr4 13901723 ACTACGTTCAGTTGCAAATCATGG 24 60.083 TCTTTCCGGTAAATACGACTGTCA 24 59.782 300

chr4 13907552 CAGCATGTGTTCGAGGACA 19 57.784 AGAGGAAAGCTCAGAGAGACCTAA 24 60.264 169

chr4 13941456 ACAGAATAAAGAAGAAGGGAGGGT 24 59.147 GAAAGTAACCTTGTTTCGCCGATT 24 60.32 270

chr4 13941794 TAATTTGTCGAAAGCCTGTGATGG 24 59.844 AGTGTGTTAGGTTTAATTGTGGCG 24 60.024 213

chr4 13953094 TAATAATTCCTCTTGCTGCCTCCC 24 60.446 GCTCTCAACCCTCTTTCCTCTTTT 24 60.748 293

chr4 13956918 ATGGTAGAAAGAGGGACAAAGGT 23 59.083 CCATTCGTATCTCAAATTGCACAGA 25 59.935 300

chr4 13957113 CTCCCATGGTCCATATCTCTTCTG 24 60.02 CCCCAGAGTTAAGTTTTGTTTGGT 24 59.596 207

chr4 14043527 TAAACTAAACCTTCTTGCCCTCCA 24 59.895 GGCATGCTTCTGTCTTTTGAGAAT 24 60.082 244

chr4 14046647 CGAAAGAGTGAGGTGGACAAC 21 58.864 GTCGTCATCATATTGGTTTGCTTG 24 58.97 201

chr4 14071743 TCTTTGTTTCCTGCTACTCTTCCA 24 59.897 CAGGAAAAGCAGTGGATTCTCAAG 24 60.083 210

chr4 14074608 CCATGAACATCAAAAGCGAGTTGT 24 60.557 AAAGGTTTTCTCTGGAGCTCATCT 24 59.957 89

chr4 14075379 ACCACACACAACCAATCTCATAGA 24 59.958 ACTGCTTTTCCTAAACCCTTCCTT 24 60.388 265

chr4 14076716 GATTTCCCATGAGCCCTTACACTA 24 60.141 ACACCTTAAGTTTTGTCAGGACCA 24 60.325 201

chr4 14077820 TCCTTCTTTTCTCCTTAACCTCCA 24 59.088 ATCGGTTTGTAGTAGTAGGAAGGC 24 59.901 291

chr4 14085660 ACAGAAAATCAGGGAGGAAATGGA 24 59.955 GGGGTTCACTTCAAGGGATAATCT 24 60.08 286

chr4 14128792 CTCATATTGAACCACGACGACTTG 24 59.968 GCTATGGTGTATAACTCAACTTGCT 25 59.182 257

chr4 14129081 TGTTTGAGAGAGAGAGAGATTGAAC 25 58.313 ACGAACTATCACAAATAGATGGCG 24 59.255 271

chr4 14130148 CCGTAGACTCCCACACAATATGAA 24 60.142 GTCAAAACTTGTTCGTCCTCTACA 24 59.193 189

chr4 14131652 CGAACGTGCATTACTTCTTCTCTC 24 59.969 TGTCGAATCGTACACAGAAGAGTT 24 60.023 190

chr4 14137170 TTGGCATGATCAGATGTTCGAATG 24 59.964 GATGTCAATCTATCCGTCTGCAGA 24 60.262 209

chr4 14144590 GAGTATTGAGCATTGAGAACACCG 24 59.967 GTGCTTCTTAGGGTTTGTTACGAC 24 60.084 156

chr4 14148630 GACCACGATTGAATTGTTTGTCCT 24 60.023 GATTTAAAGCATGTGTTTGAAGCGG 25 60.164 208

chr4 14150100 TAAAGCAGAATCGAAGGAAAAGGC 24 59.844 TTAAGACAGAAAACAGGAGGGGAG 24 59.958 266

chr4 14158716 CTGCCATGAGTAGTGTAGAATATAGAC 27 58.759 TCGTAGTGAGGTTTGGTCTTTCAT 24 59.96 223

chr4 14173795 CTACCAAAAGACCCATCAACAACC 24 60.022 TGCCTCTGGTTTCCTCATTATTGT 24 60.264 282

chr4 14177683 TGAGACACACACATAGGGAGAAAA 24 59.653 AGGCCTCCAAAGTCCTAAAGAAAA 24 60.141 245

chr4 14178334 CTACGTACCGGACATGCTACTTTA 24 59.964 GCTGCTATAATCCAACCAAAATGC 24 59.253 281

chr4 14181743 CCTTTGGCTTGTTGATTGGATTCT 24 60.021 TAATACAGCCAATCTTGAAGGAGC 24 58.874 275

chr4 14199640 GGATTCCTAGCCAACCTTCAAATG 24 59.901 GGCCCATGGATCTAAGATGATAGG 24 60.142 296

chr4 14200829 ACATCAAACTCGAGGTCATGTA 22 57.199 TCGACCTGAATCTCGGATTATATGA 25 59.056 185

chr4 14208472 CACTACAGCCATAACAGTTTGACG 24 60.143 GGAGTAGAAAATGTTTAACGCACTG 25 58.922 80

chr4 14214355 ATTTCTCCCCACTAATCACCATCT 24 59.271 AGTCGAGATGCATGTAAGTTGACT 24 60.082 244

chr4 14216328 TGAACCTTAGCTTCCTTCACCTAC 24 60.02 TAACATCCTCATTTGGTGCAAGAG 24 59.298 224

chr4 14242222 AGATGCAGCACTCAACTCTTTCTA 24 60.021 GTAGTCCTTTCGTTTCCTTTCACT 24 58.945 263

chr4 14255516 CAAGCTTCAATCAAATACACGCTG 24 59.444 TGGACTTTACACGCATCTCTTTCT 24 60.262 283

chr4 14267337 GGGGAAGCTCGTTTATGTGTTCA 23 61.116 AGCAATGAAACTCTCCCAACAATG 24 60.022 127

chr4 14287065 GGGAGGAGTAGCTTGTGGTTTTAT 24 60.324 GGAGGTTCAAATTCTCAATTCTCAC 25 58.384 263

chr4 14288293 TTGCAAGCACAGAGGGGAAG 20 60.538 CTCTCTCACCACAACTCTCGATAT 24 59.421 300

chr4 14302848 AGTTTTCTCCCTTGTTTTGTGACG 24 60.142 GTTTGTCTTTGTATCGCTAGCA 22 57.079 223

chr4 14304693 ATACAATCTCTTCTAAGCTCCCGG 24 59.718 CTGGAGGTTGAAGAAAGAGGGTAA 24 59.958 276

chr4 14308380 GCAACATGTTTGGAGAAGATGTCA 24 60.023 CAAGTGTCCAATACCCCTAGAACA 24 60.02 275

chr4 14311697 AGTAAATGCTGGAGGGCTGAATAT 24 59.895 AGCATCTTTCCTTTCTTTTCTGGC 24 60.022 188

chr4 14320999 CCAAGAAGCATGCCAAACTGAATA 24 60.082 TTTACGTGGTTAGGGCTTGAACTA 24 59.96 100

chr4 14322759 ATTTCGGTGCAATTGTCTGAAGAG 24 60.083 ATATTTGTACCAGTGGCAGTGACT 24 60.019 269

chr4 14329108 ATTTGTCCCATTCTTGAGGCCTC 23 60.879 GAGAGAAGAAATCACATGAAAGCCA 25 59.586 230

chr4 14351399 TTGGCTAGTCTGTCATTGATTCCA 24 60.019 TCATCCTTTGTACTTTCCCTACCG 24 60.081 155

chr4 14364875 AACTGGACCTATTAACACCCCATC 24 60.08 GCTTTGATAACCACCCTTTCGTTT 24 60.262 240

chr4 14368016 TACCGATCGTTTTGCTTCAACAAG 24 60.319 GATATTATCCTTTCCTCCGGCACT 24 60.02 271

chr4 14369031 TGTCTATGGTGAGTCGATTTGTGT 24 60.021 CAAACTTGAGGCCTTTTATGCTCA 24 60.022 177

chr4 14369778 GCTCCAATATGTCCAAGATGCAAT 24 59.72 GAGCTAACACCCACCACGTTATAT 24 60.442 196

chr4 14374730 AGAGATGTTGATTGATGGCAGAGA 24 59.836 CCACAGTAAATCCCATTGCCATAC 24 59.962 279

chr4 14375435 CCTTCCCTTCTTCACTTCTTTCTC 24 58.999 TCAATATACAAGACAGCCTGCT 22 57.239 300

chr4 14376847 CTTGAGCTCCCGTATTTCTCTTCT 24 60.142 CCTCTCCCCTATTGCTCTTTGTAT 24 59.651 222

chr4 14380427 CATCTTTGGTCACCTACATTGCTC 24 59.905 AAATGTTGTAGGGTCAAGCAGGTT 24 60.936 176

chr4 14380633 CATCTTTGGTCACCTACATTGCTC 24 59.905 GTGGTGAAAAGGGAAACATAGTCT 24 58.929 281

chr4 14383896 AGTTTGTTTCCACCTTAAGCACTG 24 59.902 AGTTTCGGTCTCTTGATGGATTCA 24 60.02 273

chr4 14385842 AACCACAATAAACCAGGAGTCTCA 24 59.896 ACAGAGAGGGTGAACTTCAAAACT 24 60.08 277

chr4 14397176 GTTTGGTACTAACAACTCCGGT 22 58.268 CCACGAAGAAACTCATTGTCAGTG 24 60.319 272

chr4 14399020 GAAATCACCATCCAATCATCGTGT 24 59.664 TGACATAGGATAAAGAAAACACCGC 25 59.647 128

chr4 14407158 GAAACTGCTTGAGACTCGAAAACA 24 59.966 ACCATGGTTCCATTTCACTACACT 24 60.203 159

chr4 14418303 GCTAACCCTAACCCACCATACATA 24 59.651 GGGACCAGAGAGTTTTCATAAGAA 24 58.194 225

chr4 14418754 AGGGTATGTTGGAAAGTGTATCGA 24 59.531 AGATTAGGGTTTGGAGTTGGAGTT 24 59.646 91

chr4 14420612 TCACAGACTCAAATCTTTCCACCT 24 59.897 CCCTACCTTTCTGTTCTCTCACTC 24 60.082 231

chr4 14430636 GTAAATATGGTAGCAAACGGAGAGT 25 58.953 GTCTTGGACTCTTGAGATTCACCT 24 60.021 270

chr4 14430844 GTGTAAGTCCTGTCTAGTGGCTAA 24 59.538 GGGACAAGGTGTAGTGTAAGATGT 24 60.02 274

chr4 14434875 CACCCTACACTACCATCCCTAAAA 24 59.528 AGTGTGGAATGAATTGGAGAAGGA 24 59.957 286

chr4 14447087 TGTTGTGGGCATCAAATTCCTT 22 59.029 GAGAAACTGGTTGTAAAGGGAAATG 25 58.553 127

chr4 14462074 TTTCGTGAAATGGTTGCTGTCTAC 24 60.024 ATTATTCCCAGAGACCCAAAGACC 24 60.08 232

chr4 14475842 ATCTTCCTACGCCTTTTGTCATCT 24 60.081 TGAGATGGGATTTGATGGATTTGG 24 59.103 99

chr4 14477165 GTTGTCCACCACCATTCATTTCTT 24 59.961 TGCGAGAGGTTAATACACATAGTTG 25 58.96 271

chr4 14478757 GGAAGACACCCTATCATGCACAT 23 60.434 CGTGGATTGAATTAAGAGCACCAA 24 59.844 241

chr4 14501264 ATCTAGGTGTCGATCCAAGCATAG 24 59.78 CTCTTTTCCGCTGTCCTTTATGTC 24 59.906 209

chr4 14502730 GCACTTCAATCCAAGCATATAATCC 25 58.615 TACTTGAAGAATGGCCGTTACTCT 24 59.778 246

chr4 14503285 TGGACACATCGCTTCCTATTTT 22 58.05 CGTAGTCGGGGAGTGGTAGA 20 60.108 277

chr4 14541580 TGGTGGTCGAAAATAAGTGTTTGG 24 59.963 CCATTAAACGCCAAAACCTTCTCT 24 60.022 159

chr4 14569120 GCTAATTTGTCTGGCTTGTTGTCA 24 60.262 ATGGTTGTGGAATGATAGGTTGGT 24 60.264 229

chr4 14590761 GAAGTTAAAAGGTGTGGTGTGAGG 24 59.964 ACATTCTCTTCCCAATCCCTTCTC 24 60.08 91

chr4 14592592 TCCAAGTCAGAATCAACAACGTTG 24 59.965 CACACAATGAACCCTCCAACATAT 24 59.294 258

chr4 14598662 GAAAAGGGGAAAACGAGGGAAAAG 24 60.501 TTCGTTCCCCATTACTCATTCATG 24 59.119 229

chr4 14626412 CCATCCCAATTCATCGTCTTCTTG 24 59.964 ACCGATTTCCTCTCTTCCACTTTT 24 60.202 266

chr4 14627038 TCAGCACCAGAATAATAAATCGTGG 25 59.473 ACAATCATAGAGACACGAGAAGGA 24 59.05 230

chr4 14632844 TATATGTGAGATCAGGCTGCCATT 24 59.712 CAGGTAAGATTGAAGCAACGATGT 24 59.607 239

chr4 14641782 TTATCCCTTGTGAGCCTTTGGAG 23 60.308 TTGAGAGGACAATAGCTTCGTAAG 24 58.348 300

chr4 14675993 ACGCTAAGACATCCACTAAGTTCA 24 59.78 CGTACAACTAAGCTTCACTCCAAC 24 59.85 269

chr4 14677747 TGCATGGGATGATCAAGATAGACA 24 59.651 TGGGTCCTTAGAATCTCACTTTTCA 25 59.69 253

chr4 14678554 GAGATTGTGACATTTCTTGAGAGTC 25 57.941 TCAACAACGATATAGAACAGGGTCA 25 59.814 194

chr4 14696198 GAGGACTTGCATTTCTTCACAACA 24 59.963 TCCATGAGTCCCCATTATTTTCGT 24 60.08 269

chr4 14701920 CTTGGATTTCTTGATAGGTCTAGGA 25 57.633 GAGGGTTCGAATTTTGTGCTTC 22 58.434 220

chr4 14733813 CAACCAAACAAATGACCACTACGA 24 59.964 TTTTGAGAGTCTATGAGCTTGGGG 24 60.324 262

chr4 14759054 ACAAGCCACCCTATTACATTTCCT 24 60.017 ACTGGAGGCTAGTTTTGAGTAGTG 24 60.021 211

chr4 14759158 ACAAGCCACCCTATTACATTTCCT 24 60.017 ACTGGAGGCTAGTTTTGAGTAGTG 24 60.021 211

chr4 14768461 GGCAAATGGTGAAGTTCTCTCTTT 24 59.721 TAACAGCAACAGACCTAGAGCAAT 24 60.02 225

chr4 14772825 GACACTCCTTCTATCTCCAAATTTC 25 57.453 CTCAAATTTTGGTAGGCTTTCTCAC 25 58.848 299

chr4 14780783 TTTTGCCTGTCAGAACCAACA 21 58.549 GATGCACCTTACCTTTCACTTAGC 24 59.905 186

chr4 14781220 CTGTATTCGTACATTCACCCTCCA 24 60.142 GTACTTGCCCATATGCCATTTTGA 24 59.901 170

chr4 14782092 CGTCATGATCTCTGTTGTTTACCC 24 59.67 TCAAACAAAGGAAGTCATACTCGC 24 59.549 299

chr4 14783040 CTCAACTAGTGGTGATCATCGGTG 24 60.971 ACTAACCCGGTGATCTTGAATTCA 24 60.02 296

chr4 14784576 TGACTGTCATGATCCTTTAGTGGT 24 59.468 TGAATGAGACAGTGAGATGACCAA 24 59.716 281

chr4 14786331 CTACTGCAAACTAACAAACTCTTCC 25 58.34 CCAAACCAACATCAATTCACCATC 24 59.069 264

chr4 14794437 GCTATTCGTTGATTCTTCTGGCAA 24 59.905 GAAATGATCTTAACCTAACAGCCTG 25 57.979 200

chr4 14795178 GGAGGGTTCATAATCAGTTTGTGC 24 60.142 TGAACCTCTCTCTCTCTCTCTCTC 24 59.838 146

chr4 14795761 ATTGGCTTTGTTGTAGTCTTTGGG 24 59.961 CTCGTTTTCTTTGCTTGTCTCGAT 24 60.084 219

chr4 14808583 TTATCAAAATGGCAGTCCTAAGGC 24 59.355 GGCCACTCAATTACCAACACTTAC 24 60.083 172

chr4 14821733 ACAATCGTCATAGCATCATAGGTG 24 58.767 AAAGGGGTTATGAGAGACAAAGCT 24 59.957 291

chr4 14823531 AGCACCCTATTCAAGATTCCAACT 24 60.018 ACTGGCTATGATACAAGAGGACTA 24 58.294 186

chr4 14835641 TGTTGGACTTTGTTCTCCTTCTGT 24 60.324 AGTTCTACTACCCTAGACTCAACCT 25 59.746 179

chr4 14853044 AACCCAAAGCAGAAACCATAACTC 24 59.719 CTTTGGGTTTCCTATTGGCTTCTC 24 59.841 215

chr4 14858391 GGAACAAACAAACCCTCACAAA 22 57.805 GATGGGAGAGATGGGAGAAACTTT 24 60.08 284

chr4 14875796 TTCAAGAAACGCACACCAGTAGAA 24 60.92 TTAGAGCCTCAACCAAGACCTTTT 24 60.141 273

chr4 14914553 AAGAATACGTACCTTGCATGTCCA 24 60.323 ACTAAGGTGTTATTTGGAGCGACA 24 60.263 259

chr4 14939301 ACATCGGCAAAAGGTGATCAATAC 24 59.904 AGAGGGAAGAAGAGAGAGAGAGAG 24 59.835 271

chr4 14941088 GCTAGATGGAAAATGGGTATTGCC 24 60.022 TGCATTTAAGAGGAGACATGTTGG 24 59.298 292

chr4 14941371 GCATAGAAACGAGAAGAATGTGGG 24 59.965 TCTCAACCATCCCACTATTTTCGT 24 60.02 109

chr4 14950688 TTTTATTGCCAATACGCCTCGC 22 60.223 CAGCCAATGTTCAAAATCACCTTG 24 59.55 298

chr4 14951077 TTTAATTCTGTTGGGGCACTAGGT 24 60.203 ATCTCCTCAAGTTCTTGATGGCAT 24 60.08 275

chr4 14955244 TTCACCAAATAGATGATCCTCCCC 24 59.895 TCATGTGGATGGAGGAGGTTTTAG 24 60.08 185

chr4 14962611 AACAATCTCTTCTCTCCACCCTAA 24 58.906 TAATCTTACCGCTTCTTTCTGTGC 24 59.37 287

chr4 14964940 ACCACACTTCAAAACTATAGCCCA 24 60.203 GGCTCAGACAATCGGTTTCATTTT 24 60.321 270

chr4 14982095 CATTGTTCTTCCCTTGACTGCTTT 24 59.962 GAGAGCAAGATCGAGCAAGAGATA 24 60.023 298

chr4 14987532 CCATCTAAAGTCGACACCCCATAA 24 60.142 CTCTCCTCTCCTACTCATCCTCTT 24 59.895 97

chr4 15007017 GAGGTTAAGGTCAAGGGATAAGGG 24 60.141 CGGAGAGACAAGCTTAGAATTGGA 24 60.382 258

chr4 15024109 TGTTGCGATCCCAAAATAACTCAG 24 59.844 ACTACCTCCCTCCACATATCCTTT 24 60.328 199

chr4 15032261 GTTGTGCAAGAAAATGTTACAGGC 24 59.791 TGACATATTGACATCCCGCCTTAA 24 60.142 219

chr4 15033936 AGTGTGAGAATACTTAGAGGTGACA 25 58.989 TTGTTCGACTCAAAACTTCCATCG 24 60.025 204

chr4 15034523 AAAGTAGTGTGATAGGTGATGCGT 24 60.082 AAAGTCATCATGTTCGATCTTGGC 24 59.905 281

chr4 15034689 AAAGTAGTGTGATAGGTGATGCGT 24 60.082 AAAGTCATCATGTTCGATCTTGGC 24 59.905 281

chr4 15053739 GTCATGTGGAGAAAGGAAGTGATG 24 59.606 CATCTCATTCTCCTTTTGTCCACG 24 59.906 161

chr4 15064849 ATAATCTTAGCTTGGAGTGGGTGC 24 60.687 AGACTCCATCATTCAACGTCTTACA 25 60.047 298

chr4 15074058 GGATGCATTGGAAATGGTCAAAGA 24 60.082 TGTTCATCGTCTTCTCCAATGTCT 24 60.021 215

chr4 15094302 TCAGTCCTCACAAACCACTAACAT 24 59.897 TGCTTCACATGTCTCTGCTACTG 23 60.616 274

chr4 15102476 TTTTCACTTGCTCTTTGTCACTGC 24 60.675 CCAACTTGTGCTGGTAGTATGAAT 24 59.056 214

chr4 15123049 CCTTTTGCTTCAGTTTTAGGAGGG 24 60.022 ATGACCCTTGGAGATTTTGAGGAA 24 59.955 197

chr4 15124902 ATTCCTGTGATAATGAACCGAGCT 24 60.142 CCAAAGTGTCCATAGTTTAGTCTGA 25 58.536 300

chr4 15128873 CAAATCGTGATTCTTGGGGTTCTG 24 60.38 GGTGGTTGTAGGTTGTCTATCACT 24 60.02 260

chr4 15144046 TGATATGGAATTGGAAGCAGCTCT 24 60.141 AGGGTAGAGTTGGGATTTGGAAAA 24 59.893 267

chr4 15144440 TCCAATTCCACCCATCTTAACTCT 24 59.46 GTCTTTGCCACCTCATCTTCTTTT 24 59.721 294

chr4 15145168 CTCCTTTGACATTAGAGTTGTGTAC 25 57.647 ATGGGGTTTGGTCTCTCTTAAGTC 24 60.019 227

chr4 15146093 TTGGGTATGGGAGATTAAAAGGGG 24 60.079 GGATTTGGCATTTTGATTATGGGG 24 58.935 273

chr4 15147923 GGCTCACGGGAAATTTCTCATATT 24 59.419 AGGGATGAGGTTAAATCAGAGACA 24 58.967 250

chr4 15150470 ACCTCTCTATGGAAGGTGAAGTTA 24 58.409 GGTGCAGTTTTAGCTTGAAATCGA 24 60.32 298

chr4 15150832 CAAGCGTATGGGTGTTTACTCTTC 24 59.907 GGTTCGGTTCGGTTCAGTTTTAAT 24 60.024 218

chr4 15158409 AGACTATCTGCTGGTGTATCAAAC 24 58.335 AAAGGTAAAATGTCACAACTGCCC 24 60.202 225

chr4 15161934 GCTCTTTCCTAGGATGGTTGAGAT 24 59.898 TCACACCCTAAGTCTCTAGTACCA 24 59.71 260

chr4 15165574 CCATAAATGACAGAAATTGCCGGA 24 59.902 TTTTCCTCCCTCCCATAAAAGAG 23 57.807 248

chr4 15171848 GAAACTCTCACCTTTTCCATTACAC 25 58.331 CAACTAGCCAACCTAACCCAAATT 24 59.473 262

chr4 15172947 TTTGCAAGGACCTCATAAACATGC 24 60.322 TCATTCACAACCTGTCGTCTTACT 24 59.961 274

chr4 15185899 TAACATTGAGCAACCCTAACCGAT 24 60.324 GGATGACGGTCAAGCTAGAAACTA 24 60.142 179

chr4 15197490 AGTTGTTTTCCTGCAATATAGCCG 24 59.904 CCTTTAGACTTGTGCCTATTTGATC 25 57.979 296

chr4 15202004 TTAGCTCAATCTGAATTCGCGTTC 24 59.967 CACCAGATCTCCTTCTTTGAATTTG 25 58.381 254

chr4 15203390 TAGACAGAGTTCCATCCTCCTTCT 24 60.017 TTTGCGTCGAAGTTAGCTACACTA 24 60.32 174

chr4 15205528 ACTAAGAAAGCAACGTACCTCAGT 24 59.961 AAGTTTAGCAAATGTGGGTTCTGG 24 59.961 145

chr4 15207308 CATTTCCCTCGATGCAATACAGAA 24 59.424 TACTTCCAGTTATTGCACGTTCCT 24 60.263 252

chr4 15210032 ACCCAATCCAAACTTATCTTCCCA 24 59.955 ACAGACTCGTTTCCATAGCTTCTC 24 60.381 275

chr4 15217774 CAAATTATGTCTCGTCAACCACTGT 25 59.821 CCACTCTTTCTCTGTTGCATCTTT 24 59.482 300

chr4 15225694 AGTAACCTTCGTACAAAGACTCCC 24 60.022 ATGGTAGCTAATCCCCGTTTTCTT 24 60.08 300

chr4 15230378 CAAACACTGGACCTCATTCAAGAC 24 60.024 GTGTATCTCAAGTAGCGGTTTTGG 24 59.907 178

chr4 15241468 AGAATTTGGGGTTGGAATGAGAGA 24 59.955 GTCACACCACCCATTTAAACATAAC 25 58.846 298

chr4 15246509 TATATTTCGTACGTACTGCAGCCA 24 59.963 GAGTTTGTTGAGGCGAAGATGAAA 24 60.024 191

chr4 15248430 GGCAATAGATGGTCAGAGGAGAAT 24 59.959 AATGAATATTGGTGAGCGCTGATG 24 60.023 158

chr4 15251569 GAACTTGCTAGGATCTTGTTTTGGT 25 59.758 GAGTAGGAAGGGTTGACTAGAAGC 24 60.142 213

chr4 15257639 TGAAGTCGACAAGTGAGAAGGAAT 24 59.961 CTTTTAAAACAACACCCGTGACCT 24 60.142 147

chr4 15263614 CCACTACCTCCAAACACTAACACA 24 60.444 GAGTACTACCTCGACTTAGAATTCA 25 57.449 295

chr4 15265761 AAAAGAGTTCCCATTCCAGATCCA 24 59.955 TGGGCTAAAGGTCATTACACTGAA 24 59.958 247

chr4 15274308 GACACGATTTTGGACGGAAGAAAT 24 60.083 GGAAGACGACAGACATGGAAATAAC 25 59.936 184

chr4 15298928 GCGCACATTAGAAAATACACCAGA 24 59.906 TGTCAAATCAATCACCAAACCGAG 24 60.023 249

chr4 15300665 TCTAATTGTCTAAGTCGCTGCTTG 24 59.135 TGGGAAAGCAATACACCATGTCTA 24 60.019 278

chr4 15306734 ATGAACAAATGAACACTTGGCCAC 24 60.74 CATATGGTGTCAGCAGAAAGGTTC 24 59.905 298

chr4 15307132 TCATGAACCCACACTTGTACTCAT 24 59.958 AAACCAATGACCTCCCAGTTATCT 24 59.708 198

chr4 15309044 CCTTCGTTCCTTTTCTTTTCCGTT 24 60.202 CCATTTCCACCAACTCATCTATTCA 25 59.112 288

chr4 15309236 AACCTAAGATTGTTCAGACTCCCC 24 60.019 GCCCTTTTAATCCGGTTGTGTAAA 24 60.022 266

chr4 15320774 GTGGGATCTAGCTTTGGAATCTGA 24 60.142 AGCACTCTCTGTTACAATAACCCA 24 59.714 250

chr4 15327419 TGGGAAGTGGAATTTTAAGCAACC 24 59.96 ATCTTCCCCTTGCTGATGATTTCT 24 60.079 149

chr4 15331957 TTTCTGTGGCTTCGTTTCTTTCTC 24 59.965 TACAAAACTTCAATCACCTGTGCC 24 59.963 143

chr4 15333256 CTCTCTCCGAATTTCCATGCAAAA 24 59.843 GGCGTAAAGTGTGTAAATGTCCTC 24 60.143 298

chr4 15374112 TCATTTGATTCTAAGCTGCCTCCT 24 60.08 TTACATCGTCTTGAGTCTTGAGCA 24 60.022 279

chr4 15421672 GTTTGAGTCAGTTAGGCACACATC 24 60.084 GAACTTCAATCTTCGGCAGTGATC 24 60.202 274

chr4 15433593 ACGAGTGACATGACAGACTAACAA 24 59.962 TCATGCCCATTTCTACAATGCTTC 24 59.902 277

chr4 15437471 CAAAACCGCTCCCTCTTTCAAAA 23 60.181 TATTTGTTCTTCCACCCCAAGCAT 24 60.756 257

chr4 15445438 ATTTTGTTAAGGTCAAGGTCGTGG 24 59.723 GCGAGACCTCATAAGATGCCTAAT 24 60.322 116

chr4 15461217 TCAAAGAGTACACGTGCTTGAAAC 24 59.966 GGGCAAGTTTGGTCATGTAATTCA 24 60.022 281

chr4 15462671 TTGGATCCATGGCGCCTATAAATA 24 60.019 CAGTCTCTCTTCCGAACTCTTTCT 24 59.782 140

chr4 15468192 CAATCTTGTGGAGGACTTGTGTTG 24 60.261 CCCTGGAAGCAATTGTCAGTAATC 24 59.903 149

chr4 15468820 TAGTTGAGTCAAATCTGGTGAGAC 24 58.216 CAGTTCAACACCATGCAGAAGATT 24 60.023 116

chr4 15469086 AATCTTCTGCATGGTGTTGAACTG 24 60.023 ACACCGTTTAGCTTCCATTCAA 22 58.518 285

chr4 15471262 GCAAATAAGACTGAGCAATTGGAC 24 58.902 CTTGCAGTTTTCGAGGTGATTCAT 24 60.083 162

chr4 15476749 ACTTTTCAACATCATGGCTGGAAG 24 60.022 CGTATTGCTCGAATGGTAAACAAC 24 58.981 244

chr4 15478617 TGCCTTTTCCTGCCTAGAAA 20 57.03 TCTACACCCTCCCGAGTTTT 20 57.98 300

chr4 15485375 CTATGATAAGATCTAAGGCCGAAGT 25 57.836 TCGAGAAGAGCTAGAAAATAGAGAC 25 57.746 288

chr4 15512236 GAGTAGAAAGGGGATTGGGAATGT 24 60.08 CCCCACCACCTTCTATTTCCTTTA 24 60.018 218

chr4 15512465 AATGCAGAAGGAATTGAGGTTTGG 24 60.021 GGAAGTTTGATAGCAGAGGTTGAG 24 59.366 259

chr4 15512956 TCTCCAACCTCACTGTCAAAGATT 24 59.897 TCAGGGGATGCATGAGAATGTC 22 60.159 299

chr4 15514369 TAGAATCAATTGTGCACCCTGAGA 24 60.019 CTGCAACGGAGGATAAGTATGAGA 24 59.962 250

chr4 15522113 TAGAAGCACCCACCTGTAAAGAAG 24 60.263 CCATATCAGTTAACTTCGCAGCAG 24 60.025 287

chr4 15534517 AGTCCACCTCTAACTACCTAGATT 24 57.656 TGTGTTGCCCAATTACATGCTAAG 24 60.082 209

chr4 15548618 CTTGACCGTAACCTAACATGCAAT 24 59.605 AGTTTCCTACAACCTCGACTACTT 24 59.168 278

chr4 15563727 CATTACCACAACCCATATCAAGGC 24 59.962 CGATGAAGGATCTATGGCTTGCTA 24 60.322 271

chr4 15564721 CTTGTGATGACGTGGCAATCCTTC 24 62.434 TGGACTACTGCACGATCATTTACA 24 60.082 295

chr4 15565588 ATTTTCCTACCCTGACCCAACTTT 24 60.141 TCACATCTCCAAACCACTTACACT 24 59.897 276

chr4 15567607 GTGAACAGAAGAACAGAGCACAAG 24 60.261 TGGATTATAGAAGCTCACACTCCC 24 59.655 276

chr4 15572665 TTGAAAGTTTGAAAGGGACAAGCC 24 60.383 TCAAAGAAATGGAGGGGAGACAAA 24 60.141 239

chr4 15573556 TTGGCAAAATAGGGAGATGAGGAA 24 60.018 TCAAAATTCAACTTCCACACGAGG 24 59.964 202

chr4 15575131 CGCTTCCCTTCTTTCTCTAGGTTA 24 59.84 CGACTAGGAACGATCACCATATGT 24 60.023 231

chr4 15580056 ATCTGACACGCTACCTTGAATTCT 24 60.081 TCAAAATCATGACTTGTCCCAACG 24 60.023 241

chr4 15591553 TCTCCACTCCCCTTTCTCTCTATT 24 60.016 CCACGTTCTCTTTCTTTCTCCCTA 24 60.021 292

chr4 15615904 TCGCTTCCTTTTCTCGACTTCTTA 24 60.023 GTGGTGAGTTTTGAGGCGATTTTA 24 60.024 280

chr4 15624110 AATGGCGTGCTTACCTCAACTATA 24 60.142 CCGATCCAATTGCAGCTGATATTT 24 60.023 263

chr4 15631320 CCCTCAATTCATGCATATAACCTCG 25 59.877 GCATGTCGATCAGTAAAATTATCCG 25 58.7 295

chr4 15633641 GTAAGAAGGTGAGTGGATGGAAGT 24 60.02 CTTTTGACCATGAAATTAGGATCCC 25 58.19 296

chr4 15634962 TGATAACTCAACCCAATCCAACCT 24 59.956 CCTCATACGGTCATATCATGCTCT 24 59.841 187

chr4 15664997 CGGTTACAAAATCCTAATCACGGG 24 59.965 AAATTGCTTCAACTGGTGGCTAAG 24 60.262 208

chr4 15668654 ACCTCACTTTCACTTCGTCTTCAT 24 60.202 AAATTGGATGAGACATTGGTTGGG 24 59.777 296

chr4 15674726 TCACAGGAAGGGAAGAGATTAAGT 24 58.906 CTCAACCTAGACATTCAAGAGTGA 24 57.969 294

chr4 15675698 GTTGCCATTCTTTTGTCGCCTTTT 24 61.504 TCCACTGACTTTATTTCCTCGT 22 57.115 283

chr4 15681343 AGGATAGAAATGGCCTAGAAGCAA 24 59.586 GGATCACATAATTCAACCACACCC 24 59.902 123

chr4 15682077 CAAGGTGTGTCTGCGATGATAAC 23 59.937 GCCAAAATCTGCCGACTTCTAGTA 24 60.918 293

chr4 15683086 GACAAACTAATGGGGCAAATCACA 24 60.022 CTCGTGCGAACAACCATCTTAATA 24 59.435 202

chr4 15683652 AGATGGTAGAAGTTGGAGTCTTGG 24 59.776 GTCACATGTTTAACCGTTGGATCA 24 59.785 273

chr4 15685005 GTCACCCGTTTAAATTTTGAGCAC 24 59.557 CAGTGTGGGTCTTGTTGTTTATCA 24 59.421 150

chr4 15685191 TGATAAACAACAAGACCCACACTG 24 59.421 GTCGGTAATGATGAACTTAATCCCAC 26 60.018 223

chr4 15704742 TGAGAGATGGAATTAGAGAAGCAAG 25 58.419 ATCAAATAGTGTGTTGCTTTCCCC 24 59.779 207

chr4 15707880 TTTGACCTTTCTCCTCGGAATCTT 24 59.958 TAAGATGTCCAAGGGTTCCAATAC 24 58.248 260

chr4 15708170 TTCCGATGTACTAATGGACCGATC 24 60.023 CCCCTCCCTAACCTACAACAATTT 24 60.264 248

chr4 15713010 CTCCTACATACTTTCACACACACA 24 58.462 GTCCAATTCAAGAAATGTATGCACC 25 59.193 300

chr4 15713743 CCTTGGGGTATGAGGTAGTACAGT 24 60.879 GGGGACGGAAATATGTGAATGAAC 24 59.964 279

chr4 15840448 AATTTGAGGGTTGCTGCTATCTTG 24 59.841 TCATCTTCATCATCACTTCCAGCA 24 60.081 174

chr4 15907015 TGTCTCTCTCTCTCTTTCTCTCTCA 25 59.752 TGGTTTATAAGTGGGCGGAAAGTA 24 60.02 188

chr4 15913005 AATATGACGAGTGCCAAAACTACG 24 59.671 TGGGTTCATGAAATATGCTTCGATG 25 59.991 258

chr4 16051128 GGATCTCCTGCTAAAATGGCTAAT 24 58.68 CGATTGAGTATAGAGAGAGAGTATTCC 27 58.12 298

chr4 17051266 TAATCTTGTTCGGACCTCAAAGCT 24 60.263 CCATGGACCCAACTTGTAAACATT 24 59.717 183

chr4 17052452 ATGTCGACGTGGTATAGAACTTCT 24 59.361 TAACGTTGAACTTGTTTGGGTTGG 24 60.381 278

chr4 17052553 ATGTCGACGTGGTATAGAACTTCT 24 59.361 TAACGTTGAACTTGTTTGGGTTGG 24 60.381 278

chr4 17055620 GATCACCTGCTCTGGATTCGAAAG 24 61.503 CTAGACTTACTGCTACTAGGACAAT 25 57.189 300

chr4 17056167 TACCCTTCCTGATGACCCATAAAC 24 59.835 CCGACTAAGGATCCGTTTAAGAAT 24 58.401 245

chr4 17058777 ATTGAGGGGTGGAAATATTGTCGA 24 60.08 CCATGTCCAAGATGCATCACTTTT 24 60.082 168

chr4 17061014 TCTCGAGTCTAAATCCAACAGCAA 24 60.021 GGTGAATTAGAAGCAGGAGGTGTA 24 60.081 219

chr4 17063772 GGAAAGTCTGCCAATAGAATGCA 23 59.304 AAAGACCAATGTTGATGAGCCAAG 24 60.022 182

chr4 17098367 TAAGCTTACTCGAACACTATGGGG 24 59.901 ACTGAAAATAGTGGCCGAAAGTTG 24 60.023 240

chr4 17101283 TGACGTGGGATGATGTAAGAGTTT 24 60.021 GGTGGGCAGGAATAAAATGTGAAT 24 59.838 207

chr4 17101541 ATTCACATTTTATTCCTGCCCACC 24 59.838 CGAAGCCGTAATGCAAATTTTCTC 24 59.737 283

chr4 17106791 CCATTCGCGTGCATATGAGTTAAA 24 60.261 ACAGATCAAAGGAGGTTTCATGGA 24 59.957 284

chr4 17108029 ACAACTACCTTTACTGTCTGTCCA 24 59.348 GAAGATGGAGAGGTTAATAATGGGA 25 57.921 185

chr4 17111958 TCGTTCGAGTATGGACATGATGAA 24 59.903 ACTCCCTCACTAGATACCACATGA 24 60.079 293

chr4 17114247 TTCGATGTTGAGGAAGTATGTGGT 24 60.021 CAACTCTGGATAACGAGCTCTCAT 24 60.202 225

chr4 17116116 ATCTCCTCTAATCTCCCTTACGAG 24 58.434 TGTCTTTTCTTGGTGGGATTTGTG 24 59.901 256

chr4 17116734 ATGAATTGACTCACCACACCTGTA 24 59.958 CTTCCTTCCTAAACTTTTGTGCCA 24 59.659 275

chr4 17122497 GGAGGTTTTGTGGTTTTGTTTCAC 24 59.609 GGGTAGGTTTAGGCAAGGAGTTTA 24 60.019 176

chr4 17127492 CCACGAAACAATCCTAAGCACAAT 24 60.083 ATGATAGATTCTGGAAGGTGTTGG 24 58.311 220

chr4 17127658 AGAGTATGGCAGAAACTACAACGA 24 59.78 TCGTGAATCATCTGTTGCAAGTTG 24 60.32 214

chr4 17139606 GAACCCTTGATAGTGGAGCCTTAA 24 60.081 AAAATTTTGGGGTGAATGGTGAGG 24 60.202 120

chr4 17145052 GGATTGTTTCTAGTCCACTCATTTC 25 57.922 TGGTTAGAGAAGAGAGGGTTTTGG 24 59.958 236

chr4 17147164 CTACCTCTCGTATTTCCTTTGCCT 24 60.142 AGATAAATCAGGGGTATTCGGAGC 24 60.02 177

chr4 17148778 CACCCTCAAGAATCAAATGACACC 24 60.083 GTTTGTCTTCGCTCCATTTTGTCT 24 60.261 235

chr4 17150557 TCGTTGGTAGAAGAAAAGGGAA 22 57.307 TGGAGAAGAAGAGAAAATGACGATG 25 59.128 246

chr4 17151478 GGTATTTTCCATTGTGAGCCTACA 24 59.053 TGGTCGATTAATTGCGGGAGTATT 24 60.444 247

chr4 17152899 CCTTTTCCAACTCTTGATCATCGG 24 59.905 TAGCCGCTCAAACTCCTCTAAAAT 24 60.081 166

chr4 17153580 TAGAGGTCGTTGGGTGTTGTTTT 23 60.371 TGAGTCGAATTCCCACCTTTCTTA 24 59.715 300

chr4 17182161 GTGTAATGAAGTAGCTCCTCGTCT 24 59.903 GGTTCTAGCATACCTGAATTTGAG 24 57.389 283

chr4 17193173 TACAGGTGATTATTGCAACCCAGA 24 60.019 CGTTACCTCCTCACGTCAGATAAT 24 59.964 264

chr4 17219724 ATAGAAAAGATGGTGCTGGTGAGA 24 59.774 CCCATGGCCCTTATATCAAACTCT 24 60.203 261

chr4 17221614 AAGTAGGAAGTTGTTTGGGGAAGA 24 59.833 AAGAAAAGTGGGAGCACAAACT 22 58.635 295

chr4 17223054 CTTGATTTGTGGAGCTTTCAGTGT 24 59.963 GCTCCATTGAAAGTGAATTGTGTG 24 59.317 232

chr4 17225226 TTCTACTTTCACTTCCCTATCCCC 24 59.28 TTTTGGAGGTAGGATTGAAGGGAG 24 60.018 271

chr4 17227010 GATAGAACTAAGACATGCACAGCG 24 59.792 CAACCTGGCTCTTTGTCTAGATGA 24 60.323 200

chr4 17230079 TATCTTTCACACTTCGTCCGAGAG 24 60.143 AGCAAGGTCAATAGGTGTCAGAAG 24 60.564 80

chr4 17237412 GATAATCCGCACAAAAGCTCCAG 23 60.243 TCATGATCTTCGTCTAACCAAGGT 24 59.534 288

chr4 17243890 TGTATTTTCCATTGTGAGCCTGTG 24 59.782 AGAAATCGCACGCATATGAAGAAG 24 60.025 294

chr4 17276584 TGAAGAGTCATGAGCAGAACTACC 24 60.082 GTGATGCCAAATGTGATACCTTCC 24 60.202 279

chr4 17283654 TTCTTCCTTTTGACGAACGATTGC 24 60.554 ATTGCATCCCCTACTTCATTTTGC 24 60.142 271

chr4 17286280 AATTGTGTCAAGGGTAGTGCGATA 24 60.323 TAGTAGTTCATCAATTGGTGGCGA 24 60.081 242

chr4 17299414 CACATAATCAGTTTAACGCCCCAA 24 59.843 GTCATACGAGATCACCACCCTTTT 24 60.623 296

chr4 17301786 CGTATAACTCCCAACGCTTTTGTG 24 60.668 GGGAATGGGTAGATTAGGTGTGTT 24 60.08 205

chr4 17322794 GAGAGTTCCAATTTTCGTGTCCAA 24 59.725 GGGATAGGGATAGGGATAGGGATAG 25 59.983 177

chr4 17329794 CTCCCTCTTCCTCTTCTCATTTCA 24 59.531 AGAGTAGGTCACAAGCATAAGGAA 24 59.222 297

chr4 17330069 ACCTACTCTTCACCTACAAATGCA 24 59.714 CGTCTTCAAACCATGCATATCCAA 24 59.904 295

chr4 17330245 TATCAAAGAAACACCGCTCCTAGT 24 59.778 TTCCACCATTCACCTCACCAATAT 24 60.018 84

chr4 17330930 GCAAGTGATTTCAGGTCAACAACT 24 60.202 TGTTTCCCACCTATGTAGATTACCT 25 59.032 286

chr4 17333504 CTAAATGTCCAAGCAGCTCATGAG 24 59.965 AATATTTCAAGCCCACAAGTGAGG 24 59.536 264

chr4 17340296 ACGAGTCCAAGTACCACTAATTCA 24 59.475 CTGCTATAGTGGTAATTCTATCCTCGA 27 59.663 284

chr4 17348076 TATTTGGTGGGGTTTGTGAATTCG 24 60.022 GTCCACTACAACCAAGTTATCCCT 24 60.02 176

chr4 17348346 GATGGATTGTTCTAAGAAGCTGCC 24 59.964 AACATAGTACAAACGGGCCATAAC 24 59.363 289

chr4 17356713 GGCGCCATCAATGAAAATCACTTA 24 60.44 GCATTTTCAAAGTAAGAGGGCTGT 24 60.022 292

chr4 17359014 AAAGAACAGGCAGACATTCATTCC 24 59.781 CACCGTCCATAGTTTGAACGAAAT 24 59.847 293

chr4 17372292 TCTGACCCTACAACATTTGAGTGT 24 59.897 TGGGGTGTATTCCAAGTTCTTCAA 24 60.141 274

chr4 17380478 AATCAATTTCACACTAGGCCACAC 24 59.782 CCGAACTTACCTCTAAAATGGAAGA 25 58.596 299

chr4 17380836 TGTTCATTAGAGAGTAAGTGGGACT 25 58.983 GCTAGTAGGGGAATCTAATGAACCT 25 59.455 290

chr4 17382160 CCCCAATCTTACAACACTCATTCG 24 59.905 TAGCACATTTTAGAGTTGGGGTGA 24 59.958 160

chr4 17391098 TTCTGATCAACTGTCTATGTGCCA 24 60.02 AAACTCTCACTTCCCAAAACATGC 24 60.202 295

chr4 17404443 ATCTTCTCCTCTCAAATGCCTGTT 24 60.019 GACAACATGCAGATTGAGCTAAGG 24 60.202 201

chr4 17409186 AGGGTTTGCTCTAAAGGTCTTGAA 24 60.141 AAATCTCCTCTATCATACCTCATCG 25 57.597 299

chr4 17431271 TCGGACAATGGCTAACTTTACTGA 24 60.021 GCCCTTCCCATTTTGAAAATAGCT 24 60.081 262

chr4 17439790 AAGAACTATGATTGGTCGAGGGAG 24 59.9 CGTTTGGTTGATGTCAGTTTGAGA 24 59.965 271

chr4 17462634 TGTGAGTGGGATTTTAGCGAAGTA 24 60.021 CTTCATACATGGGCTTCAATTGGA 24 59.354 266

chr4 17477404 GCTTACTCTTTCTCCATTATCCTCA 25 58.179 CCATCACTTCTGTTTCTCAAATCAAGG 27 60.787 247

chr4 17480504 AACGTCCTTAACTCCTACCATCAC 24 60.082 GTATACTGCACTTTCACACGCTTA 24 59.376 173

chr4 17568912 TGAGATGGTATAGAGGACAATTGCA 25 59.634 ATTGAAAGAAACCAAGAGTGACCG 24 59.724 220

chr4 17571575 GCTCCAACTTCCATTTCATTCACT 24 59.781 GCTTCCCCTTTCAACCAATCTTTC 24 60.562 262

chr4 17573164 ACTGGCTGTAATGGATTTAGGGTT 24 60.017 TGGATAGTGTTTAGATCATGGGGT 24 59.025 276

chr4 17602671 GATTTGGATCGGAGTTTGTGATGG 24 60.202 TTCTCTGTAGGACGAACTTTGTGC 24 61.032 253

chr4 17641050 TACAGACAAAGGCATTTCTACCGA 24 60.021 GAGTCCATCTTACACCTCCACAAA 24 60.263 291

chr4 17649017 TGTGTGCCTATAACCCCTTTACTC 24 60.081 TCCCTCAAGTTCGACTCTTTATGG 24 60.082 121

chr4 17650728 GCATTGTTGAAGTGGGAATATGGA 24 59.598 AAAAGCCCTATGAGCACGTGTTT 23 61.31 225

chr4 17656175 ACCCAAACTCCCATTCCTATCTTC 24 60.08 AGTTTCAGCCAAAGAGAACGAATG 24 60.024 158

chr4 17665279 ACTTCATCTAAACCCAACAACACC 24 59.417 TCGATCTATTGTTGTTCAGTTGGC 24 59.608 293

chr4 17666176 CAACCTTCTTCTCTACTTGGCCTA 24 59.777 AGTATGTTCTCGGATGCTGTTGAT 24 60.142 284

chr4 17706535 ATTTTGGAAGAGTGAGAAATGGGC 24 59.779 GCTAGATAGATAAGAATGGGAGAAACG 27 59.242 239

chr4 17724089 TTTGTTGAATCAGACTGAGGGCAT 24 60.751 TCATGATTGTCTACGTTGGAACCA 24 60.263 202

chr4 17725262 TGTTGTTATCCATGGTTGAGCTCT 24 60.263 GCTCTACGGTGGCTAATTTAGTC 23 58.95 265

chr4 17726338 TCATACCCTGTTATAGAGATTGCCA 25 59.156 CTATCGCAGTAGACCACCCAAATA 24 59.961 250

chr4 17727144 TTTGATGATTTGGATTGTTGCCCC 24 60.565 ATGATGCATTCCCAACAATCCCC 23 61.517 186

chr4 17727382 GGGGATTGTTGGGAATGCATCAT 23 61.517 AAATTGCTTATGTCGGTTGATCCC 24 59.902 299

chr4 17727520 GGGGATTGTTGGGAATGCATCAT 23 61.517 AAATTGCTTATGTCGGTTGATCCC 24 59.902 299

chr4 17736219 TTTCATGGCATGGCATCCTTTTAG 24 60.142 TGATCGAGTCATAATTGTGCCAAC 24 59.667 257

chr4 17736706 TAGACACTCGAACAAATCTCTCCG 24 60.143 ACACACTCTCTTCCTTTCCTTTGT 24 60.08 278

chr4 17741991 ACAAATCCCAAGTGCTAAAGGAATC 25 59.814 AAATTGGGTCTCTTGCTTTTGGAG 24 59.96 100

chr4 17748927 CTTTAGTGTGTGAACATTGGGCTT 24 59.962 TACGTAGGCAATGAAGTAAGGGTT 24 59.777 155

chr4 17765025 CTTGGTCAAAGCTTCACATCAGTT 24 59.963 ATTTATTCGGGTGGGCAATTTTGG 24 60.625 191

chr4 17766504 GTTTAGTAGTGAAATTTGGAGTGGG 25 58.09 GTTGATAGAGTTGAGTGATAAACCC 25 57.459 292

chr4 17835689 AGCTTTTGGACGGGTGATATAGAG 24 60.202 TTTCATTTATTGGGCGAGTCTTCC 24 59.602 235

chr4 17887984 CATGTTCTCTGGATTCTTCAACGG 24 59.906 GGTAGTTTGAGTTCGAAAAGGCAA 24 59.964 232

chr4 17961728 AGACAAATTGAGGGAGAGAAGCTT 24 59.957 CAGAAAGGAGGATAAAACCCCAGA 24 60.018 274

chr4 17961963 CTGGGGTTTTATCCTCCTTTCTGA 24 60.018 CTTTTGCTCTTGATGCTCCAAA 22 57.753 230

chr4 17963839 TCGGTTGTGTGAAAATACTTGTCG 24 60.026 ACAACCAATCGTCCTCTTCCTTTA 24 59.959 174

chr4 17966185 GCTTGAGTTGGGTTGAGTTGAGTA 24 60.982 CTTCGCCGATACACTCAATTGATC 24 60.085 293

chr4 17967178 CAATGGTAGAGCGTGTCCCTAATA 24 59.961 CCTACGGGTGAATAGAAAGTTGGA 24 60.081 234

chr4 17970530 CGATTCCAACCTTGATTGTCTGAG 24 59.906 GACATTTGAAGAAGCGTCCCTATG 24 59.965 297

chr4 17973693 AAGATGGCTTCAGGTTCATGTACT 24 60.019 CATCATTGCCTCGTCCTTTTCTTT 24 60.083 252

chr4 17988428 TGAACAGAAAACGATTGTGAGG 22 57.188 GTAGAGGGGATGAGAGTGTTATGC 24 60.262 186

chr4 18011094 CAACCATTTTCCCCGTTTATTGGA 24 60.021 TTTAGTCACTTACCCATTGCTCGA 24 60.021 181

chr4 18015281 TGGTGGGATATATGGGGAATTTGT 24 59.578 GCCATGAAGAAGTTCCAATTCCAA 24 60.021 191

chr4 18016022 CCTTCTTCCGTTACTGTCACTGTA 24 60.023 TGTGGTATGAGAACTTAAGCGGAA 24 60.021 246

chr4 18019764 ACGTATTGGGTTGAAGGTACATGA 24 60.02 CGGATCCCAAACTACAAACTTGAC 24 60.083 263

chr4 18020382 AATCAGTCCCCATTTCCATAACCA 24 60.017 AGTCGCTGTTATGAGGATGATGAA 24 59.9 241

chr4 18021704 TTCTCAACTAGGCTGCTTCTTCTT 24 59.959 TTATTAGGCGGAAAGGAAAGGGAA 24 60.018 297

chr4 18025715 AGAGAGCTTGAAATGTTTGTGTGG 24 59.963 GAATCAATGGACCGCTCAATTTGA 24 60.142 221

chr4 18029229 AGAACTTGTGAGCTGTGAGATTGT 24 60.444 TCCAGCCACCTTATTAACTTCCAA 24 59.956 174

chr4 18047155 CTGAATGGCGAGCAAGAGTAAGG 23 61.533 GGTAAGTCAAGGTCGAACATAGGT 24 60.082 225

chr4 18048236 CGTGACTATTTCTTTCTCCTCTTTG 25 57.945 ACTAGCTAGCACACTGTTCATGAA 24 60.021 297

chr4 18048491 GTTGAAGAGTTATTTTGGCAACCC 24 59.011 ACCCTCGATCTTTAAAGCACTACA 24 59.778 300

chr4 18050695 ATGGCTAGATGTAAACGATCGTGT 24 60.202 TGGGAAATGACCGTTTAGATTTGTC 25 59.818 247

chr4 18051434 AGCTTTAGGGTTTGGGGAGATAAT 24 59.518 AATACGTGCACACTCCTAGATAGC 24 60.262 182

chr4 18057850 GCGGAGAGTGAAGGCAGTTATATA 24 60.022 CATCTTCAATTTTGGACAAGGGCT 24 60.021 291

chr4 18059205 TTGTCACTTTATACGCCACCATCT 24 60.323 CCCCTCAAGCTTAAATGGTAGAGT 24 60.08 161

chr4 18061028 AAGTGTCGATTTCCTCTCTTTCCA 24 59.96 GCAGTGGGAAATAGAAACATAGCC 24 59.963 191

chr4 18062890 CGTGGGGTATTGGATGAAAATCAG 24 59.963 AAAGGATGAGGGATAACACACACT 24 59.71 155

chr4 18069462 GATAAGAACTTGCACGTATTGAGAG 25 58.072 CCCAACAAACAAATAGGCTCAAGT 24 59.961 234

chr4 18072594 ATATTCTCTCAGTTCGCCCATTCA 24 59.898 GTTAATGGAATGATGTATGACCGTC 25 58.112 140

chr4 18073439 CCTTCAACTAACAGGAACAAATAGCC 26 60.349 GGACCATGGACTTAAACAAGCTTT 24 59.719 235

chr4 18075399 AACTTGGTGTGTTTGGGGAAG 21 58.893 CTTTCACAATCCTTCCCACACAAA 24 59.901 228

chr4 18178499 GTCTAGTTTAACGTGCACTAATCCA 25 59.134 AAAGCCTTACCACAAAATGAGTCG 24 60.023 271

chr4 18408729 TAGGCGAGAAAGTGTCAAATCTGA 24 60.021 ACTTAGCCTTCTCTCGAAACCAAA 24 60.202 273

chr4 18416199 AGTGCATCTACTCCCTCATGTTTT 24 60.019 TGTAAGAACTTGAAGCCACTGTCT 24 60.142 244

chr4 18416691 ATCGTATTCATATGCCCCGTTCTT 24 60.263 CTCTCCTCTATTTTCATCCTCCCG 24 60.022 274

chr4 18418708 CACAACATGACAAGGACACGTTTA 24 59.965 TTGAGCAGCCTGTAAAGAGACTTA 24 59.717 199

chr4 18419159 TCCATGTTTCTTAGCATCCAGAGG 24 60.385 TGCTCACAGAGAAGCTAAAAGTCA 24 60.202 161

chr4 18421003 TCATCTCCATAGCCCGAAGAAAAT 24 59.897 CCTTTGGAAGACAGCTATTTAGAGT 25 58.589 212

chr4 18421548 GCAATTAACCACAGAAGGAAAATCG 25 59.427 GGGTCTTTTGCTCTCTGTTCAAC 23 59.996 291

chr4 18423164 AGCACTTGTACTTAATCGGGTTCA 24 60.263 ATAGGACTTTCTGAGGGGCAAAAT 24 60.017 219

chr4 18427684 AACCAAATGCATTAGGATCAAGCG 24 60.441 TATTACATGTCCAAGGGCCTTCAT 24 59.832 228

chr4 18428288 CACCCGAGCATCTCAATTAGGTT 23 60.683 CAACACAAGCACCGGAATAAAATG 24 59.615 244

chr4 18441619 AAGCTTTCTCATCCACGAAACTTC 24 59.786 AGCAGAAGAGAGATGAATGTTGGT 24 60.02 207

chr4 18452777 CTTATGAGCTAGCGGTTTCTCTCT 24 59.963 TTAGCCAGTTAATCTCGGATCGTT 24 59.901 242

chr4 18454209 CACATCTTTTATGCATGGCTACCT 24 59.418 CCGTAGCTATGTTTTGGGAATGTT 24 59.602 286

chr4 18454769 TCGAGAAAGGACATAGGCAAGAAA 24 60.021 CTTGCAATCGACCTCTGATAACTG 24 59.73 298

chr4 18479605 TGGAGGAAGTGGTAGAAAGTGAAA 24 59.589 CGCAAGGGGTATCTTTAGAAATAGC 25 59.82 263

chr4 18482110 CTGCTGTCTTCAAATATGTTGTGTG 25 59.145 GACCTACGTCGTTTTCCTTCTCTA 24 59.845 296

chr4 18486409 AACACCTTCACTTGAGCTTTGATG 24 59.963 TCGAAATTGCTTGCCATGTCTTTG 24 61.087 241

chr4 18494185 AAAATACGTTCGGCTGCATGGAAA 24 62.107 TCTATCCCTCCATTTGAAGCATGT 24 59.833 217

chr4 18503654 ACTTATCAAATGCCCCAAGTACCT 24 60.017 ACCAATTAGTTACAGCTTCCACCT 24 59.957 291

chr4 18505549 GGCACATTTGAAGAAGAAGAGGTT 24 59.721 TTCTACTCCCCGAACATCAAACTT 24 59.959 238

chr4 18565270 GTTGTTCTTAGAATTGCTACCCATCG 26 60.457 ATTTCTTCCTACCTCTCCATCACA 24 58.967 80

chr4 18570808 GCGGAAATGAAAGACGGTTAAGTA 24 59.61 TCTTCAACTTTCGGTGTCTCTCAA 24 60.142 280

chr4 18575017 GGCCCTTTCTTTATCCCCACTTTA 24 60.571 GGCACACATCTGAGTTAAGTCATG 24 59.906 261

chr4 18586784 ACTTAGTAGCTTAGGGGTGTTACAC 25 59.813 AAGGAGAGGAATGAGTGGTGAAAA 24 59.895 125

chr4 18587204 ATCACTCATTTCCTCAAACGCTTC 24 59.845 CCTCACTCCATTCTTTTCACACAC 24 60.024 122

chr4 18592111 GGTTAGGCTAATCTCTAGTCACCG 24 60.023 TCAAGATTGGTTGGTGGCAAAA 22 59.231 228

chr4 18593633 AGTAGCAAGAGAGAGGACAAAGTG 24 60.022 ACAAGAGGAGGATGAAGACTGAAC 24 60.021 259

chr4 18593952 GTTCAGTCTTCATCCTCCTCTTGT 24 60.021 TTCACATGACGTTGTTCCTACCTA 24 59.719 245

chr4 18601606 TGGGTTCTAAATGTGACCTTAGTTC 25 58.764 GGAGCTTTGTGTCATGTTCAATGA 24 60.023 261

chr4 18620576 GTGTGAGGTCCAAGCTAAAACTTT 24 59.662 TAATTTGAGTTGGGCATTGAGTGG 24 59.78 296

chr4 18624934 AACATCACCTTAACATCCACTTGG 24 59.233 TTCGTTAACTGAAGTGGATTGCAC 24 60.024 289

chr4 18625256 AGCCCTACCAATGAGTACAAACAT 24 60.018 TGCTTATACCGGTTGACTATTGCT 24 60.142 130

chr4 18628380 GTTGAATGCCGGCTAATGTTATGT 24 60.202 TACCTAGAAAACCTCCCTCTTTGC 24 60.02 253

chr4 18636280 TTCAGGCGTAGAAGTATCATCTG 23 57.812 GAAGGGTCTTTGTCTGAGTGATCT 24 60.021 179

chr4 18642692 TCTCTCTTTCTGCTCATCAATCATG 25 58.95 ACTAGATAAAGAAGGGTGCATGAG 24 58.077 229

chr4 18649166 TGTTGCCTACCCTATCTCTTTGTT 24 59.711 GAGCTCTATGTTCTTTCTCTTCGT 24 58.353 268

chr4 18649695 GAAGAATGCATAATGTTGGGAGGG 24 59.962 ACTTCTCTCATTGCACATTCTTCTC 25 59.357 291

chr4 18662566 TAATCCAAAGCATTCCACTCCTGA 24 60.019 AGGGAGATCGATAAAATCCATGGT 24 59.401 262

chr4 18666387 TGCAACATGGTTTATCGGTGATAG 24 59.426 TTAGTACATGTTTGGGGCTAGGAG 24 59.837 187

chr4 18666552 ATAATCCACTCCCACTCTACTCCA 24 60.079 CCCATAAGAACAAAATCCCTACTCC 25 59.172 218

chr4 18667393 AGTGTTGAAAGGAGTGAGGAATCA 24 59.897 CCTTGAGATGCTTGGATTGAGTTT 24 59.538 158

chr4 18675003 CTCTCTTTGGCCCTTAGTCACTAG 24 60.142 AAGATTGAGAGATTGGGTGTTGGA 24 59.957 246

chr4 18677469 CCATGCATAAAAGATCAACGAACG 24 59.27 ACACCCAAGCTTTATGACCTAGAA 24 59.711 245

chr4 18682707 AAGAAGAAGAAGAAGACGACGACA 24 59.964 ACTAAGAAGAGGAGGATGGCATTG 24 60.142 288

chr4 18691193 ACGAGGTCTGATAAGTATGAACCA 24 59.046 GCCCTAATCCTCGCCAAAATATTC 24 60.083 224

chr4 18692698 CGGTGACTTGGAAAATGGAGATAA 24 59.059 CAAGACCGAGAAATCACATGGTTC 24 60.143 139

chr4 18697529 CTCGGGTAGGATTAGAACTGTAGC 24 60.023 TAGGAAGCAAAAGAGAAGTCACCA 24 59.897 253

chr4 18736856 TTCTCTGGAGTGGATTGAATTCGT 24 60.02 CACTCACTTTTCCTTTTCTGACCC 24 59.963 211

chr4 18829896 CCATTCGACCGTTTAACTTTCTACA 25 59.594 TTAGGGGTGTAAGTCCAATCTGAA 24 59.152 292

chr4 18856724 CACAACCACTATACCAGAGCCATA 24 59.899 TTTTAAGGGACCAAGAGAGCATGT 24 60.203 102

chr4 18858391 GCCATGCAAATAATTAGCCCAA 22 57.929 ACTTCTTGTCTCTCCCAATTCTCT 24 59.156 295

chr4 19069205 ACCCACAATCAGTCATCATAACCA 24 60.019 GTTAGTTCGACCAAGAGTTTTGCA 24 59.965 227

chr4 19081993 TAAGCTCATGTTCCACAAAACTGC 24 60.262 GTGGGTAAGTTTTGTTGTAAGCCA 24 59.902 216

chr4 19089166 TCTGTTGGTTTTCGTTCTCAAAGG 24 59.904 CGAGGGGTTTGAGGTAAGATTTGA 24 60.565 196

chr4 19090980 TAATACATGCGGATCAATCACGTG 24 59.553 TGTACACCTACTCTTCTTGATGCA 24 59.473 292

chr4 19094919 CTGACTATACCCTTGGGACTTGAC 24 60.142 TCCAGTTGCTAAGTTGAACTCTGT 24 60.142 269

chr4 19096655 AAGATCGGTTTGGCTTATAGGGTT 24 60.08 TTTACTTGCTTGATGATGATGCCC 24 59.902 244

chr4 19099158 TTTCTTGCAACGTGTACACATAGC 24 60.319 CTGAGAGCTTTTAGGGGAGAAGAG 24 60.142 290

chr4 19105253 CCACCATCAATAGTCCGTATGCTA 24 60.021 CTCAGCTGGAAACCTTTTCTCTTG 24 60.023 254

chr4 19110053 GTACATTTCACTGACGCTTCTGAC 24 60.143 TGTTTGTATCATGAGGCTATTCTGG 25 58.942 285

chr4 19122891 TGACTGACAATGCCCATCAAGTTA 24 60.506 GCCCATGATCTTATCCTAAACCCA 24 60.203 250

chr4 19124131 CCCCTTAATCCTTCTCTTTTCCCA 24 60.017 AGAGAAGCCCTAAATTGTTCGACT 24 60.02 274

chr4 19124257 TTTCACACTCAAACAACTTAGGGC 24 59.903 GAAAACCAGCGTGCATCAAAATAC 24 59.91 296

chr4 19125544 CCATTTTCTTTCGGTCTCTTTGGT 24 59.722 GTTTTGTTGAAAGAATGGGCGTTC 24 60.026 278

chr4 19130642 TAGGCGTTAAATGTACCATCTCCC 24 60.202 CATCACTTGTGCATATTAGGCCAC 24 60.261 284

chr4 19148344 CCCTACCCTGGTTTAATGACGTAT 24 59.898 ACCAGTGTGTAATTAAGATCGTCCA 25 60.047 296

chr4 19153229 AAACAAAGACCTCAAGCTTCATCG 24 60.024 TCAAGTTGAGAAAGGTAAGACGAGT 25 59.931 116

chr4 19155741 AACACACAGAGTACCTAGTAAGCG 24 60.083 CAACCTTGAACCCCTACTGTATCA 24 60.02 281

chr4 19175070 AAGGTTATATACGGTTTGAGGGCA 24 59.835 GTTCTTTCACCTTTCCATCTTCTTC 25 58.327 282

chr4 19178682 CGAAGTACAATGTTGAGGGCATAG 24 59.729 CCCAACCAACCTGAAAATATGCTT 24 60.02 213

chr4 19179695 AGGATCTGCTGTTGAGTTCTTGAT 24 60.02 CGAGGGGTTTCAAGAGATTTCAAG 24 59.845 176

chr4 19180333 AAATCTATGAAGCGTGGACACTTG 24 59.607 CTCAAATTTTCCCCTAGTTTTCAGC 25 58.841 175

chr4 19189622 TTCCAAGACAAAAGCAGAAGACAC 24 59.904 GACATTATTACTCTCTTGCACGGC 24 60.026 147

chr4 19190034 CAAAAGCAGTGGAGTTAATGTGGT 24 59.962 CCCCACTCTTAATCTCTCCCTTTT 24 59.772 242

chr4 19198244 CTTAGAGGTGTGGTCAAAAGAGGG 24 60.804 ATCCAACTTCTTCCTTGCAAAACC 24 60.202 295

chr4 19198716 CCATCACATCACATTTCTCTCTCC 24 59.185 ACACTTGTCTTCTGTAACACCCTA 24 59.348 279

chr4 19209032 GTCTTCTTCACCGTTGTTGATTGG 24 60.555 CTAACCATTTGCCACACTCACAAT 24 60.022 257

chr4 19262743 CAACAGGCAGTAGAAGTAGTTTTCA 25 59.242 TTCATGTCTCTTATGCTCCACCAA 24 60.019 171

chr4 19263576 TCAACAAGAGAAGGAGCCATAGAG 24 59.839 GCTACTATGAACCCACCCTTATCC 24 60.263 269

chr4 19266820 TGCATACATGAGTCAGAATTGTGAG 25 59.417 GGTTTGCAAATATTCCTCTCTTAGCC 26 60.46 157

chr4 19274114 TATTACGCGCGTTAGATGTCAATC 24 59.619 GGGAGTATTTTGTTTGGCTAATCTC 25 58.204 268

chr4 19276217 GTGTTACACGTGGTTTCCTTTT 22 57.64 CCATCTCACATCAGAACACACAAC 24 60.084 261

chr4 19277864 CTGGTTTGGTTTGTTTTGTTTGGG 24 60.083 GCACTAAACCAAGCAACCAAATCA 24 60.739 112

chr4 19278040 GCACTATCCTAGGACCCATTCTTA 24 59.161 TGACAGCACCATCAATTTCTTG 22 57.492 279

chr4 19306397 TTAATGATACTTGCTCGCCCAATG 24 59.724 GCAGTGGGTTAATTGTTCTTCA 22 57.153 263

chr4 19352909 GGTACCAAAAGCTGTGATTAGAACT 25 59.294 CCCCAACATCAAACAAGATCTCTG 24 59.843 215

chr4 19364120 TTTTAGTTTCTTCGGCCCTCATTG 24 59.782 CTGTCCTGTCCTGAAAGCAATTAC 24 59.845 275

chr4 19365117 ACACGTCTAACAAGTTCAATGGTG 24 59.727 GTCTTCCTAAACTTTCACCATGTTG 25 58.561 253

chr4 19369441 CGAACGGGAAGATTATATGTTGGT 24 58.946 ATGCGCAATATCCCTATCTCATGT 24 60.081 252

chr4 19371799 TCTGTGAGGAAGCATTGGAGTTTA 24 59.958 CTCCTTTTATTAACACCCAAAACGC 25 59.368 282

chr4 19381207 CTCCAATGGATTCTTGCAAAACCT 24 60.021 GAAGGAGATGGTAGGGAAGCTTTC 24 60.685 257

chr4 19386219 GCCATAAACATATCGTCGCATTGA 24 60.084 TGAGTTTTGGAAGTGGATTGCTTG 24 60.202 225

chr4 19390408 CATGCATGCAGTTGAGTTTGAAAC 24 60.085 TGTTATGTTGGTCGCAAATTGG 22 58.093 192

chr4 19392148 GTTGGGGAGCAAATGATTTAGATTC 25 58.724 CTTGGATCCCTTTCTTCTTTCCTT 24 58.671 300

chr4 19392420 AATTCATTCACGTCCACTTTCAGG 24 59.784 TGACATACCAAAATGCTACCTCCA 24 60.019 256

chr4 19396394 CGAATGCATTTCCAATGGTATTCC 24 59.016 GAAGGTAATGGGTTAAGAGGAGGG 24 60.141 226

chr4 19426491 CCCGACGTCATACTTTCTACTTCA 24 60.143 GCTAAGCTTACAACACACAACTCA 24 59.727 208

chr4 19440203 AAACGACACCCAACCAATTAATCC 24 60.022 GCACGCGTTGATTCCATCTATTTA 24 60.025 120

chr4 19441380 CGCTCCTTTTAACGCATAAGTCAT 24 59.966 AAGTGTGGTGAGTGCTTTTCTTTC 24 60.142 266

chr4 19443203 CCAATCACAAATTGCCACGTCATT 24 61.092 AATGGACTAGGCTCATGGGTTCAA 24 61.866 298

chr4 19444759 TCCACTCTTCTTTCTCTCCGTTTT 24 59.899 GGTAGGTAGTTGCTGATTGACACT 24 60.323 285

chr4 19444903 TCCACTCTTCTTTCTCTCCGTTTT 24 59.899 GGTAGGTAGTTGCTGATTGACACT 24 60.323 285

chr4 19445633 GACAAATTCTAATGATGCCACGTG 24 58.97 TTTGCTGAACTTTATGGGCTTGAG 24 60.022 216

chr4 19445991 TTTGAAGCTGATCATCCCTCAAGA 24 60.019 TGATTTGTGGTTCGATCTGTAGTCT 25 60.047 176

chr4 19448363 CTGAAGCCAAGTCTCCAATCTTTT 24 59.478 GGGGTGTGACATTGTTGGTTTAAA 24 60.142 258

chr4 19452826 TTTGTCGTGGTATCAGCTCCTTTA 24 60.021 TGAACATTTGGCTTAGGAGGTTTC 24 59.477 238

chr4 19455513 TTTGTTAGAATTTGTGGGCTAGGC 24 59.78 TTACATGGAAAGTCGAGTAGTGGG 24 60.082 195

chr4 19456451 CTCTGGAAAGGGAAGGACTCAC 22 60.028 GCACAAATCCATCTGTTGCTAGAG 24 60.202 255

chr4 19460665 GTTGTGCACTATAGTCGACAATGG 24 59.967 AAAGGATGTGCAGCTATAGTACGT 24 59.9 174

chr4 19461769 CCCTTTTACGTGAGAGTAGGTGAA 24 60.022 CGGCAACAAAATTGATTTCTGGTC 24 59.85 223

chr4 19469644 GTCCCATCTTAAGTTATGCACACA 24 59.059 CACTTCACTTGTTTCTCCATTTACC 25 58.561 250

chr4 19472770 GCTTTTCTGCCATGGTGAGTAATG 24 60.676 ATTTGCAGCTGACAACTTCCATAC 24 60.083 300

chr4 19476816 CTTCCAAACAGTCCTCCAAAAGTG 24 60.202 TGCTGGCCTATATTCTCCACTATC 24 59.532 239

chr4 19477982 GCAAAGCCATTACACAAACTTTCG 24 60.085 TAGACTCTGAAATTGAAGGGGTGG 24 60.019 198

chr4 19479550 TAACAGGATCATGGGTCAATCGAA 24 59.837 GTTGCCACATGAGTTGGAATAAGG 24 60.381 279

chr4 19481588 CTACAATCAAGACCCACAATCTGC 24 59.905 ACATGTTCTTCTCACACTATACCCA 25 59.516 210

chr4 19481980 ATTGAAAGGAGAGACAGAGCACAT 24 60.02 CCGCAAGCCATGATTTTCAGATTT 24 60.917 293

chr4 19482105 ATTGAAAGGAGAGACAGAGCACAT 24 60.02 CCGCAAGCCATGATTTTCAGATTT 24 60.917 293

chr4 19487487 TCCTTATGCAGAACAAGAGACCAA 24 59.958 CACCTCGAATGAATACCCCAAAAG 24 59.904 254

chr4 19491119 ACTCTTACGGTTTGGCTTTATTGC 24 60.083 CATTGTTGCTCTGCTATATGTGTGT 25 59.934 295

chr4 19493032 AACGCGACTATAGAACTCAGTGAA 24 59.845 TTGCTCCCTTCGACTATTTCTTCA 24 60.021 186

chr4 19495014 AACCCAAACAGAAAATACACGAGC 24 60.261 CAGCTTTTCACCTCCATGTTTGAA 24 60.202 232

chr4 19498076 GGGGATGTGAACACCTAGTCTTTT 24 60.507 CGATCGTCCCAATTGAGAGATTTG 24 60.025 223

chr4 19507641 TTGTCCACTACTTAGAGCGTTAGG 24 59.843 TGGCCATGTTGACTTCTCTTATGA 24 60.019 242

chr4 19519960 TCCTCCTTTTCTGTCACTTCTCTT 24 59.344 GGTGACACGATGAATAAGTACACG 24 59.735 263

chr4 19527197 CGGTTTGTTTGGTCCTTTTAGTCA 24 59.903 GCAGAATCAGCACTAGAAACAGTC 24 59.907 281

chr4 19547727 TGGATCATATGAGCGTTCTAAACCA 25 60.163 TCTCCCTCTCCATCACGTAGAATA 24 59.896 146

chr4 19548026 TCACAGAAGCTCACAAACTTTC 22 57.117 AATCTAATGCGGGATCCTTCAGAA 24 59.897 287

chr4 19557765 TGTGGAGCATTTAACAGACAGAGT 24 60.202 GCTGCAGTAAAATCTCCATGTTCA 24 59.843 202

chr4 19561154 GACCACAAAATGACCCAGACAATT 24 59.961 CACTCTTGGCTTGCTGTAATGAAG 24 60.379 279

chr4 19566068 CCATACTGGCGTACTTCATTGTTG 24 60.202 TTAGGCTATACGAGTTAAAGACCG 24 57.935 215

chr4 19572006 CCTCCAATCACAAAGGTATGTTCG 24 59.905 TCTCCCGACATTTACACTTGACTT 24 59.96 254

chr4 19578490 TAGAAGCCTAGCAATCACTTACGG 24 60.202 GTGATTGTAACAAGTGGGATGTAGG 25 59.646 296

chr4 19582133 AAGGATGAGTACAAATGGGATCCC 24 60.141 GAAAACTAAGAAGGCCGGATGATG 24 59.964 283

chr4 19584669 ATGTCTCTCAACCCAACCCAATTA 24 59.956 TCCCATAGCAAAAGCATATGAACG 24 59.724 237

chr4 19586840 TTATGTCTAAACGAGGCTTAGGGG 24 59.9 GGAAGTTTTAGGCTGTTTTGGGAA 24 59.9 250

chr4 19587988 GAAGGGGTAGAAAGGGAAGTTACA 24 59.712 CAGACAAACGTACTAATAGCCACC 24 59.434 101

chr4 19595516 TTAGAGCCTAAACATGACGCATCT 24 60.142 AGGAGGTTATTTTAGCCCAAGTGT 24 59.956 279

chr4 19595870 GATGTGTAGCCTCAAAAGACTCCA 24 60.564 GCGTTTAAGACAAGTTGCCTAAGT 24 60.024 265

chr4 19598109 TGGCTTTCTGATAAGAACTGTCCA 24 59.958 TCTTAATCTTGACAACAGGGCA 22 57.634 236

chr4 19610096 AGGACATGAAGAACAAGGAGAAGG 24 60.263 TAATGGGGAGAAATTCACAAGCCT 24 60.264 272

chr4 19613799 AATTCTTTCATCCCAAGCGATCTC 24 59.422 CTTAGACATACCATGCCCCAGAAT 24 60.203 218

chr4 19626844 TATGAGTTTGAGGGCCGTAATCAA 24 60.081 TAATGAATGCCCTCAACACAAACC 24 60.022 243

chr4 19637199 CATGTTGCTGCAAATCTTCATCAC 24 59.674 AGGAAAAGTTATGAGAGCGACACT 24 60.021 225

chr4 19640564 TGCCATTTTCCTTCCTCACAGATA 24 60.019 AGTCAGCCTGGATTTTAGCCTAAA 24 60.018 282

chr4 19646768 ATGGACTATGTTGGATGGTACTCG 24 59.961 CTTGATCAACCACTGCTTCTGTTT 24 59.963 213

chr4 19654331 AATGAGGCTCAAGTTCAGGATTCT 24 60.019 TGAGCAACAACTGAATCCTAGGAA 24 59.958 292

chr4 19658140 AAAAGATACAAGTCCCGTGCAAAG 24 60.023 TCTTCGGTGCTTGCATTGATTATC 24 59.964 265

chr4 19658704 GTCACAAACCCCATTTAATTCCGA 24 59.781 TTAGTACAGAGGCCCAACTTTCTC 24 60.02 176

chr4 19661987 GTTATCAATCATCCTCTTGGCACA 24 59.116 AAACTTGTTCAACGGTGTGTAGAC 24 59.906 260

chr4 19666803 CATGTCTCGTGTTCCATCATCAAG 24 59.966 ACATGTCGAAGTTAGGTAGATGTGT 25 59.815 224

chr4 19677047 GGAGCATTTGAGACGATGAAAGAC 24 60.202 AATAGCCTGGACCTCAAACGTAAA 24 60.263 105

chr4 19689779 CAATGACATCAAAATCCTCCTCCC 24 59.66 ACCACCTCATTCTTCATTCGTTGA 24 60.505 258

chr4 19694004 ATGACAGAGCTACAAGGAGAACAG 24 60.082 CTATCAAATATAGGGCAATCGTGAC 25 57.704 181

chr4 19696150 AAATTGAGTTGGATCACTGGGTTG 24 59.718 GTGCATTTTAGAGCCATGATGACT 24 59.662 284

chr4 19730509 ACTACCTCTATTTTGCAAGCGTTG 24 59.846 TAACCAACAGGACGCCAATTACTA 24 60.02 108

chr4 19731285 ATATCTTCATACGCGCATCACCAT 24 60.622 GAGACCACATACATATAGGAGCGG 24 60.142 229

chr4 19752591 TGAAGCTTGTCCTTGATCGTCTAA 24 60.021 CAAGTAATGCTCCAAACGAACCTT 24 60.023 289

chr4 19756596 GATGGGAGAGGAATGACACTACTC 24 59.961 CATCATTGGAGATTGTGTGTGAGG 24 59.904 179

chr4 19758015 TAACTCACCCGCCATAGTATGAAG 24 59.961 ACTTCTGAAATGGCTCTCACTCTT 24 59.959 282

chr4 19758425 GATAATTTGGGAAGAGTCGAACTGT 25 59.125 GCTTTGGCGACTTGAATTTCATGA 24 60.852 299

chr4 19759379 TACAAACCCAGAAGTTACAGCAGT 24 60.142 TGTTGCTACTAACCACTCTAAAAGG 25 58.772 289

chr4 19761221 TCTATAAAGCTCTCAAGACGTGCA 24 59.842 TCTGATAGCAAAAGCTCAATCGTC 24 59.432 300

chr4 19764830 CGACTGTGAATCTCATCGTTCTTG 24 59.969 GATTGAGTATGGCCACGAAAGAAG 24 59.965 258

chr4 19772006 AGTAGAGCTTTACGGACGGTTTAG 24 60.143 CCCCAAATAATCTTTCTCCCAAACA 25 59.516 210

chr4 19773626 AGATTTTGACTACAGTGTTGGGTAC 25 59.004 TTCATTATTGTCGCAACTCCAACC 24 60.083 172

chr4 19831588 AGAAATTAGCATGGCATGTTGGAC 24 60.142 GGTCAACATAACCCTATACCCTTGA 25 59.869 294

chr4 19896020 AGGGTTGTTTTCGAATAGAGTCAGA 25 59.988 GGGGACTTTGTTGCGTTTTACA 22 59.901 228

chr4 19896422 CATCATGGGCTATTGGTCAGAAAC 24 59.963 TTCCAGCCCAGTCTCGTAATAATC 24 60.202 91

chr4 19897143 GTGTGTGTCAAACGTCTATTAAGTG 25 58.647 GAACGCTTTGACAACTTCTGGT 22 59.645 300

chr4 19971451 TTAAAGAGGAAGGAAAGAGGGACG 24 60.02 TACATCACGACCCTTAGAAGCTTC 24 60.142 292

chr4 19971574 TTAAAGAGGAAGGAAAGAGGGACG 24 60.02 TACATCACGACCCTTAGAAGCTTC 24 60.142 292

chr4 19982919 TTGTTTTGAGGGATTTGGGAATGG 24 59.959 AGTCGATAACCATCTGATAGCTCT 24 58.679 239

chr4 19983206 CCCTTAAAACCGCGTCTATGAAAA 24 59.845 GCAATACACCCACTACAAAACTAGG 25 59.877 109

chr4 19987515 GAGAGGCATTCAGAGAAGTAGAGG 24 59.962 GGTTTGCACACACTCATACAATCA 24 60.023 239

chr4 19989165 AGGTGCATCGAGTATATCAAGGTC 24 60.022 GCAAAGCTCAGTCCACATATACAA 24 59.364 159

chr4 19991086 TCAGTCAAGTTGTCATACCCCAAT 24 59.957 CCAAGTAGAGAAGACGGTTGAAGA 24 60.022 296

chr4 19995655 CCAATTGCCATCACCATTTCTTTC 24 59.367 GGAAGGTTTAAAGGGAGGGGTTAA 24 60.203 272

chr4 20001776 CACTTCTTCCTCACCAATTGCAAT 24 60.022 GTAGACGAGTGAAAGAACCTCCAT 24 60.082 259

chr4 20002603 ACGATAATCAAACGAAACCCTTCC 24 59.606 ATTCACTGGTCTCGTTTAGCTCAT 24 60.081 124

chr4 20003694 GCAGCCTCAAACATTTCAATTGTG 24 60.084 GGATTGAAACATTTGAAGACGTCG 24 59.154 245

chr4 20014973 TTTTCCTTTTGAGACAAGCATCCC 24 59.961 TATGAAGGTTTGGGTTGTCAGGTT 24 60.387 271

chr4 20016427 AAAGAACTACATGATCAGGTGGGT 24 59.71 ACAGGCCTACAATGGGAAATACAA 24 60.264 243

chr4 20016721 TTGTATTTCCCATTGTAGGCCTGT 24 60.264 ACACACTCATATCGACTCTCAACC 24 60.142 281

chr4 20263848 GTTTGGCATTAGAGAGGAGAGTGA 24 60.081 TGGGATTGAGACACATCATCAGAA 24 59.774 227

chr4 20267655 TGGTTCAATTTGAGGCAGGTATCT 24 60.264 GCTCAACTGATACCTGTATTTTCCTG 26 59.962 300

chr4 20272147 GGATTCTGTGTCATCTGAGTTCCT 24 60.081 TGTTTGTTGGTTTCACAGAAAGGG 24 60.322 155

chr4 20273361 AAAGGTGTCATATGGTAGGTGCAT 24 60.08 TAAAGGGAAACAACCAAAAGCCTC 24 59.9 150

chr4 20278322 ACCGGAAAATTTCACTGGGATACT 24 60.264 TTACAAGTTGGACCGAATGTTGTC 24 59.726 295

chr4 20359291 TTAGATTCTAAAGTTCCGCCCCAA 24 60.019 CTCGAGGCATCTTTGATTGTGTCT 24 61.155 239

chr4 20365588 GTGGGATCTCTTTTGGTGGGATAT 24 60.141 GGTATGGACTATGTATGTGGATTGA 25 57.995 269

chr4 20366478 AATCTGTGAGTGAGAGCTTAGGGA 24 60.816 CATGGTTTGGTGTCTTGAGTTTGA 24 59.902 291

chr4 20368080 TTGCTAGCCAACTATTGACAATCG 24 59.667 GATAATGGTCGTTGTTTGCTGGAA 24 60.083 200

chr4 20368710 ACTGAACTCTCTCCATTCTTCTTCT 25 59.22 AAGGAAAGGTAAGAGGAGATGGTG 24 59.774 263

chr4 20381524 CAGACGACTCTACCGAATTTTGTG 24 59.91 ATTGATTTCTACTTGTCCGGTCCT 24 59.775 149

chr4 20381711 AGGACCGGACAAGTAGAAATCAAT 24 59.775 TCCACTCCCCATCCTATCTAAAGA 24 59.828 195

chr4 20382581 ACCGAAAGAGAGAAGTGAGATTTC 24 58.525 CTTGGTAGTGATGGATCGAAAATCA 25 59.183 246

chr4 20386330 GTCCAAGATCTTCTGGTAGGGAAA 24 59.775 ACATGCTTGATACCCTTTCGTACT 24 60.081 201

chr4 20390869 AAATCAAACTGCTACCTCTCCCAA 24 60.203 AGCGAAATAGACAGTGGCTTACC 23 60.679 249

chr4 20392745 AAATCCATTCTTCTTTTGGCAGCC 24 60.564 CTCATTGTGGTAATTATCCTCGTCA 25 58.719 164

chr4 20399205 CACAAAGTAGAAGTGGTTGGCTG 23 59.996 TTTGTCAACTTTCCGCTTTACTCC 24 59.964 299

chr4 20403757 TTTCAATTCGAGTGTAGCAACCAG 24 59.787 ATCAGCAAAAGCATAACCTCTTCG 24 59.905 129

chr4 20406255 GTTTGATCCTTATTTCGCTACGCA 24 59.966 CCCTCTATAATCACTACCGAAACCC 25 60.279 286

chr4 20406365 CTCCAACAAACCAGTTCATTCTCC 24 60.023 GTGACATTAAAGTTGAAGGCTGGA 24 59.481 291

chr4 20409052 GGTATCTTTAGCTGAAACGAAGGC 24 59.966 GGTCTTGTAATTCGAGCTTGTTCT 24 59.309 286

chr4 20426540 CAGAAGTGTCCCGATAGCTAGAAA 24 59.902 GAGAAGATTAGGAGAGGGCTGAAG 24 59.961 243

chr4 20428432 CCCTCACACCCTCACATTACAATA 24 60.081 TCCAGAAATCATATGTAGGAGGCA 24 59.093 171

chr4 20435599 TTAGCTAGCGTGAGAGAGACATTC 24 59.964 TGCCACTTACCATGCTAGATCTTT 24 60.08 134

chr4 20445005 AGGTAGAAAAGTTGGGTCGTTACA 24 59.899 CCATACCAAAACTTGTCATTCCAGT 25 59.756 180

chr4 20627040 TGTAATCCAAACACACCAGAAGTC 24 59.18 TTTTGGTTGTCTTGCGAATTAGGG 24 60.262 271

chr4 20627719 GTAGTTGACATGCGAGTGAGAGTA 24 60.143 AATTATGTTCTCAGCTCCCCACTT 24 60.018 266

chr4 20633151 TGTAATCCAAACACACCAGAAGTC 24 59.18 TTTTGGTTGTCTTGCGAATTAGGG 24 60.262 285

chr4 20688828 GGAACAAGATCGAATAGGGAGCT 23 59.93 CAACCTGTTTATGACCCAACCAAT 24 59.717 148

chr4 20689628 CTACTTTTGCCAACGATCGAAACT 24 60.084 GTTCCAAAGTATCACAAAGAGTACG 25 58.182 265

chr4 20693821 ATTAAGGAATCTCGGGCTATCACG 24 60.322 CAGAAATAGCAGACCACAACAACA 24 59.724 249

chr4 20703710 CCCACATTGATAAAGTTGGAAGAGG 25 59.874 GCTAATCTTAATGTCGGTTGCTCC 24 60.025 242

chr4 20717649 AAAGTGTGAGAAGCATGTGACAAG 24 59.964 TTCAATATCAGATGCTCCGGTGAA 24 60.142 255

chr4 20719578 GAGGGTCGTTTTGCATTTTGTTCT 24 60.736 TGCATAAGACTGACCAACTCTCTT 24 59.716 244

chr4 20719818 CGTTGCCCCATCTACTTACTTACT 24 60.142 CGTGTGATCCTCATCCTCATCAAT 24 60.503 99

chr4 20731110 TGGAAAGGTTGGAAATTTTAGGCC 24 59.959 GGATGGGATTGAATGGTGTGTTTT 24 60.02 170

chr4 20741011 GAATAATGGGACCGAAATGTGTGG 24 60.202 CCACTTTAATTGGTAGGGAAGGAG 24 58.806 273

chr4 20744053 GATGTTCCATGCACGTGTAAGTAG 24 59.967 GCTAACCTCTAAATGTCCAATGTGC 25 60.449 259

chr4 20744247 GCACATTGGACATTTAGAGGTTAGC 25 60.449 AGTCCTCCTCAACAAACTAGACAC 24 59.961 287

chr4 20745432 TTCAAACGAAGGGGTACATACTAC 24 58.277 ATATGTTTAGCACGCAGAGATCCA 24 60.202 234

chr4 20749665 CAGGAAGAAAGTTGAAATGAGGGA 24 58.927 TCACTTCCATAGAATTAGCTCGCA 24 59.901 244

chr4 20761292 CACAGACGGCGTTCATCTTTT 21 59.469 CCCATCTATATTGCACCCTGTATGA 25 60.046 300

chr4 20766303 TGGCTATCAATTGTTAGGGGAGTT 24 59.77 ATCCATACTACAACGAAGGAACCT 24 59.285 249

chr4 20766744 TACCTTTCTTTCCAACGACTCGAT 24 60.022 GTGAAATGCATGTTGTGTTAAGCG 24 60.144 272

chr4 20766913 GCTTTGCATATACGCTTGATGACT 24 60.025 TGATCAGACGTTAAGGCCAAAGTA 24 60.021 199

chr4 20771318 CCCATCATGACACATAACTTACACC 25 59.703 CAAGGCTGATTCTCTCGTTTCCTA 24 60.382 253

chr4 20773815 TAATTTGGGAGAGTTGGGTTGGAT 24 59.955 GGAAGTTATTGCAGGAGTTGACAC 24 60.083 145

chr4 20784105 CACTAGATTCTGTTTCTTTGCGGG 24 60.143 CCATTTGCAAGCATGTTACATTGG 24 59.906 289

chr4 20785546 CACACTCAGTATTTTAGACGTTTCC 25 58.182 CCAGAGGCCACACTATAGAAATTC 24 58.94 296

chr4 20786448 CTTTGGTTCCATTGTGCTTCCTAA 24 59.719 GCCACCAACCTAACTTTTCCAATT 24 60.202 213

chr4 20793350 GCTCATCCGTTGGTAGTCTTATCA 24 60.202 ACAACCATCTCCAACTTTCTTCCT 24 60.141 300

chr4 20795857 TTCAACTTCAAGAACATCACGTGC 24 60.496 TGATGATTGACTTGAATTGGCCTG 24 59.841 193

chr4 20796273 AGTCGGTTCAAGGTGATCAACAAT 24 60.747 TTTTAACGTCCAACTCCAATGCAG 24 60.261 176

chr4 20796937 CCTAGGAGAAGTTCAACATTCCGA 24 60.082 TTGTTTGTTCCCTGTGCTGTTATC 24 59.963 159

chr4 20804914 AAAGACTTGTTTGAGGGAAGCATG 24 59.962 CCTTCCTCTCTTGATTCTTCACCA 24 60.02 265

chr4 20814848 GTAAGATTTCTCATACGCTTGGGC 24 60.025 GAAATTTCACCAGAGAGCAAGCTT 24 60.023 148

chr4 20816877 TGAGTGTTCCCTTAAATGGTGTCT 24 59.896 TACCCTTGGGTTACTTCTAGTCCT 24 59.954 157

chr4 20835751 TGTTAGACCCCTTATCATCGTAACA 25 59.342 GCTCATACATATTCAGAGACTGCC 24 59.071 299

chr4 20839802 GCCAGGCCTTTAAGTTTAACTGTT 24 59.961 ACACCAAAGACACTCCACTTAACT 24 60.081 209

chr4 20839974 AGTTAAGTGGAGTGTCTTTGGTGT 24 60.081 AATAGAACGGTGTTGATCAAGGGA 24 60.02 178

chr4 20843217 GGAATAGTCACCTCCACACTCTTT 24 60.02 GGTTTAACGACTGCAGTGATGATA 24 59.132 300

chr4 20843543 ACCTCGCTATACTTTGCCAATGTA 24 60.142 ACTATCGAGAGCTCTTATCTGGGA 24 59.958 259

chr4 20857042 CGGAGAAATTTGGAAAATGGGGAT 24 59.838 AAATTGGCTTTAAAACCCCTCCTG 24 59.958 284

chr4 20871792 GACCGTTTTCCAAATACATCCAACT 25 60.048 GCCATAAATTCGGGTCGTAGTCT 23 60.492 287

chr4 20872322 GATGCACGAAGAGAAATGGGTTTT 24 60.321 TGAAGCGTTAGACACCACTATTCA 24 60.022 192

chr4 20879054 CCTCTAGGGTTTGCTTTCTTTTCA 24 59.173 ACCCTTGCTGACCTGAGAAATTAA 24 60.203 283

chr4 20885588 TGAGTCTTGAACAGTAGGTAGAGA 24 58.123 AATACCCTCACTCGCATGTTAACT 24 60.081 213

chr4 20919566 GAGCTTGCATTATTAACCACCACA 24 59.842 ACTTGACGGATATCCTCTTTTGGT 24 59.775 199

chr4 20934490 TCAGCAATTATGTGAGTTGGGACA 24 60.506 CACTTTGGCTGTAATAGAATAGGGT 25 58.643 260

chr4 20937327 TCCTTAGGTTGGCTCAAAAGTGTA 24 59.897 TGTGTGTGAGCGAGAAATGTTATG 24 59.848 181

chr4 20945318 CTAGCCAACTGTTCCGAATGATTG 24 60.202 TCTTGTAGTGAATGTGAGGCTGAA 24 59.96 255

chr4 20946504 CTTTCCCTCAAGCTGCAACAAATA 24 60.022 GCTTCAATATTACCGTGTCAGCTA 24 58.952 276

chr4 20955046 TGAGCTCCTTAATTTGTGCTGAAG 24 59.543 GGACGCAATATTAACAGTACAGGG 24 59.49 235

chr4 20961581 TGATCGGGACCAATTCAGTAAGTT 24 60.02 GAAGAGATAAGCGGAGGAAGTAGG 24 60.023 208

chr4 20962109 GTTATACTCGGAATGCAGTGTGTG 24 59.967 CAGGGACCTCATCAATTCCATACA 24 60.141 238

chr4 20976492 GAACAGTGGAGGCTTTGGATTGAG 24 61.872 CTGTTTCTTTTATGGCTTCTGGGG 24 60.082 223

chr4 20976592 TTGAGACGGAGGTGGACTATATTC 24 59.417 CTGTTTCTTTTATGGCTTCTGGGG 24 60.082 204

chr4 20977804 CTTCACTGCTCCAATGGATTCTTC 24 59.904 GCTTTGGAATGAAGAGCTGGAAAT 24 60.082 227

chr4 20978481 GCCCACAACTTCACATACATCAAA 24 60.022 CAATGTTATGACCCAACCCGAAAG 24 60.38 181

chr4 20983386 CATGCTCTAAATTCTACCCTCTTCA 25 58.414 CATATGTGTTTGTGTACTGTGTGGA 25 59.532 242

chr4 20989386 GGCATCTCGGTACAACTAAGACTT 24 60.382 AAGAGAGTTATACGTACGGCAAGA 24 59.362 289

chr4 20991880 ATATCTCCACTCGCATGTCACCTA 24 60.993 CAAACGTCAATGTCCAAGATCAGT 24 59.786 162

chr4 20997886 GCTTTGTGATTTGGCATAGGATGT 24 60.142 TGAGGAGTAAATGAAGAGAGAGAGA 25 58.274 199

chr4 21019679 TGGAAAATACTAAATATGCCCCGTG 25 59.467 CGATCCCGTACCAAATCTAAACG 23 59.512 298

chr4 21020962 TGACGTTTGAATAAATCCGCTG 22 57.665 TATTTACAGGGCGAGGCTTAAACT 24 60.081 255

chr4 21022670 TGGGAGCCATACAAACTGATAGT 23 59.22 CTGAGAGTTCTGTGTGGATAGACA 24 59.539 242

chr4 21046407 GGTAAAATGTCACAAATGCCCTCA 24 60.022 ATCGGGTGTATCAAGAAGTATCGG 24 60.022 102

chr4 21049096 AAGAAGCAAGAGAGTGAGAACCAT 24 59.959 TCCCTCTGTCTAAAACCTTTTGCT 24 60.141 296

chr4 21051642 TCCACCTCTCAAAATCTTTCCCAT 24 59.955 TCGTAGGAAGACAACATGCAAAAC 24 60.024 296

chr4 21052279 CCGATTCCCCAAACTCAAAAGAAA 24 59.961 AATATCCATCCACCACCCTTATCG 24 60.019 156

chr4 21058480 GTCCCTCTAAACTTATCAGCTCCT 24 59.348 TCTCTTCCACACACAACAAAAC 22 57.305 286

chr4 21058780 GTTTTGTTGTGTGTGGAAGAGA 22 57.305 TGCGAGTTCAATGACAGAAGTTTC 24 60.025 194

chr4 21065261 CAATAACCGAATCAAACTCAGCCA 24 59.844 TTGTTCTCTTTGTGTTCCCTTTGG 24 59.84 186

chr4 21065658 CAACGTGGCTGATTATTGAACCAA 24 60.321 GTGTGAGCAGTGAAGAAGTTGATC 24 60.084 266

chr4 21068087 AAAATCCCTATGAATACGCCTGGA 24 59.896 TGAGAAATGTGGGATAGTCTTGGG 24 60.08 124

chr4 21075274 GGCATATCAAGATGTAAGTTTGTGAGG 27 60.468 GAGCTCTCTTCATCTCTCCAACAT 24 59.9 288

chr4 21079116 CATGGCTGTGTAGTGCAAACTG 22 60.351 TGGGCTAAGAGAAGAGGGTCT 21 59.637 300

chr4 21083047 TCGTAGATGGTAGATGTTTGGGTG 24 60.142 CTTCCATTGAACACATGTCCACAA 24 59.962 271

chr4 21083537 GAGAGACCCAAATATAACCCCTCC 24 59.958 TGTTAAGTCGAGGCTTTGATACCA 24 60.021 192

chr4 21095981 TCAGGTGGAAGGAGAAACATCAAG 24 60.506 GGTCACACGTTTGGTTATTATTTGG 25 59.14 300

chr4 21098044 CTCAAAAGCTCTCGTTCGATTGAA 24 59.849 AAAAGCCGAAGAAGATGAGGAGAT 24 60.081 223

chr4 21098867 ATGGAATTTGGTTTGGATGCCTAC 24 59.838 TATGGCGAGTCATGATCACTTCTT 24 59.9 298

chr4 21099120 AGATAGAGCCCGAACTAGGACTAA 24 59.896 AACGCATCATGTCATCTAGCTTTG 24 59.965 274

chr4 21102937 GACCATTTCCATCAATTTCGAGCT 24 59.903 GGTTCTCCTGCTAGCTCATATGAA 24 59.96 203

chr4 21103462 GCAGATTATTGGCCAAGCTTAGAC 24 60.261 ACTTCGACCAACTACAAGCTCTAG 24 60.083 266

chr4 21110144 AGTTCCTCTGATCAGCTAGCTAGA 24 60.141 CAAGTATAGCGACCACCATTGATG 24 59.788 247

chr4 21111448 ACAAATGATTCTCTACCCTTCCCC 24 60.08 TCCCTCTATCTCCCTCTGATTCTC 24 59.956 160

chr4 21115642 CCCACTCTACACTTTACATTCCGT 24 60.322 CGACTCCTATTACGTGAAGTTGAA 24 58.604 116

chr4 21118014 TCCAAAACCATCCACTTTCTCTCA 24 60.141 CATGTGTCACTCCATCTATGTTGC 24 59.965 142

chr4 21124783 ATGCGTCGGGGAAGAGTTTAATTT 24 61.106 GTAACTACAACGCACTAGAGCATT 24 59.137 240

chr4 21134653 GGTAATGGATCGAATTCTAATGGCA 25 59.295 TGGTGGCACTGTATACAAACTACA 24 59.959 243

chr4 21147895 TGTAGGAAGAATGGGGTTAATTTGG 25 59.045 CTCAAAGCTCCCTCATGTGATCAG 24 61.217 229

chr4 21150558 CCTTGCTGAATCGGGGAGAAT 21 60.134 CCTCTCTCCTCCCTCTCAAGAA 22 60.024 292

chr4 21167790 ATTTCTTGTACTGAAGGGGATGGG 24 60.325 GAAGGGGATTGAAGAAAACACTCT 24 58.927 242

chr4 21170635 AGAAGATCTCATCCTCATCTCCCC 24 60.757 AGTTTGCAACATACACTGACCAAG 24 59.963 299

chr4 21172904 GTCACACGTGTTACTTCTTTATTGC 25 59.156 AATATTCAAGATGGTGACGTGACG 24 59.433 92

chr4 21176247 GCAAAGTTTCTAAGTCCGTTGAGA 24 59.49 TCTACAGTAAATACAGCACCCATCA 25 59.576 119

chr4 21177660 TAGAAGGGCCAAATAAGTCCATGT 24 59.77 TGTGATGCTTGTCAAATGGGTAAG 24 59.782 93

chr4 21179714 GCTACATTTACTACTACCAGGCCA 24 59.9 CGACCTTTAACCTCAAACGTTTCA 24 59.966 242

chr4 21186847 GCGGCAAGTGATCAACCATC 20 59.901 TTTGTTCATTTGGAGGGAAGCAAG 24 60.202 225

chr4 21200297 AAGGTAGTAGCAGACGAATTTGGT 24 60.02 TGATGTCGATGATGAAACAAACCC 24 59.844 221

chr4 21209640 AGTCAGGTCCAGGAAATCTAGGTA 24 60.016 TTCAATCCCAAATCGAACCACAAG 24 60.022 162

chr4 21211562 CGAAGATTAAGGCAATAATGTCCCC 25 60.049 TGAGCAAGGCGAACTAAGTTTTAC 24 59.787 291

chr4 21223214 TCTTCTCTCACCAAAAGCTGAACT 24 60.142 ACAAGGTGAATTTGGAGGAGTGTA 24 59.896 233

chr4 21232339 CGATCCAAACGAGGTCAAAATCAA 24 60.084 TTTTAATGACTTGGTGATGGCTGG 24 59.78 161

chr4 21234086 GCAACCATGGAGTAAAAGGATCAC 24 60.142 CCAAGAACATGAATCATTCCCACA 24 59.536 182

chr4 21236088 GTTTTCCATGGTCAACAAGATCG 23 58.524 CTGTGCGCACGAGTAATCTTATTC 24 60.317 155

chr4 21236567 AAGAATAGTGAGAGAGAGCAAGTC 24 57.792 TCTCTCCATTGTACCCTTGAGTTT 24 59.402 144

chr4 21239248 TTGCTTAACTTAGTGCTGTCATGC 24 60.083 AAGCCATTCACCTCCGAAACAATT 24 61.536 252

chr4 21239752 AGTGGAGAGTAGAGACGAAGATGT 24 60.324 ATCGTCCCAGATTACCACCACAT 23 61.458 281

chr4 21240074 CAACCTAATTTTCATAGCACCATCG 25 58.798 TCCTCCTAGTCCTCCCCATAAAAT 24 60.077 286

chr4 21240329 ATTTTATGGGGAGGACTAGGAGGA 24 60.077 TGCCGATCCAACAACTACATGATA 24 60.142 167

chr4 21240878 AGATGAAAGGTAGAGATGTCCCAC 24 59.593 AGAGAGTTCTTCCAACGATCAGAA 24 59.475 296

chr4 21245649 AGAGAGTTAACGGAATCATGGACT 24 59.288 TGATCTTCGGTGTCAAGTTTCAAC 24 59.727 211

chr4 21248449 GCCAACCAAACAACCAAATTACTC 24 59.49 AAAACTTTTCCAGCTCTCTTCCAC 24 59.66 237

chr4 21252330 GAGTCTACTTATCAATCAGGCGGT 24 59.962 CAACAAGAGAACAAGTGTGGGTAT 24 59.178 235

chr4 21260312 TCGGTCATCCCAATCTGTTTACTT 24 60.02 ATGGGCAGTGTGAACATAGTTAGT 24 60.019 263

chr4 21269879 ATTAGGCAGTGAATGAAGGAAGGT 24 60.018 AGCTCTCTCACTAACTCAAGGAAA 24 59.164 205

chr4 21272647 TCAAGTTCTAAACAAGCCATGC 22 57.496 TTTCTGCGGACTTATTCAAACACC 24 60.024 276

chr4 21279098 GTCATGCAACCATTCGACTGTAAT 24 59.905 ATGATCTTTCTTTCTCGCCATCCT 24 60.142 240

chr4 21314620 AGAATCTCCAGGCCCATAACTTTT 24 60.017 CTCCCTACCCTTTTAAGCACTTCT 24 60.019 250

chr4 21320453 CCTTAATATCTATGCTCGCCCCTT 24 60.081 AGAAAATGGTCGTTCGTTCCTCTA 24 60.022 253

chr4 21320716 TAGAGGAACGAACGACCATTTTCT 24 60.022 GCAAACTGGTAGAATGGTCAAACA 24 59.963 277

chr4 21327631 CCGCCAGCCATTATTTGATATCAG 24 60.143 GGAAACCATCTATATGCAACAACCA 25 59.64 290

chr4 21340141 CTCGCTGCCTTCTCATTTTACTTT 24 59.845 ACATTGAACGTACATCTCACACCT 24 60.262 241

chr4 21343041 AGAGGGCAATGTGTAGAGAAATGA 24 59.774 GAGGATCCAAGTGTCTGTTTTGAA 24 59.178 294

chr4 21371567 AGCAGTTAGTTGGTTAGGAATCAGA 25 59.752 ACTTGTTGTGTGTTAAAGCTCAGG 24 59.904 283

chr4 21372212 CTCCGTATTGATATCAGCCCTTGT 24 60.263 GGGAGAGTGATAAGGTGTGTTACA 24 59.778 300

chr4 21376215 TTTCTTTCGGAGGCTCTAATGGAA 24 60.02 GCCAGTGGAAGTAACTAGTGAAAC 24 59.549 285

chr4 21380096 AGTTTCCTTCCTCTCACGTTATCA 24 59.473 AGATCAGATTAGCTTGCTATCGGT 24 59.474 299

chr4 21383887 CCCTCCATTGTTGTTCCTTTGATT 24 59.716 TAGAGCAAAGACAACACATCGAGT 24 60.262 169

chr4 21396010 TCATGTTCCATGCCCTCTAATGAT 24 59.895 TGATCGTGGCATTTATTGTCTTGG 24 59.904 217

chr4 21397408 TCATTTACAATCTCACCTCCCACG 24 60.623 ATGAATTTCACAAAGGGGAGGGAT 24 60.265 298

chr4 21403218 ACGTCCGATCACAAAGTAAAACAC 24 60.025 CTATTTTGAGGTTTGAAGTAGGGGT 25 58.754 273

chr4 21403789 ATATCCAGGCTTCAACACAGACTT 24 60.019 AACGGGTGTGGTTGATGAAATAAC 24 60.023 279

chr4 21415715 TCAATGTCCTCGCCTGTTAATACA 24 60.081 TCCCTTGTCTGCTCGTGTTAATAA 24 60.021 286

chr4 21416737 TTGGTGAGTTGATATCGACCTCTG 24 60.142 CACCCAGCAAAGTACAAGGTAATT 24 59.477 240

chr4 21476096 TACGACCGTGTATCATTTCCTAC 23 57.826 GTAGGTAGTACAATATCGGCCACA 24 59.721 185

chr4 21482764 TTTGTGAAGTTTTGAGTTGAGGGG 24 59.84 AAATTAAGGGTAAGGGAGTGTGGG 24 60.264 285

chr4 21483839 ACCCATACATGCAAGATTCCAATG 24 59.657 GGTCATACATATCCCTTCCCCTTC 24 60.019 171

chr4 21485852 TGCAATTCTTAAGACGTGGAGAGA 24 60.021 TCTTTCCCATTTCCCATAACACCT 24 59.955 223

chr4 21485996 AGGTGTTATGGGAAATGGGAAAGA 24 59.955 AGAGAGAGAGAGAAAGAAAGGGGA 24 59.955 294

chr4 21489472 GTGGGCCAAATTTAAACGATCATC 24 59.196 CCCAATGCTTAGTAGGTGTAATACA 25 58.412 247

chr4 21492052 GGCAGTGAATCATACCTTTGTGTT 24 59.782 TGAGTCTTGTCTTGTGGATATCAGA 25 59.283 279

chr4 21492819 CAAAGTGTTGGACCATGATGAAAAG 25 59.307 TAATTGGCTTTTCTCATCCGTTCC 24 59.602 288

chr4 21505724 GCTCCACGGGAACATACGATTATA 24 60.321 CAGTTATTGGGACGAGAGCTCTAA 24 59.902 236

chr4 21516848 CTGTAAGCATCGACATGGAACTTG 24 60.202 GTGTTTGGTTAGTTTGTTCTGGCT 24 60.142 285

chr4 21522543 AAATGGTTATGACATGGTTGAGCG 24 60.142 GGTTTTCGAGACTTAGTAGCATGC 24 59.967 281

chr4 21549024 GTAGAAGATAAGAGAGCGGTGGAA 24 59.661 ACTTTTGTGCCCTAGAAGTACTCA 24 59.653 139

chr4 21549928 GTTCGAGATGTGAAATATAGTGCAC 25 58.359 GAACATGAAAGTAAACACACACGAG 25 59.098 176

chr4 21551692 CCCCTCCCCTCTATATGAACAATG 24 60.019 TACACGAAGATCATACTCACAGGC 24 60.202 182

chr4 21557727 TTCTTTTCACGATTGGATTGGAGC 24 60.083 TTGAGAGAAGGAAAGAGAAGGGTT 24 59.339 122

chr4 21575560 ACTTAACTTAATGAGCTTCTCCCCA 25 59.749 GTCCAATGTCCTCTCCCAAATAGT 24 60.08 197

chr4 21576237 ATGTGATCCCGAGTTGTTCATGTA 24 60.081 AAATGGAAGGTTTATTGGGCAAGC 24 60.565 264

chr4 21579808 AGCCTTGACAACGGAAGATTTTAC 24 59.784 ATGATAGGAATGTCTCTAACGCGT 24 59.721 238

chr4 21579919 ACGCGTTAGAGACATTCCTATCAT 24 59.721 CTCTCCTACTCCGTGAAGAAGAAG 24 59.904 172

chr4 21581097 AGCCATATTCATAGTGTTACTGGGA 25 59.393 CCTCATCTGGTTTCTGTTGGAA 22 58.245 299

chr4 21594731 ACTTCGATGCCAACTATGATCAGA 24 59.9 GTGTGTGAACCTTTGACTGTACAG 24 59.966 230

chr4 21596496 TGAGTTCCTAGGGTTCACTGGATA 24 60.265 CTGAAAACATGTTGTGCAGCTAGT 24 60.261 277

chr4 21606778 TCTCGAGAACTTTGTTGTACCCAT 24 59.96 ACCAACGCTAGATACCACTTTGTT 24 60.504 222

chr4 21633466 ACCTCTCAACCCCTCTAAACATTC 24 60.019 TGTAAAAGGACTTGTCTCGATCTTG 25 59.073 287

chr4 21650846 GAAACCCTTCTCGCTATTACAAGT 24 58.823 ACAAGAAGAGCAAGATGAGAAGGT 24 59.959 215

chr4 21676363 CAACCACACTGCCTTGAAGAATT 23 59.931 TGGGTTCCAAAATGAGAGGTGTAT 24 59.956 221

chr4 21685368 CATTTTGGTCCTTGTACTTTGAGAC 25 58.561 AGTCCAATCAAGCGTGTTACTAGT 24 60.021 270

chr4 21701553 CTCCAAGAACCAAGATTGTGCATC 24 60.38 ATGTACGAGGTTGGTTTGGTCATA 24 60.02 248

chr4 21706119 AAATGTTAATTCTCCAAGGGCTGC 24 60.081 GATACTTCATCTTGGTCATTGGCG 24 60.025 253

chr4 21716248 ATGATCAAAATGCCCCTGACTGTA 24 60.325 GGAACACTTGACAAACACAAAGCA 24 60.854 277

chr4 21729669 TCGGTGGCTTAGTATAACCCAATT 24 59.835 GGACATCAAATAGCATTCAAGGCA 24 59.902 83

chr4 21732722 GCCAATAATTTTGCCCTTTCCAAG 24 59.604 TTTGTTGACGTCCCTGAATCTACT 24 59.96 172

chr4 21742211 TTAAGTTTCCTCCTCCCCAAGATG 24 60.018 TTAGTTCTTCCACTCATCGCCTAG 24 59.902 270

chr4 21743601 CAACACATACGACATTTACACGCT 24 60.143 TTCAATTCTCTACGTGAAGGAGCA 24 60.021 219

chr4 21759392 GCTCATGCCTAGATCTAAAACATTG 25 58.334 AGTCATCTTCTTTGCACATGAATCC 25 59.875 282

chr4 21766184 ACTCTTGATTTGAATGATTGCCCC 24 59.839 GGGACAAAGAAAACGTGAGCTAAA 24 59.964 224

chr4 21772595 CACATTACGCCCAATTCTTCATGA 24 59.904 CACGATCATTTTCCATAGACAACAC 25 58.749 289

chr4 21775828 GTCATGCATTGTCGTTCAGTCATA 24 59.67 CCTAAGTAAATGAACCAAGCGCAT 24 59.904 297

chr4 21779640 GTCTATTCCTCACTTCTCTTCTTCGA 26 59.906 AATAGTGTATGCATGGGTGTCGAA 24 60.384 228

chr4 21782547 GTTGGACGCCTTATTCAGAGAAAC 24 60.143 ACGAGTTCATAGTTGGCTCAGAAT 24 60.081 246

chr4 21803835 AAGATACGCGGTTTCAATGTTGTG 24 60.613 GGTGGTAAGCATAAATTTTGAAGCG 25 59.712 176

chr4 21808359 AATGACGTCTAACTTTCCATGCAC 24 59.846 CCATTCTGCTGTATGTATTGCTCG 24 60.084 262

chr4 21809788 CGAATGACTATCTGAAAAGGCTAAC 25 58.061 CCTACAATTAGAAAACATGCCTAGC 25 58.271 298

chr4 21810316 AACGACCAGATTTTGAACCATGTC 24 60.023 ACAATCGTTAAGCTAAAGTGGTGC 24 60.083 197

chr4 21810673 CACATCTAATTACTAAGTGGTCGCA 25 58.96 CCAACCCTCTTTTCTACTCCAAGA 24 59.958 271

chr4 21813821 CATTGCGAAGAAGGGAAGAAATGA 24 59.843 GAAAAGTCGTGAAAATTGCCCAAC 24 60.026 296

chr4 21816977 GTTGAAATGTTGAAGTGCGATTGG 24 59.851 TCACCATGTAGTCTGTACACCCTA 24 60.264 270

chr4 21821347 GTAGATTTGGATTTCTCGGGTGGA 24 60.384 CCGAATCATAACAATTTCTCTCCAC 25 58.281 263

chr4 21831038 AAACTTGATCCTTAACGGCTACCT 24 60.02 TTGTAGCCGCGTGATTCTTTTATC 24 59.966 282

chr4 21834746 GGAGGGCTAAAATTCTTCGGGTAA 24 60.627 GGTGAGAAACGACAATCAAGAATCA 25 59.821 291

chr4 21835369 CCATGGCAGCGGAATATTTTACAA 24 60.202 TCGGTGGATTGTAGATTTAGGGAC 24 59.9 177

chr4 21836140 TGGGAGGGAAATAGACTTGAGAGA 24 60.265 ACTCCACAACTACTACAAAAGGGT 24 59.589 94

chr4 21838593 ACTTGCAACCATTGATCTAAGTGC 24 60.083 GGAGCACACAGTTCAATAATGTCA 24 59.544 299

chr4 21840224 TTTCCCGAATGCAGTTAAACAGTG 24 60.261 AGGACCTCACGAAATATATGGTGG 24 59.96 248

chr4 21867395 TCATGTTCCAACCTTGAGACTGAT 24 59.958 GTTACCAATGTCGCCAGAACATAG 24 59.966 245

chr4 21874846 CAAATGTCTGTCACCATCCCAAAG 24 60.321 TATGGCTTCTTCATTTCCTCAGCT 24 60.08 290

chr4 21876165 GTGTAGCCCAACCATTACAACATT 24 59.78 GCGATGCAAACCAGTCTCTTTAAA 24 60.32 153

chr4 21883965 CCCTCATTTCAACTACACGATTGG 24 59.905 GTGGTTAACTCCGTTGAAGTCCTT 24 60.981 292

chr4 21885510 GACGTCAGGAATACTTTAGCGGA 23 60.181 GTATTTCTCCATATTGTGTGTGCCA 25 59.643 292

chr4 21898387 CTGTCGTCAAATCTTCTGTATGCC 24 59.967 GTGGATAAAGTAGGGAACGAGGTT 24 60.081 268

chr4 21969153 TTTTATGACCGTTGTTGGCCAA 22 59.306 AACAGTCATGGAAAAGGATCAACG 24 59.784 263

chr4 21979479 CACAAAAGCCTCCAAGTTTCTTGT 24 60.382 ATTCCCTCTTCTTGGTGATAACCC 24 60.08 200

chr4 22005890 TGAGGACGCAATCTGATATCCAAT 24 59.959 GAGAAACCCTACTTACATGCATGC 24 59.964 210

chr4 22018976 TTTTCTTCATTCGTGGGTTCGTTC 24 60.261 AAGGGAGTAAAGGAAAAGAGGGAA 24 59.332 232

chr4 22025603 TTCACTTTCCAGGTAGAGGTCTTG 24 59.96 GTGAAGCCAACCGATGTCATTATT 24 59.904 288

chr4 22026667 GAAACCCAAAACAATACCTCCCAA 24 59.654 TTTCTCTGACGCATATTCAACGAC 24 59.674 144

chr4 22029014 CTCTTCACAGGTCATTGGTTCATG 24 59.845 TAAGCCTCATCCATAACACGAGAG 24 59.962 295

chr4 22030247 ATTAGAAAAGGTGCAAAAGGGG 22 57.11 TTTTGTGTTAGCAAGTTGGGTCTC 24 59.903 195

chr4 22031473 AGGGCTTAGACTTACTCCTCTAGG 24 60.141 TAGAACCATTGACAAACACAAGGC 24 59.963 213

chr4 22032733 AGTCTAAGGGTGTCTGGAGATGTA 24 60.017 AAACCACACCCTCAATGAGTTTTG 24 60.142 205

chr4 22051266 ACAAGAATAGGCCAAAAGAAGTGC 24 60.022 ATTCCTCTCCCAACCCGATTTTAA 24 60.018 282

chr4 22055639 TGGAGTGTAATGGGTTATGGAGTC 24 59.837 GAGTTTCAAGTTTCGTCCCCAAAT 24 59.962 221

chr4 22056026 TTTCAAGAATGTTGTTCGGTGACC 24 60.202 TTGCTGGATCAATGTCTTAACTGC 24 59.843 266

chr4 22060829 ACTACTGGACCGTCGAACATAAAA 24 60.022 TTAACAGAACACCAGTCCATAGCA 24 59.959 249

chr4 22062350 GGAGAGAGGATGTTTGTCAAGGAT 24 60.081 ACATGCTAAAAGACTTCCCTCAGT 24 59.958 156

chr4 22065672 GAAATTAGGGGTGAAATCGGGTTG 24 60.142 TATACAACCCGACAACTCAATCCA 24 59.777 165

chr4 22069890 GGACACCCACGTTATCTTCCATAT 24 60.202 ACTTTTCTTCGTCGAATTCAGAGC 24 59.849 223

chr4 22071987 CGAGAATAAAGAAGCAAGCTAGGC 24 60.025 TGATTGACCTAGTACCAGAGACCA 24 60.264 265

chr4 22073373 CCGTCAACCAACAAATCACCTATC 24 60.143 TCTATTTAATCTCCTTCACAGTGCC 25 58.651 236

chr4 22074386 AAGAAGCCAATGTCGTACAAGTTG 24 60.024 CCGGACGAACATAAATGAAATGCT 24 60.202 291

chr4 22076228 TAGAAGTATCAGCAGGACGACATC 24 59.724 ATACCTTCCCCGTAGACAAATGAC 24 60.142 288

chr4 22080466 TCACAAGATAACAAGGAGACATCGA 25 59.815 TGCCACGAAGCTATAAGTACATGT 24 60.142 265

chr4 22084850 AATCAGGTAAAGGGGTAGAGGAGA 24 60.016 TCGGTAAGTTTTGAGGATGATCGT 24 60.082 248

chr4 22093450 ACACCTTTATGTCCTCTTTTCCCT 24 59.646 TCTCTCTCTCTCTCTCTCTCTCTCT 25 60.104 235

chr4 22106575 ATGTTGACGCAATTTTAGGCCTAG 24 59.904 GTAAGGGTAAGGAAAGTGAGACGT 24 60.022 200

chr4 22111669 AGGGTTATTACTTGAGTGATGGTCT 25 59.273 CATCTTTGATTCCACACACACCTC 24 60.083 229

chr4 22115229 CAAGGGCTAACAACATAAATGGCA 24 60.082 TGGTGGTTAGGATTTAAAGTAGGGA 25 59.208 165

chr4 22164130 TTTCATCAGTGCTTTGCCTTTCTG 24 60.5 CAGTTCGTGGACAAAAGAGACAAG 24 60.26 227

chr4 22165415 TGGAACTTCGATGGTGATATGTGT 24 60.081 TCTTAAATGCCCCTACCTAAGTGA 24 58.966 165

chr4 22167089 GCAACTTTGATTTATCTTCGCGGT 24 60.672 CGGAAGATTCTGCAAACGCTTAAA 24 60.613 281

chr4 22221582 GTTGAAGAGGAGTGCAGATTCATG 24 59.905 TTTGTGCTTACTAGATCTTGCAGC 24 59.607 221

chr4 22238843 ACTGTTGTTGCTAAGGAGGATGAT 24 60.019 TTTCTCTCCTTTCCTCCAACTCTG 24 59.959 286

chr4 22244649 TTTCTTTCCCCTTTTGTTGGTGAC 24 60.081 TCAAATTGGCAAAGTATTCCCGAC 24 60.082 236

chr4 22245391 CGAAATCTCCAACCAATATCGACG 24 60.085 GGACGATAAACAGTGAGGACAATC 24 59.433 221

chr4 22249710 GATATTTTGGGCAGGTTGTCATCA 24 59.598 GTTGGAGGGTGTAGTTGGGATATT 24 60.08 292

chr4 22253911 CCTCCACTCTTCCTCCTTTGTTAG 24 60.323 CCCTTTACCTCCATTCCTCTCAAT 24 59.833 300

chr4 22256163 CCAATAAGTTTCCCAATAGAGTGCA 25 59.348 TGAGATGTGGGTATTTCAAGCT 22 57.425 281

chr4 22258731 GAGAAGAAGGTTGAAGGATTTGCG 24 60.379 CCCAAATGGTATTGCGACGATAAC 24 60.554 276

chr4 22259845 TTGGGTGTTATATTCGGGATTGGA 24 59.834 CACAAAGAAAACAAGCCGACAAAG 24 59.968 276

chr4 22260397 TGCTTTGAAGATTGAGGGTTGATG 24 59.781 AATGCTTCCAAACAGTGACGAAC 23 60.243 288

chr4 22260644 CCTTAGCCCTACCCAAACAAGATC 24 60.928 GTTCCTTCGACTGTTTCTTTGCTT 24 60.202 156

chr4 22268017 GGACGAGTTTGATGCTAGGGG 21 60.473 AGACGGATGAAACTAATAGGCTTTC 25 58.95 291

chr4 22268378 CATCCGTCTTTCTTATTTGTGCTGA 25 59.878 TACAGACTTCTCCTCACTCCCTAA 24 59.708 277

chr4 22282131 GAAAGATGAAGGAAGAGGGAAGGA 24 59.774 TTTACGGGTGGATGTAGAGTTCAG 24 60.082 162

chr4 22296075 GTTCCTCCGCAAATGAAACCTAAA 24 60.023 TCCTTCGTCATCAAGATCGTCATT 24 60.142 294

chr4 22297878 CCAACGGTTAGGATGAAAACATGT 24 59.782 CAGTGATACTTCCTAACAAAGCACA 25 59.299 190

chr4 22298760 TTTTAGCAGCTTGGGAAGTAACC 23 59.176 GGTAAGTGCATTAAGTGAGAAGAAC 25 58.167 276

chr4 22298884 GTTCTTCTCACTTAATGCACTTACC 25 58.167 ACTAATTGAAGGTTGGAGGATTCGA 25 60.046 157

chr4 22303988 ACTCTAAAACACACCTTGGGACTT 24 60.08 AAATGCTTCGTCTATTGTGTAGCG 24 59.967 133

chr4 22317676 AAGAAGAACAAAGTTAGGGGTCGT 24 60.142 ATCATACTTGCACGCCTCTTAGTA 24 59.659 255

chr4 22321721 GGCTAAAATCGCAATGTGAAGCA 23 60.673 AAATTTTGGACGCTTGATATGGGG 24 60.142 226

chr4 22328626 CCTTACGATGGTGGATTGCAAATT 24 60.142 TGGACTTCTAGAATCAATCCGTAGT 25 59.108 272

chr4 22330243 AACCCAACCCAACTCTTAACTCTT 24 60.079 GTGTAAGTAAGGGGTCGAGTTGAT 24 60.082 262

chr4 22334895 CACACCCAAGCTTTATGACTTCAA 24 59.722 AAAAGTCTAAAGTCTCAAGGCCCA 24 60.141 177

chr4 22337423 CAGATGCTGGAAATGATGGAGAAC 24 59.964 AGATTTATTATGACCCTTGGGTGTC 25 58.634 188

chr4 22345438 AGGGTTCATAGAATGGTAAGGCTT 24 59.521 AGGAATTTGGAAGTTCTCAGGTCA 24 59.895 280

chr4 22345784 GCCTCACATTGACTAGCTACATTC 24 59.49 CGCTATTCACGTTTCCATGGATAA 24 59.49 272

chr4 22347894 TCGCGATGTAGTGAAGAGTTTAGG 24 60.438 ATGTGCTGATAAAGAAGGTTGCTC 24 59.603 217

chr4 22359965 GTCAAAATTCTTGGCTTGCAACAG 24 60.025 ATTAACAACCTTTTCCCTTTGCCC 24 60.202 270

chr4 22360319 GGGCAAAGGGAAAAGGTTGTTAAT 24 60.202 CTGCATGCATTTGATTTCAGTTCG 24 59.969 217

chr4 22362131 TGTGTCTAGTAGGTGCCTGAATTT 24 59.714 GACGAACAGATTGCAACTATCACC 24 60.202 276

chr4 22362543 GTTGTTCTAAGACCAAGCCTGTTC 24 60.024 TACTCTCGAACACCAAAATGCATC 24 59.608 285

chr4 22365602 AAATATGGGATGAGACGGATGTGG 24 60.506 ATCAAAGCACAGTGGAAAGACTTG 24 59.963 201

chr4 22370092 TAGCTATGCGGTCGTTAAGTCTAC 24 60.025 CCCAATTTTCTACCATCAGCACTC 24 59.903 271

chr4 22376276 TGCCCTCTGAACATAACTTTCTCT 24 59.713 CAAAGATGATTGCTTGACCCCTTT 24 60.021 260

chr4 22384199 ACAATTTAGTCCCCAAACACGT 22 58.442 CGTTGGTGTACTTGTTCTTTCA 22 57.132 233

chr4 22390931 TGGCAAGTGTATCCTCTTCATCTT 24 59.774 TCAGTCCTTTTATTTGGTTGGCTTC 25 59.989 250

chr4 22403602 GGCGTTGCTACTTTAGTTATGTCA 24 59.372 GTAACACTTGTCGAAAAGCTCCTC 24 60.084 273

chr4 22414183 GTTAACCAAAGGACTCAGCTTCTG 24 59.786 AGAGGGAAGAAAGAGAAAGTGCAT 24 59.957 286

chr4 22418696 CCCACTAAAGGTAGACAAGAAGCA 24 60.263 GACATCTGTGCACCTATCTACCTT 24 59.9 143

chr4 22420383 AGCCAAAAGAGAGAGTTGAGAAGT 24 59.898 AACCCCAACTTTTGAGATCACAAC 24 59.901 137

chr4 22422022 GGGATGCCAAATGAGTGTCAAA 22 59.438 TGGGGCCCAATGTTGTATAAAT 22 57.999 299

chr4 22427145 TGAGGATGGGATGTTTGTTAGGAA 24 59.709 AGGGAACATGAAATCTAGCAGTGT 24 60.019 208

chr4 22428263 GATTGGGTAGCAAGGTGATTTGAG 24 59.903 ATTCGGTGCATGAACTCTCCATAT 24 60.202 155

chr4 22432825 TGAGCCATCCTTTGTTAAATGCTC 24 59.842 CATAAAGTTCAGCCAATTCCTCCA 24 59.294 216

chr4 22435060 GATGACGAAAACGAGTATGTGAGC 24 60.259 TGGCCATTCTTCTTCTTTACTGGA 24 59.957 276

chr4 22435792 AGATTGATGAGGAAGGATGCAGTT 24 60.08 ATTACGGATTGGGTTGGGTTGAA 23 60.501 208

chr4 22453869 CTGGCCCAAAGAGTTACAAAAGAC 24 60.262 CTCAAAGGCCTGCACATCAATAAT 24 59.901 286

chr4 22455568 GTAGCTGAAGTATAATTTCCCATGG 25 57.559 GCGAGATATAGGAAGGAAACGTTG 24 59.555 184

chr4 22511455 TAGTGGATTAAGATGGCTACGCTC 24 60.022 CTTTTCCATGACGTCCCATTTGAA 24 60.022 213

chr4 22568868 TTTCAAAACCTAACCACACCATGC 24 60.442 GTTTATGAGTGAACTTGGAACCTC 24 57.757 214

chr4 22573424 ACTTATATACGTGCATGGCCCTAT 24 59.53 GAAAGCTAAGATACGACATGCCAC 24 60.026 280

chr4 22584438 GGACCAATATGACATAACACTTCAACC 27 60.415 AGACATTTGTTACGATCTCTCTCCT 25 59.345 247

chr4 22585343 TGTATTGCGTAGACAGATCATCGT 24 59.964 TCTCCACTTCCAAAGCAACAATTC 24 59.962 298

chr4 22585568 CTTAAAATGGTGGTGGAGTGCAAT 24 60.021 ATTCAACACTATTCGAACGAGACG 24 59.442 120

chr4 22593033 CATACGAGGGAGTAGAAACACACA 24 60.082 CCTTTGTGGTGGATGGTATTCTAA 24 58.494 236

chr4 22607735 TGGATCTGTTTGGCTGAATCTAGT 24 59.774 AGACCATCAGAGCAAATGAAGAGT 24 60.02 252

chr4 22614358 TTCCCTCCATATGCATTCCTCATT 24 59.893 TGCGTTTAGGAAGGGACAAATCTA 24 60.02 256

chr4 22615889 TGTTGAGACATTTTCCCAAGCT 22 58.435 TGTGGGGTAGACTTGAATTTTGTC 24 59.175 163

chr4 22616097 GACAAAATTCAAGTCTACCCCACA 24 59.175 GTATCGACAATGCAACAAACGT 22 58.194 279

chr4 22616509 CACTTGAGATTCTCTGTTGATACAC 25 57.712 CAAGGCGAATTTGGGTCCAA 20 59.035 285

chr4 22618622 GGGTGGATTGTTCTATTACAGCAC 24 59.664 CACTGATGAGAGTTGGTTGTTATAG 25 57.469 287

chr4 22620626 TGGGTCGGTCAAACTTCTTCATTA 24 60.202 GTGCATTTTCGGTTTGTAAGGGTA 24 60.023 259

chr4 22621453 AGAAGGTACCACGGCATTTTCTAT 24 60.081 TGCTAAAGGTAGAGATAACACGCA 24 59.841 224

chr4 22640969 TTGGATTTGACGGCTAAACTCAAC 24 60.024 ACGTCTATGTCCACTAAAAGAGGG 24 59.84 231

chr4 22662858 CTTGCAGAGAAAGAAAGGTGTACC 24 59.786 CTTTCATGCATCCTCTTTGATCACA 25 59.875 194

chr4 22663218 TGTGATCAAAGAGGATGCATGAAAG 25 59.875 TGTCTTCATGCATACCAATCTTACC 25 59.177 283

chr4 22671494 GAGTAGGGTGGCAGATCAATTACA 24 60.142 TGTTATTTGGAACTTAGTTGGGCC 24 59.475 296

chr4 22674209 ACATCATCATTATAACCCTTCCCC 24 58.108 GGCACATATTGGACATTAAGGGTG 24 59.962 232

chr4 22674934 GAGTCATATCTTGTGTCTCGAGCT 24 59.964 TATTGGTGATTTTGGGCCTAGGAA 24 60.017 114

chr4 22687093 CAAAATTTCAACGACCGGTCATCT 24 60.32 ACGATTGTTCAGATCTTGGTAAACG 25 59.88 137

chr4 22701577 TTATAGAACTTGAAATCCGCTGCG 24 59.73 CTGCAATTCTACCCCGTTTCTTAC 24 59.906 300

chr4 22726420 ACAAGTCAAAGTCTAAGTCCACCA 24 59.837 TATTAAAATGTGGGCTGGGTTTCC 24 59.532 164

chr4 22744360 GTCCAACGTCCATGAAGATAATAGA 25 58.487 GTGAAAGGAACCTCGTCAAATAGA 24 58.764 300

chr4 22746048 CCAATACACCTTCCTGCAAACAAA 24 60.202 TTGCATTGAGACGTTAGTAGATGC 24 59.433 146

chr4 22755693 TTAGTCGTTAGTGTCACTGCTTCA 24 59.963 GTCAGCAAGAATCTTGAAGAACCA 24 59.483 206

chr4 22814084 GTCAACTACTACACATTCTTCGCA 24 59.078 CCGACTTCAGACTCCAATTTCAAG 24 59.847 198

chr4 22818470 GCTTTGTTTGTTTGTTTGGTACCC 24 59.906 GGATGACATAAGGACAAAGTAGAGT 25 58.121 271

chr4 22852331 GGGTTGTTTTGTTGGCTAGAATGT 24 60.202 TGGTGATTGATGGTTTTAGGTTGC 24 60.022 245

chr4 22852650 ATGAGGAGGAAATGTCAATGCAAC 24 59.841 CATGTTTGGATTGGGTGTTTTACG 24 59.316 255

chr4 22855060 CTCATCATTCATCACGTTAACAACC 25 58.749 ACACAGTATCTTATGTCCACACGT 24 59.78 207

chr4 22858970 CGAAGATTGAAGCTCCTTTGAAGA 24 59.307 ATCTACGCTTGATTTGGAGGAAAC 24 59.364 297

chr4 22862383 GGTTTACGGCTCGATTGAAATGAG 24 60.494 GGAGTCCACCGAAGAAGAAAGAAA 24 60.743 282

chr4 22868954 GCCACATTTAGCATTATAAAGACGG 25 58.857 CTTCCTTTCAAAATCCCCTCCATG 24 59.839 244

chr4 22873543 TAAAGAGATGTTTGGAGCAGGGAG 24 60.324 TCTTTCTCTTTGACCAAATCCCCA 24 60.141 291

chr4 22875700 GAAATGGAAGAGGAAGCAGGTTTC 24 60.083 GAGGTGGGATATATGTCACGTGAG 24 60.321 201

chr4 22941583 ACCCATAAGAAGGAACCTCAAACT 24 59.646 CCCCACGTCAAACAAGCTATTATC 24 59.965 126

chr4 22948324 CTGTTCCAGTGGTAGTTTGTTTGG 24 60.202 GAAGAGTTCTCTTTCCCACGGA 22 59.701 230

chr4 22949069 GTGATGAGATAAAGGCACAAACCA 24 59.541 GTTACCCACTCACCTTCTTCAGAT 24 60.02 206

chr4 22960220 AGACTTTGGTGGAGAGTGATTGAA 24 59.897 TGGGTATTTGAAGACGAGAATGGT 24 60.02 290

chr4 22962598 CATCAATGGCTCCAAATACTCCAC 24 59.963 ACTCTGCTATTCCAACGGTAACTT 24 60.02 264

chr4 22977059 CTCCACTCTCCATCCCCATTTTG 23 60.939 TGATGAAGCGAACAATATGTCG 22 57.223 274

chr4 22981888 GGTTGATCGATATGGTTGCAATCA 24 59.724 AAGGCTCGTTTCGTTACATGTTTC 24 60.319 258

chr4 22990641 GCATTGCCCAAATTTGTTGATCAG 24 60.143 TGATGGATGGGTCGTTAACAGAAT 24 60.081 236

chr4 22995554 AAGTGTTTGGTCCGAAGAAAACTC 24 59.904 GCTCGTAATCTCCAATCACACTTG 24 59.967 200

chr4 23009422 CAAAGTCTGGACACCTGTAATTGG 24 59.784 AAGGAAATGGTTTTGGACAAGGTC 24 59.899 295

chr4 23024612 ACCTACACACTGTACCTACCATCT 24 60.264 CAATGTGAAGGAGAACGGACAATC 24 60.143 199

chr4 23030699 GGTCACACAGTATTACCAAAACACA 25 59.703 ACTTGAGATTGGATTATGGGCAAC 24 59.354 233

chr4 23032148 ATGGTAGAAACGTTGTGAAGTTGG 24 59.725 AGCATGTCGAAATAGGGAAGCTAT 24 59.959 267

chr4 23034721 TTCTTACGACCAACATCATCTCCC 24 60.383 CTTTGGAATCACGGTAGATGGAGA 24 60.142 297

chr4 23036997 GGAGGAAAACCTAAAATTTGGACCC 25 60.28 CTCACCGCTACTGATTTTGGTTTT 24 60.023 189

chr4 23039199 TCCATCACCATCAGTTTAGGGAAG 24 60.08 ACATCAGAGTCTATGAAACCGCTT 24 60.081 229

chr4 23045248 TGGCACTTTGGTAGGATTCTTAGT 24 59.711 CCATGGCATTTATTTTCACCCA 22 57.515 292

chr4 23057254 TGGGGTTACAGCAGAAATGAAGTA 24 59.958 TTTTCTACCGCAACCCCATGT 21 60.203 231

chr4 23077540 GGGCCAAACACAGAAAGATAATCA 24 59.538 CTGGTACCATATCCTACAAGCCAA 24 59.897 277

chr4 23080411 CCCAACAAATAACCAACCCAGAAA 24 59.898 TGTTGATTGGGAGGTTTTCTTTCTC 25 59.698 216

chr4 23080520 CAAAACAACTTCTTCTTTGATGCCC 25 59.765 TGTTTAGGGCTGGATTGGTATTGA 24 60.018 232

chr4 23081547 CTAATTCAACGCTCATGTCCTCAC 24 59.967 TACCGTATAGTCGTTCAAATGGGG 24 60.202 214

chr4 23082158 TGAGAGAGGGAGAGAGATGGTTAG 24 60.141 CCCTACCTCCTCATCTTCTTCTTC 24 59.656 128

chr4 23159659 GGAGGAAAGATAATGAAATAAGGGCC 26 59.79 GCCCAAATAACACTTTACACACCA 24 59.962 258

chr4 23171711 GAACGGGCCAAGTACTTTTCATTT 24 60.262 ACTTGATGACCTAGATAGACACCT 24 58.228 290

chr4 23175095 ACGGTTGCAATATCATCTCTAAGC 24 59.248 CCCAAAATGATGTGACAAGGACTT 24 59.718 156

chr4 23175228 AAGTCCTTGTCACATCATTTTGGG 24 59.718 GCAATTCCATTAATCCACCGAGAT 24 59.48 215

chr4 23187279 TGAACCTCTAAGCTCGTTTGATGA 24 60.021 TGATGTCTAGGCTTCTCTTTGCTT 24 60.02 273

chr4 23191963 TTTTGGAAAAGTGTGATGATCCGG 24 60.022 AGTAGGTGTAAGAAACTCGTCGTC 24 60.084 274

chr4 23197557 CTTGGATCCATTTGCTTGTTGAGT 24 60.022 GGAAACATACCCTACTCTCCAAT 23 57.368 248

chr4 23212893 TTTAGACTACCCATCGAGCCAAAA 24 60.02 CGTACGTGAATGTATTTGAGTGTGT 25 59.882 295

chr4 23218814 GAAAGGGAGAGAGGTTTAGGGTTT 24 59.957 TGTACCATTTAAAAGAGACCGACCT 25 59.988 198

chr4 23226830 CCCTTTTCATTCTTTTAGCTTAGCG 25 58.97 CGCTTAGTACCTGCATGAAGTTTA 24 59.132 207

chr4 23230855 ACCAGAATATGCAACAAGAGGCTA 24 60.08 GGCACTTATTCTTTGACCGATGTA 24 59.126 142

chr4 23232430 CACCAATGGCTAGCTAATTTCCAG 24 59.963 GATTCGTGCAATGTGGTAAAGAGT 24 59.846 243

chr4 23234814 CAGTACTAGTTTTGACTAAGGGTTG 25 57.346 CAACTGACCCTTGACTTGAAAACA 24 59.842 274

chr4 23254141 CACTGTATTCATGATAGACCATACCTC 27 58.968 CCTTTACAATTATCACGTCTTGGGA 25 59.122 300

chr4 23280858 AAAGTAAGAAGGATGAGTGGTGCG 24 61.098 GTCTTGTTGGTGACCTCAAAATGT 24 59.902 211

chr4 23320073 GAGACTCGGTAACAAGGACATGAT 24 60.142 CCCTAAAACTTCCGCAAATAAACTC 25 58.909 248

chr4 23337619 TGAGCAAGTAAGGAATGGAAGGAA 24 59.957 CCTCCATTCCCTTTTCTCTCTCTC 24 60.142 280

chr4 23337913 GGGAGAAGGAGGAAATTCAGAGTT 24 60.019 AACACTGTCCTTTTCCGTCTTTTC 24 59.904 151

chr4 23339083 ACGACTAATGCATCCAACTCTATCA 25 59.931 AGAGACATTTGGTGGGATACTTCT 24 59.215 286

chr4 23340555 GTGGTAGCTTTTAAATTTTGGGGC 24 59.306 GAATCCAGGACGAATGTGATGAAC 24 59.965 251

chr4 23342850 AAAACAGCAATCAACAGTATCCCG 24 60.083 CCAAGCTGAGAGAGAAAGAGAGAG 24 60.142 82

chr4 23348977 AGACTTATGGATGCTAGGAGGACT 24 60.141 GATTATGAGTTGAGGGAGGGTGAG 24 60.202 275

chr4 23349311 CCTATCTAGCGGCATTTCTCTCTT 24 60.022 TAGCAACCTTAAAGACCTCCAGAC 24 60.02 88

chr4 23362781 GAAGTTGTGAGTATTGAGTGTGGC 24 60.084 TTCCAACACAAAAGATACACAGCC 24 59.963 237

chr4 23369235 TTTGTTCAATGATTTCCTGGCCTC 24 60.021 AGTGTAGATTGTTACGTGTGGAGA 24 59.478 193

chr4 23370679 GCCAAATCTGAAGTCCTTAAACCC 24 60.082 CGAATTGGTTAATCTTGCAAGGGT 24 60.082 299

chr4 23446683 ACCGACTCTTGGATTTGATGGTAA 24 60.02 GGGACCCAAACATCAATAATCGTC 24 59.964 289

chr4 23450215 AACTAAAGTCTAGGGTGGGTTGTG 24 60.202 CGTAAGCTTAGACTTTAAAAGGGCT 25 59.358 155

chr4 23484570 GCATGTGACCCTCCCCATTATTAT 24 60.509 ATGTCTTATCCATCTGCCAGTCAA 24 59.835 292

chr4 23486886 GTGTACCACCTACTACCTTCCTTC 24 59.841 CCTTTGCACCTCAGTTTTCTCAAT 24 59.962 260

chr4 23490141 ATGGCTCCAATTCACTTCCTTTTG 24 60.021 TCCATATACAACGTCAGAAGTCCA 24 59.292 183

chr4 23490642 CGCTAGAGAGAGTTTCGTGAGTTA 24 59.907 AACCAGCAGTAGTGTTAGTCCAAT 24 59.958 99

chr4 23502634 TGGAGATTTCAGACACTTTCACCT 24 59.897 AGAACTTCTCATTGCTTCTGGT 22 57.636 245

chr4 23506284 AGAGGGATCAAATAGACTGCTTGG 24 60.142 TCGGCAACAATAGAGAGAAAGACA 24 60.021 141

chr4 23516094 ACTTGTGTCTTCGTATTTGGCTTG 24 60.024 AGGATAGGAGAATGCATGGTTGTT 24 60.08 151

chr4 23517460 TACTGCCATTGTTGTGAAAGGAAG 24 59.722 AAGAAACGGTACTACAAGACTCGT 24 59.723 215

chr4 23549782 TAGAACTTTGAACTACAGCCGTCG 24 60.85 GAGTGGAAATGAAGTCGAGAGGAT 24 60.142 172

chr4 23551083 AGGAGATTGGAACCTTCTACGAAT 24 59.283 ATCTCCATCGAACCAAAATCATGC 24 59.963 232

chr4 23558059 TACAAGAGATGGAGGGTAGGGTAA 24 59.766 GGAAGAGAGTGTGTGTGAGGTAAT 24 60.021 239

chr4 23561491 TGTGCCAAAGTTGATCCCTATT 22 57.954 CCCTAAAGCTTGGAGTTGAGTTTG 24 60.023 298

chr4 23575319 AAAGTACGACCCATAACACCCTAC 24 60.081 GTCAAGCTTACCAAATTTTGTGCC 24 59.789 236

chr4 23575824 ACCAACCCAATCCAATCTAATCCA 24 60.017 TCATCTTCGTGTCCTTTGTGTACT 24 59.961 256

chr4 23584821 GGCATCAAACAAAGGGGTCATAAA 24 60.021 TCAACTCTGATGAACACTTCTCCA 24 59.655 283

chr4 23586365 TTCGTTGGCTTTCTCTTCATTTCC 24 60.023 CCTGCAGACATAACACTAATTCGG 24 59.729 292

chr4 23600233 CTAATCGGCACCCAAAACTCTTTT 24 60.022 CGTCGGTCTCTTTATCTATTTAGTCAC 27 59.413 246

chr4 23600495 GTGACTAAATAGATAAAGAGACCGACG 27 59.413 GAGAGATCGTTGTGTTCTACCTGT 24 60.083 291

chr4 23603327 GGAGAAATGAGAAGGATTGAGAGGA 25 59.87 TGTTGGTATACTAAAGCTCGTCGT 24 59.843 168

chr4 23613742 TTGTCCCCAAATCAAACTCACATG 24 59.961 GATAGCATAAGTGTTGTCCCAACA 24 59.059 281

chr4 23616054 TATAGAAGGCGAAAACTGAACCCA 24 60.02 TGCTACTCCCAATATTGCTTCTTG 24 59.116 264

chr4 23617148 TGGATCTTGATCAAACTTCAACGAG 25 59.59 CAACGAATTAGCTTGGATTGGTATC 25 58.57 257

chr4 23619358 TCTTCACTCTCACCAAACTCTAGC 24 60.022 AATTGAGGGGATGGCTACAAAGAT 24 60.079 105

chr4 23654949 TGTGATGACATGAGAGAGGAAGTC 24 59.841 AAAATTAGATGCTGGGTTGGGTTG 24 60.02 231

chr4 23661852 GGATTTATATATGTCGGTCTCTAGTGG 27 58.593 CGCACACAACCACATATTCCATAA 24 59.904 261

chr4 23663692 TGAGGAAAATGATGGAAGAAGGGA 24 59.708 AAACATAGGGAAACGAGAAATGCG 24 60.143 290

chr4 23666361 TGAGGAGAGAGAAGAAAGGAAAGC 24 60.021 GATAGATGGGAAGTGAAGGAAGGG 24 60.202 229

chr4 23711758 TAGAGTGCATTTTGGAGAAGGTGA 24 59.958 ACCCCTATAGCATTTTGGACATCA 24 59.832 241

chr4 23721299 GGACCCAACCTTTCTTTGATTTCT 24 59.411 AATTAGGAGTGGTGGTTGAGGTTA 24 59.4 203

chr4 23721841 TGCAATAGGACTACATGATCACCC 24 60.202 CATTTTGCTTACGTCATAATCCCTG 25 58.798 192

chr4 23727289 TATGGTAAATGCATCACGAAAGGC 24 59.964 CCATCTTTTCAATCCTTGCCACTT 24 60.021 247

chr4 23744565 ACGACAAGTATGAGAAGCAAGT 22 57.741 TGGTTGACTATTCCATTCGTGAGT 24 60.021 113

chr4 23745640 CAGACTATCAACAAAACCGTCACA 24 59.49 GCATGAAAGGGGAACGTGTAATTA 24 59.603 285

chr4 23745748 CAGACTATCAACAAAACCGTCACA 24 59.49 GCATGAAAGGGGAACGTGTAATTA 24 59.603 285

chr4 23749050 ATGTACACCCACACGAGTTTATCT 24 59.778 AGTTGAGTTGGATTGGGTGAGTAA 24 59.896 147

chr4 23753855 TCCCTGCACTTATTAACCCTTGAA 24 59.956 AGTGAATTTCTCTCTGGACGTTGA 24 59.961 290

chr4 23754252 TGAACAATGTGTGAGAGGAAAAGG 24 59.42 GTGATTGTGATGAGAGAGAGTGGT 24 60.082 167

chr4 23754358 ACCACTCTCTCTCATCACAATCAC 24 60.082 CTTGTTTGTCCCAAATAAGCAGGT 24 59.961 273

chr4 23758875 CTACATTAGTTGCCTTTCACCCAC 24 59.844 ATCCTTTTAATTAACCAGCCTGCC 24 59.594 269

chr4 23763544 GAACAAATGATCAGCGGGAGAATT 24 59.903 CCTCTTTCCATCCTCTTTACCACA 24 60.019 276

chr4 23765075 AACGACCACAGTAAACAACGAA 22 58.739 TTGTTGATCCCTAACCACATGA 22 57.351 273

chr4 23765992 CGGACAGTAGACATCAACGATTTG 24 59.968 GCATTACCAAAATCGCTTCAACTTC 25 59.939 219

chr4 23770622 TACCCTCAAACCCATCTACACTTG 24 60.02 ATGGATCTTCTTCTTCAACGTGGA 24 60.02 195

chr4 23770793 TCCACGTTGAAGAAGAAGATCCAT 24 60.02 GATGGTTCTAACATTGCCGGAAAT 24 59.902 265

chr4 23778432 GTGGTAATTTGCGTGCTGTATTTG 24 59.677 TGGTTGTCACACTAACATCTCATG 24 59.003 297

chr4 23779897 TGACTGAGTGTTATGAGTGATGTGT 25 59.989 AACGCATGCTATCTAGTGACATCT 24 59.961 287

chr4 23783903 AATGGATCGATCAGTTGTAGGTGT 24 59.838 CATGGTGGAGGCTATGCTCTATAT 24 59.592 281

chr4 23785319 TCCAAAAGTTCGTATTGCCATTCC 24 60.082 CGAGGAGAATGAAGGAATGAAGGA 24 60.142 284

chr4 23787702 TGTGATTTCAGGTCATGTCAAACG 24 60.024 GATTCCCAACACAAGAGAGAGAGA 24 59.778 297

chr4 23789350 TCTCCGCATTAAAACCTCTAACCT 24 59.776 AGCCCTCCTGAATGAAGTATGC 22 60.159 270

chr4 23820202 CCCATTGATCTTAAATACTCTCGCA 25 59.008 TCTTGAAGAGTGAGTTAGGTTTCGT 25 59.931 298

chr4 23930292 TGTCTGCTATCTTCCCTCATTGTT 24 59.774 AAACTTAGCTCAACTGATACCCGT 24 60.02 212

chr4 23932809 AATTCCCATTTTCCCCTTCGTTTC 24 60.021 CCCGACACATTTCCATCAAAATCA 24 60.082 255

chr4 23937498 GCCAAACTTCCTTCTCTTTAACCC 24 60.022 TTTCTCTATCACCGTCGATGTTGT 24 60.082 171

chr4 23940770 CTCTCGGTCATCTGTGTTAAAGGT 24 60.322 ATATCCGCCATGTATCTCTGTGAG 24 59.841 299

chr4 23942855 GAGCAGTACATAATACGTCTCGGT 24 60.024 ACATGGTAGAGAGATTGCTGAACT 24 59.531 193

chr4 23945419 AAATGGCGATTGTTGCAGTTATGG 24 60.915 ACTGAATCCTGTTCGACCTTAGAA 24 59.473 294

chr4 23978555 TGTGTATGTGTGACAGAGAAGAGA 24 59.173 TTCTTTCTTCTTTCCTTTCGCCAC 24 59.964 269

chr4 23985220 CAAAATTTGAAATCGACCGCACAG 24 60.144 CCTTGAAGTTAGCTCCACACATTG 24 60.083 289

chr4 23994215 AATGGTGTGGAATCTGATCATGGT 24 60.326 GTTAGATTGAAAATCTGTGGAGGAC 25 57.922 248

chr4 24000513 TAATTATGGCTCCAACTATCCCAG 24 57.873 GAGATCCGTGACCAAAATGAACTC 24 59.906 256

chr4 24011733 GAAGAGTAACAATTCGGTCCAAACA 25 59.762 TGGCCGTTGTAATCATAGTCTTAC 24 58.646 218

chr4 24111654 GACAATGCAAAGAATCGACACCTT 24 60.32 AAACTATTCTCCATTCGTGGGA 22 57.163 259

chr4 24112711 GGAGAAAATTACAACCGAACCCAC 24 60.321 AGTTAAGCACGAATGAGATGACCT 24 60.081 282

chr4 24115751 CGATTTCCCTTTAGTGGATGGTCA 24 60.625 GGCCCTGTCTACGTACAAGAAG 22 60.417 292

chr4 24116086 GGCATTGGGTTTATTGTGAGTGAA 24 60.022 TTCCCGCTAAAGTTACCTCATCAG 24 60.383 173

chr4 24116238 CTTCAAACAAACACACAACACCGA 24 60.849 AACGGCTAACTCATACCCAATCAT 24 60.141 190

chr4 24118526 CGAGGCTTTGTAGTTTTGGTTTCT 24 59.964 CACTCCTTTCAACCAGTTTAGTTACC 26 60.017 182

chr4 24119479 ACGTCTTGCTTTCTTAGTGACAAC 24 59.73 ATTGTTCGACGTTCATTGACAGTC 24 60.085 255

chr4 24123015 GAGATCGTACAACAAAGGTCATCA 24 58.831 CTTCACTATGCAAAATAGCCAAACG 25 59.487 243

chr4 24162906 AGTAGCTTGAATCCAATCACCACA 24 60.263 ATTTCCTCCAACTAAGACCTTCGG 24 60.324 206

chr4 24186277 TGGACGTAGGCAAAAGAAAATTCC 24 60.023 TCCGGAAACATCCACCTAAATTGA 24 60.264 141

chr4 24186696 CGAAAAGGTTTGGATGGAATGAGA 24 59.541 GGTCGGTTTCCCTTTATCTTCAAC 24 59.845 265

chr4 24208637 GCATGCATAGGAAAGACACTGATC 24 60.024 CCTCTCACCATTTTCTCTTCCAGA 24 60.02 209

chr4 24219818 TTTCTTAACTTCGTTGGGCTCTCT 24 60.202 GAACACCGTCCTAACAAAATGACA 24 59.726 249

chr4 24228511 TGAGACGAGTCAAGATGAAGAAAGA 25 59.758 GGAAGCCGTCATTAATTAAAAGGGA 25 59.641 269

chr4 24241952 CTCTCTACATTGTTCGGTACCACA 24 60.082 AAGTTGACCCGTTTGAGAGTACTT 24 60.142 145

chr4 24252348 GGGTTGCTATTTCCACAAAAGACA 24 59.961 GAGTCTTGAATTGGTTGTTGGGTT 24 59.901 285

chr4 24263070 CGACCTTCTCAACAATCAAACTAAG 25 58.406 TCCAAAATAGTTACCTTCACCCTGT 25 59.926 213

chr4 24265073 GTTCTTGTAGTTGAGTTCTCCTTAG 25 57.123 TCTTGTCATGTCGCTCTATCTTGT 24 59.841 218

chr4 24277688 ACCTTGAACGTCGTTATTGTGATC 24 59.612 TGAAGGGGTAAAATGGTGTAAGGA 24 59.646 111

chr4 24278152 CTATATCATTGCTTGCCATGGTGC 24 60.618 GCTTGTTGATCATATCGTCGGTTT 24 59.966 250

chr4 24278916 CAATTCGGCTTTAGAGTGTCGTAC 24 59.969 TGAGACCCAAGGAACCTACTTTAC 24 59.715 271

chr4 24279926 CGATGCTGTAGGAATAGGATGTGA 24 60.022 AAAGAAGAAGGCTGAAGGATTTGC 24 60.022 178

chr4 24280392 CATTTCATTTCGCCAAGCATTCTG 24 59.968 ATAGAGTCAAAGCCACCATGAAGT 24 60.019 204

chr4 24286660 GACCTTGAATATTGTAGATGACTCGTC 27 59.62 CATCTTGACAGTGGTAAACGATCG 24 59.968 273

chr4 24296450 TTTAAATGTTGTGGCACCCCTTAC 24 59.961 TGCCACAGATATGCATTTTCGAAG 24 60.202 258

chr4 24303417 ATCGAAAGGACAAATGGAGACGTA 24 60.082 CGAAATGAGAGCCCAAAAGAAAGT 24 60.023 230

chr4 24311113 GTCCCTTCTCATGTCTCTCTTTCT 24 59.534 CAACTTCAATGCCACGAGAAAGAT 24 60.083 109

chr4 24324103 AAAAGAAAGGGAGAAAGAGGGAGG 24 60.203 GAAGGGTTTAAGTAGGAAGCATGC 24 59.903 212

chr4 24326785 AGAGGTCAAGCTTTTCCATTTCATG 25 60.048 ACCTTCCCCTTCCTTCTTCATTTT 24 60.141 266

chr4 24327616 TCCTCATTTAATGGACAACGCAAC 24 60.083 CTGGGTAGATGTCTATGGTACTGC 24 60.022 249

chr4 24342879 TCATTCAAGTGGGGATTTCGTAGT 24 60.02 TTTGATCGATTTCTACGGTTGCTG 24 59.907 133

chr4 24343730 GTCAACAACCACTACCCGATATCT 24 60.142 AGCTGAGGTAAGGAAGAGAGTTTG 24 60.021 155

chr4 24347353 TGACGTTCGTAGTACCAAATAGCA 24 60.083 GAGAGAGCAAAAGTTGTGTGGAAG 24 60.261 233

chr4 24379039 ACATAAGTTTCTGGACTAGCAGATC 25 58.422 ATTTCTAGCATTTCTTCCAAGCCC 24 59.596 283

chr4 24404208 TTATGGCCCGGGTTTTCAAATAAC 24 60.082 ACTCTCTCTCTCTCTCTCTTTCTCT 25 59.276 184

chr4 24406836 CCGAGGAAGGAGAAGAGAAAAGAA 24 60.021 CTGGAGTAGTTTCAAGGTAGGAGA 24 58.983 202

chr4 24413627 GGAAAAGAGGGTTTAGAGGGAGTT 24 59.957 CCTCTAAAACTCTCTATTCCTTTCTCC 27 58.799 264

chr4 24415217 CTCAATATGGACGATGAAGGGCTA 24 60.021 AAGCTTAAGTCACACGTTCACATG 24 60.025 187

chr4 24416980 ACAGAAGTGAGTTTTGAGTAGGGT 24 59.591 GTCTCTCTTACTCCTTCTTTCATCA 25 57.836 181

chr4 24419470 CACGACAAACCCACATGAACAATA 24 60.024 CAAGATTAAACCCCATCATGCACA 24 59.84 114

chr4 24420638 CAATATGCTAGAGGTCGAGGGATT 24 59.778 ACATCGCATTTTCCAGATTGTCTC 24 59.905 236

chr4 24421024 CCTCTGTCTTTGTTCCTCACTACA 24 59.961 GAGAGTGTGGTGGTTTTGTGAAAT 24 59.902 159

chr4 24424315 AAAGAAGGGATCGGTATAAGCACT 24 59.591 ACGATGTAGGTGGTATTTGTCGAA 24 60.082 249

chr4 24426039 TCCCATTATTTCCTTTTCCCTCTCT 25 59.504 AAGTTCTTCCCTTGGGTTTAGCAA 24 60.633 292

chr4 24429060 AAGAGGATTGGATGATTGAACCCA 24 60.017 GGATGCCCTATAAAAGTGATGACA 24 58.623 299

chr4 24436018 ATGACGGGTATGTGGAAGACTTTT 24 60.263 CGGTAATGATGGCAAGTAACAACA 24 59.845 292

chr4 24439613 ATACTTTTAGACCCGGAAGCTTCG 24 60.68 TTCACCAACCTTCACAACAAACTC 24 60.082 299

chr4 24441201 CTTCCCCAAATAATCCTTCTCCCA 24 60.079 TTGCGGGAAGGAAAGAAATAAAGG 24 59.78 287

chr4 24442367 TCACTACAAAAGCCACCTCGATAA 24 60.021 TAACATCTTCTCTACAGCGCGTAA 24 59.905 175

chr4 24442560 CTCCTCCTTCATGTCCTGGATAAC 24 60.202 ACTGCTGGAAAACAATCTTGGATG 24 60.022 284

chr4 24457839 AACTCCCGATGCACATGTTAGGAA 24 62.385 CCATCGGTAAGTGGTTTAGTTTAGT 25 58.832 295

chr4 24458645 AATTAATTGTCCACCCTCTTCCGA 24 60.019 GGGAGTTTCTCGATGCAGGAAA 22 60.615 237

chr4 24459737 TCTAGAATGGGTGGTCGTGAAATT 24 60.02 GCCTGTTCTTTCAACCATTCTCTT 24 59.721 155

chr4 24466593 TATAACATCGGATTTGTGCCTGAG 24 58.944 CCCCTTTGCCGAGTCTTTAAATTC 24 60.381 286

chr4 24467155 GAAGCTGTAAATTGAGACGGAAGC 24 60.436 ACAGACCCGTTTGTACTCATTGTA 24 59.961 260

chr4 24472122 AATGGAGTTCAGAAGGGCCTAAAT 24 60.017 GATTGGACCCTTCACATCAGAGAT 24 60.142 197

chr4 24475472 AGCTGGGTACTTCTTTGACATCAT 24 60.019 AGAGAATTCCATCTTTACCCCTGA 24 58.96 279

chr4 24475961 GGTCATTGGTCCCATAGCTTTTAC 24 59.662 TCCTCCACTTCTTGTTAGTTTCTTC 25 58.711 267

chr4 24478097 GAAAACTTGGGATCTAATTCACTCC 25 57.91 AGACCAACCTTCAAAGACTCTTCA 24 59.836 258

chr4 24478418 TGAAGAGTCTTTGAAGGTTGGTCT 24 59.836 GTCCCTTCTGCTTTCCATTTCTTT 24 59.718 258

chr4 24478586 ACTCTATCCCACTGTCTCCAACTA 24 60.017 GTAAGAGGGAAATGTCAGGAGGTT 24 60.019 203

chr4 24480580 ACCGCTTGTTCTCGAATCATCATA 24 60.441 AACGCACCCACTACTAATCTTCTT 24 60.02 205

chr4 24499170 AAGTGTTAGTGGGAATGGAATCGA 24 60.02 TCTCTTTCCTCACTTTGCCTCTTT 24 60.141 281

chr4 24499456 TGGCGTTTGATCGTTTATAACCAG 24 59.906 ATTGGAAGAGAAGAAGAAGAGGGG 24 59.773 189

chr4 24501889 GCTTCACCCTCTCTTCATTAATGC 24 59.964 GTCTTTAAGAACATTCAAAGCGCG 24 59.682 255

chr4 24505675 TGTGTCATCTTTTCATGTTTGGGC 24 60.501 CCCTTCTTCTTCTACTGTGAGAGA 24 58.986 266

chr4 24507679 AAGCGAAAGTGAGTTTAGTTCAGC 24 60.025 TAATGTTTGTGAGTCTTGCCCTCT 24 60.202 291

chr4 24517347 ACCATAGTGTGAGAGTGTGTAAAGA 25 59.461 GAAACACACAGATTCACACACACA 24 60.143 132

chr4 24524018 TATAACTCCGTACACAGCACACAA 24 60.022 GACGACGACCATGCTCAAATATTT 24 59.966 203

chr4 24532247 GCCACATTACGCATTAATACAAGAC 25 59.093 GGAAAGTAGTTAAATGAAAGGAGGG 25 57.608 177

chr4 24563373 ACTGGATGGAAGAATCTTTGGGAA 24 59.955 AGAAGAATGCACAGTGAAATTGGG 24 60.022 186

chr4 24604087 AGAAAGGTTGTTTGAGGAGATGGA 24 59.895 CTAACACTTGTCCATGCGTTGTAG 24 60.143 288

chr4 24605318 TTGTACGTGTGGTCTCATTTCATG 24 59.549 GTGTGTTGTAGCTAAGGTTTGCAT 24 60.023 269

chr4 24691823 GCACACCCTCTTTATTACACACAC 24 60.083 AGGGTAAGGTTGGGAAATTTGGAT 24 60.203 278

chr4 24695499 CTGTTTACTCTCTCACCCACAAGA 24 59.961 TGGATAAACTATGTCATGCACTCG 24 58.95 243

chr4 24698031 TCAATGAACGCCTTAGAATTCTGC 24 59.905 TACCTACCTTTATCTGAAGCTGCA 24 59.285 238

chr4 24698756 TCCTAAACGAGTGGAAATTTTGTCG 25 60.05 ATTGGTTTTCGCCCTTCTCAAATG 24 60.561 247

chr4 24700264 GTTCGATTCTCTTCCTCCCTCTTT 24 60.081 AGCCCAATTTCAAAGTCTACAAGC 24 60.022 282

chr4 24700956 TGATCATACATTGGAGGATTGTGTC 25 58.942 CATTTGATGAGGTGGGCAATTGAA 24 60.323 290

chr4 24714299 TGCATACAGACTGGCAGCTATATT 24 59.959 AATCCCTGCTGTAAAGATGGAAGT 24 60.018 230

chr4 24716462 TGCTGGATTGTCTGGTTTCTAAGT 24 60.202 TCCATGTTTTACCTCCAGTCCAAT 24 59.956 227

chr4 24717407 TGTTTCTTCCCTGCACACATAAAC 24 59.963 TGCTACACAATCTCTCATGCCTAA 24 59.838 119

chr4 24718690 CTTCACCAGCAGCAATACGTACAG 24 61.957 CAAAGAGAACATCGAAGGTGCTAC 24 59.908 243

chr4 24723511 GAATTCCAAAATGAAGAGGCCCTC 24 60.142 AAATCAATGGAATACTGGTGGGGA 24 60.017 238

chr4 24733240 AAGTGTTGAAAAGAGGTGAGGAGT 24 60.08 TGGGTTCTGATTCCACAAACAATG 24 59.961 267

chr4 24747235 TTTCCTTTCACCTCTCACCATCTT 24 59.895 GAAAAGAGAATTTGGAGGTGGTGG 24 60.022 134

chr4 24760378 AGTCATTAGCAGGATCCAAGTTGA 24 59.774 GTACCAACTTTTCTTCAGTGTGCA 24 59.904 174

chr4 24787183 AACAACGTTGGTAGTACATGAACC 24 59.487 AAATGCGTTGCCATCTTGTACTAC 24 60.143 235

chr4 24799341 CCTCCTTTAGCTCTCCCTCAAAAT 24 60.08 CACTTTCTCTCCTTCCCTTCTTCA 24 59.959 242

chr4 24801581 TTGTTGCACTCCTATCTCTACCAC 24 60.082 GGTGTGGTTTTATGGTTTGGACTT 24 59.9 216

chr4 24801957 AAAGAAAGAACATGCGATCAAGGG 24 60.083 CAGTGAACGTAGGCTTCTTCTTTG 24 60.084 231

chr4 24889760 AATCACCAATCACTCCCAAAACAC 24 59.961 GTTTCCTGTGATGTGGTTGCTTAA 24 59.963 174

chr4 25004964 TCTCGTAAACATCCACTTCCAACT 24 59.96 GCTGAAATGTTGGAATCTCGGAAA 24 60.083 204

chr4 25007407 CATTGCAGAGTGGGTTTTGTTTTG 24 59.966 TACACATAACACAACAACCAAGCG 24 60.261 224

chr4 25007659 CGCTTGGTTGTTGTGTTATGTGTA 24 60.261 TTGCGAGTGGGTGTAGTTAAACTA 24 59.961 287

chr4 25010220 AGTTTCAAAGCACTAACACGAGAG 24 59.492 CACAATAGCACATACATGAACACGA 25 59.937 271

chr4 25011988 ACAACCTGTAATTTCTCTGCTTGC 24 60.023 CAGCTCATTTTGGTTAGATGGGTG 24 60.142 299

chr4 25012245 AAACACTCCCACCTTTCTCTTTCT 24 60.08 CAACAAGAGCCATGAAAGAAACCT 24 59.962 167

chr4 25032228 AGGGGATGGAAGATCACATTTCAA 24 60.017 TGTTGCTTGTGTAGTCTCAGATGA 24 59.961 245

chr4 25033753 AATCTGATTGTATGATTGTCGCCG 24 59.788 CAGGAGCAATTGACAAAAGGAACA 24 60.202 227

chr4 25118056 GGATACACACACTTCCTATTAACTCC 26 59.015 GGACTATGGCATGTTACATGTTACA 25 59.18 263

chr4 25126604 GCCTTGACGTAACCCAAACATAAA 24 60.023 AACTGAAGCTTCTCTCCCATGATT 24 60.019 209

chr4 25130035 AGTTCCATCAATTCCATTTGTGTCC 25 60.047 CAATGAGTGATCCGTTACAAGCTC 24 59.967 278

chr4 25134177 CCTGGCCCTATCATACCATTATCC 24 60.141 TAGGCGCTAAAAGGAAGTTCTACT 24 59.535 232

chr4 25140337 TACCCAACTTCTCCTGTAAAAGCA 24 59.897 AGAATCAACCACTGCCTCTAAAGT 24 59.958 266

chr4 25211651 AAAGGATATGGGATGGAGTGTGAC 24 60.141 GAAAATGTGCCTTGTTGTTTGGAG 24 59.729 295

chr4 25214325 CTTCTCTTGTGGCAGTTCTCTTTG 24 60.024 AATCGACCAGCCCTTCAGAAATAT 24 60.141 234

chr4 25215675 CTCTGCCTCAATGTTCGGTTAATC 24 59.965 TCAATGCAGGATAAGTGGGAAGAA 24 60.019 288

chr4 25216129 CTACTCTTTCTTGTCCCCTTTCCA 24 59.958 GCTTCCACTTGTTTAGGTTCGTAG 24 59.848 284

chr4 25273043 ACACAACACTCCCAACATCTTACA 24 60.385 TGTCTCTCATTCTTCCTTCTCCTT 24 58.91 275

chr4 25304029 TGAGAAGGATCAGACACCCAATTT 24 59.957 AGACAAATTGTTCCTGTTTCCACC 24 59.901 257

chr4 25315940 TGCCCCTAAGAACCGAATTAACTT 24 60.264 AGGGTTGACAGCACTTTCTTTT 22 58.635 167

chr4 25324384 CACCTCATCCTTGGCTTAATCTCT 24 60.142 TACCTCTCTAAATCTCGCATGCTC 24 60.023 297

chr4 25333881 GTCTTTTCCAATCTCCAACCACAC 24 60.262 TAATCCCACAGAAATCCCCAACTT 24 59.955 256

chr4 25337334 TAGGCACACACACACACACATATA 24 60.021 GCAGAATTCGATTGGGCATTAGTT 24 60.202 252

chr4 25344664 ATTGGCGGAGAATATGCATCTTTG 24 60.023 CGAGAGAAGAGAGGTTTTAGAGGG 24 59.903 218

chr4 25356385 GTTTGAACGGAAAGCAGTGTTATG 24 59.325 ACCAACTAACTACAACGCTCTTCA 24 60.202 188

chr4 25369546 TGGGGATCACTTACAGAAAAGAACT 25 59.927 ACCAACCACATACGACCATTATTG 24 59.362 295

chr4 25377441 AACCCACCTAACCTTACAACACTT 24 60.08 CATGACATGAAGATTTGCTGCTCT 24 59.903 271

chr4 25386986 GGAGGAGTTTGTGGGAAGAGATAG 24 60.142 ACTCAGAACCATGTCCTTTCTTCT 24 59.651 261

chr4 25389430 GCATAAATTTTGGTCCACATGCGT 24 61.15 CTTAAAGCTTGCGGACTATAACTCT 25 58.957 291

chr4 25390926 GTCAGTTGGAGTTGGTTTCGTAAA 24 59.667 AAACATGTGGTGATCATCGATTGG 24 59.903 185

chr4 25393448 GAATCAACACATTCCTGGCTAGT 23 58.735 AGGTGAAGACTCGTGAAAATGTTC 24 59.488 296

chr4 25490421 ATCTTCAACGTGCTCTCTCAAGAT 24 60.082 GAGGAGAAGAGTGTCAAAGCAAAG 24 59.787 291

chr4 25491760 AGGGTAGAAAAGGAGAGAGGAAGA 24 59.954 GAACCACCAAATCAAGATCTCTGC 24 60.143 277

chr4 25543397 GAGAAGGATTAGTCAGGATCGGTT 24 59.657 TCAAACTGACCTCGACATCAATGA 24 60.262 117

chr4 25569164 CCAACGGTTAGGATTAAAACATGAC 25 58.681 GAGTCCGATCACAAATTGTCATATC 25 58.118 160

chr4 25570577 CCGTAGACAACCTTGCTTTGAAAA 24 60.202 TAGTGCTTGTAATTGGAACCTTGC 24 59.782 273

chr4 25610191 TGAGTAACTACCAAACTAACGTCGT 25 59.991 ATTATGCCATTTCCCCTTTGCTTC 24 60.142 295

chr4 25638177 ATTCGGCCTTCCCAAAACAATAAG 24 60.082 TCTCTCTCTCTGTATCTCTTCGCA 24 60.142 296

chr4 25654003 TGTGAGGTGGAAGAGAATTGTTGA 24 60.142 GAAGGAAGAGAGAACAGGGAGAAG 24 60.082 227

chr4 25659614 GTTTGCGAAAGTTAAGATCCGGAA 24 60.084 ATAACTCACCCCAACCAAAAGT 22 57.807 159

chr4 25664386 CGGAACCATCGGATATCTTACCTT 24 60.021 AGGTAGAAGTTTGAGAGCCCAATT 24 59.957 201

chr4 25673516 GCTTCTGTTTCACTCTGTTCACTG 24 60.261 GGAAGTGAGTAATACGAAACCGGA 24 60.381 260

chr4 25685643 AAAGTTTTGTTGGGATCAGATGGC 24 60.263 AGGGATAGCATAGCAAGAAAGAGT 24 59.342 222

chr4 25703291 AGGTAATCTATTGTCACTTGGGCT 24 59.525 TTTGCGAAATCATCAGTTGTCTCC 24 60.083 258

chr4 25755820 GGTGTCACTTTTAGGGCTACTACT 24 59.778 CAATGAACTTATCTCGTGAGTGGC 24 59.967 262

chr4 25773624 TGGTTGGAGAGACTTTGTTTGGTA 24 60.08 TGCTGAAGTAGTACCAACGGATTT 24 60.263 171

chr4 25774197 TCCTCCATCACAAAAGAAAGCAAC 24 59.962 CCCTCTTCTTCTTTCGTCCAAGTA 24 60.021 228

chr4 25777473 CAACCAATGCATACCTCCACTTTT 24 60.021 AGCCTTACGAGTGGTTGATATAAGA 25 59.403 244

chr4 25788854 GCTGTTCTGAAATTGGAGATGGAG 24 59.904 ATGGGGATGCACGTCAAATTTAAG 24 60.142 221

chr4 25791357 GTGGCATTTACGAGTTTGTACACT 24 59.787 TAGAGGCTCTACTTGAATGGGAGT 24 60.326 279

chr4 25792589 TCGTAAAAGTCTCCAACACCATTG 24 59.486 CACACTAAGAAGAACTCAAAAGCAG 25 58.574 261

chr4 25798408 CTTCTTTTATGATGGAGGTCAGTGT 25 58.824 TCGGCTAGCTAGAAATCTAAAGTCA 25 59.407 275

chr4 25799104 AAGGTGTACGTGTAGGCATTAAGT 24 60.021 ACTCAACTACCAACCATGATCCTC 24 60.081 262

chr4 25799543 TATGGTCGGTTCGTTCATAGATCG 24 60.32 AAGTGCAGCATTACGATAATGACG 24 60.026 220

chr4 25801444 TCATGACTTCAATGACCGATCTC 23 58.313 ATTCGAATTCCAACCACCACTTTC 24 60.022 289

chr4 25805263 TCTAACATTCTTCGCTTCCTCCAA 24 60.021 CCAATGTACTTAGCGTTAGAGTGG 24 59.196 278

chr4 25815547 TTGGGGAAGAAGCATGGAGTTTAT 24 60.264 CAGTGTGTTGTCCTCAGATTGTTA 24 58.944 258

chr4 25828888 GATAGATGGAGTGAGTGTTGTACA 24 57.791 ATATGACATGTGATAACCGAGCCA 24 59.96 210

chr4 25834147 AAATGGTGCATCGATTGTGACTTG 24 60.617 GCATAGACAGTGAGTTCTCATCCT 24 59.901 245

chr4 25836019 AGAGAGAAAAGATGAAACGGGGAA 24 59.958 GTGAGAGGTTCCATTTTCAGCAAA 24 59.962 116

chr4 25836932 CGAACCGACTTAAATCCTAAAGCA 24 59.372 GAAATCACTTCTTATGCGGTTCGG 24 60.494 277

chr4 25838852 GGTTTGAGATGAAAGGAAAGATGCA 25 60.048 CACCACAACCAAAAGTCCCAAAAT 24 60.625 146

chr4 25839406 TTTGGTTGTTTCTTAGGGGTTTGG 24 59.837 CTCTTGAAGTATAGTGGTAGATGCA 25 57.956 260

chr4 25842257 GTGAAAATAGCTCGCAATACAAGGA 25 59.935 GCAGGATAGGCTCGTCTAAGTATT 24 59.781 212

chr4 25843832 AGGTTTCTAATTTCCCCACACCAT 24 60.203 ACTCATCAATCCTTACCAAATCGC 24 59.424 234

chr4 25848497 TCCATTTATGTGCACCTCAACCTA 24 60.019 AGCTCCTGAACACTAATCTCATCC 24 59.899 222

chr4 25874057 TTGATGGTTGAATGTGATGTACGG 24 59.605 CCACCACCACAATGATCATTCTTT 24 59.779 229

chr4 25877745 TCACATGGACATAGCAACTCTTGT 24 60.263 GATGTAGCAGTGAGGGAGATTCAA 24 60.142 244

chr4 25883222 TGGTTGCAAGAATGATAGAAATGGG 25 59.872 AGACAATTACAATAAAAGGGGTCCC 25 59.044 190

chr4 25978046 GAGTCTTCCCTTATTGTTCTCCCA 24 59.775 TCGTTCTTCATGCCAAATAACAGC 24 60.379 161

chr4 25978502 TCTTCAAAATGGTGCACTGACAAG 24 60.202 ATATCCAACAAATTCAACCTCGGC 24 59.902 277

chr4 25978862 GCCGAGGTTGAATTTGTTGGATAT 24 59.902 TCAAGAGGGATGGAGAAGGAAATG 24 60.08 298

chr4 25989886 CCCAATTGAAAAGAAGGTGCAAGA 24 60.202 CCTTATAGACCCCTACTGACAAGG 24 59.411 290

chr4 26003459 AAAGTGTGTGTGAGAGTATAGGCT 24 59.471 TGATACGAACACGATCACATACCA 24 59.903 265

chr4 26003559 AAAGTGTGTGTGAGAGTATAGGCT 24 59.471 TGATACGAACACGATCACATACCA 24 59.903 265

chr4 26003927 CAAGAGGAAAACGGAATCTTTGGG 24 60.321 CGCTTAAGAGTCGTATCCTATCCA 24 59.545 177

chr4 26006992 AAGAAGAAGGAGTAGGAGGAGGAA 24 59.954 ATGACGATAAAGCCTACATCCCTC 24 60.021 272

chr4 26008305 CTGCTATTGATTGTTTGCGTTGGT 24 60.852 CGGCAGGATTTTCAACATTCAAAC 24 59.85 119

chr4 26010253 CGAGTGTATGCATGAGTTGAGAAG 24 59.733 AGCAAGGTTTTCAAAAGCTACC 22 57.678 250

chr4 26014826 CTTCCGTACTTTCGTTCAAACTGT 24 59.731 CAATAGCGAGTTGGATGAATTTTGC 25 59.769 296

chr4 26016994 TGATGATAGCCTCTCACTTGAAGA 24 59.041 AACTCCCTCTTACTTGTTACGACG 24 60.321 180

chr4 26018886 TCGCTTTTCCTTATATCATCCCGT 24 59.96 TGAAGAAGGAAGAAGAAGAGCGAA 24 59.961 123

chr4 26029220 ATTAACCTTTGCGCTTACTTTCCC 24 60.082 GAATGAGAAGCGTGTTTGGTAAGA 24 59.549 249

chr4 26030057 TGTAAACCACTATTAGGCACCGAT 24 59.837 TCTAGCTTTTCAAGACGTCCTGTT 24 60.202 272

chr4 26031888 CTCCATTGAAAAGCGTGACAGATT 24 60.083 GGGTCTAGTCACATTCTTTCGCTA 24 60.142 240

chr4 26032124 TAGCGAAAGAATGTGACTAGACCC 24 60.142 CCCCACGAGGTAGTTAAAGTCTAT 24 59.352 200

chr4 26041872 GGAGCATTAAACCAAGAACAGAAGT 25 59.758 TGTGGATTGGGAACTTCTTCTTCA 24 60.141 94

chr4 26042610 CTCACGTTTGGATCACTTGAAGAC 24 60.084 CACCAATCCTCAAGAACTGCAAAG 24 60.558 239

chr4 26044303 AATTGACGTTCTTGATTGACTGGC 24 60.32 TTTTCTGGTTCATGAGAGGATGGA 24 59.711 226

chr4 26044484 TCCATCCTCTCATGAACCAGAAAA 24 59.711 TTCCCATCTGGCTTTGTCTTCATA 24 60.019 169

chr4 26045198 CCATAAGGGCATGAGTTAAAACCA 24 59.291 TCTAGCATGAACAGAGAGCCAAG 23 60.119 234

chr4 26060072 GGGCATCTTTTGGAGCGATTTTAA 24 60.382 TCCAGCCCTGATCGTAACAAATAT 24 59.897 271

chr4 26061182 AAGAGATCCGATTGTTAGTTGCTG 24 59.127 ACCGATCTTAGTCTCTCAATGGAA 24 59.043 288

chr4 26068039 ACCATAACGAAGAAGAGACAACGA 24 60.023 TGTTGCGGAGTATATGCCTAATTG 24 59.245 166

chr4 26069931 CAAACATCAAAGAGCTATCACAGGA 25 59.355 AACGTCTACTTTAATGGGGTGGTT 24 60.202 204

chr4 26074395 CTTGTTCGGAAAAGAGATGGATCG 24 59.967 ATTGGGATGGAGGGAACAAAATTG 24 59.774 279

chr4 26141275 TGTTCCACTTTGAAAACTCGAGAC 24 59.668 CGAACGACTTAATTTATGCGCATG 24 59.627 125

chr4 26141824 TGGAATGGAATCAGAGAAAGGTGT 24 59.957 GGACATGATAATGCTGGATTGTGG 24 60.023 215

chr4 26145359 GACGAATACCCGAGAGTCAATCAT 24 60.262 AACAAATCCGAACCATGAAGTG 22 57.496 291

chr4 26148679 ATGCTCTGACATCAACTTCTCTGT 24 60.02 CTTTGCTTTACGTTCTCATCCGAA 24 59.848 145

chr4 26160607 ACCCATGTTCCATATTTCCTGTGA 24 60.018 TCAGCTTTCAGTTGTTCAGGGATA 24 59.958 142

chr4 26173058 ACTCCACCCATAATCCACAATCAT 24 59.831 TCTAAACCAAAATCCCCTCTGACT 24 59.398 262

chr4 26376706 GTTTGCACGAAGAGAGTTTTGGTA 24 59.965 CATAAAGCAGGTCCCAATGTGATC 24 59.963 99

chr4 26442974 GGATAGCCAATGAAGTGTTTGAGG 24 59.903 AGATACTCTCTCCCACCTCTTCC 23 60.117 237

chr4 26521197 AGTGTCTTCCCATAGGTTCGTTTT 24 60.202 TGGTCCACTAGATGTTGAGTTTGA 24 59.653 257

chr4 26558867 TCGTGGTAAGTTTGATGATGGACA 24 60.263 TGTCGAAAATGTTGTTCAGTGGAG 24 59.965 247

chr3 293894 TGGAAGATTTTGGCACTACTGGTA 24 59.958 GTGTGTGTGTATGTGTGTTTCGAA 24 60.202 211

chr3 380153 CTCATCGGTTTCACAAGGAGGATA 24 60.142 TTCGGTATGAATCCAGCTTCTTCT 24 59.838 244

chr3 405981 GCCCTCTTTGTTCTTATCCCAAAC 24 60.082 TGAGAGCAAACTTAAAGCCTTG 22 57.164 266

chr3 407377 GTACACCAAAACCCCTTTCCTTTC 24 60.202 GAAGAAGAAGAGAGTGTGGGGAAG 24 60.563 145

chr3 409586 ACTTGGAGTGGAGAGATTTGAACA 24 59.897 TCCTCTCCTTGTTCTTAGTGTTGG 24 59.96 262

chr3 418174 TATCACCAACACTTTCCCTCACTT 24 59.896 GTGTGCTCTTTGTTTCTCCTTTCA 24 59.904 147

chr3 418589 CCCCTGGAAATAAAATTAGTACACCAC 27 60.148 TAGAAGAATTGCGACACTCATTGC 24 59.907 240

chr3 423719 CAAACTTTATACGTGGACTCTGAAC 25 58.182 TGGCGAACTCTCCATAATAAACGT 24 60.382 253

chr3 428277 AATCGCTAATCTTCGTCCGTTTTC 24 59.967 CTACGTCGTGGAAGGCTTTAAATA 24 58.896 159

chr3 433222 AATATCCGCGGTGTAATAGAAGCT 24 60.021 TTTGCTTCATCATTTCCACCTCTC 24 59.54 280

chr3 530324 AACTCTATGCAACAAGAACACAGC 24 60.024 GACATCCTTTCCCCTATGAACAGT 24 60.08 230

chr3 538445 TCCTATACCACCTCACTTTTCAGC 24 60.081 GCGGCAATCTGTCTCCAAATTTAT 24 60.202 264

chr3 1691064 TAAGATGGGGAAGAGAGGACTGAT 24 60.078 AAAGGGAATCAAAGACAAGAGGGA 24 59.894 141

chr3 2696676 CCGAAAGCAAGTGAGTAAAATCAAG 25 59.147 CTCTTTCCTCCATACACACTCACA 24 60.021 235

chr3 2711504 GTGAAGTTTTGTGGCTTTGGAGAA 24 60.382 GAACCCCAACAACTAACGACAATT 24 59.963 299

chr3 2726377 CGACACTAAAGGCGTTGTAATGAG 24 60.202 AGAGATTTGGAGGTGGAGATCAAA 24 59.462 225

chr3 2732889 TCGAACAGATTAGGGTTTAGCACA 24 60.021 TAAACCCATTGATTACTAGCCGCT 24 60.142 133

chr3 2733865 TGGGGTATGTCATTGTTATCTGGG 24 60.141 TGACAGTAAGCTAGCCATGTGAAT 24 60.081 281

chr3 2736706 GATTCATTATCTAATTGGTGGAGGG 25 57.306 TGAAGATCTAGAATGGCAGAGACT 24 58.793 270

chr3 2775501 TTCATGGTGAGGACTGAAGAACTT 24 59.897 GGTCTCTCTCTCTCTCTCTCTCTC 24 59.961 225

chr3 2792195 CAACTTCCCTTTCACAATCCATCC 24 60.082 TGAACAGAAGGGAAGAAGAAGAAGA 25 59.633 286

chr3 2795290 GCAGCATTGGTCCATTTATCACAT 24 60.202 CATTGGCAATAGATGTTGTGTGC 23 59.139 213

chr3 2795916 GGGTAGCAACTGACAACACATTTA 24 59.483 CATTTCTCGCGCTTCTTCCAAATT 24 60.908 257

chr3 2801334 GCTGGGATGGATAGAGATGTAAGG 24 60.081 TACTAACATTGGGAGGGAATTGCA 24 60.018 262

chr3 2801540 TCCCTCCCAATGTTAGTATGCAAA 24 60.018 CCAAAGTTGAAACCAATGCAAGAG 24 59.491 101

chr3 2804283 TCAGCTGCCATCCAATTTAATTCC 24 59.9 GTGTTCCACCATTCAAACTATACTCC 26 59.906 194

chr3 2813933 GCCCTACACTTTAAACATCATAACC 25 58.208 TCCCATATAAATCTGCTTTGCTGC 24 59.721 248

chr3 2831564 GTTGCCTAGAAAAGAATGGTCGAC 24 60.143 GTTCTGTTCTGGGCTTCTTTGTTA 24 59.421 165

chr3 2840057 AATTTGACCCTCTAACTCAGCAGA 24 59.713 CGAACAACAAATCTTAGCAGTCCA 24 59.787 299

chr3 2840363 TGGACTGCTAAGATTTGTTGTTCG 24 59.787 GTTCTGTTCCATGTGATTCTTGCA 24 60.023 128

chr3 2840552 ACACTACTAACGATGGCTCTTTGA 24 59.78 TCTTTCCATCGTACCATTCCTCTC 24 59.9 126

chr3 2841614 GGAGACGAAGGGAAAAGCAAAATT 24 60.262 TCCGAGCTGTCCCTTAAGTTTTAA 24 59.959 194

chr3 3068300 GGATTTTGCAGAGTACCTTAACCA 24 58.992 CAGTTTTGTTGGGTCATGGATTGG 24 60.8 273

chr3 3184065 TGCCTTAAATCTTTACCGACCTCT 24 59.776 AGAGCTCCATTCTTCTTCTCGTTT 24 60.021 275

chr3 3329388 TCCCCTCAACTCAAATCCATGAAT 24 60.017 GTTCTAGGGTTTCGTTTCTGTGAA 24 59.187 254

chr3 3420883 GATCGATGAATTCACAGCCAATGA 24 59.726 CCCATTGAAGTATCCTACCCTGTT 24 59.834 256

chr3 3973756 CTTCGCCATCTTCATCTTCGTTTT 24 60.143 ATTTGGAGATGGAAGGATGTGACG 24 60.925 206

chr3 3989673 GGGGTATTGGCAAGAAACAACTTT 24 60.202 GATTGTTGATCTGTTTCTTGCCGT 24 60.32 296

chr3 4093810 ATTTCATAGTGGTCTGTTTGGCAC 24 59.782 TACCAAACTTTGTATCGCCCAATG 24 59.843 233

chr3 4100887 TGCTTTGGATTTTGGTGGGATTAG 24 59.777 GTAGTGCTCGGCTTTTGATTTGTA 24 59.847 248

chr3 4104332 CGGTAAGTCACTAAGGATCATTGC 24 59.492 CTTGACCAAAATCCACCAGAAACC 24 60.501 268

chr3 4108709 TTCACTTTCTTTCTAGCCTAGCGT 24 60.021 TTGTGAGTTTCGCCTTTTCAAG 22 57.972 235

chr3 4150602 TACATCAAATTTGCCTCACCCCTA 24 60.018 TCATTTCTTGCAGGGGATGAATC 23 59.044 254

chr3 4151573 CCACGAAAATCAACAAGCTGAGAT 24 60.083 CTCTCAAGCTTTTCTAATTTGTGCC 25 59.139 272

chr3 4163270 GCAAATGTTTAGCTGATCCTCTTGT 25 60.106 CGACCCCTCTAATTTCGAGTAACA 24 60.142 154

chr3 4166956 GTAAGACATGTCAACTAACGCTCA 24 59.078 CACACGGATAGTTAGTTTGAGTCC 24 59.137 235

chr3 4167565 TACGTTGGGTAGAGTAGTGAAGCT 24 60.809 AGTTAAGCATTGATCCAACGGT 22 58.314 285

chr3 4169263 AGGCCTATGAGTTATGGATATGCT 24 58.964 CCCCTAAAATGTTGGTGGAATGAT 24 59.284 273

chr3 4169377 AGGCCTATGAGTTATGGATATGCT 24 58.964 CCCCTAAAATGTTGGTGGAATGAT 24 59.284 273

chr3 4170200 AATTCCAGCCATTCAAAGTGACG 23 60.057 GCATAATCAATCCTTATCGCGTGT 24 59.848 300

chr3 4171935 TGTAAGCAGATCTAAAACCAGCTG 24 59.062 CACATTGAGAGGCCATTTGACAAT 24 60.082 262

chr3 4174119 GCTCATTTTCTCCTACTCGCTCTA 24 59.963 TTCCTATGCTTGAGATGAGACTGG 24 59.899 168

chr3 4177496 TTCTTTCTTTGGGTCTTTCATGGC 24 59.961 ACTCCCATGTTCTTCTCCTTCTTC 24 60.02 236

chr3 4180188 TTCTCAATGTGGGATTCTCAGCAT 24 60.325 GCCTCGAGACTTTAGTTTATTGCA 24 59.37 241

chr3 4182083 CGGTGAGTCTAGAGGATAGTAGGT 24 59.959 AATCCCTTTTCCCTTGAGTTCTCC 24 60.509 251

chr3 4183628 AATTTACTCTCGCCTTCACTTGTG 24 59.548 TCTTTAGAGCAGGAGATTGTAGACT 25 58.809 294

chr3 4184169 CCTTTTACAGTGCTAGGAGTGACT 24 60.021 TCGTTAACCCAAGCTAGTCTCTTT 24 59.717 275

chr3 4185382 ATTTGAGATAACTACGGAACCACC 24 58.574 CTTTACCCCATTCCTTTTGACCAC 24 60.021 249

chr3 4185528 AGCATGTCAATATTCCTGCCTAAG 24 58.931 TTGTCGGTTGGATTTGGATGTAAC 24 59.783 259

chr3 4187526 GATGAGTTTGTGTTTGGTTTGGGA 24 60.142 GTCTAGTACAAGCACACCTAAACT 24 58.219 209

chr3 4206158 TGACTTTCCATGACTCTCGATACT 24 59.05 TCTTTTCTAGGAGGCTTCTGGTTT 24 59.649 257

chr3 4224119 ACTGCCCTACTTGAGTTTATCCTC 24 59.837 GAAGAGTGAAAGTGATGAGACCCT 24 60.021 214

chr3 4236836 GTGTACCTTTCTAGCCGTTGAATG 24 59.907 GAATTTGGACGTGCAATAGTTGGA 24 60.083 294

chr3 4238295 CGACTGCAGGGAAAATTGTAAACA 24 60.261 ATCACCACTGACAAAACTTTCGAC 24 59.965 188

chr3 4238550 GCCGATGATAGTCAATGATGGTTG 24 60.084 TGATCACTAGCAAACCTCCAATCA 24 60.019 194

chr3 4251092 TTAACCACGGAAGAATAGCTGGAA 24 60.02 AGCATAGGTTGAAAGGTTGAAAGC 24 60.022 288

chr3 4251261 GCACCATAATCAACATACCTGCTC 24 60.024 GGGTCTAAATCCTGAGGTAGCTTC 24 60.202 271

chr3 4253498 GGAGGTTGAGGCAGATGAAGATAA 24 60.142 ACCGATCCAACAATGCTAAAACTG 24 60.083 251

chr3 4254408 CAAACGAATGATTGGGGAGTTCAT 24 59.842 CTGGTAGCGTTGATTAAGTCTTGC 24 60.202 132

chr3 4278947 GATGTCAGGTTCAAACACTCATCC 24 59.845 GTACTAAGTGTTGTTACCAAGTGTG 25 58.059 293

chr3 4309269 CCAATTTGAGGGGAAAATGAACGA 24 60.022 TTTAACCTGGGGCTAAAACGACTA 24 59.959 299

chr3 4451833 ACTGACTACCATCTGCATCTAGTT 24 59.041 TTTCCATGTCTCAGTGAGTTGTCA 24 60.142 238

chr3 4579335 TCCCAAATAACCGTACCTGCTAAA 24 60.02 GTGGGGTTGAAGAAGACAAAGAAG 24 59.963 199

chr3 4703868 AGAATGTCTCCCAACTCAATCCAA 24 59.957 TGACATTAAAGAGACCAACAACGTC 25 59.764 213

chr3 4864246 TTTGGGGTTTTAAGGTGTGTGAAC 24 60.082 CATTGAGGCATTGAGGTAGGGG 22 60.751 153

chr3 4887370 CTCATCTTCAATGTCATCTTCTCTTCC 27 59.775 ATGCTAGCAAGAAACAAGGAAACC 24 60.022 164

chr3 4889564 TGTAGCTTGTTTGAGTGGTTTC 22 57.107 TCATATGGGTTGATTGCTTTGC 22 57.61 289

chr3 4894222 AAGGTCTTAGCACTTGGATTGGAT 24 60.018 TCTTTATGAGCTAGGAAGGGACC 23 59.032 272

chr3 4897264 ACTATAGGGGAGGCATAGCATAGT 24 60.016 GACGAATGATTGTGCGATACTTGT 24 59.967 265

chr3 4897399 ACAAGTATCGCACAATCATTCGTC 24 59.967 TGAAAGGCATGCTAGACAACATTG 24 60.083 180

chr3 4924918 CTTTGAGCTAGTTTGGACGACTGT 24 61.034 ACACCTCGTTCTAAGTTTTCTCCT 24 59.656 272

chr3 4935224 AGTTGAAAAGATTTCCACCCCT 22 57.801 CCATTGCCACTTGTCCTTTTAACA 24 60.202 117

chr3 5313098 CATCTCTGTTTTCGTCCCTCTCTG 24 60.913 GCGTACCAAACCCAATCAAATACT 24 59.843 163

chr3 5541186 TGTTGCAGTTGTCTACCATTTG 22 57.433 CCAATATTTCTAATTCCATCGTCGG 25 58.168 238

chr3 5555449 TTATCCACCAATTGTTGCAAGACC 24 60.022 GCGTGCTTAGGTTGGAATATTTCT 24 59.664 193

chr3 5556468 ACATTTACTTTCTCGCCTCACTCT 24 60.021 GTGGGCTGAGACATTTTGAAAGAA 24 59.962 229

chr3 5589144 CCTGCTAGGTGTCTGTTAACTTCT 24 60.021 TGGGTGTGTTTAAGAATGTGTGTG 24 59.903 230

chr3 5597863 TGTCGATAGCCAAGCAGATATTGA 24 59.961 TAAGGCTTCATGATAAGGTCGAGG 24 59.961 297

chr3 5599199 TGGAATAGCAAAGGAAATCTCACTC 25 59.117 TGGAGGGCTATCAAATGGTAATCA 24 59.585 231

chr3 5743262 TCCTTCTCCACAATGATGCACTTA 24 60.019 CCTTTAGACCCGAGTACCATAGTC 24 59.722 190

chr3 5743480 TTCATCCACAAGTGCTTAGAGACC 24 60.564 GGGCTTTTATTCCTTCAAGTAGCA 24 59.295 294

chr3 5749747 AATCTATCGTGGTCTGTCGTTGAT 24 59.902 GAATACACAACCCTCAAACGTCAG 24 60.084 195

chr3 5751750 CTTTGAGTTGTTCGTGCCTTGTTT 24 60.91 GGACAACACTTATCAAAACATGCC 24 59.077 281

chr3 5753126 TTGAAGTTGGAATATAGCGGTGGA 24 60.081 GTGTTGTCTTCACCCAATTGCTAA 24 59.963 252

chr3 5754540 TTTGCTCTTTCCATACACAAACCC 24 59.961 ACTCAACTCATCCACATCACAACA 24 60.445 251

chr3 5754719 TGTTGTGATGTGGATGAGTTGAGT 24 60.445 ATAGAAATTTGACCGTTTGTGGCC 24 60.322 244

chr3 5755085 GTTGTTGATTGCATGTACGTTGTG 24 59.854 GACAGCTTATCACAAATACTCGCC 24 60.026 167

chr3 5755911 AAACGAGGCACAACTGAAATATGG 24 60.083 CACATGCCTTTCCATTAATCATCCA 25 59.931 283

chr3 5757130 CTTTCTATGCACTAATCCTCACGC 24 59.79 CGCCAAAAGTAAGGGAAACAGAAT 24 60.022 284

chr3 5761278 CTCTCGCAAATATCGCTTGACTTG 24 60.55 AGATCAACTTTCTATCACGTTCCCT 25 59.812 260

chr3 5763529 ATTCCTCTGTCAATCTTCATGCCT 24 60.08 AAAAGACATGGTTGCTCGTTCATC 24 60.32 239

chr3 5764901 GAGCTTTGATTTGGTCCAATTTTGC 25 60.334 AATGACTTTACATGTAACAGCGGG 24 59.605 211

chr3 5771019 TGGCCTTCACTATCTTCTTTCCAA 24 59.957 ATAGTTTCGCAGCTCCCTTTTCTA 24 60.081 271

chr3 5774755 ATAGTATGCACCCAAACACAAACG 24 60.083 AATATTCCTCCACACTCCACACTC 24 60.081 254

chr3 5778580 TTCATGATGCCCTCTCTTTCTCTC 24 60.142 AGGAAAGACATTGTTAGGGAGGTC 24 60.019 277

chr3 5785935 TTATCAAGGGGTAGAGGTTTCACG 24 60.081 CCATCAGTTGTAGCTAGGGTTTCT 24 60.081 201

chr3 5786308 GGAAGCTCTGTGTTCATGGAAAAT 24 59.781 AACAATCTTCACAATGTACGAGCC 24 59.846 269

chr3 5786872 CCTTGGTGGCTCATTTAATCAGTT 24 59.536 TTGTTCTGTTCATCTTTCAGCCAC 24 59.963 109

chr3 5788643 TGCTCATATTGTACAGTGGCCTAA 24 59.837 ATTGGCCAGACGATGAATATAGCT 24 60.02 192

chr3 5795620 CCTCCACGTTGTCATTGATTCAAA 24 60.023 AGATTGTAGGAGGATGGAGGAGAA 24 60.078 258

chr3 5798715 GGCTCATGTTTGCAATATTTATGGG 25 59.302 GGGTTATAATAGCTTTGGTCTCTCA 25 58.172 300

chr3 5811325 CTGCAGATGACAAAGCTAGAATCG 24 60.026 CACACCTTTTCAAACCATTCAAGC 24 59.729 243

chr3 5922923 TGTAGTTGTAGGGTAAGGGTGTTG 24 59.959 TCCACCATACACCATCCAATATCC 24 59.957 235

chr3 5927034 CGAAGGAAAGGACCAACTAGAGAA 24 60.021 AAAGTGAGTTTCAAAAGGCTCTGG 24 59.901 157

chr3 5930949 TCCATTCCCTGCCTTAAATAACCA 24 60.017 AGGGAATTGAATAGCTGCTGTA 22 57.222 107

chr3 5937527 GTTCCATTTTCTGCATGCATTCAG 24 59.671 AGACTCACTTTTACCCCACTTCAT 24 59.649 274

chr3 5937977 GATCCTCCTTTGTCCCACCTTTTA 24 60.264 GGCACCAAGTTGAGTTTTGAGTT 23 60.119 164

chr3 5942527 TGTGCATGACTTCTTGTTCAATCG 24 60.32 AGGCAAAATATAGGGGAGCTTTGA 24 60.079 259

chr3 5950976 TCACTTTGAAGGAGATTGGCAATG 24 59.781 GATGCAAGTTTGTTATTGTGGTTGG 25 59.823 287

chr3 5954540 AGCTGCCAATCAAATGAGAGAAAG 24 59.842 CAACTCAAAGCCCACTCATAAACA 24 59.722 298

chr3 6004770 CTATGGTTGGGTGGCAAATAATGT 24 59.595 TTCATCGAACCCTCTTGTGTATCA 24 59.778 285

chr3 6009255 CACTACGATCAAACACGCATAAAAC 25 59.497 GTAGCTATACTCCACGTTTTGTTTG 25 58.469 182

chr3 6015262 CCCTCACCTCTATTCACTTTCACT 24 59.776 TTGGAGAGCTAGGGAAAGATTCAC 24 60.081 253

chr3 6016907 GGACATGGTTACGGATGAACTCTA 24 59.901 TTTTGTGAAGGTGAATGGGAGCAA 24 61.419 291

chr3 6023292 CGATGTAAACGTTCCTAGAGTGGA 24 60.143 CACTACGTACTCTACTGGTAAGCC 24 59.965 263

chr3 6024120 ACACACCAGTATTTACAGTTCAGTG 25 59.242 GTGTTACAAAATCAACCCAACCCA 24 60.142 268

chr3 6030853 GGAGAGCTTGAAATCTTAAAGTTGG 25 58.384 GATCTCTCTCCATATGCCATATACT 25 57.162 261

chr3 6031158 TGACTTCTTGCCCAAATTCCAA 22 58.696 CCATAAAAGTCTATCGGTCAAGAAGTC 27 59.777 95

chr3 6031737 TTCAGTTCATGTTGACAGACGTTG 24 59.966 GGTTTGGGATAAAAGGTTAGAGATG 25 57.665 205

chr3 6038015 CCCCATTCAATATAACCCTACTCGA 25 59.752 CCGAGAGAGACCAAGAAAAGAGAA 24 60.022 299

chr3 6040327 ATGACGAGGAGCCTTACAAGTTAG 24 60.142 CGGCTTCTCCTTAATCTTCTTCCT 24 60.142 153

chr3 6046065 GATTAATCGATAACACGCGCG 21 58.268 GCATGACCTAAACGATCACTTACAA 25 59.65 286

chr3 6067372 TCAGTGGAAGACATATAGGCGAAG 24 59.962 TCGTTCTCTTCTCTCAACCCATTT 24 59.96 272

chr3 6137860 AATGAATCTCTGAAGCACCCTCAT 24 60.08 AAGGACGTCAAGGTTAACTCTCTC 24 60.022 185

chr3 6139288 ATGAGTGTGTGAGAAATGGGAAGA 24 59.958 CTCTTCCTATGTGTGTTCAATGCC 24 59.905 290

chr3 6139405 AAGGAAGGAAGGAAGAAGAAAGGG 24 60.203 TTGGTTGGGTGATATAGTGGTAGC 24 60.142 238

chr3 6141789 GTTGTCCAAAACGTTCCAAACTCT 24 60.38 GGTTGGATCTTTGTGCGTAGTTTA 24 59.547 300

chr3 6142639 AAACGTCAGCAGAAAATGGGAATC 24 60.321 AGCTTGTGGTTTCTGCAATATTCC 24 60.082 230

chr3 6143763 ACTTGGCAGTAATTTTGGGACT 22 58.155 GAGGAAATAGGGGTAGGCTTTGAG 24 60.445 127

chr3 6144031 CTCAAAGCCTACCCCTATTTCCTC 24 60.445 TTTTCACACTCCCAATCCCTAACT 24 59.894 281

chr3 6150979 TCCCTCCTAACATCCTTCTCTCAT 24 60.078 CATATGCCCCTAATCACACCCTAA 24 59.957 265

chr3 6747103 GGTACTCATTTCAACACTATGGCG 24 59.966 ATAAGACTTGAGCCGGTAACATGT 24 60.081 261

chr3 6751120 CTGCTCTAAGGCCAGTTCTAGTAG 24 59.963 GAAGACCTTATTTGCACACACGTA 24 59.55 203

chr3 6755890 CCATATCGAACCGGAGTACTTCTT 24 59.962 TCCAAGAAAGGGAAGAATCAGAGG 24 60.019 295

chr3 6759917 CTTCCATTGTTCCCAGACCTCTAA 24 60.019 ACGAATAAATATAGGTGTGTGGGG 24 58.383 157

chr3 6763047 CTCTAATTGTTGTGTTGCTAGCCC 24 60.143 GTAGCAAAGCAAAGGACAAACACT 24 60.44 280

chr3 6764783 GCATGCATGTGGGAAGGTAAAATA 24 59.901 ATCCAACAATTCCACCATCTCTGT 24 60.264 247

chr3 6775550 CATTCTCCTTGGTCTACTCTTGCT 24 60.081 GAAGTTTGAATGAGACAGTGGTGG 24 60.024 266

chr3 6775892 CACACCCCACAATTACTTCATTTGA 25 59.989 CAACCCATCAAACAAAACAAACCC 24 59.904 290

chr3 6777488 GACAGCAACCACTAAATTAACCACA 25 59.991 AACTTGAGCTTCGTCATGGATTTC 24 59.845 271

chr3 6789534 TCGCGAGTACTGCTTCTATACAAA 24 59.905 CTGATGAAATTAAGAGAACGCATGC 25 59.32 244

chr3 6801717 TCTGGGTCCTATCCTTTATGCAAG 24 59.897 TCGGTGCCTTGTGATTAAGTTCTA 24 60.021 213

chr3 6802912 CTCAACCGACCGATGTATACTCAT 24 60.023 CACAATCACAAGAAAACGACATGG 24 59.324 190

chr3 6804058 TTATCCGTTGAGCTATGCACGATA 24 60.023 AAAGAGACAAATGCAGGTGAGTTG 24 59.963 259

chr3 6823504 TTTTGAGAGAGAGAAGGCTTTGGA 24 59.897 ATCATTATGCTCACACACACACAC 24 59.845 229

chr3 6826138 AATAGAGGAAATCGGAAAGACGGT 24 59.838 GAAATTGTGGGGTTTGTGTAGTGT 24 59.901 172

chr3 6833755 CTAGGGTTTCTCTGTTCTGTTCGA 24 60.022 GCGAAATCAATAAGCTCGAACGTT 24 60.724 278

chr3 6837816 ACCCTTTCTCTGATCAAATCCTCC 24 60.08 TAAGAAAATCCCTGAATGATCGGC 24 59.178 179

chr3 6861072 TGATTTCCTACAACCTAGCAAGTCT 25 59.752 ACTTTCCTTCTCGTCCCTCATTTT 24 60.202 203

chr3 6868626 CTTCATCAAACCAATCCAAACCCA 24 59.96 GTATTGGAATGGGATGTTTGGTGG 24 60.142 251

chr3 6869399 AGCTGGAAGGAAAATCGAAACTTG 24 60.023 GTCAAAACGAACACACTTCCTTTTC 25 59.715 264

chr3 6876239 CCGAGGCTAAAGAAGGAACATTTC 24 59.905 CCATAATCCAAACTTCCTGACATCA 25 59.112 210

chr3 6876658 CTTACCTCCAATTCAAACGCTGTT 24 60.023 CTACACAGCAAAACCCACTAGTTG 24 60.024 261

chr3 6881559 TTTTATCGCTTGTCTTGTTCCCAG 24 59.785 TATGGGTTTCTTGATTTTGAGGGC 24 59.534 100

chr3 6882461 TATTAGGCCGGAGTTTCACAATCA 24 60.081 TATCTCTTCCTTCCCTTCCATGTC 24 59.344 221

chr3 6891525 TCCAGTATGTCCTAGTCCTACTTGT 25 60.044 AAAGCAATCACAACATGTACGTGG 24 60.557 181

chr3 6897333 CTTGTTGCAGTTTAGGTTTGTGAG 24 58.963 AACCGAACCATCTACCTGAATCAA 24 60.02 294

chr3 6900978 GAATTCCTGGTGCTCAACTTCTTT 24 59.721 GTACAGCTGCATTCAACATAACAGT 25 60.107 299

chr3 6903417 GCTTTGGTACTTCATAATCTCGCC 24 60.025 CCACCCCAAATTCTAGTCAGTACA 24 60.02 108

chr3 6910974 GCAAGGGATTGGTGAATTTTGAGA 24 60.021 AGAGAATGTAAGAGTGAAGCTGTGT 25 59.988 242

chr3 6911371 GTTGGAGAGTTGAAATGAAGAAAGC 25 59.083 GCAACATACTTATACCAGTCGACC 24 59.256 135

chr3 6915445 TAAATCGACCCTGCCGTTTAAAAG 24 59.845 GAAAACCCTAACATGTTGCACA 22 57.685 289

chr3 6915965 CGATGGATGATCACCCTATTTAACA 25 58.77 CAGAGCACGTTCTATATTTTCCACA 25 59.419 235

chr3 6917397 GAAACAAAGGGTGCAAACATTGGT 24 61.161 AAGGTTGAGGAAGATACGTGTCTC 24 60.082 294

chr3 6920637 GAATGAGAGCCTAGAGTTAGTGCA 24 59.901 CCCATGTTCCCTGAATTCAAAGTC 24 60.082 219

chr3 6923114 TGAAGTAGTAGCAGTAGGGTGAGA 24 60.019 CGCCATAACAAAATTCTCACCACT 24 60.083 159

chr3 6923421 CCACTACACAATGCATCTCTTCCT 24 60.625 GCCATCTTAAATTCGCCATATTCCA 25 60.048 295

chr3 6968613 GGAGGATGTATTATGTGGCCTTCT 24 59.958 AGAGAACAATTAACAGGAGTCGCT 24 60.021 142

chr3 6979702 TAAAGTAAAGCCCCGAATAACCCA 24 60.019 CCTTAGCTTGCTTTTCATCCCAAA 24 60.021 297

chr3 6979841 TTACACATTGCTCCTTGAATTCCC 24 59.538 CAACTGGACTATTTGTGAAGCTCT 24 59 287

chr3 7026233 CACCAAACAAATTAGAACCACACG 24 59.262 AATGATTGGGTTTTGCAGGGTATC 24 59.838 227

chr3 7027440 GGAAACGGTTAATGTTGAAAGTTCG 25 59.603 CTGTTGGACCCCTCTTAATCTCTC 24 60.142 206

chr3 7028899 TATTTGGGTTTGGAGTCCTGATGA 24 59.709 AAGAAGTTGATTGAGCGAGGTTTC 24 59.786 282

chr3 7031636 CAAACTCTGTAGCAACCTGACATT 24 59.483 TTATATATGTACTCGAGGCAGCAG 24 57.804 285

chr3 7032385 AAGCATTGGACAACAGTTTATGGG 24 60.021 ATCATATGGCTACGACAATCCTCC 24 60.081 274

chr3 7034856 AAATTTGGGTCGGGGTGTTCTTTT 24 61.668 GGTAGACATGCCCAACAAAATTCA 24 60.022 246

chr3 7038496 ATTGTAATGGGCTGGGTTGATCTT 24 60.573 CACAATTTCACTTCCATGGATGATG 25 58.96 263

chr3 7042982 GGAGAGTTTGATTCTTACCTTTTGC 25 58.617 AGGCTAGTAGAAACCTTTTCTCGT 24 59.473 227

chr3 7044035 GTTTACCGAGCAATTCCGACATAA 24 59.669 TTAGAGGATGACTGAGTTGTTGCA 24 59.96 192

chr3 7053744 GGAGAAAAGAAAAGAAAGGGGTCC 24 59.719 TATTGGATTGGACTGGGTTGGAAT 24 60.017 227

chr3 7057588 CCACGCAGCTCATTTTGTATAGAG 24 60.025 CTAATAGATTCGGGCTTTGGAGGA 24 59.96 286

chr3 7070454 CTCTTCTCTCCTTTCACACTTGGA 24 59.96 CTCTTGTTCTCCACCCACATTTTC 24 60.023 251

chr3 7070847 GACAATATCCGGCCCACATATTAT 24 58.563 ATTCCCACCACTCTTAAACCGTAA 24 59.958 299

chr3 7074562 GCACTCACTTTTCCATTATTCCCA 24 59.538 ACTAAGATGGACTGTAGGCATTATG 25 58.237 229

chr3 7082193 GGTATGTTCTGTGTGTTGAGCTTT 24 59.724 TGGTTTGTAAGGGGTATGTTCCAT 24 59.955 244

chr3 7088398 TTGTCGTAATGGTTGGATTGTTCG 24 60.084 AACCCTCTTCCATGACAATCTTCA 24 59.957 215

chr3 7088913 GGTTTGAGAGAGGAGGTGAATCAA 24 60.263 CTGTCTCTCTCATTCTCACACACA 24 60.023 212

chr3 7094928 ACAAAAGTCTCCCTGGATATTGCT 24 60.018 CATGTCAAAGTCAACCACCATCAA 24 59.962 284

chr3 7256904 TGATGGAGATGGATGAGGAAGATG 24 59.714 CCTCTGGTATTCTCCCTCACAATC 24 60.202 252

chr3 7262607 CTCTTCTTTTATTTCGTCAATGCCC 25 58.97 CAAAACTCAAACCCTAAATCGACCA 25 59.991 300

chr3 7270300 GGTACTTTTCGCTGTCACTTTCAA 24 59.965 TCCTAGCCACACCAAATTTCCTTA 24 59.956 203

chr3 7271166 CTCGAGACATCTTTGAAGTAATGGT 25 58.897 CCAAATATTTTCGACCGACTATAGC 25 58.182 222

chr3 7272811 AGACATTATTGAGGGAGATTCGCA 24 59.898 AAGATCACACAGTCCTTTGCTTTC 24 59.723 201

chr3 7274671 GGTTATTGTCAATTTGTTGCGGTG 24 59.85 CCAATTTTCAAAACTCCCACATCC 24 59.003 158

chr3 7284362 CCTTCTCCAATTTCGTTTGCTTTC 24 59.319 TAGCCATTACATCTCATTGCTTGC 24 59.723 283

chr3 7287383 CAGCTAGTCGTTGTTTCTTTTGGT 24 59.965 GACATCCACTACACCAACTACGAT 24 60.142 280

chr3 7289468 ACTTCCCTCTTGTTCCTCTCTTTC 24 59.959 GCCCTAATTTGACACAAGCATAAAG 25 59.193 268

chr3 7290170 CCTCAGCCACTTTCTAAAAGGTTA 24 58.686 TCTCCATCTCACCCTCGTTTATTT 24 59.53 218

chr3 7297533 CGATCAGACCCTTCTTTGCATAAC 24 59.965 AGTTCGGTTCCTGACATCAAACTA 24 59.96 281

chr3 7307486 TTAGTCGTTGTGCTAATGGTGGTA 24 60.021 TGGCCTATACACTAACTACAGTCA 24 58.488 117

chr3 7311499 TGTACAGATTGGCACTAGGTTTGT 24 60.202 ATTTTCCTTTCCAGCACAGTTCTG 24 59.962 206

chr3 7324304 CCAAAATTTCACCTCGCGATTGTA 24 60.379 TGCATGACTTCACGAAACTAGTTG 24 59.789 200

chr3 7347428 TGGAAATGAAGAGATCTGGAAGCA 24 60.019 GAGCCAACAAATTTTAGGTCACTGA 25 59.99 295

chr3 7354593 TATATGCTTTGGTTATAGTGCCGC 24 59.306 GGACATGTTACATTTGCCCTTGAA 24 60.022 269

chr3 7357199 TTTAGCCTCGAATCTTACCTGCAT 24 60.142 ACCTTGGATCTCAGTTTTGCATTG 24 60.022 280

chr3 7365982 TTCCACAGTCTTCCCAGATGATTT 24 59.957 TACAGAAAGGAGAAGGTACACTGC 24 60.021 134

chr3 7386098 GGTGACTGACAAATGGGAATGTTT 24 59.961 AGTCCCTAATCTCAAGCTTGGTTT 24 59.957 244

chr3 7392774 CCTATTGATCGTAGTTGAATTTGCG 25 58.871 AGAGATGTTTACTGTGGGTAGGTT 24 59.155 194

chr3 7397699 CTGAACCTGCCCTCAAACAAATAT 24 59.536 GAGCCACACCCTACCGATATTAAT 24 60.02 300

chr3 7398782 ATAATGTATGTGCTTTGCTTCCGG 24 59.963 TCAAGAGGGAGGAATAGAGGCTAT 24 59.891 295

chr3 7406061 GAAGAGGCCAAGAAAGAAACTTCC 24 60.023 TCCCCTTTTCCTCTCTCTCTAGTT 24 59.954 261

chr3 7407361 ATCCAAGCCGTACTATGACCTTAC 24 59.961 TATTCATACACCGGCAGTCTTCAT 24 59.899 165

chr3 7421723 CGAGAGAGAGAAGAGAGAAATGGG 24 59.963 GTGTTGGGTGAAAGTGGAAGAAAT 24 59.901 216

chr3 7421945 TCACCATTGTCAATTCTTCCTCCT 24 59.957 ATCTAACACCGACCCTTTAGCTTT 24 60.02 127

chr3 7426233 CCGTGAATTTTGTGTTTCGTTTGG 24 60.26 AAGCCACCTTACACAGATCTGATT 24 60.019 271

chr3 7427697 TTCACTTCGAATGAGTACAGAGGT 24 59.477 GCATCAAATTGGGCATATCAGT 22 57.404 185

chr3 7432984 TTTCAGCTTTTGGGTTAAGTGGTC 24 59.901 TGAATGGAAGGCAGTCAAAGTAGA 24 59.958 234

chr3 7433252 TATCGACTCGGTTGTTTGTAGTGA 24 59.784 GACCTGGACGATTATCAAAACACC 24 59.906 299

chr3 7443118 CCTCTGAAACTATCCCACCAAGAT 24 59.835 CTAATGCTCTCAAGGTCAGGACTT 24 60.081 299

chr3 7610122 GATTAAGATACACTACCCGTCCCC 24 60.022 TTGAGATCAGAGACAAGGAATGCA 24 60.02 257

chr3 7610827 CTCGCTCGCTAGTTGATGATATCC 24 60.963 CAACCCCAAGAAAACTACATGACC 24 60.022 296

chr3 7610955 CTCGCTCGCTAGTTGATGATATCC 24 60.963 CAACCCCAAGAAAACTACATGACC 24 60.022 296

chr3 7613968 GAACGGGTAAGAGAGGGTAAAACT 24 60.021 ACTCTTCATAATCGCCATCTCCTC 24 60.022 144

chr3 7618827 GGACTTTGGTGCTGATGGAAG 21 59.185 GAACACCAAACTCTCAAACGTCTT 24 59.906 285

chr3 7631362 GAATTCCTCAAAACTAAGCACGCT 24 60.083 CCTGAGTTTATGAGAGAGACACGA 24 59.604 197

chr3 7635058 CCGCTTCTTAAATACGACATGGTG 24 60.26 GGCAACACGAATATATCCCACAAA 24 59.663 280

chr3 7635590 CATGCCCTGAAAGCTCCAATAAAT 24 59.9 TTATTGTCTTCTTCCCTCGAGCAA 24 60.021 298

chr3 7649839 CGGAGGCGATTTTGTTGTTAAGAT 24 60.143 CCGACACATGGGAAGTACATTTTC 24 60.143 297

chr3 7800525 ATGTTTCCTTGTGCCACTCAATTC 24 60.262 GGTTCACGGACTTTCATTGTTGAT 24 60.023 300

chr3 7804769 TGGTCGTAGTCATTTAAGCATGTG 24 59.37 TCCAAGTACATACAGCTCAGTAGA 24 58.493 253

chr3 7807771 CTAACCATTTCTCCACCTCCTCAA 24 60.019 CTTCCTTGGCAAATCTCATCCTTC 24 59.902 240

chr3 7808352 GGGGAAGGGAATAAAGGGTAGAAT 24 59.581 GATCAAACCAACCAAGCTTCTTCA 24 59.962 216

chr3 7822911 ATAATAGTGGGGTTGGGGAGTCTA 24 60.078 CATTATCAGCTCCACCCCTATCAA 24 59.958 140

chr3 7827586 GAAACATTGACCCAAACCCTCATT 24 59.96 GAACTCCCTTACCATTACCCAAGA 24 59.773 84

chr3 7829449 TTGTAATGTTGGTCGGGATTTTGG 24 60.022 GAATTCCCAATTTCCCGATTTCCA 24 59.839 263

chr3 7829581 TGGAAATCGGGAAATTGGGAATTC 24 59.839 TCCCATTTTCTCCTCTTTCCTGAA 24 59.646 104

chr3 7833034 TATTGTTGTTGAGGTGGCAGAGTA 24 59.959 AAGTAGGGAAATGCATGGAGATGG 24 60.691 215

chr3 7834081 GGAGGGTTCAATTTGGAGAGTTTG 24 60.022 GAGAAATGGGAAATGGGAAATGGG 24 60.142 168

chr3 7834202 CCCATTTCCCATTTCCCATTTCTC 24 60.142 CATTCTCTTCATGAACGTGCTCTC 24 59.967 289

chr3 7844586 TGATCATTACCAACCAGAAGACGT 24 60.021 TTAGTTCAAGTCTCTTCCTGCGAT 24 59.779 249

chr3 7850732 GGCAACCAACAATCAGAAGTCAAA 24 60.441 GAGGAAGTGTATGCCAGAGTGTAT 24 59.9 272

chr3 7857651 TAGAGAGGCGGTTCATGAAGATTT 24 59.838 CCAGATGAATCCGAGCAAAATTGA 24 59.903 288

chr3 7860845 AGAGTTAGGTCAAACACACGTGAT 24 60.202 TTCAAATCACGAAAGGTTATGGCG 24 60.379 265

chr3 8104791 AGGATGACTGGGACACATTGTTAA 24 59.957 ATTTCCATCTACAGGGTGTTCAGC 24 60.868 271

chr3 8156730 ACTAGCATGACTACTCTATAGGGCT 25 59.985 CATCTACATCTCACACTCAAACTCA 25 58.607 299

chr3 8160990 AAGACCCTTACATCTCCCTGTTTC 24 60.019 TGGGGTTGTATGATTCTGAAGCTT 24 60.264 196

chr3 8168849 TTACCAAAGAGAGCACCCACAAT 23 60.181 AATGTTGGTAGTCAGATCTGCAGT 24 60.02 158

chr3 8169725 GATTGAAGACGAAGAGTGGGAAGA 24 60.322 CCATCCACCACCAATAGACCTAAA 24 60.08 157

chr3 8173352 ACAAGACATCTCGGATCACATTGA 24 60.081 ATTTCTTACCTAACCGTTGGCTCC 24 60.866 243

chr3 8178770 GAATTAGATCCGAAAGACTTGCCG 24 60.026 TGAATTCAAATGTGGAGGGAGGAT 24 60.017 246

chr3 8183153 CACAAGATTCTTCCGAGCCTAAAC 24 59.906 ATCATGTACATTTTCCTTAGCGCG 24 60.025 246

chr3 8183715 ACACTCCGATTTTCCCAAACCTAT 24 60.264 TGGGTAAGGGTGATAGAAGTATTGA 25 58.793 182

chr3 8185287 ATCACTCAACTAGACATGCATGCA 24 60.625 TATACCTTGGTGTGAGTGTTGCAT 24 60.263 219

chr3 8193277 ACATCCAAAGAACCTACCGATTGA 24 60.02 CCATAGGACTTGGGATTCGTACTT 24 59.899 141

chr3 8271444 ATCAAGTGTTCGACCATACATCCT 24 59.838 CCACTCTTCCGGTATGTAGAACTT 24 59.84 260

chr3 8280536 CTTGGAAGGCTGTGATTGTTGATT 24 60.022 GGAAACCAGAAAACACACTCAACA 24 60.082 197

chr3 8296837 TTATGAACTTGGTTGGTTTGGAGC 24 59.961 CTCAACAAAACGAGGGGAAGTTAC 24 60.024 171

chr3 8304600 CATTGTCACATTTGTTTACGCC 22 57.116 AGATACGAGATCCTACCTGATGCT 24 60.264 144

chr3 8310351 TGTAATGTGAAGCCCATGGAAAAC 24 60.022 CATGCAAAGAAGGGAAAGATGGAA 24 59.779 185

chr3 8311907 CGTCATCTCCACTAGTACCTTTGA 24 59.602 GTGTGTTTGCATTTGTCCTGGATA 24 60.022 276

chr3 8316296 GGTTCCTCCTCTATACTCCATCGT 24 60.75 GAAACCATCAAAATGCATCCAAGG 24 59.367 287

chr3 8321262 TGAGTTTCGTGGATTCTTCAGGAT 24 60.02 ATGGATCTCGTACTCACATTCACC 24 60.202 212

chr3 8333413 GGTGGATCCTTCAAACTAATACCG 24 59.185 TGGGGCTAGTTTCTGATATTTGCT 24 60.08 298

chr3 8339399 CTCCCATCAACCACAATTCCTCTA 24 60.08 TACTATGCTTGGTGACTATGGTGG 24 59.899 207

chr3 8351222 ATGTTCAAAGGGTTACAAAAGGGG 24 59.653 GTGATCACTGGTGCACATTCAATA 24 59.604 285

chr3 8351710 TCATGCTGATACGCTCTCTTAAGG 24 60.262 CCACCAAATGTAATGTCTTCGGAG 24 59.905 265

chr3 8356962 AGGCACATCGTATCAATCTCCAAT 24 60.202 CCACGAATAGTATAGCCATCCACA 24 60.021 155

chr3 8367475 GCGGTTGTTGAATTGTATGATCCT 24 59.904 ATCCATACATGATTCGGCATACCT 24 59.774 216

chr3 8370479 CGGTTTAGGATTTGTGAAAGGCTT 24 60.022 AGCGAGTGAATAACAAGATAAGGGA 25 59.871 255

chr3 8382772 CGTGGGTTAGTGTAGGCTATTTCT 24 60.142 ACCCTCTTCACTACTGTCAAGAAA 24 59.346 225

chr3 8385831 TATGTTGTGCTGCTGTACTTGTTC 24 59.787 CCAAGGCTCAAATACATTTACGCA 24 60.142 202

chr3 8389576 AGGTGTGTGATTAGGCAGGTAATT 24 60.018 GAACCACGCAAATTTGAAACCAC 23 59.999 148

chr3 8390683 TGGAAGGTGATGTATTATGCAGAGA 25 59.634 CGCTCACTCACTCACAATCAAAAT 24 60.084 265

chr3 8392251 GAAAGGAAAGGAATTATGGAATGGG 25 58.18 TTTTCTGTTACCTCGTTTTGGCTG 24 60.202 171

chr3 8400000 GGCAGTTCTCCTAGTCTCTAACAT 24 59.353 ATTTAGTTGCATGGTGGTGGGTTA 24 60.754 187

chr3 8403261 CTCTTTGATGTGATTGACGGTTGT 24 59.786 AGTATATCCTCGACCACACTAGTC 24 58.693 115

chr3 8403469 GACTAGTGTGGTCGAGGATATACT 24 58.693 GCCTTTCATTTGCTTCTCTTCGAT 24 60.143 257

chr3 8404687 AGACCAACTAGCATAATCGACGTT 24 60.142 GCATACTTGTTTTCCTTGTATCCAG 25 58.445 260

chr3 8420646 GTGCTGTTGTCTTTTGATAGAACGA 25 60.05 CCTGCCCTGAGACCTACTTTAATT 24 60.08 290

chr3 8425623 ACGTCTTCTTATAGTCATGGTCTCA 25 59.114 CTCTCCAACTAATGTGAAAATGTGG 25 58.385 168

chr3 8435972 ACCGGCATTTCTTATAGCATTTCG 24 60.024 GTGATGATAACGAAACCTCTGCTG 24 59.967 256

chr3 8439382 CGAGTGATGAAATGGGAAATCGTT 24 59.905 TCCCATAAAATTCGATCCCAATCC 24 58.923 108

chr3 8443551 TGGCTTACTCTTGTGTGTATGTATG 25 58.894 GATTTTGCTAGGTATGGAATCGCT 24 59.243 156

chr3 8459250 TTATTGCGAAGAGTCTGTCAGTGA 24 60.022 CCCTTTGGCCTTTTGAATATGTGG 24 60.624 276

chr3 8460746 TACACTCATCGTCCTTTCCTTTGT 24 59.96 GGAAAGTGGGAGGAGGTAAGAAAT 24 60.018 239

chr3 8461788 TCCAAGAATCAAAGGGAAGGATGT 24 59.955 GGAATGGGATTGCTCTTGAACAAA 24 60.021 279

chr3 8472269 ATCATTCGGCTCCAAAATCCTAGT 24 60.141 GCTGGCCTGTTTGATTACTTTTCT 24 60.022 247

chr3 8474558 GCGTAGTTGGATCTAGGACATGAT 24 60.022 TGTAAGCAATGTTTCTGGAGATGC 24 59.843 266

chr3 8493379 CATTTGAGTGTTTCGCCCTTGAA 23 60.243 CAGGTGGAGTTGCTTTTGTTGG 22 60.482 247

chr3 8514211 GGGGAGATCAAAACTGTAATTTAACCC 27 60.364 TGTAGTTGATATCTGCTTCTGGGC 24 60.443 202

chr3 8555135 GTGTTGTTTTATTATGGGGACGACA 25 59.818 TCTCGCCTTGGTTACTTATCACAA 24 60.021 229

chr3 8570725 GGGAGGATGAGTGGATGGATTTAA 24 59.895 GTTGTTAGTGGTTGTGTTAGAATGC 25 59.087 146

chr3 8572552 TATCACGTTCAAACCTCGAATTGC 24 60.143 TAATTCCAAAGAGAAAAGCAGCGG 24 60.083 292

chr3 8574137 GGTTTCTCAATTCCTTCCACCCTA 24 60.264 GATTGTCAAAGTTTAGGGTGCACA 24 59.963 279

chr3 8574833 AATTTGCTCTCTCATGGGTTCTCT 24 60.019 TGTAAAATGCTACCTCCAGTCACA 24 59.959 237

chr3 8589474 AGTTCAGTTTAGCTCTTCCCTCAG 24 60.021 CCGCCGTAGAAATATCCACTAAGA 24 60.023 200

chr3 8651039 CTGTCTACCATGCATGTTTGTGAC 24 60.379 GTAAGCGAGATCAAACAATCACATG 25 59.038 270

chr3 8652969 GGGATAGTGAAGATAGTCGTGGAC 24 60.023 ACTTACCCGAATTTTAAGCTTGGC 24 60.082 162

chr3 8656525 TTCGATTTAAGCAATGTCCAGGTG 24 59.844 TGCCCTTCTTTCACTATAAACCAGT 25 60.222 291

chr3 8658477 GAACACTTCATTTCTTGCCCGTTA 24 60.024 GGTTTTCCCTTTCACTTGCTGA 22 59.307 141

chr3 8659155 ATCTCATAGAACAAAATCCGGGGT 24 59.834 ACCTCCAACAGAAAGAGAGAAGAG 24 59.717 251

chr3 8664039 AACAAGAGACGAAATCCTGTACCT 24 59.716 AGCCCTCACTAATCTTAACGCTTT 24 60.324 267

chr3 8680537 CAAATTGAGTTAGGTTTGTTGGCC 24 59.25 CATTCTCAATTCGACACTCTTTGGA 25 59.59 270

chr3 8680927 ATGAAAAGACATGGCCTCAAGTTG 24 60.022 AGTACCTAGGTTTCAGAAGTGCTT 24 59.406 248

chr3 8692541 ATCTTGTTCCAGGGTTGATTCCA 23 59.924 GCATCCTTCGTCACGTAATACAAG 24 60.027 281

chr3 8698327 CCTGCTTGATTATTTCCTGCTCAG 24 59.964 AACCCCTTAATCCGTGGAAAAGTA 24 59.957 253

chr3 8701250 CAGCCATCATCATCCATTCACTTG 24 60.262 GGAATCGTTCACTCATTGTCTGTC 24 59.908 207

chr3 8720408 CATCGGACCAAAAGCAAGCTG 21 60.402 AGCAAGATGGATGGTTTGACTCTA 24 59.774 297

chr3 8892897 TCTACGTTGTTACTCTTATCGGTGT 25 59.587 TGAAACGAAGCATAGACTTTTGACC 25 60.05 285

chr3 8961599 CAGGAAAAGATGCTTGTCTCATGA 24 59.301 GAACAGAATCCAACAACTTCACCA 24 59.662 209

chr3 8962448 AGTAATGCATAACCCTTTGTCCCT 24 60.017 GCAGCATTTGACATTTCCATTTCC 24 59.906 216

chr3 8990421 TGAAGGTTGTTGAAGACGTTATGG 24 59.486 TGACCATCTGATCATGATTGGGAT 24 59.647 279

chr3 9189380 AGGAAATTAAGAGGCTATGGTCCC 24 59.895 CTCCAACTATCACTCACAAACATACTC 27 59.723 251

chr3 9219696 CCAATCCAATCGACTAAAGAAGCG 24 60.26 AGTGAGTTGAAGATAGGTGAAGCT 24 59.47 244

chr3 9225643 AAGAAGAGAGAAAGGTCAGAGTGT 24 59.101 ATATCTTCTCTCAACTTGCAGGCC 24 60.686 169

chr3 9226352 GGGAAGAAAGCCGATTTGAATAGA 24 59.118 TGTGGATGACGTTGGATATATGCT 24 59.96 135

chr3 9284674 GGTTATATGACAGCTGCGAATTGA 24 59.489 AGAGATCTTCAGACACACAAGCAT 24 60.02 197

chr3 9286116 ACAACGAACGACTTAACACTCTTG 24 59.733 TTTAGCAATCACATTAGCATCCCG 24 59.724 267

chr3 9287203 GGGAAAGGAATGAAAACGCATGAA 24 60.561 CCCCAACAAACTTCATCTTTCTCC 24 60.022 270

chr3 9304902 CAAAATCTAAGGGTGGCCAAACTC 24 60.322 TTGGAACTTGAGCTTTTGTTGC 22 58.479 202

chr3 9306275 CAAACCTCTCCCAACCTTTTCAAT 24 59.655 ATAGGGCTCGCTGATTATGGAATT 24 60.019 194

chr3 9320245 AGTAACATAAGGCTCCACTCTGTG 24 60.081 GTGCTGTTGATGTTTGGAGAACTT 24 60.202 297

chr3 9323457 CAAAAGATCCTAGTGAAAACGGCA 24 59.785 AGGATAGTTGCTACGAGGGAAATC 24 59.961 297

chr3 9332546 ATAGAAATAGCGAGCTTGGGTCAT 24 59.959 AGACGAAGAAAGAACACAAGGTCT 24 60.142 196

chr3 9413180 GTTTAGAAGTGAAGCCAACCCTTT 24 59.659 GGTAGTCCAAATGTTCACTCCATG 24 59.604 297

chr3 9413825 CAATTTAAGTTGGTAGACTCGGTC 24 57.595 ACAATTCTAACGTGACATAAGGCG 24 59.671 285

chr3 9453925 GAAATTTTGGGAAGTGGTGTGACA 24 60.142 TGAAAAGGAGTGGAATACAAGCCA 24 60.447 285

chr3 9482706 ATTCTTGTTCTGCTCAATCCATGG 24 59.598 ACCACTCCTTTATCACAATTCCAC 24 58.99 299

chr3 9493279 CCCTCTTACCTTCCCCTTTCATTT 24 60.264 AGGATGTGATAGCTAGGTAGGGTT 24 60.141 278

chr3 9497242 GCACTTCCACTACAACACAACAAT 24 60.202 CCTACCCCTTCATTCGATACCAAA 24 60.142 258

chr3 9507446 CTAGTTGGACAGCGGTAGACAC 22 60.416 CACCTTCTTGTTCTTACTTGTTGCT 25 59.933 291

chr3 9521221 TTTGATGATGATGATGATGAGCCG 24 59.545 ATATCATATTGGCCCACGTTCCTT 24 60.203 217

chr3 9524870 CTTTCCTTCCCTTTTGTCCATTCC 24 60.021 GGATAGTGTAGTGGGTAGGGTTTC 24 59.899 280

chr3 9535918 GTAATTTATGCAATGCGGTTCGAC 24 59.563 ATCTTAGCTACCATCCCTCACCTC 24 60.999 292

chr3 9614234 GCATTGTTGTATATCTGGTCTGCC 24 60.024 GAAATCAGAGATGAGCCAACCATG 24 59.964 224

chr3 9630449 ACCGATTCAAAGGACCGAAATAGA 24 60.081 TTCGGGCGAGATTGAAAATTATCG 24 60.025 281

chr3 9631236 CGTTCTTACACATGCCATTCACTT 24 60.083 AGCAAATGTGATAGACTCTGCAGA 24 60.081 240

chr3 9669977 TGTTTGAATGGTGGGTTTTGAG 22 57.601 AAGGGTTTTGATGAATGTATCCGC 24 59.902 188

chr3 9676012 CATCTCAACTAAAATGACACCCGC 24 60.437 GTGGGAAGCTTTCAATGGTGTAAA 24 59.961 289

chr3 9695938 GAACCTTGATTTTGTGATGCCATC 24 59.134 GACAAACATTCGAGTTCTTGGAGG 24 60.084 211

chr3 9697124 AACAAAATGGAGGAACAGCTTAGC 24 60.022 TTCCCCTCGATGAATTAAGACCTC 24 59.899 151

chr3 9701847 TTCCAACTTCTTTTCTTGAGCGTC 24 59.965 CTTTTGATGAGGAAGATGCGTACC 24 59.965 142

chr3 9705663 TTTGTGGAAGGTGGCTTAAAGAAC 24 59.901 ACCCCATAGACGCTAATCAAAAGT 24 60.081 212

chr3 9706566 ACTTGAGAACTTATCAGACCACCA 24 59.407 TACTGCCCTTCTTTAGACACACAA 24 59.898 105

chr3 9716607 GTTTTGGAACGGGGCATTTTAT 22 58.067 TGTAAAAGTCTGTCAGAGCAAGAG 24 58.707 214

chr3 9720942 CTTGGACTCACTCAATTCAAAACAC 25 59.026 TCTCATCTTGCCCCTGTTCAAAA 23 60.436 223

chr3 9722453 TCAAATTACTTAGACGAGCGGGTT 24 60.322 GGGATTTCACTGTCAAGAACGATG 24 60.143 267

chr3 9728111 AATAGCGACGAGACTTGAGTACTT 24 59.604 TTTGATGACATGTGCGAAGGTTAC 24 60.084 135

chr3 9731336 CGATAGCCCTAAATTACTGTCCGA 24 60.023 TCTTAGCACTACAAATACGCAACTC 25 59.424 254

chr3 9733510 CCCCTAAAATATGCAAAGTTAGTCACC 27 60.416 CAACACAGTGCACTACATCATCAG 24 60.143 149

chr3 9734588 AAGGGGAGTTGCTTGGTATTTAGT 24 59.956 TGTGAAGGCAAAACTAGGTAAGGT 24 60.141 294

chr3 9737350 TATTACAACGTTTGAGGGGAGGAG 24 60.081 GTTGCCAAACCCTACACACATAC 23 60.058 234

chr3 9737638 ATTGCGTATTCCTACTTTCCCCTT 24 60.08 GTGGAGGGGTTGATGGTGTTTATA 24 60.325 121

chr3 9742569 AAAGGAAGAGGTATTTGTCGCTCT 24 60.02 AAAAGACACCTCCCTACATCTAGT 24 58.657 257

chr3 9742943 GGTAGTCTTCAAGCCACATAGTTG 24 59.368 CGTTTCATGGAGAGTCACTTCTTT 24 59.248 300

chr3 9750435 CAAGAAAGAAGCGAATATTGTAGGG 25 58.281 AGAAAGGAAAAGAAGAGCCAAACC 24 59.658 274

chr3 9754222 TTGACCTCTCATCTGCCATGAAAT 24 60.325 GAATAGGTCAATTGCAAGAAAGTGG 25 58.905 299

chr3 9759088 CACTTGCTCCCTAATACATCATAACC 26 59.572 GTACATAATGCAGGTGATGATGACA 25 59.24 168

chr3 9761185 TTTCAATGCTAGACCGTGTTTGTC 24 60.024 TCTCTGACCGTTTGAACATCGATA 24 59.843 254

chr3 9770732 ACTGATGATAGAACCGAACCTTGT 24 59.777 GCAAGATTTCTAGAGGTGGGTTTT 24 59.233 298

chr3 9773809 GACGGCAACAAAACAAGTACATCA 24 60.497 GAGTTGAACCACACTGTAAATCTGT 25 59.472 280

chr3 9781142 TCCCTTTTACTCCCAACTTGTTCA 24 60.08 ATAAAGTTGAGGAGACACGTAGGT 24 59.229 253

chr3 9782162 CGAGAGGTTCAAATCTTTTGTCCC 24 60.083 CATCCCATGCATTCGTTGACTAAC 24 60.496 261

chr3 9807337 GCATAGACTCAGTGTAGGGCATAA 24 59.961 TAGGAGTTGGATGGATGGATGGAT 24 60.702 184

chr3 9823229 TTGTACCCTTTCATGTTCCAATGC 24 60.022 CGCAGGTTAGTTGACAATTACACA 24 59.788 276

chr3 9823703 CCTCGTGATTGCCTTCTAATTTCC 24 59.964 AGACAACCAGAAACTACCTACCAA 24 59.343 300

chr3 9875121 AAGATCATAGTTTGGTGGGTGTCA 24 59.957 TGGTGATATCCCATTTGTGAGGTT 24 60.018 249

chr3 9876258 ATTTGTGTAGAACCTCCTCAGTCC 24 60.02 GAAAGGATAAAAGAGGGAACATGAG 25 57.673 150

chr3 9893839 TTCTAAAACAATATCACCACCGCC 24 59.603 CCCCTAACCTTAACCAACACTCAA 24 60.447 263

chr3 9951471 GGAAACAGGGGATGACGTAATAGT 24 60.142 CATGGGTAGAGTTGATGGGGTAAA 24 60.08 229

chr3 9953872 TCGGTCTAATATGCGATGCCTAAT 24 59.84 CTGTCTCCCTTAATCCTTGGTTCA 24 60.019 278

chr3 9957060 GTGCTCAACTTCGATCAAAGATTG 24 58.915 TTTGTTTTGTACGTGTGGATGAGG 24 59.964 195

chr3 9977321 TGAGATCAGCTGTGTTAACCATGA 24 60.02 TGCCGACTAAAACACTTCAATTCC 24 60.024 269

chr3 9982638 AGGAGTAGGCCCAAGATTTTAGTG 24 60.08 CTCCTCGGTCCAAATCAATAGCTA 24 59.961 252

chr3 9987286 GGGTTGAAGAGTGTTAAGTGCATC 24 60.083 CTCAAACCAAGTTCAAGTCCACAA 24 59.842 131

chr3 9987433 TTGTGGACTTGAACTTGGTTTGAG 24 59.842 ATTTTCTTGCCCTTGTGATTGGAG 24 60.021 291

chr3 9987786 GTAGGCATCATAAAGAGAGAGGGG 24 60.021 CACCCTGTAATCATCCAACAACAC 24 60.083 218

chr3 9993071 TCTTTTGTTGGGTGTCGTCTAGAT 24 59.96 ATACGTTCACACCTCATCTTTTGC 24 59.846 199

chr3 9993410 GCAAAAGATGAGGTGTGAACGTAT 24 59.846 CAACCACAACACCTTTTAACCCTT 24 60.081 287

chr3 10010768 CAACTCCTCCTACTCACACTCTTC 24 60.082 GATGACCCATTGTTAGAGGCCTA 23 59.609 157

chr3 10011462 ACACTCGCATATGTAGCCGATTAA 24 60.262 ATGCAAATGAGAGTCTATCACGGT 24 60.142 194

chr3 10018209 CGTGTGCTTGTGAGTAAATGGAAT 24 60.083 CTACTGCCATGGGATAACGAGTT 23 60.181 300

chr3 10029559 AAAGTTAGTCGGGGAGTTGAAGAA 24 59.898 GCATCTAAAGCAGCTCAATCGATC 24 60.377 295

chr3 10030189 GGACCTCTTGAACGAAACCATTTT 24 59.962 GTTCCTCTCTCCTACCTTCGTTTT 24 60.021 268

chr3 10030514 AAAACGAAGGTAGGAGAGAGGAAC 24 60.021 ATTTTGCACCCTATATGAGCCTCA 24 60.141 211

chr3 10031454 CAGAGCCTTCTTGCAAATTGGATT 24 60.322 AATCCACCACTCAGCAATTTCCTA 24 60.264 267

chr3 10032916 GCTTTGTTATCTGTGGCTAGCATT 24 59.903 CCCTCTCAACAACCATGGAATCTA 24 60.08 300

chr3 10035613 TCATGTCACATGGTATAGCAACTCT 25 59.871 AAACTCAACTCTCCAAACCAAACG 24 60.142 108

chr3 10036852 GTCATCGTCCCATCTTGATTTTAGT 25 59.182 CCGAGGTGGTTAACTTTCTTGTTT 24 59.663 300

chr3 10037207 CCTCGGAATCTTTGAAACTAGAAG 24 57.345 CCCTCCAATTCAATCATTCATTCCT 25 59.399 300

chr3 10043470 TATGATCCCTTCCTCCCTCTTGAT 24 60.14 TTGTCAAAACGAGCAAAACCCAAC 24 61.382 269

chr3 10049618 ACCTAAGAAGCACATACCCTTTAGT 25 59.512 GACACCATAAGACAAGGACAGGTA 24 59.778 300

chr3 10052348 AAGTAGGATGCAAAAGGAATTGGC 24 60.081 GAGTCACGAAACACAACCTACATG 24 60.085 275

chr3 10055484 GAACACGCGTCAAGAATGGTAAAT 24 60.378 CACTATTTGTTTTCACCAGGCAGT 24 59.962 252

chr3 10066346 TTCCGAGTTAGAATACCCAAGCAA 24 60.02 CTTGTTGAATTCTTCGAGGTGAGG 24 59.847 190

chr3 10066671 GCATCCAGAGAACTTTAGGGTTTG 24 59.843 GGTGTTCGTTCGTTTGTTCTTCTT 24 60.436 281

chr3 10076289 GCATGTGTTTCCTTAAGTCTTCCA 24 59.481 GGAGAGTGGAGAATGGGAATTGTT 24 60.569 199

chr3 10082763 TGCTGTAATGTCCTAGGATTCAGA 24 59.038 CGTTGTTACTTGAATTCTTGTGGC 24 59.325 280

chr3 10084041 TCAGTAGCAGATGGAAATGGATGT 24 59.835 ATCCACAATTCAGTACAAAAGGCG 24 60.083 272

chr3 10108540 TAGGTGTGTGAGTTTTAGCCAAGT 24 60.142 AAGGAACAAGGATGACTAGCAA 22 57.359 223

chr3 10125847 TATGGTGATGTTGATGAGTGGGTT 24 60.019 GAGGGTGACAAATAGGCGAAAATC 24 60.202 244

chr3 10156591 GGTTTGAGCCAGTAGAATCGAATA 24 58.643 TTATTGAAAGTGGTGGATTGTGGC 24 60.022 245

chr3 10208035 TGATCAGCTCCCTTCAAAATCACT 24 60.264 AAACACCATAATCCAAACACACCC 24 59.96 166

chr3 10278544 GTTCACACTCTTATGGCTTTCGTC 24 60.143 GATTGATTCACCAGAGGAACACAC 24 59.845 262

chr3 10301667 AATCTAATCCAAACAACCCACCGA 24 60.508 ACACGCAATCTTCCAGTACTACAT 24 60.082 91

chr3 10302526 TCTACAAATAACCTTCCTCGACCC 24 59.839 ATGAGTAGTTCCCACGTCGTTTAA 24 60.022 289

chr3 10307645 TTATTTCCCATACCACCAACTCCG 24 60.627 CCGAAAATGGTGATGAATGGGAAT 24 59.9 272

chr3 10326938 TTTCTTCTCGTAATCCTACCGACC 24 59.903 TGGATTAGGTGGATGTTGTGGAAA 24 60.203 250

chr3 10337135 GGATCGCCATGAGAAGAATGAAAA 24 59.664 TTATCCTACACACTCACCACAAGC 24 60.563 297

chr3 10339510 AGACCAATTGTACTCACCCTCTTG 24 60.263 CACACTCCTGTCTCTTTTCTCTGA 24 59.961 273

chr3 10339706 GTCAGAGAAAAGAGACAGGAGTG 23 58.445 CCCGACAAACACAACAATAACTCA 24 59.964 288

chr3 10339938 TGAGTTATTGTTGTGTTTGTCGGG 24 59.964 CACTCAAGCTCCACAAACTCTTTA 24 59.184 208

chr3 10341926 AACGTCAAGGTGAATGGGATCTTT 24 60.75 CGAGAAGGATAAGAAATTTCAACGG 25 58.521 230

chr3 10342699 AGAAGGTTTTAATACACACGGTCAC 25 59.532 GCCGTACAGATAAGCCGTCTATAT 24 59.904 293

chr3 10387963 TCCCATATCTCCGCTTAAGTTTACA 25 59.636 CAACTGACCTTTCAAAGACTGGAC 24 59.964 125

chr3 10431776 CATGAACCCTTCCTACTTTACTTTC 25 57.621 ACCAACAAAAGTCGATCAAAGCTG 24 60.498 281

chr3 10432671 GACGGGTGTAGATTCTCTTCTTCA 24 59.842 AAACGCATACCTCGAATCTCAGTA 24 59.903 135

chr3 10440772 TGAAGTTCCAAGGTTTGTCATG 22 57.083 TTTGAGTTTTGACCACTTGCTTCC 24 60.382 271

chr3 10440973 ATGGTCAAAGGTTAGGTGTTGT 22 57.821 CTTATGGTTTATTGTAGGCAGCCC 24 59.722 157

chr3 10448888 AGGCTGGACTTGACTATGTTTCTT 24 59.958 CAAGTTTTGGTTAGGCTTTTCAACG 25 59.995 273

chr3 10449974 AAGTGAAGGTAAAAGCCAAGCATC 24 60.022 TCATAAGCCGGACATTTCTCTT 22 57.512 244

chr3 10450829 TGTGGTGCATAACATAGCCAAAAG 24 60.082 TCCTACAGAACCGAAGAACACAAT 24 59.96 291

chr3 10459445 GGTCGAAAGTTAGAGAGATGGACT 24 59.6 GCCATTTATCAAGCAATCGAGT 22 57.441 300

chr3 10463429 CGTCAAGCTGGAGTTAGACAAAAC 24 60.319 TTGCTTGTTGTAGTTTGCTTCCTC 24 60.202 296

chr3 10463810 GCATATAATCGCCTCTTCCAAACT 24 59.243 ACTGACAGACTAAGAAATCCGTGT 24 59.719 300

chr3 10464960 GCGGGATTTGATTGATTTAGGTGT 24 59.902 ACCACCGTCTTCTTCAATCTTCAT 24 60.263 173

chr3 10466195 TAAACACATCACAGTTCAGTACGC 24 59.554 AGAGGTTTGTGGTGGTGATTACTT 24 60.141 162

chr3 10471175 GGTAGCTGCAAAAGTTAACGAA 22 57.523 CTCTTCTTCAAGCTGTTAACCGTC 24 59.849 290

chr3 10476189 TCAGTCGACCAATGCATTAACA 22 58.33 GTTGCGTTTCCTTCAAATCTTTCTC 25 59.6 267

chr3 10476403 AGAGAGAGAGTGCATGAACTTTGA 24 59.717 GTTGGAAATAACATGCTTTGCC 22 57.073 269

chr3 10476687 GCATGTTATTTCCAACTGTTCCCA 24 60.022 GGAGAGAAAAGAAGAAAAGGTTTGG 25 58.492 277

chr3 10478403 TCTTCTACCTCCGCCAATACAAAA 24 60.02 ATCAATCCCACCTCCATTACTACG 24 59.96 181

chr3 10489383 GGTCGGTTGGATATAAGGAGGAAT 24 59.714 ACCACTGAATCGATTATGAACGGT 24 60.383 191

chr3 10491485 GCTGATGGACCTGCAATTTAAAATC 25 59.479 GGGATTGGTTTCTTTGCAAGTGAA 24 60.443 249

chr3 10491738 CCATTGATCCCTCCAAATTGCTG 23 60.181 GTTTTGGTTGACGACTTTTGTG 22 57.184 85

chr3 10496559 GCGTCTCCAAATTTCCATCATCAT 24 59.963 TATTTGTGTGCTTTGCCTCTCTTC 24 59.784 197

chr3 10510906 TGCGAATCCTTATCAACAACCAAG 24 59.844 CACACATTCGTTGCTTCACTAAAC 24 59.329 249

chr3 10513391 TCGTCTCAAACAGCCTCTTAGAAA 24 59.961 AACATCTAGAAAGCTTGGTCAACC 24 59.238 219

chr3 10526805 TAAATAAGGTTGATTGGGCACTGC 24 59.84 GCTCAACCTGGCTAGTAGTATTGT 24 60.142 206

chr3 10529318 TTGGAAGTTTCTCTAGCCTTGTGT 24 60.141 ACCCTTATACCCCTTAACCAACAC 24 60.018 149

chr3 10534959 GAAACCGGCACACATTACTGATAG 24 59.966 TGGGAAATAGGCCAATTCTTCT 22 57.392 277

chr3 10722206 AGTCATTCATTACGTGGGGAAGTT 24 60.263 GAATACAACAAGCCTAACATGCCT 24 59.601 168

chr3 10844457 CTCGCACAATTAGAGTCGTCAAAA 24 59.85 CGTCACACAAACTCGAATACTTGA 24 59.557 190

chr3 10850605 TTAGAGTCAGCTGAAGTTGTCCTG 24 60.262 TTGCTTCGATAGGAACATGGATCT 24 59.898 145

chr3 10860570 AGAAGACTTGGGAAAGGAAAAGGA 24 59.832 ACGGGTTGATATGGACACTTTT 22 57.971 169

chr3 10862927 CTGTTCCAGGTATACTCCAGTTGT 24 59.777 AGATTAACAACAGCCCATCAGAGA 24 59.774 298

chr3 10865325 TCTTTAAGAGAGGCCCTAGACTAG 24 58.303 TGATTGCTTGGGATAGGGCTTTAT 24 60.141 300

chr3 10870248 AACTCAAACCACACATTTTCTCCC 24 59.901 CACAAAGGAGCAAAAGAAAACCCT 24 60.383 136

chr3 10872178 AGCTCACCATCTACTCACCAAATT 24 60.019 CACACGTGGGAATCTTTGTCTTTT 24 60.202 226

chr3 10875411 TGAGAGAGAGAGGTTTTGATTGGA 24 59.159 TTTTCTGTGGGCTGTTCTGC 20 58.968 216

chr3 10876168 TGGAGACGTGGAGTTGATATGAAA 24 59.778 GTGCCCATTTCTCTCTTTTCCAAA 24 59.961 166

chr3 10876339 TTTGGAAAAGAGAGAAATGGGCAC 24 59.961 TGACAATCTTAGTAAGCAGGTACAC 25 58.604 300

chr3 10878572 CAAACTCTGTCCCCATCTTCTCTT 24 60.263 AAGAATTGGGAATTAGGGTTGGGA 24 59.953 127

chr3 10953885 CCATCTACTTTCATTGATAACGAGGG 26 59.575 CGTGAAGTCAAGGTAAAACTGTTGT 25 60.164 149

chr3 11357539 GTCAAAAGTTCAATCTCACGCTCT 24 59.788 CGACCAAAATGATATTGTGCTCGA 24 59.966 182

chr3 11358759 AACCTTGCGAGCTTAAATTTGTCG 24 60.847 ACGAAGATGCATACTGGTGTTAATG 25 59.706 299

chr3 11359653 ATTTAGGGTCAACGGGTTAGGTTC 24 60.566 GGAAGTGTGTATAATGGGTTAGTTGAG 27 59.72 168

chr3 11397920 GGGTATCAATGCAGTCGATTTCAA 24 59.665 TCCACACATTACATCTTCCATCGT 24 60.081 272

chr3 11398206 AAAGGATAAGCATTGACGGTTGAC 24 59.844 TGGAGGTAGGCTAATGTTGACTTT 24 59.711 190

chr3 11506155 GGACAGAGACTTTTGCACTTTTGA 24 59.904 AGGGTTATACAAGAGCTCACAAGA 24 59.222 124

chr3 11578683 AGAGATGAGAGATTGAAGGGCATC 24 59.959 AATGCTACTGTTCCTCCGATGAAT 24 60.142 277

chr3 11597884 GGGAAAGGAAAGATATGGAATGCA 24 59.105 CATGACTTAAAACCGGCTTGACAA 24 60.261 267

chr3 11623717 GTTCTCGGCTTCAATGCTAAGATC 24 60.025 AACCAATGCCAATAAAGGAAAGGG 24 60.019 297

chr3 11627336 AGAACTCACTTCACGGAACCAATA 24 59.96 ATAGTCTCTTCCACTCCGTACAGA 24 60.08 235

chr3 11628887 AGAAGCTATGTATGTTCAGACGCT 24 59.901 CCAAAACCCTCGAGATCATCCTAA 24 60.142 257

chr3 11632497 GCGTGTCTTCTAGTGCTTTCTTTT 24 60.025 TCCTTGTGAGTGGTTCTTAATCGT 24 59.96 248

chr3 11635330 TAAAAGGTCAAGTGGTGGAGAAGT 24 59.835 GTGCATGAATGTGAAAGAGCAGAT 24 60.142 272

chr3 11635724 CCTTTTCTAGACTTTCCAATTTCCACG 27 60.785 AGCACCATTGAAGGAGAGGTAAAT 24 60.018 208

chr3 11641605 TGGAAGACTCGTTGGTAGAATTTG 24 59.005 GTTGGAGTTGGATTGAGTTGGATT 24 59.476 267

chr3 11650734 TAAAACGTCACAGTCAGTTGGTTG 24 59.906 GAGTCGCAGGAATTCATCTCAGTA 24 60.202 207

chr3 11651460 TGAAGGGAGTGAATTGGAAATTGC 24 60.021 TACTCAACCGGATTTACGCTAACA 24 60.082 222

chr3 11654293 GATCTCGAAACCTACGGCGTTAAG 24 61.538 CCCATCGAATCCAAACCCTTATTC 24 59.722 173

chr3 11683422 TCCTCTTCATCTTCAAACCCCTTC 24 60.264 GGACATAGCGATTGGAATTGAAGG 24 60.024 192

chr3 11711965 TGGCGTGGTTAGGTTGAAAATTAG 24 59.783 TCTAACATCGGGCGTAGTTAACAA 24 60.082 179

chr3 11713900 ATCTATCTTGCTTGCAAACAACCC 24 60.082 TACTCTCACATGACACACACACAA 24 60.142 209

chr3 11719726 GCAAAGTGAAAGAGATCGTGAACT 24 59.788 TTCATTCACCATCTTTACGTCCCT 24 60.02 264

chr3 11728276 GGGTAGCAAATCCAAGTCTAAACG 24 59.906 AGCTAACCATGTAGATGTAGGCAG 24 59.96 183

chr3 11729388 CTTAAAGTACAGTGGCCAATATCAC 25 57.984 ACTCGTTGATTGTTGCAATTACGC 24 61.134 250

chr3 11730477 TACCGATGCCTTGACAAACTCTT 23 60.244 CACGTTTTAGACAACAAAGTTGGC 24 59.736 300

chr3 11735420 ATGCCCTACTGAAGTAAATGCTGA 24 60.08 GTGTCACACTTCCATCCTGAAAAG 24 60.024 173

chr3 11738192 TACATTCCCAATAAGCCACTCCTC 24 60.141 TGGTCTGGTGTGTTATTGACTTCT 24 59.897 262

chr3 11748373 CTCTCACCACAAATGTCCACAAAA 24 59.902 GTCTTGCTTGGTCAGGTTAACATC 24 60.083 300

chr3 11751467 CCTTAAGGTGTCGTGACTATTGAT 24 58.339 ACCCTCCCGTATTCTTACATTTGT 24 59.773 271

chr3 11758078 AAACTCGACCTAAAGACTACTCGG 24 59.844 CTTAGCATGCTCCAACTTGTTCAT 24 59.843 295

chr3 11761131 TTCTTTTGCCAGCTTCCTCATTTC 24 60.262 TAAACAGCTATCACTCACTCGGTC 24 60.142 283

chr3 11764183 CCCAAACGTGCTAAGAGGTAATTC 24 59.906 GGTAGTTCTGAGTTGCACATGGAT 24 60.865 173

chr3 11777265 CGAAAAGCATAGGGAGTCAGAAAC 24 59.906 TACGGTCTCCTCATTGTTGAACTT 24 59.96 183

chr3 11785233 ACCAAGCGATCTTAGAAACATTCAG 25 59.647 GCACTATCTGAATTAGCTTGTCGA 24 58.956 268

chr3 11787652 CTTTTACTGTAACACGTGACCCAG 24 59.789 CTTGTCACGATTGGTAAAAGGAGA 24 59.005 289

chr3 11790251 CAGCTCCAAAACCGTTGTCATAAA 24 60.261 CATGAAAGGACAAAGTTCTGCCAT 24 60.022 128

chr3 11790502 ATGGCAGAACTTTGTCCTTTCATG 24 60.022 TTAGGGAGAGATGCTTGGAGATTG 24 59.898 297

chr3 11805397 AATTATGGGTTGCCGACATACACT 24 60.628 CTTCAAGGGTATTGCATTTGGTGT 24 60.021 154

chr3 11808464 CTTAACGGTCTGAGCAACAAGAAC 24 60.319 TGAAGTAGTGGGAATATGATGCGA 24 59.656 269

chr3 11813327 GAGGCTTTTGTACATAGAGAGGGA 24 59.594 CTCCAAAAGAATGAGTGGCTGGTA 24 60.807 135

chr3 11814558 CACAGGTTGGCAGCAGTTAAAAG 23 60.797 AGAGGTAAAGAGAGACAAAGATGG 24 57.707 282

chr3 11824183 CGTGATACTCATGCTTTTCCATCC 24 60.025 TTTTCACCTGTGTTGTTGAATCCC 24 60.142 201

chr3 11881526 GCTCTCACTGTTGACAATTCCAAA 24 59.963 CAATGAAGGTGTTACGGTGAAAGA 24 59.486 290

chr3 11882466 CTAATGTTGCTTTGCTTCCTTCCA 24 60.022 TAGTGCGTTCTAACCAAATCCAGT 24 60.263 244

chr3 11886744 CTTGTGCCACTTGAAAGAGAAGAC 24 60.261 GAAGGCGGATGTTTGGAATTGTAA 24 60.082 245

chr3 11897027 AATCACAACCCTTCAATTTCCACC 24 59.96 TCACAGGCTATGAGAAGATGTTGA 24 59.533 194

chr3 11901007 TAATCCCATTCCCATCTTAGCCAG 24 59.957 GTAGTTGCTCACCATGGATTGAAC 24 60.143 137

chr3 11909028 CCGTTCCCACTCTCAAATCTTAAA 24 58.999 AATAAACTCTCGTAATCCTCGCCC 24 60.501 249

chr3 11922761 CGCCATGATACCGTTACGAAATTT 24 60.26 CTTAGTCTCTCATCCACACGTGAA 24 60.083 190

chr3 11926538 ACTAGTCTCACGAATTGGTTCCAT 24 59.777 AATGTCAAAGATGGAAGGTGGGTA 24 59.956 234

chr3 11934644 CACTTTCCTATGATCTAAACAACCG 25 57.995 TGACAATTCTTCTCGGCGTATAAG 24 58.959 292

chr3 11940611 GTGCAAATTTGTTCACGTCAACTAC 25 60.053 GCCATATTCTCTCTCCATTTATGTG 25 57.625 285

chr3 11940851 CACATAAATGGAGAGAGAATATGGC 25 57.625 GCTCTCAAAACTTCCATCACATGT 24 59.783 294

chr3 11951009 TCATCTATTGTGCTCTCCTATCCA 24 58.603 CACTCTCATCATTTGTTGTCCACA 24 59.484 80

chr3 11953729 CTAGTCGCTATACATGTCACACAA 24 58.421 GCATGATCAACTTCAACTTCCTAGA 25 59.123 253

chr3 11964435 ATGAACATGACTCGTGCGCTCAAC 24 64.161 TTGGACCAATCACAAATTTGCACG 24 61.267 295

chr3 11969885 AACCCTCGTAATTATGGCATGGAT 24 60.203 GAGTCCACGATCCAACCCAATC 22 61.001 291

chr3 11977090 CAACTAAGAAAAGATTGGGGCGAA 24 59.782 GGTTTGAAGAGTTGTTTGGTGAGT 24 59.842 289

chr3 11991959 CAGTTACCTTATGCAGTACAACAGA 25 58.837 AAGACAAATTGATCAGACTCCCCT 24 59.709 121

chr3 11994298 CTTCCAAAAGGAGAATGTACGTCT 24 58.761 TGTCACTATGAGGAACAGTAACCC 24 59.778 175

chr3 11995189 GTTCACTATTCCACCATCAAACCA 24 59.235 CTATCTGCCACCATGAACAAAGTC 24 59.905 299

chr3 11997254 GCGTTCAAGCGAGGTTTAACATAT 24 60.202 GGTCATTCTCTTCAAGCAAAAGCT 24 60.023 89

chr3 12006804 CTTGTCCATAGGTACATACAGCCA 24 59.899 TCACAGAGCATGAAATTCAAACCC 24 60.022 257

chr3 12022369 AAAAGCACACAGAAAAGGAGATCC 24 59.721 ACATACGCCTACATTTGGGAGATT 24 60.141 295

chr3 12030525 GCCATTGTCACATGTCACATGTTA 24 60.083 GTCATTGCTCGTCACTATCTCTCT 24 59.964 239

chr3 12032942 ACAGCACAACCTAGAGAACCATAG 24 60.081 GGGGTTTTGTTCATAATACAGCCT 24 59.29 207

chr3 12035326 TTTTCCCCTCGTACAACCCTAAAT 24 59.957 CCTTTCATTTCGGTTGACACTTGA 24 59.964 119

chr3 12037012 AGCCAACCCAATAATCAAATCTGC 24 60.142 GCCTTTGGTGAGTCTTTTGGATAC 24 60.083 213

chr3 12048184 AATCAAATGCTCCCACCAAATCTG 24 60.081 GGTCGGTTTGCAAAATTTGTGGTA 24 60.974 181

chr3 12054189 GTTAGGCCTCCAATTTCAGGGA 22 60.025 CTCATAAGTATCCATCTGAGTTTACCG 27 59.19 282

chr3 12054816 TCAATTGGACCCACTAAATCTCACT 25 59.986 CCCATCTATTTTGTGTGTGTGTGT 24 59.722 213

chr3 12057851 TATTTCCCGCCGCCCTATATAATC 24 60.202 AATTGAGGAAAGGAGGGAAGAGAC 24 60.019 178

chr3 12081301 GCCAATAGTTTGCCTTCCATTTTAG 25 59.188 GAACTTTCAATTTCCCCGCCC 21 60.067 258

chr3 12085149 CGATAACAAGCATCAAGTACCCAA 24 59.366 TGGTTGTTTTCCGTTCTTCACT 22 58.651 300

chr3 12088868 GTTTCAACACACGAATCTTCCTAG 24 58.083 GCAGCTACATGACATCAGATGAAC 24 60.025 237

chr3 12100035 GATTTACACATGACCGGGCAAATT 24 60.382 TGAGGAATATCGTTCTTTGTCACCT 25 60.047 295

chr3 12100698 TTCCGTCTTCTGTTTCATTGTTGC 24 60.497 TTCATTCGCGTAAAGAAACAGAGG 24 59.848 150

chr3 12102506 GTCTTGTCCTATGCTTGAATTGCT 24 59.603 TAGAAGGGTTGATTTGTGAAGGCA 24 60.447 234

chr3 12102984 CTCACATCTTCCTTCCTCCCTATT 24 59.342 TAGGGTTTATGTGTGGAGTTTGGA 24 59.649 296

chr3 12105808 TCAAATTGTGTTGTCCAAGTCGAC 24 60.202 AGTAGAAGTTTGAGGAGGTTCACC 24 59.959 199

chr3 12106117 GGTGAACCTCCTCAAACTTCTACT 24 59.959 TGTCGTGTCAATTAGCATATAGAGG 25 58.553 287

chr3 12107130 TTTTCAAACTAGTCAGGGCCACC 23 61.001 GAATTCGTGCGTTTCGTTCTTAAG 24 59.455 278

chr3 12112590 TTATGAACTCTGTGCTGCTTTGTG 24 60.024 CGCTTCTTTCTCTTCATCACTTCC 24 59.907 182

chr3 12115810 TGGTGTGTGATCGAGATTTCAA 22 57.998 GAGTGAGGTAAAAGAGCGTAGACA 24 60.083 174

chr3 12185931 AACTTGGACCTTTTCTTAGCCCAA 24 60.633 GATCCCCTCATAACCTCATTCACA 24 59.897 256

chr3 12187879 ACATTCAAGCTACAACTGGTCTCT 24 59.959 AGACATCTCCTAAGCACCATCATC 24 59.96 155

chr3 12187980 GATGATGGTGCTTAGGAGATGTCT 24 59.96 CAAGTGTTTGGAAGCAATTTGGTG 24 59.966 242

chr3 12189425 ATCTTTCGTATGCCACCACCATTA 24 60.385 CTTCCTCAACACCCTCAAAATGG 23 59.743 283

chr3 12192196 TCCTCTCTCTACATGCGTTAGAAA 24 59.052 GTACCTCCTACCCTCCCTAGTTAA 24 59.831 299

chr3 12193801 TCTGACCTTATCTTCTCCTTCCAA 24 58.658 TTCCAATTTCTTGTACTGACCGGT 24 60.445 278

chr3 12199948 TTGTTACGGATGTACATGCTCTCT 24 59.84 ATCACCACATGCATCACTCTACAT 24 60.142 145

chr3 12216119 GCAGATGAAGCATTAAATCGGTGA 24 59.964 TATAGTCTTCCACCTCTTGCTTCG 24 59.902 200

chr3 12222721 TATCTCTTTCAGCTTCACCCTCAC 24 60.081 TGTCACTCAAAATGATCCACTCCT 24 59.958 254

chr3 12232245 AGAGCTCCCCAATCCCTTTAATTT 24 60.016 CTAGCAATGACGCATGACAAGATT 24 59.965 283

chr3 12232971 TGCATCACCCTTACCACAATAGAA 24 60.019 CAAACATATTGACGAGTGCTCAGG 24 60.202 230

chr3 12238260 TCCTCTCACACCTAAATAGTAACAC 25 57.833 GGAGGTGTGTAATGGTTGACTAGT 24 60.02 224

chr3 12251154 TCAACCAGCCAACCAATAAACAAC 24 60.442 TCATGTCTTTAGTCACACTTGGGG 24 60.505 268

chr3 12259310 GCGTGACTTTCAAACCCATAATCA 24 60.083 TCATGTCTTTAGTCACACTTGGGG 24 60.505 104

chr3 12273487 AAGTGAATTGTAAGGCGAGAATGC 24 60.143 TGAGTCATTCAGTTGATCCCATCC 24 60.385 102

chr3 12274340 CCTACATTTTCACTTCATCACCACC 25 60.106 TTGGGTTCAACTACTTGTGAATGC 24 59.963 278

chr3 12285331 GTCCCTCTCCTACACTTCTAATGG 24 59.657 GTTGTCCACGGCGAATTTAACTTA 24 60.084 286

chr3 12299232 CGTCTAGAACTTCCAAAACATCAGC 25 60.39 CAAGGTCCATTAATGCCAAACGTA 24 59.843 265

chr3 12365961 TTGATGCATAGAGAAAGGGAGAGG 24 59.898 GGGGTTGCTCAATTTGTAACAAAAC 25 59.993 161

chr3 12366528 AATCAATCCACACCATCCAAATCG 24 59.902 CCAGATCAAGAGAGGTTGGATCAT 24 59.897 298

chr3 12368710 GGCGAGACATCAATTCTGGAGAT 23 60.494 AAGTGCACAACCAATTCTGAGAAG 24 59.963 259

chr3 12376470 GCACTCGAACTTCTTCTCATCATC 24 59.733 TCCACTCAATCCATCATGATCGAT 24 59.715 215

chr3 12512030 GAAAATCAAGCATGGAAGGGTCAA 24 60.021 AATTAACCTCGTGTACTTTGGGGA 24 59.958 177

chr3 12516564 CGGTCGATTTAGCTTCATTCGTTT 24 60.202 TCTGTTGGCTTTCTTTACGTTGTC 24 59.965 277

chr3 12522549 GGCGAGCTTAGAGTATTTCATTCG 24 59.851 GGCCCCACTCCTATAAATTATTCT 24 57.859 218

chr3 12530235 TTTTAAAGTCATATGCACCACCGC 24 60.38 AGAGAGAGAGAGAGAGGGAAAACA 24 59.956 290

chr3 12535261 ATTGGTTATGGAGGAGGGATTTGT 24 59.767 GATGCGTTGTGTGTGTGTGCTTTT 24 63.466 208

chr3 12542001 TGCCCCAACGTAAAATTTAGAACC 24 60.022 TCAACTGGATCCAAGCAACTTTTC 24 59.962 212

chr3 12543081 TGCCTTCTATTCTTCCAAATTCCC 24 59.044 CTCCATGAAATATGCCAAAACGAC 24 58.963 261

chr3 12546595 GAAGACCTACTACGAGTACACAGG 24 59.668 GCTTTTCAGTCAACTCCACATCTC 24 60.084 256

chr3 12550326 CTTTTAACTTCTTTGCCTCACGGT 24 59.964 TCAGGACCTATGAATTTTGGCTCA 24 60.019 257

chr3 12552897 GGGAGAAGAGAAGAAGGTTGTTGA 24 60.202 CCCTATCGGTATCCCAAATTCAGT 24 59.958 178

chr3 12554293 TTGTGATCATTCTTGTTGGTGCAC 24 60.5 AGGTTGAAACTTTGAAGACAGCAG 24 59.903 293

chr3 12557688 CGCTTTCAATCAATCCCTCCATTT 24 60.142 CTTTCAAAAGCGTCACCAAATG 22 57.303 157

chr3 12559181 CTCATTTTCCATAAGCGAGAACAAC 25 58.976 CTTCAAACAACTGCCTTTAAAGCTC 25 59.54 160

chr3 12720499 CAAAATCGTACGTATCCCATTGCA 24 59.965 CAAATGTGCAGAATTTTGTCAGCC 24 60.084 273

chr3 12720854 TTAAGATCCAATCAAGGCCCATCA 24 60.079 ATCAATCTCGACTTCATCCATGGG 24 60.505 138

chr3 12721593 GACACTCTCATCTTTTGTTTCAACC 25 58.798 ATTGGAGTCGTTGGTTTCACAA 22 58.716 299

chr3 12722171 GGGTACTAGAGCGGGTAATGTTTT 24 60.383 TTATGTATGCTATTGAGTGCCCGA 24 59.96 273

chr3 12754297 TGTGTAAAGCAGTACTGAAGGTGA 24 59.9 ATGGAAAAGAGAGCAGTTGAGGAT 24 60.019 295

chr3 12755038 AGATCTACCGTTGATTCTTGCACT 24 60.081 CCGACCAAACAACGTCTATCAAAT 24 59.847 263

chr3 12758480 CCCACACACACACTCACTTTTATT 24 59.662 AAATATTATTGATGGGCTGGCTGG 24 59.471 266

chr3 12771092 CCTAAACAGAAAGGGCCTAACAAG 24 59.541 CTTCTGATGGGAAATTTTGGCTGT 24 60.021 277

chr3 12772911 GCAAAGAAGAAACTAGAGGATGAGC 25 59.935 TCCTGAAAACTCGGTCACAATAGT 24 59.96 227

chr3 12773486 GCAACTCTACCTTGAATGTGAAAAG 25 58.854 TGCACGATTCTAAGATATCCCACA 24 59.656 241

chr3 12776391 TAAGAGGGGTAAGAAAGGCAGTTC 24 60.02 ACCAAACAACTACCAAACAAGGAG 24 59.599 219

chr3 12781487 ACCAACCCCACAGATGACAAATC 23 61.067 CGACAGGAATAGGGGTGTATTTTC 24 59.185 269

chr3 12785389 AGTTTTGGGCGTTTAGAGGATTTG 24 60.022 TGATCTCTCGATTCTTTGCTCCAA 24 60.081 229

chr3 12785640 CTTTTACTCACGATTCAGGAAGGC 24 59.906 TAAAAGCACAAACTTCTTCCGGAC 24 59.964 156

chr3 12785991 AAAAGCTTGATTCTTCTCCCACAC 24 59.721 TGAAGAAGACTGGACAGAGTAGTG 24 59.479 270

chr3 12791017 GGTTTTGATCCCTCTCACATGG 22 58.98 AGATGGAGATAGCAATAGGGATGA 24 58.402 140

chr3 12794672 TGCCTGTCTAAAATCCATCCATCA 24 60.08 ATTTCTTCCCTTCTCCAACCCATT 24 60.203 256

chr3 12803660 CATTTCACACCAATTTCCTGACCA 24 59.961 TCAGATATGGTGGAAAGATCAAGGA 25 59.333 250

chr3 12807736 TCAAGACCCCTATGAGCTACAAAC 24 60.081 ATCAAAGCTTGGGAAGAGAGTGAA 24 60.203 300

chr3 12823501 GTAAAGGATGACGACAAGTGCAAG 24 60.377 GACATAGTCCATCTTCAGCTCCTC 24 60.262 248

chr3 12830122 AAAAGTTCTGGGTGCTCTTCTTTG 24 59.901 TTAGAGTTGGACTTGTACTTGGGC 24 60.504 256

chr3 12836762 GGGTCGCTTGTTTAAAGTATGGAG 24 59.906 CCAAGAGGTCGGATGTTGTACTAA 24 60.082 295

chr3 12840490 GCATTTCATCCCGTTACTTACATAC 25 58.344 CTTCTAGGTACACTCAATACAAAGC 25 57.473 290

chr3 12843115 ACCTGCACCAAAGATTCATACTCT 24 60.019 ACTAAACCCCACTTGCCTATCTTT 24 59.956 186

chr3 12843322 TTCCTTGTGTGCTCATGTTTTCAG 24 60.202 GTGTTCAACATATGCAGAGTAAGGA 25 59.125 243

chr3 12845342 ATTAGCAATCCTTGATCACCGCTA 24 60.202 GTCTATCACCGATAAATCATACGAGTC 27 59.307 263

chr3 12855359 ATAAAGTGTTTCGTGCAAGACCTG 24 60.024 CGAGAGAAAGAACCATCACCTTCT 24 60.322 239

chr3 12868344 AAGCAAAGGTACAAACTTGGTGAC 24 60.142 AATTCATGTAGCAGGGCCTATCAT 24 59.956 178

chr3 12874903 ATTTTCCCTCACTTTGCTCAATGG 24 60.021 GGGTTGAAATACACATTACCGTTAG 25 58.222 212

chr3 12888848 GGAGCCAAATCTTTTCCACTTACG 24 60.379 CTACGTACTAAAACCCCTCACTCC 24 60.142 296

chr3 12892681 CCAACGGGCTTTATTTGACACTTT 24 60.501 AGTTGGTGAGTCAGTTGCATATCT 24 60.02 296

chr3 12893677 GCATAACACGAGTAAACAACACCA 24 60.025 ACGAGTCCATAAGTCACTAATGCA 24 59.84 238

chr3 12893982 TGCATTAGTGACTTATGGACTCGT 24 59.84 CAAGAGAAGAAATGTTACGAAGAGG 25 57.945 247

chr3 12894560 GTTGAGCTAAGTTCATGTTGGTCA 24 59.485 TCTCACTCACCACGAAATTCAAAC 24 59.727 283

chr3 12900333 ATTGAGATGGGTCGTACTGTTAGG 24 59.9 ATTTAGATTCAACTCTCGGGCTCT 24 59.593 160

chr3 12901504 ATTATGGTGTGGATGGAGGGAAAA 24 60.017 CCCACCCCTAAGTTCATTCAAAAC 24 60.021 237

chr3 12903772 ATTCTTCTGGGAAGCACAAACATG 24 60.022 GTATCAGTCCTGTGGTTCCTCTTT 24 60.02 98

chr3 12905539 ATTAGTGTTGGGGTGTCAAGCTAT 24 60.018 GGTGCAATAACTATTCCCTCTCCT 24 59.897 262

chr3 12907754 ATCCAATGATCATCGATGCTAGCA 24 60.323 TCAGAGTGGCAAAGTAATAACATGG 25 59.352 254

chr3 12908660 TTATGAAAATACCTCTGCGTGGGA 24 60.081 TAATTCTCAAGACCCAACCCGAAA 24 60.202 275

chr3 12910515 AAGGTTACAGATGCAAGCCAAAAG 24 60.262 TTCAATGACAACTTGCTGTGTACC 24 59.964 219

chr3 12912177 CGTCCCCTTTCTTCTCTCCTTTAA 24 60.02 CCGAACAAGAAGTGAAGGGAATTT 24 59.722 293

chr3 12917950 TACCATCAAGTGAGAACATTGGGT 24 59.957 CTGCATTGTCGTTTCCTCTTCAAT 24 60.083 208

chr3 12925244 CAGAGACCCCTTTCCTCCTTAATG 24 60.385 GGTCAAAGATCTAGCAATGAAGGC 24 59.964 138

chr3 12942634 TCTTCAATCCCTCCATGGTTTT 22 57.597 GGCTTTTCTTTTCTCCCAACTTTTG 25 59.706 199

chr3 12954281 TGTGTTGGTGATGAAAGAAGGTTG 24 59.902 CTCGTGATACTTACTGTGCATGGA 24 60.44 260

chr3 12964243 TCACGTTTGAGTCCCTACATATGT 24 59.535 TATCACTGATAGACCACGTTGCAA 24 60.082 215

chr3 12989910 GAATATAGGAGTTGGGAAGGTGGG 24 60.202 CAGATAGGCCCAACTCAAGTCTAA 24 59.838 290

chr3 13025607 CGGTGAGGCGAGGTTTTATATAGA 24 60.023 GAGGTAAATGCATCTGGTTTTCGT 24 59.844 257

chr3 13062303 ACGTTTAGCTAGATGGTTCAACAG 24 59.07 GCTTACTTTGCCAGCTAGTATCTA 24 58.156 220

chr3 13072893 AGTTCATCTATGCATCCCTTCTCA 24 59.342 TAAGTGCATCCCAAACATCACATG 24 59.842 284

chr3 13075066 GCATATGGTTATTGTATTGCATGGC 25 59.422 AAAACAAGAGAGGGCCAGAATACT 24 59.957 180

chr3 13098224 AGCGTGTTACTCTCCTGTTTTACA 24 60.202 TTAGCCAGAGTTTACACATGAGCT 24 60.02 256

chr3 13099687 TGATGTTCATCCTTGTATGTGGCT 24 60.324 AGCCTTCAGTTGAGAGGTATGTTT 24 59.958 287

chr3 13113043 CACGAGGGGCAATGACTACAG 21 60.74 GTGTCATACAACCGTGAATTTGAGT 25 60.05 203

chr3 13116433 ATCCAACCCAATTCACTTACCCAT 24 60.265 GCCTCTTTGTCTCTTTGTTAACCA 24 59.421 92

chr3 13116965 GTGAACCACGGTAACTTTTGATGT 24 59.964 CTTTGTGATGATCTCGACAGTTGG 24 59.907 300

chr3 13123197 CTTCATAACTCAACCCTTCCTCCA 24 60.019 GTGGTTAACATGATCGGGAAAATG 24 58.66 287

chr3 13131545 GGCATCAATTCAACAGACCTATGG 24 59.963 AATAGGAAGGAAAGGCATAGTGGG 24 60.141 186

chr3 13134387 TCGAGAACACAAAAGCAATCATGG 24 60.32 AATCTGGATCGTGACACTTCTGAT 24 59.839 298

chr3 13137597 TACAGTTTCGATATGTGGTGGGAG 24 60.142 CCATCAGTATCAATTCAGATCTCACG 26 59.634 300

chr3 13142067 CTTCTAGGGTTTGGGTTTGGTTTT 24 59.592 ACACCAAAATTTACCCGATTAGCC 24 59.841 191

chr3 13143045 CATTGGCGGCATAAGTACTCATTC 24 60.32 TCTCAGTACACAGTTCACAGTTGA 24 59.599 175

chr3 13143153 CATTGGCGGCATAAGTACTCATTC 24 60.32 TTTAGTTGTGTTGCCTGATTTGCG 24 61.026 220

chr3 13143302 TCAACTGTGAACTGTGTACTGAGA 24 59.599 ACTCTGGGTATACTTTGGTTGACT 24 59.155 259

chr3 13143517 CACTGACAGTTTTCAAAACCAACC 24 59.375 CCCAAATGATACGTCTCCAAAAGG 24 59.904 288

chr3 13144339 AAGGAGCAGTCACGAGCAATATAT 24 59.96 ACGAATCTTTGGCTCTTTCTTTGG 24 60.023 262

chr3 13145284 GAAGAACGCATTGTCTCCAGTTTT 24 60.261 TGATGATGATGATGGTGATGGTGA 24 59.897 265

chr3 13148617 AGGAAAGTCTATATGAGCCCCTCT 24 60.141 ACAAAGTGTGATCATAGTGCTCCT 24 60.02 129

chr3 13152289 GATTTCTGTTTTCTTCTCCGGCAA 24 60.023 TATCCAGTCAATACACGTTCAGCC 24 60.68 177

chr3 13179663 TGTCCTTGTTCTAGTGTAGTGAAAC 25 58.722 GCCTTTATCCAAAAGAATTCGGCT 24 60.142 293

chr3 13216502 AATTCTCACTTTCCTCTCCACTCC 24 60.02 GTTGAATTGAAAACACGAAGGGGA 24 60.202 216

chr3 13218861 CGTAAGGAGATAGGTATAGTCGAAA 25 57.336 GATGAATGCGTACTTGAGGACTTG 24 59.967 194

chr3 13222207 TTGAGGTCAAGTTGAATGTTGCAG 24 60.202 TTCTGGATCTCTTCTCTTTTGGGG 24 60.019 293

chr3 13226148 TGATCAGTATACACCACAAAGCGA 24 60.082 CTCAAGAAAGCCATGGTTACACTG 24 60.083 263

chr3 13226497 GCCCAAATATTCAATCCGGTCTTT 24 59.901 ATGTATAGCAAGGATGTGGAGACC 24 59.959 129

chr3 13227713 AGCTCAACATTCTCAGGCTTAAGA 24 60.02 AGAGAGGGGAGAGAGAGAACTTAC 24 60.08 147

chr3 13228994 AGTTTTAATCACTCTCTAGGCGCT 24 59.839 AGCACTAAGATCTACTGCACACAT 24 59.839 195

chr3 13244617 ATTCATCTTCTATGCGAGCCTCAT 24 60.02 TTCACAAACACAATCATGGACTCC 24 59.722 200

chr3 13245750 TAGTGAAGTGTAAACATAGCGGGG 24 60.382 AATAGAGAGAGGGGAGGAGAACAT 24 59.828 189

chr3 13248616 GATGCATGAAAAGGAGGGAGACTT 24 60.87 CTGGTGAGTTCTCATTCAATGTGG 24 59.845 167

chr3 13248970 AATGTGTTCGAGACTTTTGGTAGC 24 59.787 GATGTGCAGAAGAAGACAAAAGCA 24 60.261 287

chr3 13255368 TTCTTTTGTTTCCAACTCTACCGC 24 59.964 ATTGTAGCTATGCATGATGGACCT 24 59.957 223

chr3 13270319 CGTTGTCGGATTATTTAAACACCCT 25 59.877 CGACGTTTCAACTTATGCTCCAAT 24 60.143 277

chr3 13274564 AGCTCCCATTTCTTCTCCTTTCTT 24 59.956 TCTCAATCTCTCTCGACGTATAGC 24 59.313 261

chr3 13275205 GCTTTCGATTCCACATATTCTTCTC 25 58.346 CGTAAGGTTTGGTTTGAGTTGCA 23 60.181 297

chr3 13291401 AGCACTAAAACAACCCAGGAAGTA 24 60.141 CTTCCATTGGATAGTTAGGGGCTA 24 59.402 213

chr3 13298756 CGACGACCATTCAATGTCAGATTT 24 59.907 GGTTGGGTTATTGTCAGTTGTGTT 24 59.901 215

chr3 13301799 AATCCACCTTCCTCGTGTTGATAA 24 60.02 TCTCATCACAATCACAATCCCAGA 24 59.774 152

chr3 13304304 GCAAGGAAGATTTGTGAACTGCTT 24 60.5 CAACCGATGAATTAGATTTGCCTGA 25 59.934 215

chr3 13307489 ACACAAGTTCCTTCTGATGACAATC 25 59.529 GGGCTTTTATTCAACATACAACCTC 25 58.67 287

chr3 13307757 GAGGTTGTATGTTGAATAAAAGCCC 25 58.67 TGAGGCAAGCTAAGTTAGAGTTGA 24 59.717 196

chr3 13310885 AAGGTTGATTGGTAGCGACAGTTA 24 60.263 TATTGCTGGCTAATCTCCGACTAC 24 60.022 264

chr3 13311023 TTGGTGTCAAATAGAGGTTGGTTG 24 59.417 GGGGCAAGATTTAGTTTTAGTTGAGG 26 60.35 247

chr3 13314482 TAAGAAGATGGTGAAGGAAGGAGG 24 59.529 CTCGTAAAGGTTCAGAGTCTTGGA 24 60.022 292

chr3 13320450 CCGGAGAAAAGAGAAGTGGAAGAT 24 60.323 GCTAGACGTCTATGGCAATACACT 24 60.262 226

chr3 13322508 AGAGTCTCAACATAGAAGCTTTGGA 25 59.753 CTCACAACCAATTAGTCCTAGAAAC 25 57.634 178

chr3 13337669 TTAAGTCCCTGGAAAGAGATCATC 24 57.759 GCTGCCAACTTTGCTCTTAAATCT 24 60.322 300

chr3 13338340 CCTACGCAATTTATCCAGCCATTT 24 59.962 CCGTCTTCAACTCACAACATTAGG 24 59.848 149

chr3 13338991 TGGAAGAGACAATTTGAACCCTGA 24 60.141 ATCGTATGAACTGTGCGGATTTTC 24 59.966 254

chr3 13341222 GGTGACACAGATTCGACATCAATG 24 60.202 GAATTCCAATGGATGTTGATCGCT 24 59.963 286

chr3 13344935 TACAGATATTAGCCAAGTCCTCGG 24 59.477 AAGATGTTAGTTGGACGTTTGGTG 24 59.725 268

chr3 13348117 CCACTTTACTCCCCACCGTAATTA 24 60.081 ACCTTGAAGTCGGTCATTTCTGTA 24 59.96 273

chr3 13365289 CTAAGCCAAACATGACATCAAGCA 24 60.083 ATATTTCTCTGTCAATGGCGCTTG 24 59.964 232

chr3 13366926 TGCATAAGACCAGACCACGAAATA 24 60.081 ACCCTAATGTCGAGAATGAGTGAA 24 59.534 265

chr3 13367575 TTGTAGCTTATGATCTGAGGTCCC 24 59.655 GGAACGTTGAAAGCAGTTTTGT 22 58.492 295

chr3 13370195 CTATGAGCTTAAGAGTTACGGGCT 24 59.962 GGTAGACTTTACTTTAGTTTCCCAC 25 57.336 268

chr3 13370875 GCCAGGGGAGAAAATAAAAGTCAC 24 60.082 ATGAAGCACAGTGAGTCTATTGGT 24 60.02 191

chr3 13472145 ACCAATGTATCATGTTCGAGCGTA 24 60.441 ACAAAGCAGATGAAAAGGGGTCTA 24 60.203 253

chr3 13476704 GCCGCAAGTTCACGATTTCTATAA 24 59.966 TGACGACATATCAACCTCAAGTCT 24 59.537 233

chr3 13478480 CGAAGTGGTGATACAGAAAAGCTC 24 59.908 GTCGAGACTACCACTAGAGTTGAC 24 59.907 296

chr3 13480452 CAACCCACCCTCTAGATTGAAGAA 24 60.019 CAAGTTTCTGTTGTCGTCGTTGTA 24 59.968 256

chr3 13488425 GGAGTCTCACAAACTTTAGGAGGA 24 59.716 AGAGGGAGATTTAAGGAGGGAGAT 24 59.826 294

chr3 13495846 AATACTCTCTCTCTCTCCCTCTCC 24 59.649 TGGTTGCTCTATGGTTACGATTGA 24 60.081 205

chr3 13501898 AGACGCGTTTTCTTTGTAGAGTTG 24 60.026 AACACCATGCAATTTAGTTCCTCC 24 59.779 116

chr3 13506636 TTAAAATGCGGCAGATGTGTGATG 24 60.675 ATGCGCTCCTCATGTGATTCA 21 60.134 289

chr3 13510934 AGTGGAATTTTACGCGCATGTT 22 59.772 TTCTTCCATCCTTCACCCTTCAAT 24 59.955 242

chr3 13511235 TTGAAGGGTGAAGGATGGAAGAAT 24 59.955 AATGAAACGTGGCACCATTCTCTT 24 61.528 205

chr3 13518408 GAGGGAGTCTGTCTTTGTTTTATGT 25 59.002 AAAACAAAGAAGCCCCACAAACTC 24 60.623 273

chr3 13531939 TCCTAACTTGGAGATGAAAGGCTA 24 58.97 GGGCTGTGAGAGACTTAAATGGTA 24 60.081 257

chr3 13532205 CAAACGATATCCACCATCCTTTCG 24 60.025 CTTCAAATAAAAGTGACCGGGCTT 24 60.022 233

chr3 13540132 ACTTCTATGACTCATTGCAGCAGA 24 60.081 ACTTACCATTTATGAGGGGCTTGT 24 60.017 247

chr3 13543219 TTGGCCTTCACTATCAATTCTCCA 24 60.019 GGTATGAGTGTTGCAGTTGTCATC 24 60.143 254

chr3 13544765 GCCCAACAATCCAATCATATCCAT 24 59.471 TTTGAGTCTTGAACGCTCTTCTTG 24 59.729 129

chr3 13551946 CCTCTAAAATTGGGTATTGTTCCTTCC 27 60.148 CCCACCCGAGCTATAAGTATGTAC 24 60.082 138

chr3 13556859 GTAAACAACCTCTTCTTGCGCATA 24 59.847 GCTTACTGTGGGAATTGTCTATGC 24 59.964 230

chr3 13557234 CCTTCAATCAGGGACCCAATAGTA 24 59.588 TGTTGAAGTGCATTTGGAGAACTC 24 59.963 221

chr3 13557438 GAGTTCTCCAAATGCACTTCAACA 24 59.963 TGGTGAGTTAGGTGAGTGTTTTGA 24 60.081 139

chr3 13557711 TCAAAACACTCACCTAACTCACCA 24 60.081 GGGGTCGGATCTTTACAAAAGTAT 24 58.566 280

chr3 13557843 GGAGAAGAAGATGAATAGGTTTTGTCC 27 59.934 GCTCGAACTAGGACTAAGCTTTCT 24 60.142 288

chr3 13561086 AACTCATGAAATAAGTGGGCAAGG 24 59.536 CAAGAATAAATTGACTACCTTCGGC 25 58.513 252

chr3 13561739 GGTGATAGTTGGAGTTGACTGACT 24 60.021 CATTGTCGGCTAACTCTTCTTGAC 24 59.908 291

chr3 13567743 CAATCTCAACCCAAAGCTTGAACT 24 59.962 ACATCGTAGTAGTCTAAACGGAAGA 25 59.123 165

chr3 13600354 CTTTCTTTCTTGGAGATGAGGGGA 24 60.019 AACCAGCTGTCTCCCTCAATATAC 24 59.898 298

chr3 13602393 ATCAAGAGGTCACTAGTTCGAACC 24 60.082 TAAGTCGGAGGAGTAGATCATTGC 24 59.721 125

chr3 13602937 CGTCGACAAAGGCCCAAATAATAT 24 59.725 GTCACGTGTAAGAACCTCAACAAA 24 59.669 245

chr3 13605564 ACACTCCAAACTTCGAAAGCAGAA 24 61.161 ACATGTCGGATGATGGGGAAATAT 24 59.957 217

chr3 13610556 TAGGCTATGCAGATTTCGAGTAGG 24 59.781 TCCCCTAACATCGAGATAAGCTTC 24 59.719 249

chr3 13613344 CACCACCAAGATAAAACAGCCAAA 24 60.202 AAGCATTGAAAGGACCATTGATCG 24 60.142 281

chr3 13618714 CGTGTCTTGGTTTTGTCACTTAGG 24 60.261 TCACAAGTTATCTCCATCTTCTCCA 25 59.279 279

chr3 13620928 GTGTGTGTGCATGTGGAATTAACA 24 60.499 AGTGTATCCATCCCTAGCTAACAG 24 59.164 300

chr3 13623264 TCTCGTGGCATTAGATTTCATTGC 24 59.964 CTACTACTTCGGCATTTTCGTCAC 24 59.969 244

chr3 13623473 GTGACGAAAATGCCGAAGTAGTAG 24 59.969 AGCATTGGTTGTTTGGTCAAAGTG 24 60.919 199

chr3 13627474 ACTTGGCAAAAGAGTGTGGTAAAC 24 60.142 CGCATTATAATCATTGGGCCAAGT 24 60.022 282

chr3 13634384 GGCAAACAAGTGAAGAAAAGAACC 24 59.433 GCTTTTGACGAAGATTGCTCGATA 24 59.967 194

chr3 13646094 ATATTGGATCTGGTGTTGGAGGAG 24 59.896 CTTGTACCTCTTCGATTAGCTCCA 24 59.902 297

chr3 13668299 ACACAAGATTCACCAAAGACCTCT 24 60.141 CCTCCAGCCTACCTAGTTTCTTTT 24 60.019 282

chr3 13668545 GTGTTGCCATTTGAGGGTACAATT 24 60.262 GGGAGTTTTGAGAAATGAGGTTTAGG 26 60.072 232

chr3 13669791 GATCAAATCCCCTCCCTCATTT 22 57.81 GAGATGTACACTCCAATAACTTAGC 25 57.298 298

chr3 13670319 ATTTGCATGCCAGATAAGGGTTTG 24 60.383 AATTTATGAAAAGAGGGGTTGGCC 24 59.775 265

chr3 13681811 TGTTGGTGAGTGGGGTTTATAGAG 24 60.02 CCATAAAACGTCTCTCACACACAC 24 60.085 200

chr3 13682408 TGGGGCTCAAATTTCTCAGTTTTG 24 60.202 GTTTGGCCATCATCCTCTCTATCC 24 60.806 150

chr3 13683711 CATCACCCTCGAACTACCAAAATG 24 59.905 TAACCATCATGCCATATCGACAGT 24 59.96 228

chr3 13687129 TCCTTCCTCCAAACTTCTTCTCAG 24 59.959 CGGAGAGACTGGTGAATGAGTTAA 24 60.082 296

chr3 13696121 CCATGCCATTTTCATCCTAGGTTG 24 60.202 CCATACTTCAAATCCCAGTAACATG 25 57.969 281

chr3 13699087 GGATAGGCTTATGGTGATGGTGAT 24 60.019 GGGTAAGACAAATGCACAGAGTTC 24 60.083 249

chr3 13702105 TGGTCTTTTACTAGCTGGCTTCAA 24 60.202 TTTACTTGGAAACTTTACCTGCCC 24 59.414 281

chr3 13708423 GACAAATTCGGAGGTCTTACTGTA 24 58.283 CGTCTTCAATATCGTTCTTCACCG 24 60.028 210

chr3 13709655 AAGATGTGAGCCAATAACCCTTCT 24 60.018 TGGTAAGACTTTGAAGCAGTGAAG 24 59.184 271

chr3 13711707 AGAGAGAGATGAGATGGTGGGTTA 24 60.079 ATGGTTGAAGTTATGAGTTGGGAC 24 58.99 237

chr3 13712066 CGAATGAGACATAGTTAACCAATCC 25 57.821 CGATTTACACGTGTACTAGCAACC 24 59.969 283

chr3 13712940 CGTGAGTTTGAGCATATCTGATTGA 25 59.479 AAATTGTAGCTTTCTCCCCAGGAT 24 60.017 209

chr3 13714130 TATATGCTTCTTTCGTGGTTAGGC 24 58.946 GGCTATCAAACCATGCTCATTGTT 24 60.142 298

chr3 13714352 ACAATGAGCATGGTTTGATAGCC 23 59.617 TCACCCTCAAGAGAATTTGGAGAG 24 60.02 142

chr3 13721910 AGTAGGAATAGGATGCGAGTTTGT 24 59.594 ATCAGACGTTTAGGCCAAGTAGAG 24 60.142 276

chr3 13724966 TTCAACGCATAGCTCTTAAGTCCT 24 60.082 TTCACCCGTCATCTGCATATAAGT 24 59.899 279

chr3 13732733 CTCCAAATACAAACCAACAAGTCCA 25 59.932 CCCGACGTTCTGACATTTCTTAAG 24 59.908 252

chr3 13745956 GAACACCAAGAATGTCATGAGCAT 24 59.843 CTCGGCTCCTAGGTTGTACATTAT 24 59.719 266

chr3 13747640 TGGTAATCCCTCCACAAAGTTCAT 24 59.956 TTTAATTCCCACCATCTTGCACAC 24 60.022 259

chr3 13754207 CCATTTCATTTTGTAGCATCGGGT 24 60.142 TACAACTTAGGAAGCACTCAATGG 24 58.754 236

chr3 13783455 GAGGTGATGAGTTTAAGAAAGTGAC 25 57.877 TCCACATATTCCACTATTCTGCACA 25 60.105 142

chr3 13784696 TACATGGGTTAGCATTCTGGAGTT 24 59.773 AAGGGTGGAAGAATAGTGATGCAA 24 60.264 282

chr3 13785895 ATGCTTTCCAACAACTATTCCAGC 24 60.082 GTTGGTCTCACTCAATCTCACTCT 24 60.022 282

chr3 13789755 AGATCTCATTTGCTAGCAGTGTCA 24 60.081 AATCCTTGAAGCTTCTTTGGCATG 24 60.322 202

chr3 13798813 AGCATGAAGTTGGAAGAATGAAGC 24 60.082 CACATCATACAACATGCAACCAGT 24 60.083 228

chr3 13799247 AAAATTACTCCAAGCTAAGCACGC 24 60.379 GAAAGTTGGTAGGCATGTAACACC 24 60.083 296

chr3 13799510 GGTGTTACATGCCTACCAACTTTC 24 60.083 TTTTGGTTCGAATCTTGGGCCATA 24 61.054 113

chr3 13800960 TTAGCTGTGTCATCTTATCTCGCA 24 59.902 GCTTCATTTCGTTCTTCACCTTGA 24 60.024 205

chr3 13801673 GCCTCTACCTGATTGTATGGAGTG 24 60.502 AACGTACGACCTTACATCTGGAAT 24 59.841 249

chr3 13803234 TATAGCGGATGAGTTTTGAACCCA 24 60.081 TTCGATACACCTGATGCACTACAG 24 60.44 275

chr3 13803441 CGCCCATGATATCAAGTGCATTTA 24 59.784 GAAACTCAGCTCACACATCAAGAG 24 59.848 272

chr3 13813174 TAGGAGTTGAAACAACGCAAACTC 24 59.965 TGTAGAGCAAGAGTCACTGAATGG 24 60.322 288

chr3 13816140 ACTGTTCAAGACTCATCAGACAAC 24 58.947 TCGCAACCTTTCAACTCTAAAACC 24 59.964 226

chr3 13818631 TGGCGTCTTACTTGATGTATGGAT 24 59.899 TCGGTATTCATTCAGATTCTGCCA 24 60.142 269

chr3 13825126 CAAAGAAGAGAGGGAAGAGTGTGA 24 59.96 CCTCCCTTCTTTTAATGTCCTCCA 24 60.018 281

chr3 13835750 ATCGTATTGTGGGTTGTGAGATCA 24 60.081 AGGTTAGATATGTTGTCCGGTGAA 24 59.531 153

chr3 13838416 GCTTGGAGAGGTTGATTATTTTGC 24 58.895 TCATTATTGAAACCACACCCTCCA 24 60.203 211

chr3 13887343 TAATCCTCCAAGAGTCATGCTACG 24 59.962 AGAAGGAACAATCGAACACGGATA 24 60.082 297

chr3 14125970 CTGGTTGGTTTGTGAAGATGACTC 24 60.024 CATGCATCGTGATAGGCCAAAATA 24 59.784 242

chr3 14167625 GAGTGACGAACAAGATAATGCTGG 24 59.967 GAGGTTAGTATGCTGGTTGATTTTG 25 58.445 297

chr3 14183192 AAACTAGTCGTCAGCCACAAAA 22 58.462 GAGATGGTGGTATAACGGCTAAGT 24 59.961 158

chr3 14184922 AGTACCCAATGACCACTACAACAA 24 59.897 CAAGGACAGCTTGTAGGAAAGAAG 24 59.546 196

chr3 14190378 TGTTTCTTGATCCAATGGGCTACT 24 60.264 TAGATTATCCTATACCCCAGACGA 24 57.353 239

chr3 14190775 AGAAAAGAAGGAGATTGAGAGGGG 24 59.773 CAAGAAATCATGAAACCCGACGAA 24 60.084 213

chr3 14191543 AGGACAAAATATCGCACATTCACG 24 60.202 ACATTATTTGAGGGGATGTTGGTC 24 59.044 127

chr3 14197515 ATTCTACTATGGAGGTTGAGCTCG 24 59.72 CATTCTCATACCCCATTGCACATC 24 60.023 271

chr3 14198391 TCCCTTGATTTGTACGAGTGAGAG 24 60.082 TATTCTCAGCTCTCCACTCGATCT 24 60.446 111

chr3 14198746 GTTGTTCATTGGAGGAGCATTGAT 24 59.841 TCTTTACGGACTAGGAAATGGACC 24 59.839 285

chr3 14200225 AATGAATGTTTGACCTTCTGCTCG 24 60.083 TTCTCTTCTTCAGATAGCGTCCTC 24 59.663 196

chr3 14204366 CACTCTAAAACATGTCCCGAAAAGT 25 59.761 CTCTTTCTCTTGTCACTCCAGGAA 24 59.96 226

chr3 14204592 TTCCTGGAGTGACAAGAGAAAGAG 24 59.96 GTGAGTTACCTGAGGCAATTGATG 24 59.905 182

chr3 14206984 TGGTTCTTTTGGGTTCGCTTATTC 24 60.023 ATATGGCCTCTCTCTCTCTCTCTC 24 60.019 252

chr3 14207557 TTGTCCTTCTCCTTCACTTTGGAT 24 59.895 CCCAAATGACTTAAACCAGCACAT 24 60.021 190

chr3 14208790 GAAGATCCTTAACCTGCTCCAAAA 24 58.99 TGGGTCTCTCTGTTTCTTCTTCAG 24 59.96 261

chr3 14210275 TCCTTATCCCTAATCAAACCCCAC 24 59.833 CAGTAATGGTAGTTTGTAATGACGC 25 58.525 243

chr3 14214780 TGCGATGTTCTTGTCCTATAGTCT 24 59.355 ACAAGTACTCCCACTCAACATACC 24 60.02 131

chr3 14220236 GATACACCGATGCTAGAGGAAGG 23 60.057 TATGTGCATCGTTAGCCCTTGATA 24 59.96 175

chr3 14344221 CCGAACCTCTCTCCACTCTATTAC 24 59.724 CGGAAGTTGGATCTTGTAGCAAAA 24 59.785 278

chr3 14348549 GCCACTTCAAACAAATCCTCACAT 24 60.262 GGGTGGATTAAAATGAGTTAGCGG 24 59.964 191

chr3 14349644 GTTCTCCTTACTCGTAAATGTGCG 24 59.969 GGTGACATCCCATCCATTCATTC 23 59.429 288

chr3 14350600 ACTAGTATTGTTGGGAATTGGCCT 24 60.017 CCAACCCAATCCGAAAATAACCAA 24 60.021 288

chr3 14353437 CTCAAACTCATCTTAGCTTGCCAG 24 59.905 ATTTTAAGTCAGGCGAGGAAGAGT 24 60.02 239

chr3 14353864 CGTTTGTAGATTCTGACTTGCCAA 24 59.787 AGCTTCTCAATGCCTAAGTTGTAG 24 58.818 199

chr3 14359015 ATCCATAGACCCAGCATATACAGC 24 59.837 AAGTACATTGATCCGAATCCCCTT 24 59.834 244

chr3 14360191 GGGGAAAGATATTAGACCAAGCTCT 25 59.928 TGCTCTGATACCATATCAAAGGGA 24 59.093 274

chr3 14360824 TGCGAAAAGACCTTGTGACTACTA 24 59.962 GTGGTAGAGGGTCAATAAGCAGAA 24 60.081 240

chr3 14370397 AGAGAAACCAAAGGCCAAATCTTG 24 59.96 ACTTCCACTACACCTTGAAACCTT 24 60.08 149

chr3 14371611 GCACACTAATGGATTGACATATGGA 25 59.235 CAAGTGATATTTCTACGAGGGGAGT 25 59.931 233

chr3 14385145 TCCACCATAATATTGCTTGCATCTG 25 59.758 CCCACACGATTCAACTATATATCCAC 26 59.41 238

chr3 14386660 ACTAAACTAAGTACCCCACACACC 24 59.959 GTTTGAGAGTAATGGGCAGATTGG 24 59.903 300

chr3 14391602 GATGATTGATGGAAAGACGTGTGG 24 60.202 AGTTAGTGGGTCAAAGTTTGGGAA 24 60.326 153

chr3 14396157 GTTGAGTACTAGTGGTGGTCTCG 23 60.12 GATAGAACTAAGACATGCACAGCG 24 59.792 177

chr3 14396765 CATGCTATGTGATCTTGAATGGGA 24 58.932 CTCAAGCGTTTACCACACTCTTTT 24 59.965 199

chr3 14402867 ATTTGGGTTGAGTTATGCCAAAGG 24 60.02 CGTCGTTTTCATCAGTTTCATCCA 24 60.084 221

chr3 14403235 AGTTGTCAACCCTCATGAGATTGT 24 60.203 CATCTTGACAAACCACACTTAGGG 24 59.784 292

chr3 14403909 CCCAATCATCTGCATTCCACAAAT 24 60.142 TCATGCACCAATTCTCTCAGAT 22 57.501 209

chr3 14411208 CACCACAACTTCACATCTTCAACA 24 59.904 TATTTTCGAGCTACCCTGGATGTC 24 60.202 277

chr3 14412403 ATGACGTCTTCCATGTGAACAAAC 24 60.024 GCTCGTATTGGTTGAAACTTAGCT 24 59.608 288

chr3 14412878 ATTGGGCTTTGGGATTAAAATGGG 24 60.08 GTTGAAGCTCATCGATCTCTTTGC 24 60.494 287

chr3 14416474 ACAATTCACGCAAAGAGGAAGAAG 24 60.024 TTTCTTCCCCTTATCTCCATCGTC 24 59.899 174

chr3 14420606 TTCTATACACTGAGATGGAGGGAC 24 58.859 CCTTTCCTCCTTAAACAACGCTTT 24 59.962 258

chr3 14421142 AGAGACCAAGAAAGAACCCAAGTA 24 59.341 TTAGGCACGGATCAAAACATAAGC 24 59.904 289

chr3 14421872 AAGAGAACAAGTAGGTGTGTGTGT 24 60.081 CAACAAAGAGGACAACGTGAAGAA 24 59.906 237

chr3 14422628 CTCCAACCTTGTTATTGTATGTGCA 25 59.817 CGAGGTTTTAGGTTCACGTTTACT 24 59.253 300

chr3 14438410 ATTATCATCCTCTCCCAAAGCGTT 24 60.141 GACCAAAAGTTGAAGGAAGGAAGG 24 59.962 252

chr3 14439083 ACCATGTGTGTCCATATCTTTTGC 24 59.842 TCACCGATGTCTCATCCTCTAAAC 24 59.902 285

chr3 14439269 ACCATGTGTGTCCATATCTTTTGC 24 59.842 TCACCGATGTCTCATCCTCTAAAC 24 59.902 285

chr3 14439412 TAGGGGTTGCTACTCTGGTTACTA 24 60.017 CTTACAAAGACTCACCATCCGCC 23 61.476 128

chr3 14442036 GGTCTTCAATGCTGACGTAAAGAG 24 59.908 TCGACAAAAGGGTTTACAAAAGGG 24 59.902 287

chr3 14457903 ACAACACACTAAGGATTCAGGTCA 24 59.897 AGAGCGGGAGTATTCTACACAAAT 24 59.594 143

chr3 14463090 TTTGTGAGAGGGATCAAGGTTGTT 24 60.386 TTACGATGATCTTTGAATGCCTGC 24 59.964 232

chr3 14463317 GCAGGCATTCAAAGATCATCGTAA 24 59.964 CCCGCCCACAACAAAGTTTATTTA 24 60.262 244

chr3 14484804 CCTTTGGTCAAAATTGCAAGGTTC 24 59.727 GTCCATGCCTTTAACTCAAGCATT 24 60.082 224

chr3 14488330 AAAACTGCCAGACCTTTAACCATG 24 59.961 CCTCAACCGGCTACTTATTCTCAA 24 60.383 233

chr3 14489143 CGGTGATCCAATCCAACAAGTTTA 24 59.542 TTCCTAGACGACGAGTTTTGATGT 24 60.023 233

chr3 14492948 ACAGCTTTTCTCAACGCCTTATTC 24 60.083 GTTCCTTCGACTACTTCTGGCTTA 24 60.082 234

chr3 14493100 TAAGCCAGAAGTAGTCGAAGGAAC 24 60.082 TGATCGTGAAGAAGAAGGAGAAGG 24 60.082 115

chr3 14493459 ACCAAAGTCAATTCTCTCGTCTCA 24 59.961 GTGGGTAGAGGGTAAACACATTCT 24 60.02 230

chr3 14493884 ATTATACTTCTTGTCGCTTGCACG 24 59.967 AAGAGAGGCCCTAACTTGAGAAAG 24 60.02 268

chr3 14495299 CGAAGACCTCATAACTCTACACCC 24 60.202 TTCAAGGCAAATCACTCAAGTTGG 24 60.202 239

chr3 14499383 GTTAGTTGAACGGCGCTCATAAAC 24 60.954 TGATAGAGGTCCATGCTCAAGTTT 24 59.774 157

chr3 14502283 CTTCTCTCTCTCTCTCTCAAGGGA 24 60.08 CGTAGGAGATGCATGATGGTTCTA 24 60.022 214

chr3 14507019 ATGCCAAAGAGCTAAAGGAACAAC 24 60.022 CTGCACTCCAAACACAGACTATTG 24 60.084 250

chr3 14507860 AGGCCGAGATGACCAATTTACTAA 24 59.837 AAGTGAGAGAAATCGAGAGGGAAG 24 59.84 229

chr3 14510026 TGTTGGTGCCTCATGATTCATACT 24 60.324 GTTCATGTTCGATTTGGTTGCTTG 24 59.851 234

chr3 14510155 TGTTGGTGCCTCATGATTCATACT 24 60.324 GTCAGAGCCAATGGATATATGTTCA 25 58.767 254

chr3 14517673 CTGCCTTCCTTCCCCATATTTCTA 24 59.895 GAACCAATCACTTTCAGATGGACC 24 59.843 150

chr3 14525346 TGGATCGTACACAAGAATCACAGT 24 60.021 GAATGTCGGAATGGCATACTTCTG 24 60.025 255

chr3 14526471 AACCTTTAACCCTGCAGTTTGAAG 24 59.901 GAAAGTGACCATAAATTCTGTGCAC 25 59.141 225

chr3 14531080 CTTTGGGAGTCTGAGATTTGATCC 24 59.119 CGGTGACCAAATTTCAAATGTCGA 24 60.556 290

chr3 14531871 GCCATGACACATAACGCTAAATGA 24 59.965 TCTATTTGAAATCGCCACCCTACT 24 59.837 274

chr3 14544999 CCATGTGTCCGACTTGTTACCTAT 24 60.383 ATTGAACTAGAAGGGTGAGACGTC 24 60.082 290

chr3 14545410 ACGAGTCTTAAACAAACCCTAAGC 24 59.246 AGTGGCTATATGTTAGGGTTTGCA 24 60.08 126

chr3 14546009 CTCCCCACAATAAAACACATCTCA 24 58.99 GGAAGAGTAAGAGGAAGAAGCCAA 24 60.02 292

chr3 14560520 CAAAAGCACAACTCATTCCCTTCT 24 59.962 AAAACAACATCCCTATTTCCCTGC 24 59.777 280

chr3 14560855 CCTTTTGACTGTCTGCAATTTTGG 24 59.491 TAATGTGTACTGGTTGGAGTTTGC 24 59.483 236

chr3 14572798 TCTCCGTCCGATTTCTCTTCTTTT 24 60.021 TAGTGGTAAAGGAGACGAACGAAA 24 59.722 216

chr3 14572953 TTCGTTCGTCTCCTTTACCACTAA 24 59.722 ACCGCAATTTTGGACATACATG 22 57.891 272

chr3 14574187 TCCTCCCACCTCAAACTTTATTGT 24 59.894 ATCCTCCTTTTGCCTTTCATTTGG 24 60.02 256

chr3 14577815 AATTGGACATGCTTGGTTGGTAAG 24 60.021 TGATCTTGGCCATCCTAAACTCAA 24 60.019 201

chr3 14581188 CCATGTGGGCAAACTGACATA 21 58.555 CAGAGTGACGATGAAATGGAAAGG 24 59.906 186

chr3 14594402 CCTCTTTTGAAATGATCGATATGGGG 26 60.072 GACACATCAGACTAGTCACCACAT 24 60.082 204

chr3 14594564 ATGTGGTGACTAGTCTGATGTGTC 24 60.082 ATGATGATGCACACCTACTCACAT 24 60.142 224

chr3 14595630 CGAATAAAACGTGGATAGAGAGCA 24 58.959 GACAACCACTAGCTGAATGTGC 22 59.84 298

chr3 14597002 ACCCTAAGTCCAAATATGCTTTCCT 25 60.044 CACGCTTCAAATTTGGGTTTAGGA 24 60.262 239

chr3 14601389 GCTAGGTTGCATCTAAGTTTCATAC 25 58.051 AGGGATAAGTGTTCTAAAGGGACA 24 58.901 147

chr3 14610204 AATCAAACAACTCCAACACGATCC 24 60.023 AAAGATTGGTATTGTCGGAAGGGT 24 60.264 130

chr3 14612786 CAGAAACTTGGTCGACATTTGA 22 57.188 CTTCCTCCCACTTTCTTACCATGA 24 60.019 145

chr3 14614383 CACCTCCTTAACGGCTTCCTAATA 24 59.9 TACCAATTTGATCGGAGCCTACTT 24 59.837 291

chr3 14616164 GGTAAGTTCAACCGCCATTTAAAAG 25 59.368 CACCAACTTCTACTTCTTCTATGAC 25 57.18 152

chr3 14695326 TGGTTCTTTCCCATAGCTTCAT 22 57.409 CTGGTGATCAAAGATATTTCGTTCC 25 58.281 262

chr3 14697195 CCCTATAGGCATACTTTTCTAGGAT 25 57.258 CATGTTGCCACTTCTGACTCAAAT 24 60.023 187

chr3 14702263 GAAGTAAACTAAGGGCGAAAAGGT 24 59.241 GAGATGGTCGGCATAAAACACATT 24 59.904 197

chr3 14715151 ATTCACTTCCGAACCTCAACCATT 24 60.75 TTCTGACTCCTTTTAATCTCGGGT 24 59.469 291

chr3 14722744 AGAAGACAATGTTGAAAGAAGGCG 24 60.024 AAATGTTGGGAATGTTGAGCCTTC 24 60.263 123

chr3 14723965 TGTTCTTGAAGTTGGAGTCTTGGA 24 60.081 GACCAGATCGACTCAATCCCATAA 24 59.961 291

chr3 14743790 CTTTCTAAATCTTGTGTTGTGCGC 24 59.62 AGACCCGTAATTCATCTCACTAGC 24 59.962 264

chr3 14879010 TTTGGGCCCATCAATAACAAATCG 24 60.382 GAAATCCTACCAAACCAAAGAGCA 24 59.477 263

chr3 14881827 ATGAATCCTCTCGACTGCAGTAC 23 59.933 ATCTTCTCCTAAAAGTCACAGCCG 24 60.622 288

chr3 15252567 AAACTCAACCTACATCCACTTTCG 24 59.245 GAGAGCTTCTTATTGTATTGTGAGG 25 57.524 251

chr3 15341187 GAAGAATGTCTGAATGGGCACTTC 24 60.143 AGTCTTTCTCTGCAGCTTTGAGTA 24 59.96 97

chr3 15430168 ACACCGAAACGACTATGATTGGTT 24 60.802 GTGAGGGTTTATGATACGATGACA 24 58.402 246

chr3 15451461 TCCTAGCAAGTCATAAAACCTCGT 24 59.778 TCGTGGTTGATAGTGAAGTTGACT 24 59.961 264

chr3 15456814 TATGTATCGTGGACAGGGAATTCC 24 59.96 TATCCAAAGTAGACAAGCAGGCAT 24 60.08 260

chr3 15465758 CGGAAAACCACCTAACTAAAACCA 24 59.42 CTTGCTTGGACCATACTGTTACCA 24 60.806 174

chr3 15475319 TGAAAGTTAGACGTGGCCAGATAA 24 60.021 AAAACAACCCCAACACTTTCTACC 24 59.839 261

chr3 15486577 GAAGCAAACTCCCCTTAGAAAACC 24 60.022 AGACTTTAGCTTTTGATGTGGGGA 24 60.203 177

chr3 15492294 TCCTTATACCCTCCAAACACATCT 24 58.963 GCTTCTCACCCACTTCCTTAAATT 24 59.233 278

chr3 15493366 ATAGCCAGCTTCGTTTCCATAGAA 24 60.142 TTTTACTTTGCAGCCTTGAGTGAG 24 59.963 254

chr3 15500794 GAAAACCATACTCAGTAGCAAGCG 24 60.202 CCCAATTCTTAAACCCAGCTCATG 24 60.142 183

chr3 15501077 AACAGCTCACCTAACGATCAAAAC 24 59.787 GTGATTCCAACCAAATTCCCACAT 24 60.02 243

chr3 15512210 ATACAATTGTGAGGTTCGAGTGGA 24 60.021 TGGAGCCTGTTTATGAGACATGAA 24 60.019 188

chr3 15519833 ATGTTGGTGTTAATGTTGGACGAG 24 59.785 ACCTCTTTCAATTCCGAATCCA 22 57.699 211

chr3 15520630 GCTGCTTTAAGTTGCTTGGTCTTA 24 60.023 CCCCAAGGTAGAAATAGCAGAACT 24 60.08 282

chr3 15529924 CATACTCGCCCTTTTGTTTTCTTCT 25 60.049 GCGATTGGGGCAAACTGTATTAC 23 60.489 252

chr3 15551923 ACACCCCTAACTCTACTCAATTGG 24 59.775 CTTGCAGGGTGACAAATTTTCTCT 24 59.962 256

chr3 15552712 CGCTATTCCGAACCTACAATGTTG 24 60.26 CATGAACTGGATGTCAACTGGTGA 24 61.044 264

chr3 15553456 TGTCATTAGTTTGTAGACTCTGCTC 25 58.609 AAGAAGAGAGCAGGGTATGAAACA 24 59.713 182

chr3 15557678 GAGGGTTTTGATGAACTGTTGTGT 24 59.902 GTTTTCCTCACTCTCACATTATTCC 25 57.922 139

chr3 15581913 TGGCCAATCTAACCAACTTCTCTC 24 60.566 CTGATCGTCGATGTTTCAAACCAA 24 60.084 272

chr3 15587753 CTTTTGGATGGAATTCGACGTTGA 24 60.084 GGTTGGGTTAGATTTGGTTGTCAG 24 60.022 297

chr3 15590062 TGGAAATTATGAAGACGTTGCTGG 24 59.844 GGGTCATCCTTGTTTCCTTTACAT 24 58.983 270

chr3 15611747 CTCTCCTCTTTGACTCACTCACTC 24 60.083 AAACAGTGCTAATGTGACATTCCG 24 60.083 295

chr3 15635392 TTCGAAAACATACTGGCTTCCA 22 58.254 GAAGTGTGATATAACAAGGGAAGCT 25 58.884 260

chr3 15637948 TCATATGCGTCCCCTTTAGAGATG 24 60.021 CTTGGTCTATGGCCTTCTACCTAC 24 59.961 146

chr3 15638150 ATGCCAAGAAGCTCACTCTTAGAA 24 60.02 GCAACTCTAATTTTGTTCTTGGCC 24 59.314 166

chr3 15642153 TATGGTTGATTGGGATAGGACAGG 24 59.649 ATGTAGACGAGGAAGAAGATGCC 23 59.931 300

chr3 15642432 GCATCTTCTTCCTCGTCTACATCT 24 59.963 CGAGATCACACTTTCATCCAAAACA 25 60.049 85

chr3 15643003 GTTATAGGGTTTGCTTTGGACACC 24 60.082 TGAAGAGAGTGGAAGAAACGTCAA 24 60.142 296

chr3 15644027 ACCCCAGTACATTTCTATCACCAC 24 60.081 AGTTGTGAGGTTTCTATCTCTCCT 24 58.662 296

chr3 15648167 GCGAAATAGTGCTCCAATCAGTAC 24 60.026 GTGAGCTGGCTTCAAAAGTGTAAA 24 60.202 246

chr3 15662810 GAGATTGTTCAGGGCAGCTATAGA 24 59.96 GCACCTAGTTGACTTACCCTCTAG 24 59.902 222

chr3 15683202 AGAACGAAGGGAGAATTGGATTGA 24 60.019 TCAAACAAGAGTCGGTAGTGATGA 24 59.72 282

chr3 15687255 ATCACCTTCATACCACAAGTGACA 24 59.958 CCACAACTTCAATCAACCTCTCAC 24 60.024 271

chr3 15692962 CTCGGATTGCCTTACTTTCCTTTC 24 59.905 CTCTTTTCTCCTCACAAATCGCAC 24 60.378 245

chr3 15695828 ATATGATTTGAGGCAGACAGGGTT 24 60.08 GCCTACGGTTTGAACACAATACTT 24 59.785 278

chr3 15702500 AGATTTTGAAAAGGGAGTGTCGTG 24 59.724 CCCTCTCTTCCTCTACCTATCACA 24 60.141 170

chr3 15703731 GGTTTGAGAAAGAAAAGACAGGCA 24 59.902 GATAAGGGGAAAAGGTGGGTAAAA 24 58.665 300

chr3 15706933 ATGATGGAGAGGAGGAAGAAAAGG 24 59.834 CAGCTATTTTGTTCATCAGCCCTT 24 59.841 184

chr3 15711920 TGTCGCCAAAAGGATATTGTTTCC 24 60.082 TCTCTCTCTCTCTCTCTCTCTCTCT 25 60.104 87

chr3 15715352 TATGCTTATTCTCGGTGGTATGAG 24 57.974 GGAACAAACAACCTAACCTCCAAG 24 59.962 263

chr3 15725761 GCTCCACTCGAAATTAAAGTTGGT 24 59.784 GTCGGAAAATGAGAGGTTGGTTTT 24 59.962 258

chr3 15801141 TGAAGATCTGAAGGTCACGTGTTC 24 60.796 AGTCAATCACGTGGCATAAGAAAG 24 59.607 262

chr3 15847414 CATGCTTGAAAGGGAAAGGGAAAT 24 60.02 TTTCCCATCGACATAACCTTTTGC 24 60.082 268

chr3 15867224 AAGTTTCGCATTGCAGTCTGAAG 23 60.304 GTGGGATAGTATGACATAAGGAGCT 25 59.518 296

chr3 15868872 TTTTCTTCCTTCAAACGTTCAGGG 24 59.903 GTGAGAAAGAAGGAACGTAGAGGT 24 60.022 284

chr3 15871782 AGTAGAAGTTGGTGGGGAATGTAC 24 60.02 CGATTCTTCTCTTCTATGCTTGCG 24 60.085 258

chr3 15872030 CGCAAGCATAGAAGAGAAGAATCG 24 60.085 GAGGAAGAAAGGGGAAAAGGGTAT 24 60.017 291

chr3 15880871 TGTCACGTAGAGTTCTTAGAGCTT 24 59.239 TGCTCAAGTTAGTACAACCCTAATG 25 58.829 273

chr3 15882271 CAGTTAGTCAGGTACATGTAACACA 25 58.548 TGAAGTCTCCATGGGAAGATTCAA 24 59.711 179

chr3 15885529 CATGAGGAAAACAATGGCGAGAAG 24 60.674 ACCTGTTAAGCTCTTGTGGACTAG 24 60.021 246

chr3 15889874 TCATTGAAAGACCGTGGTAGAACA 24 60.202 AGCTTCAAACAACCTAACTCACAC 24 59.664 219

chr3 15893103 GGTACTTTAGCTGGCCTGATTATA 24 57.89 GTCACAGACGTTAATAGAAGTTAGG 25 57.262 190

chr3 15897231 ATGCCCTAAAACTCCACCAACTAT 24 60.017 ATCATAGCAGCCATTGTTCTCTCT 24 59.897 134

chr3 15901004 TTTGAGAGGTGTTGGAATGAGTGA 24 60.142 AGCCTGCAGTGAAATAGACTTGTA 24 60.02 195

chr3 15938901 GCGGATATGAAGGTTGTTACTGTG 24 59.966 TCCCACCATGATAACGTACGAAAT 24 60.142 262

chr3 15939469 CCCCTCTAGGTAGTGATAAGGACC 24 60.754 CACCACACACATCCAAACGAATTT 24 60.736 210

chr3 15942513 CAATTTCCAATCCACCTGAACCAA 24 59.96 TTCTAAACCCAACTCCACTACCTG 24 59.959 285

chr3 15943220 CTTCTTTTGGCTTCTCTGGAGTTG 24 60.023 AAAGGAAGAATAGAAGGCTAGGGG 24 59.586 153

chr3 15943488 ATTCTTTCAACATCAGCCAAGGAG 24 59.538 TGGAGTTTCAAGAGGTGTTGGTTA 24 60.08 239

chr3 15950737 CAATGAGGCTTGATAGAGGTCGA 23 59.931 AATGCTTTCGTTGGGGTATGAATG 24 60.142 86

chr3 15957174 TCAAATGATCATATTGCCCTTGGAC 25 59.697 AGTTTACCCGCTTTTCTTTAGAGC 24 59.545 296

chr3 15975133 TACAAACACTTCCGCTTTACATGG 24 59.785 ATGTCCTTTGCATCATTGTCATCG 24 60.202 230

chr3 15983923 TCAACCTTCTTCTTTACTTGGCCT 24 60.141 TAGAGAACGATTCAGGATCCTTGG 24 59.658 258

chr3 15994490 ACAACTTAGTTCCAGCCATATCGT 24 60.081 GCAATTGGTTAGTTGGTGGGATAG 24 59.902 221

chr3 16001241 AAGAAGGCTCAGTTTCACTAGGAG 24 60.021 GCAACCAGAAAGACAGCAGTTATT 24 60.023 206

chr3 16004015 ATAGACTGCTCACCCATATCATTG 24 58.198 CCACTACAAACATGAATGAAAGTCC 25 58.618 259

chr3 16004534 TCTTGTAGCAATGGTTGAGAGGAA 24 59.958 AAAGAACCCCATCCACAACCATTA 24 60.45 196

chr3 16004720 AATGGTTGTGGATGGGGTTCTTTA 24 60.45 CCCATCCAAACTACAGAAGCACTA 24 60.323 142

chr3 16005880 TCGTCAAGGGATAAGGATGGATAC 24 59.473 CATACCCAAATTGCGACTACCTTC 24 59.965 206

chr3 16012600 TATATGCCAGCTCCACACCTTAAA 24 59.835 GGAAGAGATTGTGATTCATGAGCC 24 59.725 191

chr3 16013004 TAATGCGTACCAGTGATGGAAACT 24 60.323 AGGAAAGTTGCTTGGACATAAAAGG 25 59.989 80

chr3 16014729 GGCACACGATCATTTAAATTTGGC 24 59.967 GTAGCAAAATCTAAACGACCGTGT 24 59.849 151
[truncated: 643,544 more chars]
